# Harnessing Benzoyl‐Urea Secondary‐Sphere Hydrogen‐Bonding to Enhance Oxygen Evolution Catalysis by Cobalt Corroles

**DOI:** 10.1002/smsc.70283

**Published:** 2026-04-25

**Authors:** Rwiddhi Chakraborty, Sahanwaj Khan, Subhajit Kar, Tanmoy Pain, Biswajit Das, Pravansu Panda, Narayan Ch. Jana, Sanjib Kar

**Affiliations:** ^1^ School of Chemical Sciences National Institute of Science Education and Research (NISER) Bhubaneswar India; ^2^ Homi Bhabha National Institute (HBNI) Training School Complex Mumbai India

**Keywords:** cobalt corrole, oxygen evolution reaction, proton‐coupled electron transfer, secondary coordination sphere, water oxidation catalysis

## Abstract

Secondary‐sphere control is leveraged to boost cobalt corrole oxygen‐evolution catalysis. We install a pendant benzoyl‐urea unit on a corrole scaffold to position a hydrogen‐bond donor above the metal site. The ligand is obtained via an isolable N‐benzoyl‐dicyclohexylurea intermediate, converted to the FB corrole, and metalated to Co(III); a pentafluorophenyl analog lacking the urea serves as control. X‐ray/time‐dependent density functional theory (TD‐DFT) show axial pyridine ligation lifts the pendant carbonyl toward the catalytic pocket with reduced saddling. In MeCN, CV features reversible Co (III/II) couples at −0.62 V (urea) and −0.52 V (control) versus FeCp_2_^+/0^, plus ligand‐centered oxidations; spectro‐electrochemistry/EPR (*g*_iso_ = 1.9986) confirm a corrole‐radical [Co^III^(corrole^•2‐^)(py)_2_]^+^. Water addition unveils catalytic waves ~1.12V(urea) and 1.21 V (control), versus FeCp_2_^+/0^. Under CPE (1.78 V, vs. Ag/AgCl), the urea–bearing complex delivers 83.9% Faradaic efficiency**,** TOF 1.19 s^−1^ (vs. 59.6%**,** 0.47 s^−1^ for control); a KIE = 1.3 implicates PCET. DFT supports WNA at a Co‐oxyl, [Co^III^(corrole^•2‐^)(O^•‐^)(py)], with the pendant carbonyl H‐bonding to the incoming H_2_O (O···H = 1.71 Å), thereby lowering the activation barrier relative to a truncated analog. These results establish benzoyl‐urea secondary‐sphere engineering as a concise, general strategy to enhance charge utilization and O—O bond formation in cobalt corrole oxygen evolution reaction catalysis.

## Graphical abstract

Secondary‐sphere hydrogen‐bonding modulation in cobalt(III) corrolato complexes enhances electrocatalytic water oxidation. Introduction of a pendant benzoyl‐urea group in the secondary coordination sphere promotes efficient oxygen evolution reaction, achieving a Faradaic efficiency of 83.9% (TOF = 1.19 s^‐1^), surpassing the control analog (59.6%, TOF = 0.47 s^‐1^). Controlled potential electrolysis at 1.78 V confirmed sustained oxygen evolution, supported by structural, spectroelectrochemical, density functional theory, and GC analyses.

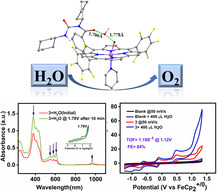

## 1 Introduction

**SCHEME 1:**
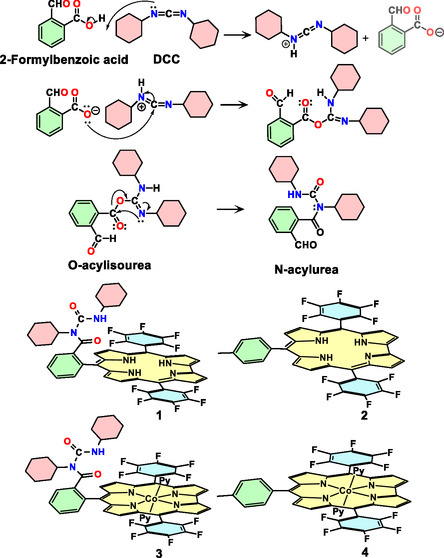
Schematic representation illustrating (top) the proposed mechanism for DCC‐mediated activation of 2‐formylbenzoic acid and the formation of *N*‐acylurea derivative (*N*‐cyclohexyl‐*N*‐(cyclohexylcarbamoyl)‐2‐formylbenzamide), and (bottom) the chemical structure of 10‐(2‐{N‐cyclohexyl‐N‐(cyclohexylcarbamoyl)benzamido})−5,15‐bis(pentafluorophenyl)corrole, **1**; 10‐(4‐methylphenyl)−5,15‐bis(pentafluorophenyl)corrole, 2; 10‐(2‐{N‐cyclohexyl‐N‐(cyclohexylcarbamoyl)benzamido})−5,15‐ corrolato cobalt(III) bis(pyridine) complex, **3**, and 10‐(4‐methylphenyl)−5,15‐bis(pentafluorophenyl) corrolato cobalt(III) bis(pyridine) complex, **4**.

Electrochemical water splitting is central to a carbon‐neutral energy future [1, 2, 3, 4, 5, 6, 7]. Its efficiency is limited by the kinetically demanding oxygen evolution reaction (OER) [8, 9, 10, 11, 12, 13, 14, 15, 16, 17]. Among first‐row metals, cobalt is particularly attractive owing to its redox versatility, structural adaptability, and natural abundance [18, 19, 20, 21, 22, 23, 24, 25, 26]. Early 1980s studies with simple cobalt precursors established cobalt as a water‐oxidation catalyst, while later heterogeneous systems and cobalt‐layered double hydroxides, borates, and MOFs demonstrated diverse redox pathways with improved surface area, tunability, and durability [27, 28, 29, 30, 31, 32, 33, 34, 35]. Molecular cobalt complexes complement heterogeneous catalysts by providing well‐defined platforms for mechanistic studies. Macrocyclic ligands such as porphyrins [36, 37, 38] and corroles [39, 40, 41, 42, 43, 44] stabilize multiple cobalt oxidation states, enabling access to high‐valent intermediates and allowing detailed investigation of O—O bond formation and proton‐coupled electron transfer processes [45, 46, 47, 48, 49, 50]. Building upon the concept of the primary coordination sphere, where ligands are covalently bound to the metal center, secondary‐sphere effects have emerged as decisive in OER catalysis [51, 52, 53]. Systematic studies have shown that modulation of urea‐ and sulfonamide‐based donors around Co‐OH units can significantly alter metal–oxygen bond parameters, vibrational features, and redox behavior, even when the primary coordination environment remains essentially unchanged [54]. These findings demonstrate that secondary‐sphere hydrogen bonding effectively tunes the electronic structure and energetics of metal‐oxo and metal‐hydroxo intermediates relevant to water oxidation. More broadly, pendant acids, hydrogen‐bond donors, and proton relays are known to accelerate catalytic turnover by facilitating substrate preorganisation and proton‐coupled electron transfer (PCET) [55], as exemplified by the cobalt hangman corroles of Nocera and co‐workers, where tethered carboxylates promote efficient hydrogen bonding and PCET in analogy to the oxygen‐evolving complex in photosystem II [56, 57, 58, 59]. In this context, a benzoyl‐urea pendant has been introduced to act as a rigid and strategically positioned hydrogen‐bond donor capable of stabilizing oxygenated intermediates while preserving the integrity of the metal‐corrole framework (vide infra). To introduce a urea‐based secondary‐sphere motif in a straightforward manner, we employed dicyclohexylcarbodiimide (DCC), a commonly used coupling reagent that activates carboxylic acids via transient O‐acylisourea intermediates [60, 61, 62, 63]. Although such intermediates often rearrange to unreactive N‐acylureas [60], this pathway was deliberately utilized here to isolate a stable N‐benzoyl‐DCU derivative, N‐cyclohexyl‐N‐(cyclohexylcarbamoyl)‐2‐formylbenzamide, formed in the absence of an external amine and providing a convenient entry to a preorganised urea functionality (Scheme 1). The FB corrole ligand (FB = free base), incorporating an N‐cyclohexyl‐N‐(cyclohexylcarbamoyl) benzamide moiety, was synthesized and subsequently metalated with cobalt(III) (Scheme 1).

SC‐XRD shows that the pendant N‐Benzoyl‐DCU is oriented above the macrocyclic plane, with steric effects from axial pyridines positioning the carbonyl group close to the catalytic center (DCU = dicyclohexylurea). Owing to synthetic challenges in porphyrinoid chemistry, systematic secondary‐sphere modification for OER remains limited. In this context, we report the first cobalt corrole incorporating a pendant N‐benzoyl‐dicyclohexylurea secondary coordination motif, demonstrating that precise urea‐based hydrogen‐bonding control markedly improves OER kinetics compared with a control cobalt corrole lacking this functionality, lowers onset potentials, and enhances turnover, thereby establishing a new bioinspired strategy for secondary‐sphere‐driven water oxidation catalysis. Thus, the pendant N‐benzoyl‐DCU group underscores how precise structural design, together with noncovalent functionality, enables efficient and durable water oxidation under mild conditions.

## 2 Results and Discussion

### 2.1 Synthesis and Characterization

Using the urea‐functionalized aldehyde precursor, N‐cyclohexyl‐N‐(cyclohexylcarbamoyl)‐2‐formylbenzamide, the FB corrole (**1**) was synthesized following the Gryko methodology [64]. Cobalt(III) corroles were prepared following established procedures [65, 66, 67, 68, 69, 70], with relevance to structural [71, 72, 73, 74, 75, 76], catalytic [77, 78, 79, 80], redox and spectroscopic [81, 82, 83, 84], as well as CO_2_ reduction [85, 86], O_2_ reduction [87, 88, 89, 90, 91, 92, 93, 94], and water oxidation studies [95, 96, 97, 98, 99, 100, 101]. Accordingly, metalation of FB corrole (**1**) in pyridine afforded the cobalt(III) corrolate (**3**), while the reference corrole (**2**) and its cobalt(III) complex (**4**) were prepared under analogous conditions following reported procedures [102]. All synthesized compounds N‐cyclohexyl‐N‐(cyclohexylcarbamoyl)‐2‐formylbenzamide, **1**, **3**, and **4** were fully characterized by UV‐Vis absorption, fluorescence emission, IR spectroscopy, multinuclear NMR (^1^H, ^13^C, and ^19^F), SC‐XRD, and ESI mass spectrometry (Figures S1–S24, Tables S1–S2). The NMR spectra of the FB corrole **1** and cobalt(III) corrolates **3** and **4** show characteristic aromatic, cyclohexyl, and pyridine resonances consistent with the proposed structures, while ^13^C and ^19^F NMR data confirm the presence of carbonyl and pentafluorophenyl units (Figures S15–S23). IR spectra display diagnostic aldehyde and amine stretching bands, and ESI‐MS analyses further corroborate the molecular compositions of **1**, **3**, and **4** (Figures S6–S12).

### 2.2 Crystal Structure

**FIGURE 1:**
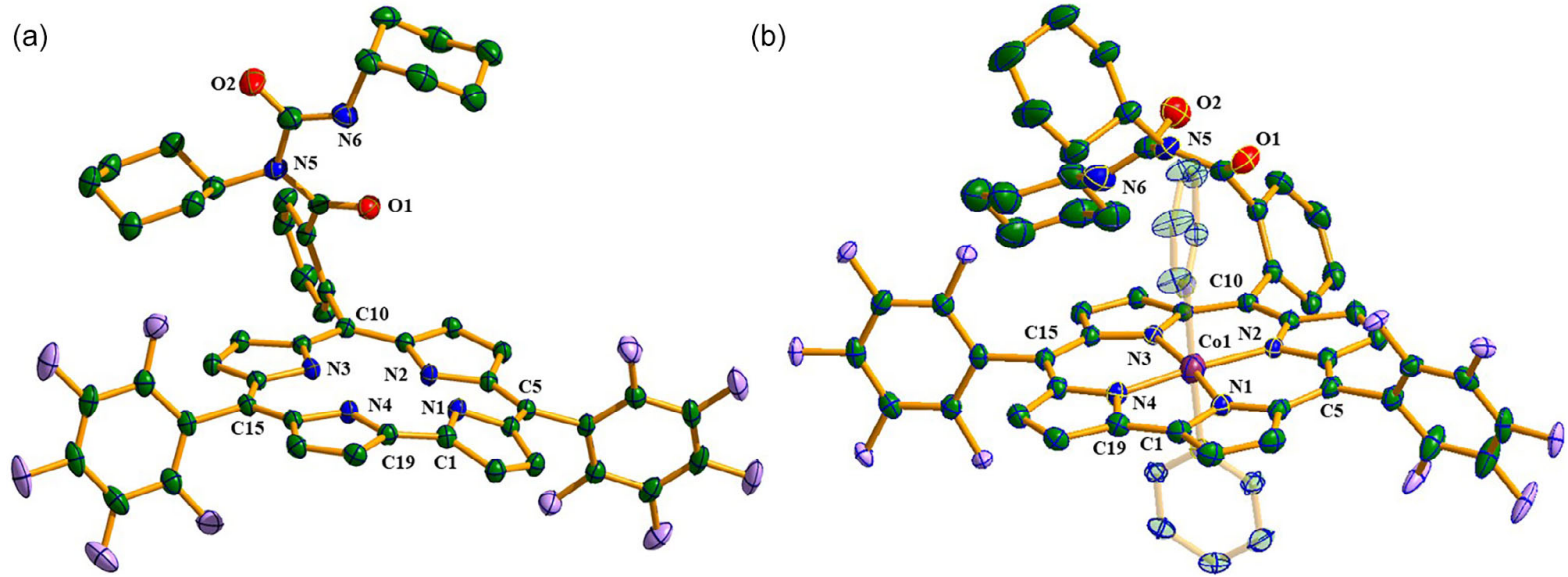
(a) Single‐crystal X‐ray structure of compound **1** and (b) single‐crystal X‐ray structure of compound **3**. Hydrogen atoms are omitted for clarity. Thermal ellipsoids are drawn at the 50% probability level.

The solid‐state structures of compounds **1** and **3** are shown in Figure 1, while that of compound **4** is presented in Figure S24; selected crystallographic parameters are summarized in Table S2. Compound **1** crystallizes in the triclinic system (Z = 2), whereas **3** adopts a monoclinic lattice (Z = 4), and **4** again crystallizes triclinic (Z = 2). In FB corrole **1**, the N‐cyclohexyl‐N‐(cyclohexylcarbamoyl)benzamide substituent projects above the macrocyclic plane, while in cobalt(III) complex **3,** steric repulsion from axial pyridine ligands displaces it toward the macrocycle periphery. DFT‐optimized geometries for **1** and **3** closely reproduce the experimentally determined structures.

Cobalt insertion in **3** leads to a pronounced reduction in macrocycle saddling relative to the FB corrole **1**, as reflected by a decrease in the dihedral angles C2‐C1‐C19‐C18 and C8‐C9‐C11‐C12 from 16.61° and 26.01° in **1** to −2.75° and 6.47° in **3**, respectively. Complex **3** adopts a distorted octahedral geometry with two trans axial pyridine ligands, exhibiting Co‐N bite angles of 81.31–95.05° and equatorial Co–N bond lengths in the range 1.87–1.90 Å, in close agreement with DFT values. The axial Co‐pyridine distances are 1.988–1.999 Å, with an N7‐Co‐N8 angle of 178.40°, consistent with near‐linear axial coordination. A similar structural motif is observed for **4**, which shows minimal macrocycle distortion (dihedral angles 1.03° and −4.63°) and comparable Co‐N bite angles (81.69–95.59°), equatorial Co—N bond lengths (1.87–1.90 Å), and axial Co‐pyridine distances (1.975–2.001 Å), with an N5‐Co‐N6 angle of 179.03°. Overall, the Co(III)—N bond lengths and coordination geometries of both **3** and **4** are in good agreement with DFT calculations and reported values for related corrolato cobalt(III) complexes [45].

Intermolecular interactions in the crystal lattice were examined by Hirshfeld surface analysis [103, 104]. The d_norm_ maps and two‐dimensional fingerprint plots (Figures S25–S30; Tables S3–S5) reveal dominant H···F and H···C contacts, which play a key role in lattice stabilization, along with additional F···H, C···H, F···F, and C···F interactions contributing to the supramolecular assembly. Comparative electrostatic potential maps (Figures S31–S33) further show distinct ESP distributions for **1** and **3** relatives to **4**, arising from the peripheral N‐cyclohexyl‐N‐(cyclohexylcarbamoyl)benzamide substituent.

### 2.3 Electronic Absorption and TD‐DFT Analysis

**FIGURE 2:**
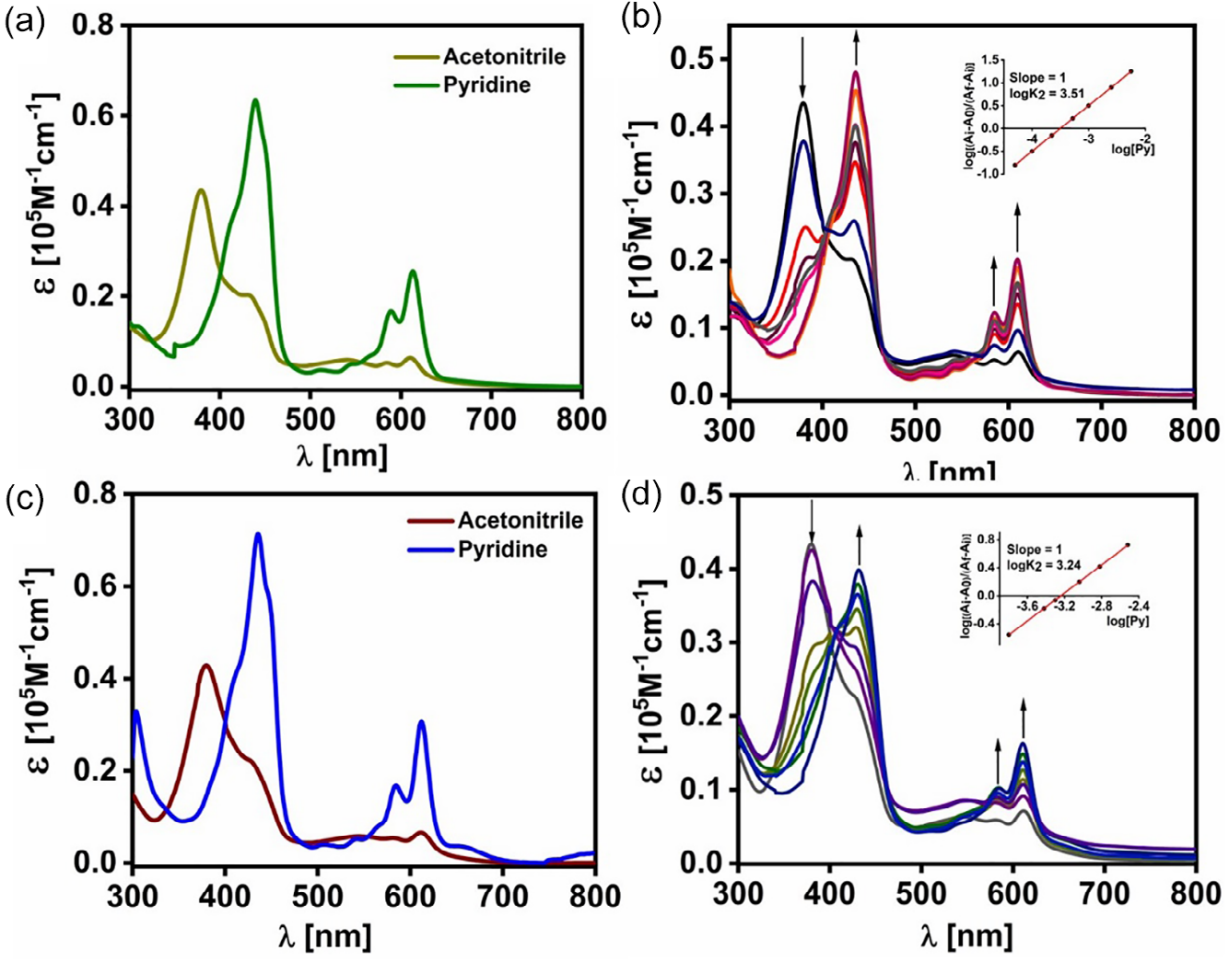
UV–vis absorption spectra of (a) corrolato cobalt(III) complex **3** in CH_3_CN (pale greenish yellow) and in pyridine (dark green), recorded at a concentration of 1.0 × 10^−5^ M. (b) Titration of complex **3** with pyridine; the inset shows the corresponding Hill plot used to determine the binding constant. (c) UV–vis absorption spectra of corrolato cobalt(III) complex **4** in CH_3_CN (brown) and in pyridine (blue), recorded at a concentration of 1.0 × 10^−5^ M. (d) Titration of complex **4** with pyridine; the inset shows the Hill plot used to determine the binding constant.

The electronic absorption spectra of FB corrole **1** in CH_2_Cl_2_ and its cobalt derivatives in CH_3_CN and pyridine are summarized in Table S1. Compound **1** exhibits an intense Soret band at 410 nm and three Q bands in the 550–650 nm region (Figure S1), with molar absorptivities of ~0.8 × 10^5^ M^−1^ cm^−1^(Soret) and 0.4–1.3 × 10^4^ M^−1^ cm^−1^ (Q bands). TD‐DFT calculated UV–vis spectra of **1**, **3**, and **4** show good qualitative agreement with the experimental profiles (Figures S1–S3 and S34–S36; Tables S6–S8). For **1**, the Soret band at 410 nm originates predominantly from ligand‐centered HOMO/HOMO‐1/HOMO‐2 → LUMO/LUMO + 1 excitations, whereas the Q bands (550–650 nm) arise from HOMO/HOMO‐1 → LUMO/LUMO + 1 transitions, as supported by TD‐DFT analysis (Table S6, Figure S37). The electronic absorption spectra of cobalt corrole (**3**) in CH_3_CN and pyridine (Figure 2, S2, and S4) show a Soret band at 379 nm with Q bands at 540–615 nm in CH_3_CN, and a pronounced redshift in pyridine to a Soret band at 440 nm (shoulder at 451 nm) with Q bands spanning 500–620 nm [75, 90]. The computed spectrum of **3** reproduces the experimental features (Figure S35, Table S7).

**FIGURE 3:**
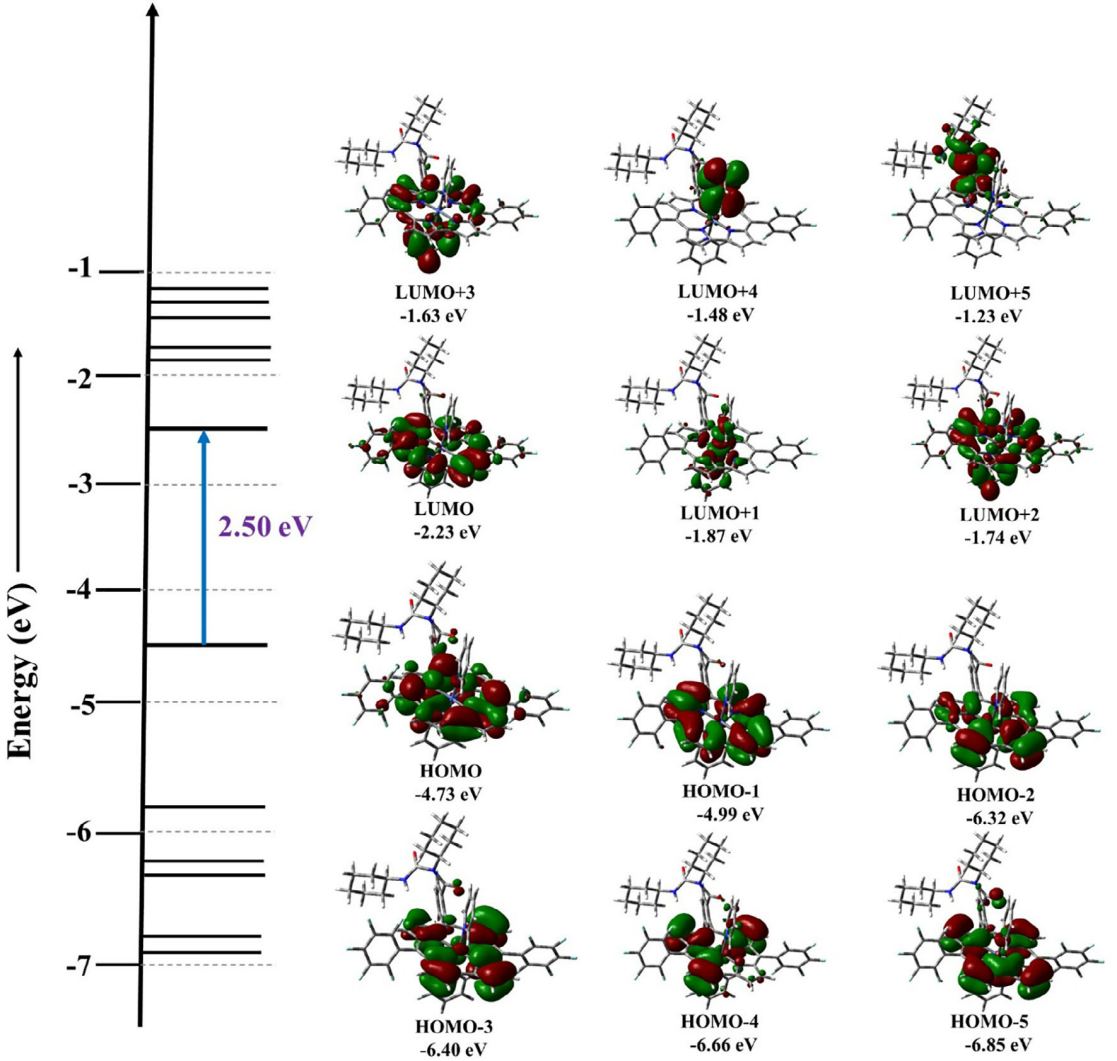
Selected Kohn–Sham orbital energy level diagram of corrolato cobalt(III) complex, **3** (iso‐value of 0.02).

TD‐DFT attributes the Soret and Q bands primarily to ligand‐centered π‐π* excitations with contributions from HOMO/HOMO‐1/−2 → LUMO/LUMO + 1/ + 2 transitions, accompanied by appreciable LMCT character arising from cobalt involvement in the low‐lying LUMOs, while MLCT contributions are negligible (Figure 3). The electronic absorption spectra of cobalt corrole **4** in CH_3_CN and pyridine (Figures S3 and S4) show a Soret band at 378 nm with Q bands at 545–615 nm in CH_3_CN, and a pronounced red shift in pyridine to a Soret band at 435 nm (shoulder at 448 nm) with Q bands spanning 500–620 nm.

The computed spectra reproduce these features well (Figure S36, Table S8). TD‐DFT analysis indicates that both the Soret and Q bands are dominated by ligand‐centered excitations involving HOMO/HOMO‐1/−2 → LUMO/LUMO + 1/ + 2 transitions, with negligible MLCT and only minor LMCT contributions arising from weak cobalt d‐orbital mixing (Figure S38).

The axial coordination of pyridine to complexes **3** and **4** was examined by UV–vis titration, and binding constants were obtained using the Hill equation [84, 105]. Progressive Q‐band enhancement and replacement of the original Soret band by a redshifted feature indicate conversion from mono(pyridine) to bis(pyridine) adducts. Hill slopes ≈1 confirm single‐site binding, with log K = 3.51 for **3** and 3.24 for **4**, consistent with weaker pyridine affinity in the latter (Figure 2b,d, insets). Spectral shifts between CH_3_CN and pyridine support five‐coordinate mono(pyridine) species in CH_3_CN and six‐coordinate bis(pyridine) adducts in pyridine, the latter displaying redshifted Soret bands with enhanced Q bands (Figure 2; Figures S2–S4). Even trace pyridine (0.5%) induces rapid bis(pyridine) formation, accompanied by a distinct color change. Comparable behavior is observed for **4**, while the increased electrophilicity imparted by pentafluorophenyl substituents enhances pyridine binding relative to unsubstituted analogs [43]. Additionally, compound **1** exhibits strong fluorescence at 652 nm upon Soret excitation (Figure S5).

### 2.4 Electrochemical Analysis

**FIGURE 4:**
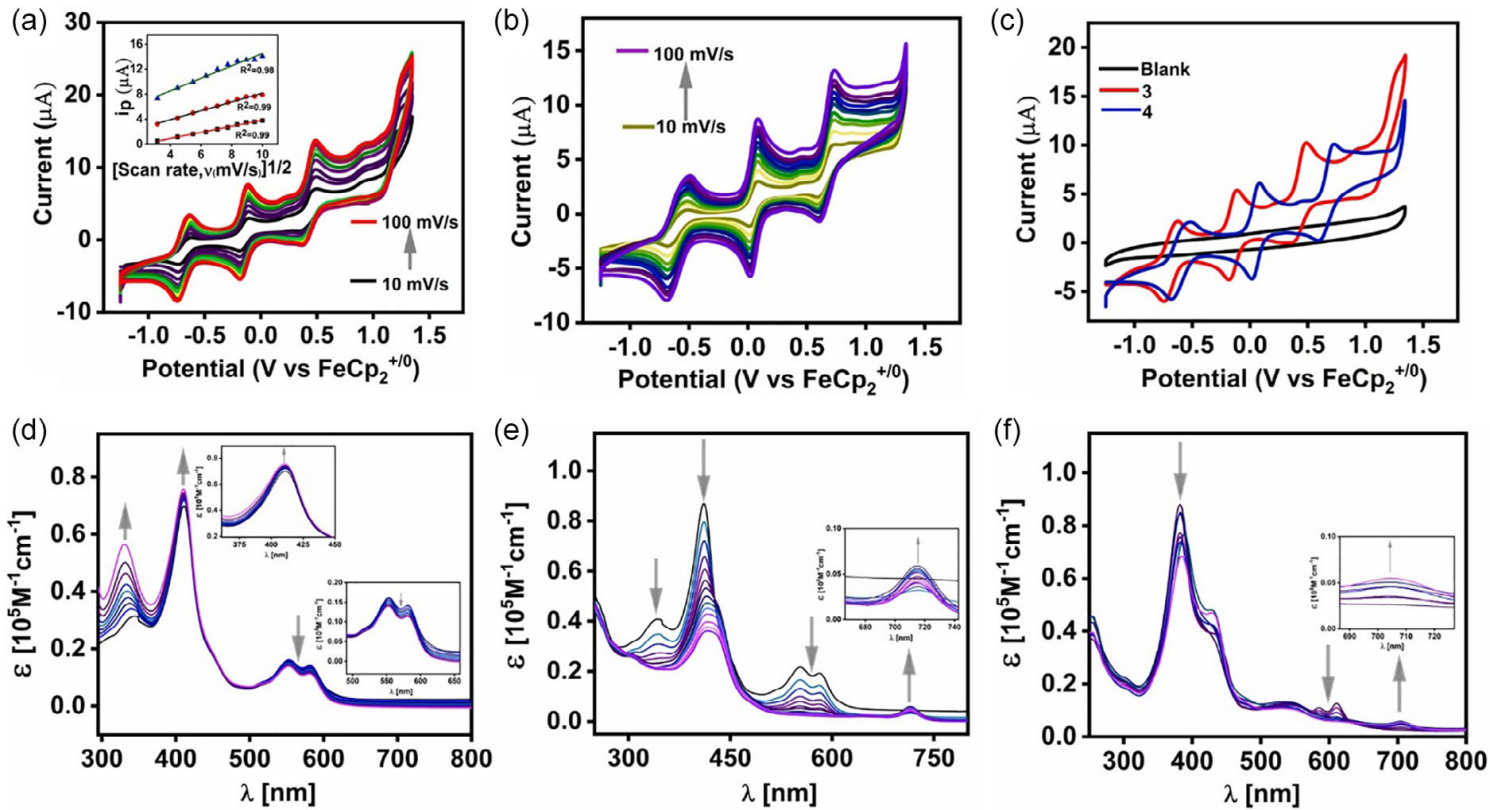
(a) Cyclic voltammogram of complex **3** and (b) complex **4** (0.5 mM) in dry CH_3_CN at scan rates of 10–100 mVs^−1^ under Ar. Inset of (a): linear dependence of peak current (I_p_) on the square root of the scan rate for complex **3**. (c) Comparative CVs of complex **3** (red), complex **4** (blue), and blank electrolyte (black). All electrochemical measurements were carried out in 0.1 M TBAPF_6_/CH_3_CN using a glassy carbon working electrode, Pt wire counter electrode, and Ag/AgCl (3M KCl) reference electrode. UV–vis absorption spectral changes of complex **3** (1 × 10^−5^ M) upon chemical oxidation and reduction using (d) CoCp_2_ (1 × 10^−3^ M) in CH_3_CN, (e) FcBF_4_ (1 × 10^−4^ M) in CH_3_CN, and (f) AgPF_6_ (1 × 10^−3^ M) in CH_2_Cl_2_.

The electrochemical behavior of complexes **3** and **4** was evaluated by cyclic voltammetry in rigorously dried CH_3_CN containing 0.1 M TBAPF_6_, using a glassy carbon working electrode, a platinum counter electrode, and an Ag/AgCl reference electrode, with potentials internally referenced to the FeCp_2_^+/0^ couple. Complex **3** exhibits a reversible Co(III/II) couple at −0.62 V, while **4** shows the corresponding process at −0.52 V (Figure 4a–c), highlighting the ability of the noninnocent corrole ligand to stabilize multiple cobalt oxidation states relevant to water oxidation catalysis [75]. To elucidate the electronic consequences of the redox events, chemical oxidation and reduction experiments were carried out in a gas‐tight cuvette, followed by UV–vis spectroscopic measurements. Solutions of complex **3** (1 × 10^‐5^ M) were prepared in dry, degassed solvent and maintained under an inert atmosphere throughout the experiments. Redox reagents were added in a stepwise manner without dilution, and spectra were recorded after each addition. Chemical reduction of complex **3** in CH_3_CN using CoCp_2_ resulted in an increase in the Soret band intensity (~410 nm), accompanied by a gradual decrease in the Q‐band intensities (Figure 4d), consistent with a one‐electron Co(III)/Co(II) redox process [84, 105]. Chemical oxidation with FcBF_4_ led to attenuation of both the Soret and Q bands, along with the emergence of a new absorption band at ~714 nm, indicative of the formation of a corrole‐centered radical species (Figure 4e) [41].

**FIGURE 5:**
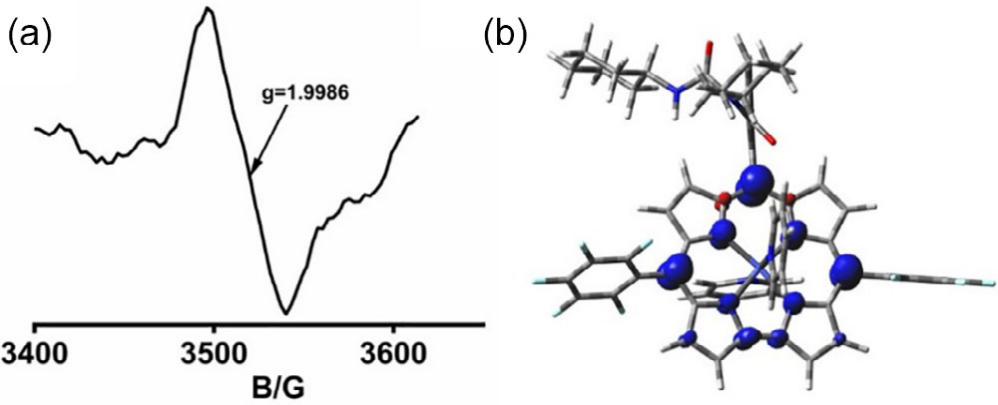
(a) X‐band EPR spectrum of [Co^III^(corrole^•2‐^)(py)_2_]^+^ generated from complex **3** (2.5 × 10^−5^ M) by controlled potential electrolysis (CPE) at + 0.01 V for 30 min in aerated CH_3_CN at RT, showing an isotropic signal at *g* = 1.9986. (b) DFT‐derived spin density plot of [Co^III^(corrole^•2‐^)(py)_2_]^+^ (iso‐value = 0.005).

**FIGURE 6:**
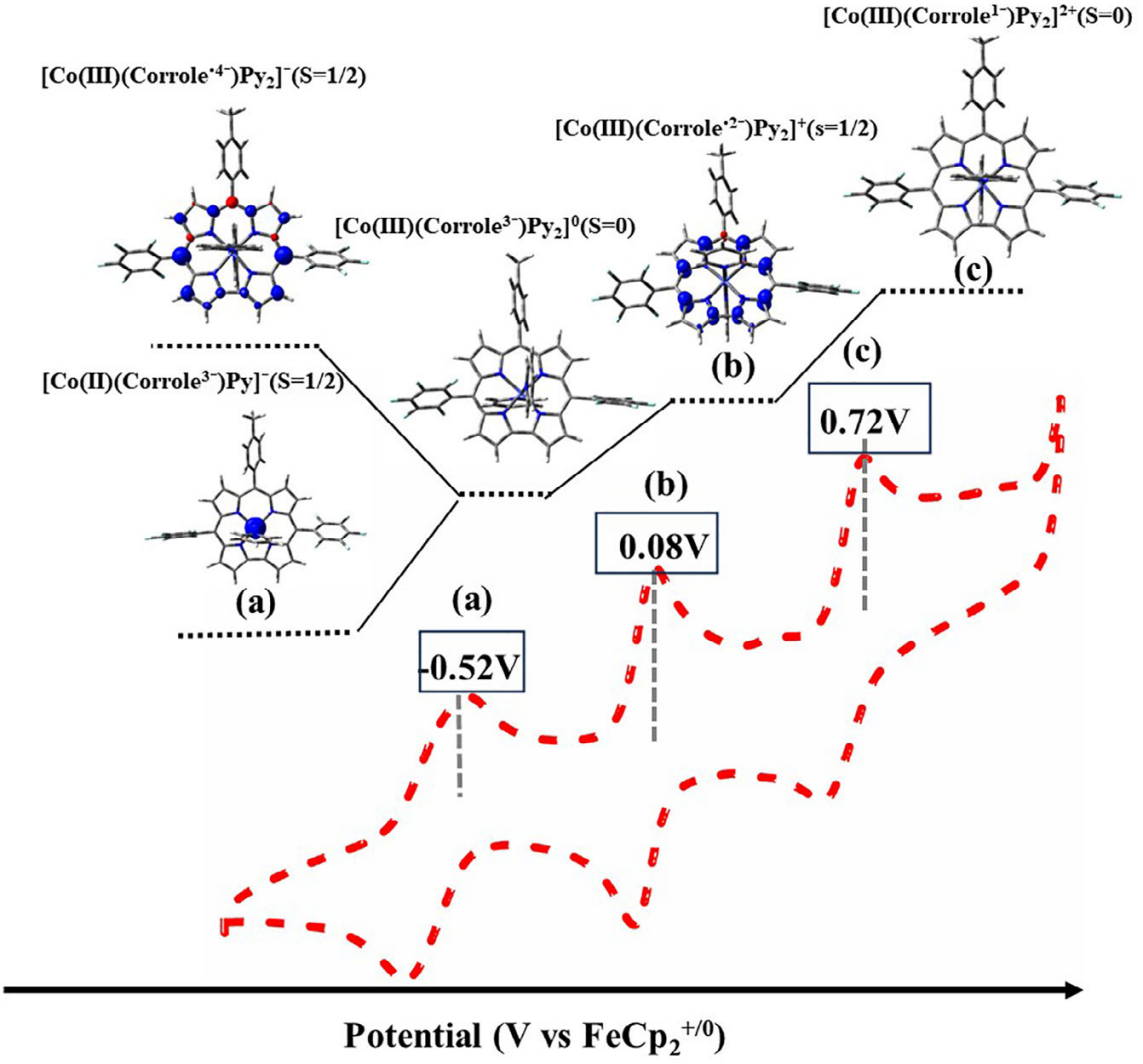
Diagram of cobalt corrole **4** with corresponding cyclic voltammetry (CV) profiles, showing the oxidation states of the cobalt center and corrole ligand as predicted by DFT, along with variations in axial pyridine ligation.

Further oxidation using AgPF_6_, in combination with controlled‐potential electrolysis, generated a new absorption feature near ~ 705 nm, consistent with stepwise oxidation localized on the corrole macrocycle (Figure 4f). EPR spectra of **3** and **4** were silent in CH_3_CN under ambient and cryogenic conditions, consistent with low‐spin Co(III) ground states [43]. Controlled potential electrolysis of **3** at + 0.01 V generated an isotropic signal at *g* = 1.9986 without hyperfine splitting, indicative of a corrole‐centered radical formulated as [Co^III^(corrole^•2‐^)(py)_2_]^+^ [106]. DFT calculations corroborated this assignment, revealing spin density predominantly localized on the macrocycle (Figure 5). Complex **4** exhibited nearly identical behavior, indicating a consistent ligand‐centered redox response across the series. Progressive oxidation of complexes **3** and **4** defines a well‐resolved redox manifold (Figure 6).

The neutral resting state is assigned as closed‐shell [Co^III^(corrole^3−^)(py)_2_]^0^ (S = 0); one‐electron reduction yields [Co^II^(corrole^3−^)(py)]^−^, while oxidation affords the ligand‐centered radical [Co^III^(corrole^•2−^)(py)_2_]^+^, (S = 1/2), as confirmed by UV–vis, CV, and EPR. Further oxidation produces a two‐electron‐oxidized species with pronounced Soret/Q‐band attenuation and EPR silence, consistent [Co^III^(corrole^1−^)(py)_2_]^2+^. Spin‐density analyses support dynamic redox noninnocence, with electron redistribution between the cobalt center and corrole framework across oxidation states from −1 to + 1 [107, 108].

### 2.5 Catalytic Oxygen Evolution via Electrochemical Oxidation

**FIGURE 7:**
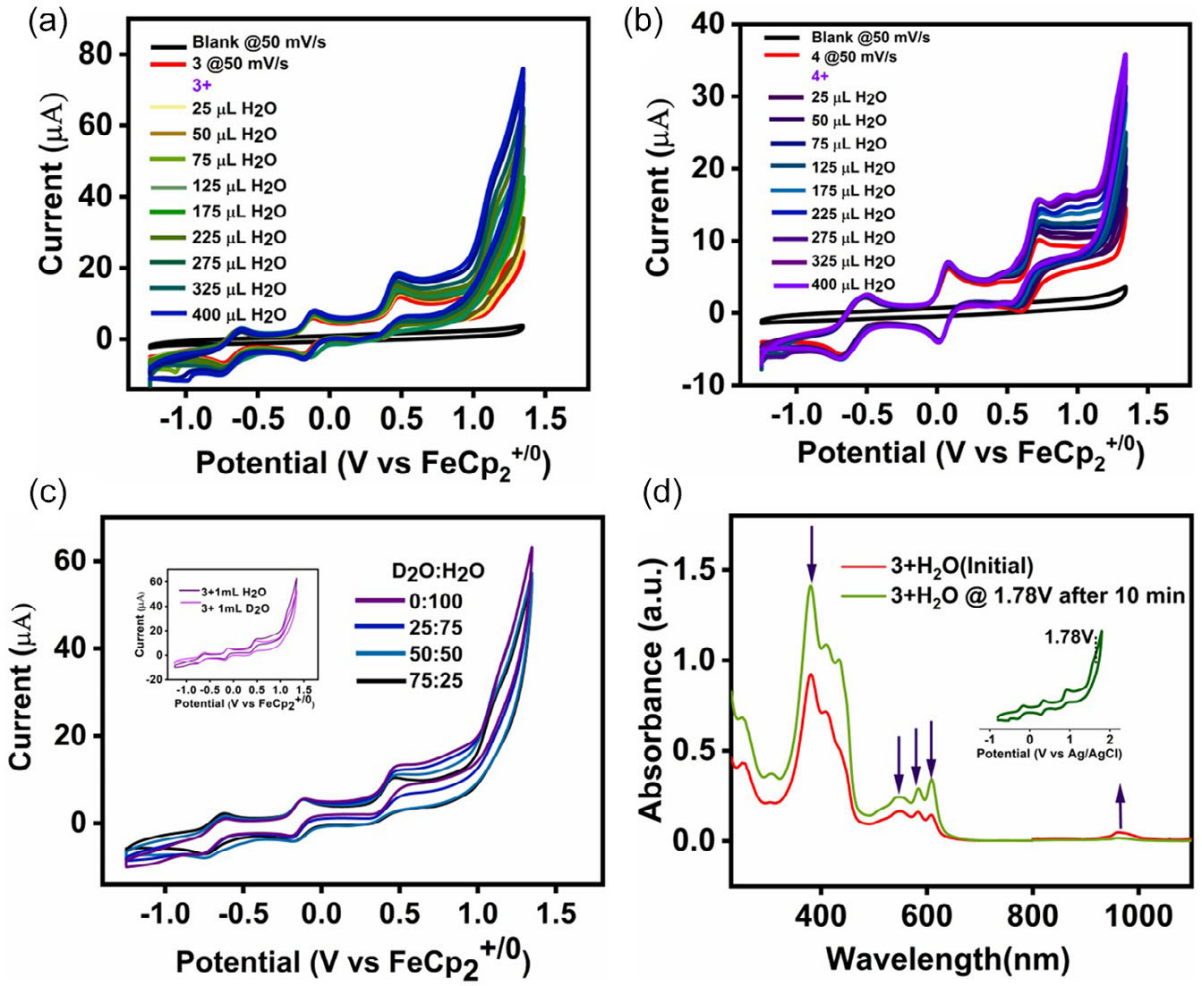
(a) CV of compound **3** and (b) compound **4** (0.5 mM) in dry CH_3_CN at 50 mV/s scan rate after the addition of a different quantity of water (25 μL to 400 μL) under Ar atmosphere. (c) CV of compound **3** (0.5 mM) in CH_3_CN: H_2_O/D_2_O (19:1) with a different volume ratio of H_2_O and D_2_O at 50 mV/s under Ar atmosphere. The data was recorded with a Glassy carbon working electrode, Pt wire counter electrode, and Ag/AgCl (in 3 M KCl) reference electrode in the presence of 0.1 M TBAPF_6_ electrolyte. Inset of 7(c) shows the overlap of CV of complex **3** in presence of 1 mL H_2_O (red trace) and 1 mL D_2_O (blue trace) to determine K_H_/K_D_ ratio. (d) Spectro‐electrochemical plot of compound **3** in the presence of water at 1.78 V versus Ag/AgCl concerning the CV.

The electrocatalytic activity of complexes **3** and **4** was evaluated by incremental addition of water (0–400 μL) to acetonitrile solutions. A well‐defined catalytic wave emerges at ~1.12 V for **3** and ~1.21 V for **4** (Figure 7; Figures S39–S41), marking the onset of O_2_ evolution. This feature is consistent with water coordination at the cobalt center following dissociation of an axial pyridine ligand, thereby generating a vacant site for substrate binding [39, 40, 44]. Isotopic substitution studies were subsequently performed by varying the H_2_O/D_2_O ratio in the electrolyte solution to probe this mechanistic proposal.

The decrease in catalytic current with increasing D_2_O content confirms the involvement of proton‐coupled electron transfer (PCET) in the catalytic process [40]. A modest kinetic isotope effect (KIE = 1.3; Figure 7c) is consistent with established mechanisms for transition‐metal‐mediated O_2_ evolution [95]. Controlled‐potential electrolysis at 1.78 V (Ag/AgCl) demonstrates sustained catalytic activity, with complex **3** achieving a Faradaic efficiency of 83.9%, which is significantly higher than that of complex **4** (59.6%) and in good agreement with values typically reported for highly efficient Co(III) corrole‐based OER catalysts (∼80%–100%). Turnover frequencies (TOF), estimated from the ratio of catalytic current (I_c_) at the third oxidation wave to the peak current (I_p_) of the first redox event, are 1.19 s^−1^ at 1.12 V for **3** and 0.47 s^−1^ at 1.21 V for **4** (see the Supporting Information). The TOF values obtained in this study are comparable with those reported for some of the most efficient molecular Co(III) corrole OER catalysts, such as the Hangman corrole (0.81 s^−1^), Co(tpfc)(Py)_2_, and Co(BAPC)(Py)_2_ (0.20 s^−1^), as well as *p*‐methylcarboxyphenyl‐ and *p*‐nitrophenyl‐substituted Co(III) corroles (1.67–1.86 s^−1^) [39, 40, 43, 58, 99, 109]. The gas‐chromatographic analysis confirms molecular O_2_ as the dominant gaseous product. Mechanistically, catalysis proceeds via a high‐valent cobalt‐oxyl intermediate [Co^III^(corrole^•2‐^)(O^•‐^)(py)] (**3c**, **4c**), identified as the catalytically competent species [39, 40, 43, 44, 98]. Nucleophilic attack of water at this electrophilic center generates the hydroperoxo intermediate [Co^III^(corrole^3−^)(OOH)(py)]^‐^ (**3d**, **4d**), which subsequently evolves through superoxo and peroxo intermediates (**3e/4e** and **3f/4f**) prior to O_2_ release (Figures S42–S43). Although the oxyl species is EPR‐silent, its formation is supported by spectroelectrochemical data and precedent in cobalt‐corrole chemistry [39, 43, 44]. Dissociation of an axial pyridine ligand provides the vacant coordination site required for water binding, consistent with the experimentally defined resting state [39, 40, 44]. Anodic oxidation generates the cobalt‐hydroxo precursor [Co^III^(corrole^•2‐^)(OH)(py)] (**3b**, **4b**), which serves as the entry point to the catalytic cycle. Subsequent oxidation generates the cobalt‐oxyl radical [Co^III^(corrole^•2‐^)(O^•‐^)(py)] (**3c, 4c**), a transient yet catalytically competent species widely implicated in corrole‐based water‐oxidation mechanisms [40, 43, 44, 75]. Under catalytic conditions, nucleophilic attack of water or hydroxide at this electrophilic oxyl center constitutes the rate‐determining step, forming the hydroperoxo intermediate [Co^III^(corrole^3−^)(OOH)(py)]^‐^ (**3d, 4d**). Spectroelectrochemical measurements support the formation of an oxygenated intermediate, as evidenced by attenuation of the Soret and Q bands and emergence of a new absorption near 950 nm (Figure 7d), consistent with Co‐OOH or related Co‐O_2_ species reported for molecular OER catalysts [41, 44]. Further deprotonation and one‐electron oxidation yield the cobalt‐superoxo [Co^III^(corrole^3−^)(O_2_^•^)(py)]^‐^(**3e, 4e**), which upon additional oxidation forms the oxygen adduct [Co^III^(corrole^3−^)(O_2_)(py)] (**3f, 4f**), the immediate precursor to O_2_ release [40, 43, 44]. This stepwise hydroperoxo‐mediated pathway contrasts with lattice‐oxygen coupling in heterogeneous cobalt oxides, yet closely aligns with molecular mechanisms in which O—O bond formation proceeds via water attack at a high‐valent metal‐oxo/oxyl center [110, 111, 112]. Collectively, these results establish the cobalt‐oxyl radical as the electrophilic trigger for O—O bond formation and highlight the critical role of hydroperoxo and superoxo intermediates in cobalt corrole‐mediated oxygen evolution.

**FIGURE 8:**
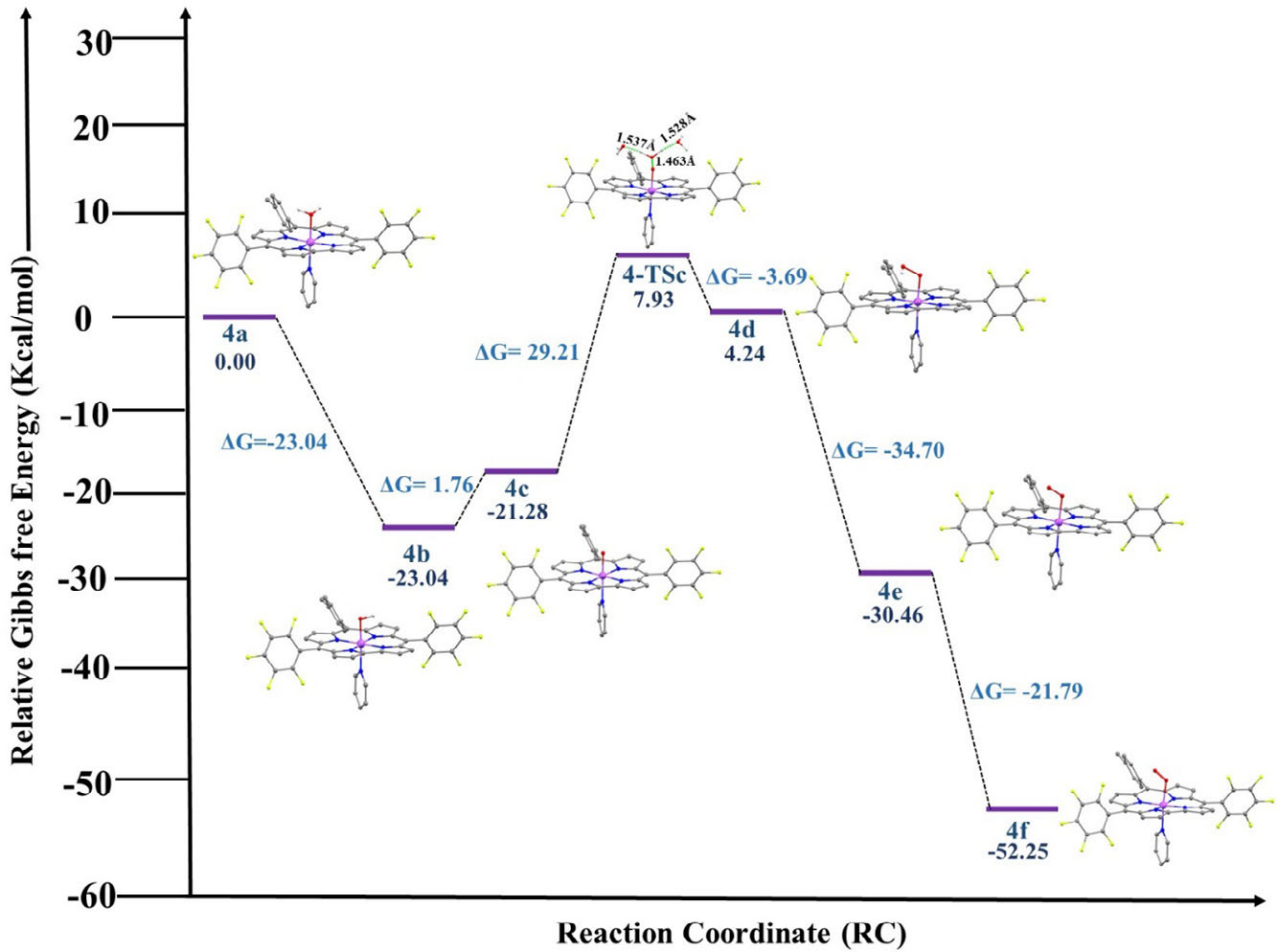
Free‐energy profile (ΔG, kcal mol^−1^) for O—O bond formation in the corrolato cobalt (III) complex [Co^III^(corrole^•2‐^)(O^•‐^)(Py)]^0^, (**4c**), proceeding through a water nucleophilic attack (WNA) transition state. The cobalt center is shown in violet, oxygen atoms in red.

DFT calculations support the proposed water‐oxidation pathways for complexes **3** and **4**. For complex **4**, the computed free‐energy profile (Figure 8) begins from the aqua‐bound Co(III) species **4a** (0.0 kcal mol^−1^), which upon sequential oxidation and deprotonation forms intermediate **4b** (−23.04 kcal mol^−1^) and the cobalt‐oxyl species **4c** (−21.28 kcal mol^−1^). O—O bond formation proceeds via an associative WNA at the electrophilic Co‐oxyl center, with an activation barrier of + 29.21 kcal mol^−1^ (r(O···O) = 1.463 Å), leading to hydroperoxide intermediate **4d** (+4.24 kcal mol^−1^) and subsequently to thermodynamically downhill superoxo species **4e** and **4f** (ΔG = −30.97 kcal mol^−1^ relative to **4c**) [113], with solvent‐assisted hydrogen bonding stabilizing the WNA transition state (Figure 8, S44). In the urea–functionalized complex **3**, oxidation and deprotonation similarly generate a cobalt‐oxyl intermediate (**3c**), which undergoes associative WNA with a higher activation barrier of +36.23 kcal mol^−1^ (Figure S45); here, intramolecular hydrogen bonding from the pendant amide carbonyl stabilizes the transition state, facilitating downhill formation of hydroperoxide and superoxo intermediates (Figures S42, S45). Consistent with this interpretation, a truncated model lacking the pendant carbonyl group exhibits an increased WNA barrier (+39.87 kcal mol^−1^), confirming the critical role of intramolecular hydrogen bonding in transition‐state stabilization, with the corresponding hydroperoxide and superoxo intermediates being significantly less stabilized (Figure S46). ESI‐MS detection of cobalt‐oxyl like species further supports the proposed intermediates (Figure S47), and collectively the calculations identify WNA at the Co‐oxyl center as the rate‐determining step, highlighting secondary‐sphere hydrogen bonding as a key design element for efficient O—O bond formation.

## 3 Conclusions

This study establishes that secondary‐sphere design is a powerful lever for advancing molecular cobalt corrole catalysts in water oxidation. By introducing a pendant benzoyl‐urea functionality into the corrole scaffold, we demonstrate that a strategically placed hydrogen‐bond donor can profoundly influence both electronic structure and catalytic performance. Structural data and DFT calculations reveal that the benzoyl‐urea functionality is oriented above the macrocyclic plane, positioned to interact directly with substrates and reactive intermediates. This spatial arrangement enables stabilization of high‐valent cobalt‐oxyl species and promotes substrate preorganization, lowering the energetic barrier for O—O bond formation via the WNA pathway. Electrochemical and spectro‐electrochemical studies, supported by EPR, provide convergent evidence that catalysis proceeds through sequential Co and corrole‐centered redox events culminating in a cobalt‐oxyl radical intermediate. The urea–substituted complex exhibits a marked enhancement in performance**,** with reduced onset potential, Faradaic efficiency approaching 84%, and a 2.5‐fold increase in turnover frequency relative to its control analog. Importantly, mechanistic probes identify a modest kinetic isotope effect, underscoring the central role of proton‐coupled electron transfer. Beyond establishing a robust cobalt corrole catalyst, these findings underscore a broader principle: secondary‐sphere hydrogen‐bonding interactions can rival or even surpass primary coordination modifications in dictating catalytic outcome**.** The benzoyl‐urea motif thus emerges as a versatile design element that integrates noncovalent functionality with redox‐active macrocycles, echoing the outer‐sphere control elements of the biological oxygen–evolving complex. Taken together, this work provides a blueprint for rational molecular design where outer‐sphere motifs are deliberately positioned to stabilize high‐energy intermediates, manage proton flux, and accelerate bond‐forming steps**.** Such strategies are expected to generalize across porphyrinoids, polypyridyls, and other ligand platforms, offering new opportunities for tailoring earth‐abundant catalysts for sustainable energy conversion**.**

## 4 Experimental Section

### 4.1 Materials

The precursor's 2‐carboxybenzaldehyde, dicyclohexylcarbodiimide, N‐methyl morpholine, pyrrole, 2,3,dichloro‐5,6‐dicyanobenzoquinone, 2,3,4,5,6‐pentafluoro‐benzaldehyde were purchased from Aldrich, USA. Cobalt‐acetate tetrahydrate and pyridine were purchased from Merck, India. Other chemicals were of reagent grade. Hexane and CH_2_Cl_2_ were distilled from KOH and CaH_2_, respectively. For spectroscopic and electrochemical studies, HPLC‐grade and anhydrous solvents were used. The FB corroles were synthesized according to the Gryko methodology [64]. The cobalt(III) corrole derivatives were prepared following previously reported procedures [65, 66, 67, 68, 69, 70].10‐(4‐methylphenyl)−5,15‐bis(pentafluorophenyl)corrole (**2**) was synthesized by following an earlier reported procedure [102].

### 4.2 Physical Measurements

UV–vis spectral studies were performed on a Perkin‐Elmer LAMBDA‐750 spectrophotometer. The elemental analyses were carried out with a Euro EA elemental analyzer. Emission spectra were performed on an Edinburgh FLS920 spectrofluorometer equipped with a PMT980 for the visible and with a Ge‐detector for emission in the NIR spectral region, using an optical cell of 1 cm path length compared. FT‐IR spectra were recorded on a Perkin‐Elmer spectrophotometer with samples prepared as KBr pellets. The NMR measurements were carried out using a Bruker AVANCE 400 NMR and 700 NMR spectrometer. Tetramethylsilane (TMS) is the internal standard. Electrospray mass spectra were recorded on a Bruker Micro TOF‐QII mass spectrometer. Cyclic voltammetry measurements were carried out using a CS350 electrochemical test system (China). EPR spectra was recorded with a Bruker EMX System (ER 073) (X‐band frequency of ca. 9.5 GHz). Synthetic quartz glass tubes were used for the measurements. EPR measurement was performed at a temperature of 298 K. The gas‐chromatography data was recorded in Shimadzu GC‐2010 porapak capillary gas chromatograph, with TCD detector. A glassy carbon working electrode, a platinum wire as an auxiliary electrode, and an Ag–AgCl reference electrode were used in a three‐electrode configuration. Tetrabutylammonium Hexafluorophosphate (TBAPF_6_) was the supporting electrolyte (0.1 M), and the concentration of the solution was 10^−3^M for the complex. The half‐wave potential E^0^_298_ was set equal to 0.5 (E_pa_ + E_pc_), where E_pa_ and E_pc_ are anodic and cathodic cyclic voltammetric peak potentials, respectively. The scan rate used was 50 mV s^−1^.

### 4.3 Crystal Structure Determination

Single crystals of **1** were grown by slow diffusion of solution of **1** in CH_2_Cl_2_ into hexane, followed by slow evaporation under atmospheric conditions. Single crystals of **3** were grown by slow diffusion of solution of **3** in CH_2_Cl_2_ into hexane, followed by slow evaporation under atmospheric conditions. Single crystals of compound **4** were obtained by layering a CH_2_Cl_2_ solution of the compound with hexane, allowing slow diffusion to occur. The crystal data of **1, 3,** and **4** were collected on a Rigaku Oxford diffractometer at 100 K. Selected data collection parameters and other crystallographic results are summarized in Table S2. All data were corrected for Lorentz polarization and absorption effects. The program package SHELXTL [114] was used for structure solution and full‐matrix. Hydrogen atoms were included in the refinement using the riding model. Contributions of H atoms for the water molecules were included but were not fixed. Disordered solvent molecules were taken out using the SQUEEZE command in PLATON [115]. CCDC 2494375‐2494377 contains the supplementary crystallographic data for **1**, **3**, and **4**. These data can be obtained free of charge via https://www.ccdc.cam.ac.uk/data_request/cif

### 4.4 Computational Methods

All quantum chemical calculations were carried out using the Gaussian 16 suite of programs [116]. Geometry optimizations and vibrational frequency analyses for compounds **1**, **3**, and **4** were performed using density functional theory (DFT) at the B3LYP level of theory in conjunction with a mixed basis set approach (GENECP). For main group elements (H, C, N, O, and F), the 6‐311G(d, p) basis set was employed, while the cobalt center was described using the LANL2DZ effective core potential (ECP) basis set. The optimized geometries exhibited estimated bond length deviations of approximately ±0.01 Å. To account for solvent effects, the polarizable continuum model (PCM) was applied, using acetonitrile as the solvent environment for compounds **3** and **4**. Time‐dependent DFT (TD‐DFT) calculations were conducted on compounds **1**, **3**, and **4** using the same functional and basis set combination to probe their electronic excitation properties. Transition state (TS) searches were carried out at the same theoretical level using the Berny algorithm, with full frequency calculations performed to confirm the nature of the stationary points. Solvation for TS optimizations was modeled using the integral equation formalism PCM (IEFPCM) with acetonitrile as the continuum solvent.

### 4.5 Bulk Electrolysis

Bulk electrolysis (BE) and chronocoulometric experiments were conducted in an air‐tight 60 mL glass vessel. Three of the necks were fitted with electrodes: a 0.5 cm × 0.5 cm glassy carbon working electrode, a platinum wire as the counter electrode, and an Ag/AgCl (in 3 M KCl) electrode as the reference. The sample solution purged with Ar before starting the electrolysis. The vessel was charged with 40 mL of a 0.5 mM solution of the complex in a 19:1 (v/v) mixture of CH_3_CN: H_2_O, containing 0.1 M (TBAPF_6_) as the supporting electrolyte. All electrodes, were inserted with septum in a gas‐tight manner. The solution was purged with argon gas for 30 min to remove dissolved oxygen. After purging, the chronocoulometric experiment was initiated at an applied potential of 1.78 V versus Ag/AgCl. The evolution of oxygen was measured using gas chromatography. A blank bulk electrolysis experiment was performed under identical conditions in the absence of the catalyst to confirm the catalytic nature of the observed reaction. All experiments were carried out at room temperature.

### 4.6 Synthesis

#### 4.6.1 Synthesis of N‐Cyclohexyl‐N‐(cyclohexylcarbamoyl)‐2‐Formylbenzamide

2‐carboxybenzaldehyde (530 mg, 3.53 mmol) was taken into a 250 mL round‐bottom flask (RB), after which 150 mL DCM were added to it, and set to stir in an ice bath. After 5 min, N‐methyl morpholine (1.2 mL) was added to the previous mixture, along with dicyclohexylcarbodiimide (1.136 g, 5.9 mmol). The flask was removed from the ice bath and left to stir at room temperature overnight. The reaction mixture was filtered through a filter paper, after which phase separation was carried out using a water–DCM mixture. The organic layer was passed through anhydrous sodium sulfate, and the solvent was removed under reduced pressure using a rotary evaporator to afford the white solid compound.

Yield: 11% (133 mg); ^1^H NMR (400 MHz, Chloroform‐*d*) δ 10.06 (s, 1H, Ald‐H), 7.91–7.82 (m, 1H, Ar‐H), 7.70–7.55 (d, *J* = 8.5 Hz, 2H, Ar‐H), 7.46–7.35 (dd, *J* = 6.8 Hz, 1H, Ar‐H), 6.88–6.84 (bs, 1H, N‐H), 4.25–4.03 (m, *J* = 1H, ε‐cy‐H), 3.48–3.36(m, 1H, ε‐cy‐H), 1.90–1.15 (m, 20H, cy‐H). ^13^C NMR (101 MHz, Chloroform‐*d*) δ 193.24, 153.41,146.22, 137.84, 134.30, 132.32, 129.59, 126.78, 125.71,122.48, 49.45, 33.94,32.10, 31.99, 30.58, 25.25, 24.51. HRMS (ESI) *m/z*: [**M**+Na]^+^ Calcd for C_21_H_28_N_2_O_3_Na 379.1998; Found 379.1985 (Figure S9).

#### 4.6.2 Synthesis of 10‐(2‐{N‐Cyclohexyl‐N‐(Cyclohexylcarbamoyl)benzamido})−5,15‐Bis(pentafluorophenyl)corrole, 1

The FB corrole was prepared following a slightly modified Gryko method [64]. In a 500 mL round‐bottom flask (RB), N‐cyclohexyl‐N‐(cyclohexylcarbamoyl)‐2‐formylbenzamide (0.356 g, 1 mmol) and (pentafluorophenyl)dipyrromethane (0.624 g, 2 mmol) were dissolved in methanol (100 mL) and stirred at room temperature. Separately, an HCl solution was prepared by dissolving concentrated HCl (12 M, 10.5 mL) in distilled water (100 mL). This acidic solution was added to the methanolic reaction mixture, which was then stirred at room temperature for 2 hr. During the course of the reaction, the solution gradually turned cloudy and developed a pink–brown color. The reaction mixture was transferred to a 1 L separatory funnel and extracted with chloroform (3 times). The combined organic layers were washed with water (2 times), dried over anhydrous sodium sulfate, and diluted with chloroform to a final volume of 300 mL. To this solution, DDQ (0.680 g, 3 mmol) was added, and the mixture was stirred at room temperature for 1 hr. The crude reaction mixture was then concentrated to ≈20 mL and loaded onto a silica gel column prepacked with a 1:1 CH_2_Cl_2_/hexane mixture. The fluorescent macrocyclic product (**1**), visible as a distinct band at the top of the column, was eluted using 100% CH_2_Cl_2_ to afford a purple solution. Removal of the solvent under reduced pressure yielded compound **1** as a shiny purple solid.

Yield: 10% (65 mg, 0.068mmol); UV/Vis (CH_2_Cl_2_): *λ*_max_/nm (*ε*/M^−1^ cm^−1^) in CH_2_Cl_2_: 410(83786), 563(13701), 611(8582), 638(4338) (Figure S1). ^1^H NMR (700 MHz, CDCl_3_) δ 9.11 (s, 2H, Py‐H), 8.68–8.65 (m, 4H, Py‐H), 8.56 (s, 2H, Py‐H), 8.16 (s, 1H, Ar‐H), 7.84−7.82 (m, 3H, Ar‐H), 6.14 (bs, 1H, N‐H), 3.66−3.63 (m, 1H, ε‐cy‐H), 3.00 (m, 1H, ε‐cy‐H), 1.48−1.26 (m, 20H, cy‐H). ^13^C NMR (176 MHz, CDCl_3_) δ 172.45, 153.13, 146.77, 145.37, 142.46, 140.58, 138.64, 137.92, 137.20, 135.37, 128.50, 125.75, 117.52, 113.88, 109.50, 59.16, 53.39, 49.08, 41.31, 34.63, 32.02, 26.05, 24.37, 22.58, 20.66. ^19^F NMR (377 MHz, Chloroform‐*d*) δ: −137.83(t, *J* = 24.3 Hz), −152.55 (t, *J* = 20.4 Hz), −161.63 (t, *J* = 21.7 Hz). HRMS (ESI) *m/z*: [**1**+H]^+^ Calcd for C_51_H_39_N_6_O_2_F_10_ 957.2975; Found 957.2981 (Figure S10).

#### 4.6.3 10‐(2‐{N‐Cyclohexyl‐N‐(Cyclohexylcarbamoyl)benzamido})−5,15‐Corrolato Cobalt(III) bis(pyridine) Complex, 3

The incorporation of cobalt(III) into the FB corrole framework (compound **1**) was accomplished via a modified metalation protocol [65, 66, 67, 68, 69, 70]. A solution of the FB corrole (50 mg, 0.052 mmol) in 10 mL of pyridine was prepared under ambient conditions. To this solution, cobalt(II) acetate tetrahydrate [Co(OAc)_2_·4H_2_O], (130 mg, 0.52 mmol) was added as the cobalt source. The reaction mixture was then heated at 100°C in an oil bath with continuous stirring until the characteristic fluorescence of the starting material had completely disappeared, indicating successful metal insertion. Following completion of the reaction, the solvent was removed under reduced pressure using a rotary evaporator. The resulting crude residue was dissolved in a minimal volume of dichloromethane and purified by column chromatography over silica gel, using a 1:1 mixture of *n*‐hexane and dichloromethane as the mobile phase. The desired product appeared as a distinct brown band, which was collected and further purified by recrystallization from a 3:1 *n*‐hexane and dichloromethane mixture with the addition of a few drops of pyridine to aid its axial binding.

Yield: 75% (46 mg, 0.039 mmol); UV/Vis [CH_3_CN, λ_max_, nm (*ε*, M^−1^cm^−1^)]: 379(44527), 434(21237),540(7190), 584(6358), 611(7368); UV/Vis [Pyridine, λ_max_, nm (*ε*, M^−1^cm^−1^)]: 440(63642), 451(sh)(52114), 510(4080), 542(4951), 562(6853), 589(17093), 613(25848) (Figure 2, Figure S2 and Figure S4,); ^1^H NMR (400 MHz, Benzene‐*d*_6_) δ 9.30–9.28 (t, *J* = 4.3 Hz, 2H, Py‐H), 9.02−8.88 (m, 3H, Py‐H), 8.85−8.68 (m, 2H, Py‐H), 8.08 (dd, *J* = 7.0 Hz, 1H, Py‐H), 7.87−7.17 (m, 4H, Ar‐H), 5.72 (bs, 1H, N‐H), 4.76 (brs, 2H, Pyr‐H), 4.27 (brs, 4H, Pyr‐H), 3.90 (t, *J* = 13.3 Hz, 1H, ε‐cy‐H), 3.10 (d, 1H, ε‐cy‐H), 1.37 (brs, *J* = 9.3 Hz, 4H, Pyr‐H), 1.34−0.75 (m, 20H, cy‐H); ^13^C NMR (176 MHz, Benzene‐*d*_6_) δ 175.12, 168.24, 153.19, 147.39, 145.97, 142.23, 141.82, 140.94, 139.32, 139.19, 138.73, 137.30, 135.34, 134.57, 131.34, 131.17, 129.66, 129.37, 129.05, 125.71, 125.26, 121.27, 119.70, 117.24, 114.53, 110.08, 108.39, 95.06, 94.85, 60.44, 49.10, 48.05, 32.54, 32.13, 30.23, 26.69, 25.19, 24.62, 23.15. ^19^F NMR (377 MHz, Benzene‐*d*_6_) δ (−138.83)−(−139.30) (m), −154.22 (dt, *J* = 37.1, 21.6 Hz), (−162.94)−(−163.19) (m); ESI‐MS: *m/z* calcd for C_51_H_35_CoF_10_N_6_O_2_ [**3**‐2Py]^**+**^: 1012.1994; found: 1012.1830. (Figure S11).

#### 4.6.4 Synthesis of 10‐(4‐Methylphenyl)‐5,15‐Bis(pentafluorophenyl) Corrolato Cobalt(III) Bis(pyridine) Complex, 4

A solution of FB corrole, **2** (50 mg, 0.069 mmol) in pyridine was prepared in a round‐bottom flask under ambient conditions, to which cobalt(II) acetate tetrahydrate [Co(OAc)_2_·4H_2_O] (173 mg, 0.69 mmol) was added. The reaction mixture was heated at 100°C in an oil bath with constant stirring until the characteristic fluorescence of the FB corrole had completely disappeared. The reaction mixture was then cooled to room temperature, and the solvent was removed under reduced pressure. The resulting crude residue was dissolved in a minimal amount of dichloromethane and purified by column chromatography over silica gel using n‐hexane/dichloromethane (1:1, v/v) as the eluent. The desired cobalt(III) corrole complex was isolated as a distinct brown band and further purified by recrystallization from a 3:1 (v/v) mixture of *n*‐hexane and dichloromethane with the addition of a few drops of pyridine to stabilize axial coordination.

Yield: 75% (48 mg, 0.05mmol); UV/Vis [CH_3_CN, λ_max_, nm (*ε*, M^−1^cm^−1^)]: 378(43893), 428(23271),546(6745), 583(6361), 612(7606); UV/Vis [Pyridine, λ_max_, nm (*ε*, M^−1^cm^−1^)]: 435(71952), 448(sh)(57914), 507(4278), 539(5432), 563(8194), 584(17159), 611(31352) (Figure 2, Figure S3 and Figure S4).; ^1^H NMR (700 MHz, Chloroform‐*d*) δ 9.20−9.17 (m, 2H, Py‐H), 8.90 (d, *J* = 3.1 Hz, 4H, Py‐H), 8.75 (d, *J* = 3.8 Hz, 2H, Py‐H), 7.93 (d, *J* = 7.6 Hz, 2H, Ar‐H), 7.47 (d, *J* = 7.5 Hz, 2H, Ar‐H), 6.36 (brs, 4H, Pyr‐H), 5.60 (brs, 6H, Pyr‐H), 2.64 (s, 3H, Methyl‐H); ^13^C NMR (101 MHz, Chloroform‐*d*) δ 141.74, 141.29, 139.70, 138.87, 136.30, 134.71, 134.41, 130.60, 129.37, 128.25, 127.52, 124.12, 118.81, 21.44. ^19^F NMR (377 MHz, Chloroform‐*d*) δ−138.26 (dd, *J* = 25.2, 8.4 Hz), −154.84 (t, *J* = 20.9 Hz), −163.00 (ddd, *J* = 25.2, 21.0, 8.4 Hz).; ESI‐MS: *m/z* calcd for C_38_H_15_CoF_10_N_4_ [**4**‐2Py]^**+**^: 776.0469; found: 776.0433. (Figure S12).

## Supporting Information

Additional supporting information can be found online in the Supporting Information section.

## Funding

This work was supported by the Department of Atomic Energy, Government of India; Board of Research in Nuclear Sciences.

## Accession Codes

CCDC 2494375−2494377 contains the supplementary crystallographic data for this paper. These data can be obtained free of charge via https://www.ccdc.cam.ac.uk/data_request/cif, or by mailing data_request@ccdc.cam.ac.uk, or by contacting The Cambridge Crystallographic Data Centre, 12 Union Road, Cambridge CB21EZ, UK; fax: +44 1223336033.

## Conflicts of Interest

The authors declare no conflicts of interest.

## Supporting information

Supplementary Material

## Data Availability

The data that supports the findings of this study are available in the supplementary material of this article.
