# Supplementary material for: Harnessing Benzoyl‐Urea Secondary‐Sphere Hydrogen‐Bonding to Enhance Oxygen Evolution Catalysis by Cobalt Corroles
Source: Small Sci. 2026 Apr 25;6(4):e70283. doi: 10.1002/smsc.70283 (PMC13116115; doi:10.1002/smsc.70283)
Supplement: Supplementary file 1 — Supplementary Material [file SMSC-6-e70283-s001.pdf]

# Supporting Information

## Harnessing Benzoyl-Urea Secondary-Sphere Hydrogen-Bonding to Enhance Oxygen Evolution Catalysis by Cobalt Corroles

Rwiddhi Chakraborty, Sahanwaj Khan, Subhajit Kar, Tanmoy Pain, Biswajit Das, Pravansu panda, Narayan Ch. Jana, and Sanjib Kar*

[†]*School of Chemical Sciences, National Institute of Science Education and Research (NISER), Bhubaneswar – 752050, India, and Homi Bhabha National Institute, Training School Complex, Anushakti Nagar, Mumbai, 400094, India. E-mail:* sanjib@niser.ac.in

**Table S1** UV-Vis. data[a,b,c] for **1, 3 and 4.**

**Table S2** Crystallographic Data for **1, 3 and 4.**

**Table S3** Contributions of various interactions in percentage to hirshfeld surface area in **1.**

**Table S4** Contributions of various interactions in percentage to hirshfeld surface area in **3.**

**Table S5** Contributions of various interactions in percentage to hirshfeld surface area in **4.**

**Table S6** TD-DFT Calculated electronic transitions for **1.**

**Table S7** TD-DFT Calculated electronic transitions for **3.**

**Table S8** TD-DFT Calculated electronic transitions for **4.**

**Figure S1** Electronic absorption spectrum of **1** in dichloromethane. Inset Free Base Corrole, **1** in dichloromethane overlaid with calculated vertical excitation energies (blue lines) obtained from TD-DFT (B3LYP/6-311G(d,p) for atoms; solvent = $CH_2Cl_2$, PCM model).

**Figure S2** Electronic absorption spectrum of **3** in Acetonitrile. Inset Corrolato cobalt (III) complex, **3** in $CH_3CN$ overlaid with calculated vertical excitation energies (blue lines) obtained from TD-DFT (B3LYP/6-311G(d,p) for light atoms and LANL2DZ for Co; solvent $CH_3CN$, PCM model).

**Figure S3** Electronic absorption spectrum of **4** in Acetonitrile. Inset Corrolato cobalt (III) complex, **4** in $CH_3CN$ overlaid with calculated vertical excitation energies (blue lines) obtained from TD-DFT (B3LYP/6-311G(d,p) for light atoms and LANL2DZ for Co; solvent $CH_3CN$, PCM model).

**Figure S4** Overlap of UV-spectrum of compound **3** and **4** in acetonitrile and pyridine.

**Figure S5** Electronic emission spectrum (excited at 410 nm) of **1** in dichloromethane.

**Figure S6** FT-IR spectrum of **1** as a KBr pellet.

**Figure S7** FT-IR spectrum of **3** as a KBr pellet.

**Figure S8** FT-IR spectrum of **4** as a KBr pellet.

**Figure S9** ESI- MS spectrum of N-cyclohexyl-N-(cyclohexylcarbamoyl)-2-formylbenzamide in $CH_3CN$ shows the measured spectrum with isotopic distribution pattern.

**Figure S10** ESI- MS spectrum of **1** in $CH_3CN$ shows the measured spectrum with isotopic distribution pattern.

**Figure S11** ESI- MS spectrum of **3** in $CH_3CN$ shows the measured spectrum with isotopic distribution pattern.

**Figure S12** ESI- MS spectrum of **4** in $CH_3CN$ shows the measured spectrum with isotopic distribution pattern.

**Figure S13** $^1H$ NMR (400 MHz) spectrum of N-cyclohexyl-N-(cyclohexylcarbamoyl)-2-formylbenzamide in $CDCl_3$.

**Figure S14** $^{13}C$ NMR (101 MHz) spectrum of N-cyclohexyl-N-(cyclohexylcarbamoyl)-2-formylbenzamide in $CDCl_3$.

**Figure S15** $^1H$ NMR (700 MHz) spectrum of **1** in $CDCl_3$.

**Figure S16** $^{13}C$ NMR (176 MHz) spectrum of **1** in $CDCl_3$.

**Figure S17** $^{19}F$ NMR (377 MHz) spectrum of **1** in $CDCl_3$.

**Figure S18** $^1H$ NMR (400 MHz) spectrum of **3** in $C_6D_6$.

**Figure S19** $^{13}C$ NMR (176 MHz) spectrum of **3** in $C_6D_6$.

**Figure S20** $^{19}F$ NMR (377 MHz) spectrum of **3** in $C_6D_6$..

**Figure S21** $^1H$ NMR (700 MHz) spectrum of **4** in $CDCl_3$.

**Figure S22** $^{13}C$ NMR (101 MHz) spectrum of **4** in $CDCl_3$.

**Figure S23** $^{19}F$ NMR (377 MHz) spectrum of **4** in $CDCl_3$.

**Figure S24** Single-crystal X-ray structure of compound **4**. Hydrogen atoms are omitted for clarity. Thermal ellipsoids are drawn at the 50% probability level.

**Figure S25** (a) Hirshfeld surface, and (b) Hydrogen bonding interactions in **1**.

**Figure S26** (a) Hirshfeld surface, and (b) Hydrogen bonding interactions in **3**.

**Figure S27** (a) Hirshfeld surface, and (b) Hydrogen bonding interactions in **4**.

**Figure S28** Fingerprint plots of **1** with different interactions are highlighted in colour.

**Figure S29** Fingerprint plots of **3** with different interactions are highlighted in colour.

**Figure S30** Fingerprint plots of **4** with different interactions are highlighted in colour.

**Figure S31** (a) DFT optimized structure, and (b) Potential mapping of **1.**

**Figure S32** (a) DFT optimized structure, and (b) Potential mapping of **3.**

**Figure S33** (a) DFT optimized structure, and (b) Potential mapping of **4.**

**Figure S34** TD-DFT-based electronic absorption spectra of **1**.

**Figure S35** TD-DFT-based electronic absorption spectra of **3**.

**Figure S36** TD-DFT-based electronic absorption spectra of **4**.

**Figure S37** Composition and Energies of Selected Kohn-Sham orbital energy level diagram and Molecular Orbitals of FB corrole, **1** (iso-value of 0.02).

**Figure S38** Selected Kohn-Sham orbital energy level diagram of corrolato coblat(III) complex, **4** (iso-value of 0.02).

**Figure S39** (a) Comparative CV of compound **3** (light green trace) and with 400 μL of water (deep green trace), compound **4** (orange trace) with 400 μL of water (red trace). (b) UV-Vis spectrum of compound **3** with different water concentrations.

**Experimental Protocol for Rinse Test**

**Figure S40** Rinse test for the compound **3** in dry MeCN. Conditions: 0.5 mM complex in a 0.1 M $TBAPF_6$ solution in MeCN, 50 mV/s scan rate. The data was recorded with a Glassy carbon working electrode, a Pt wire counter electrode, and Ag/AgCl (in 3M KCl) reference electrode in the presence of 0.1 M $TBAPF_6$

**Faradaic efficiency calculation**

**TOF($K_{obs}$) Calculation**

**Figure S41** Bulk electrolysis (or chronocoulometry) experiment for compound **3** (red line) and compound **4** (blue line) with blank (black line) was performed by holding the constant potential 1.78V (vs. Ag/AgCl couple) in an Ar atmosphere. The data were recorded with a 0.5 cm × 0.5 cm carbon working electrode, Pt wire counter electrode, and Ag/AgCl (3M KCl) reference electrode in the presence of 0.1 M $TBAPF_6$ electrolyte.

**Figure S42** Proposed catalytic cycle for water oxidation via the water nucleophilic attack (WNA) pathway in Co (III) corroles bearing a single apical pyridine (Py) ligand. The mono-pyridine species is generated under catalytic conditions by ligand exchange from the bis-pyridine Co (III) precursor. In this pathway, the R group denotes N-benzoyl-DCU (benzoyl-urea) derivative for **3a-3f** and 4-methylphenyl derivative for **4a-4f**.

**Figure S43** (a) O-O bond formation step *via* water nucleophilic attack (WNA) on the doubly oxidized cobalt corrole complex. Spin density plots (iso-value = 0.005) of $[Co^{III}(corrole^{\bullet 2-})(O^{\bullet -})\ Py]^0$ for (b) intermediate **4c** and (c) intermediate **3c**.

**Figure S44** Free-energy profile for the WNA step showing transition-state stabilization arising from hydrogen-bonding interactions between the incoming water nucleophile and two additional water molecules.

**Detailed theoretical calculations for cobalt complex 4 used to construct the energy profile diagram.**

**Detailed theoretical calculations for cobalt complex 3 used to construct the energy profile diagram.**

**Figure S45** Free-energy profile (ΔG, kcal mol$^{-1}$) for O-O bond formation in the corrolato cobalt (III) complex [Co$^{III}$(corrole$^{•2-}$)(O$^{•-}$)(Py)]$^{0}$, (**3c**), proceeding through a water nucleophilic attack (WNA) transition state. The cobalt center is shown in violet, oxygen atoms in red, and selected atoms of the secondary coordination sphere are highlighted.

**Detailed theoretical calculations for Truncated version of cobalt complex 3 used to construct the energy profile diagram**

**Figure S46** Free-energy profile (ΔG, kcal mol$^{-1}$) for O-O bond formation in the corrolato cobalt (III) complex [Co$^{III}$(corrole$^{•2-}$)(O$^{•-}$)(Py)]$^{0}$, (**3c-T**), proceeding through a water nucleophilic attack (WNA) transition state. The cobalt center is shown in violet, oxygen atoms in red, and selected atoms of the secondary coordination sphere are highlighted.

**Figure S47** ESI-MS data for water Oxidation intermediate for complex **3**.

Appendix 1 **Optimized Cartesian Coordinates of 1.**
Appendix 2 **Optimized Cartesian Coordinates of 3.**
Appendix 3 **Optimized Cartesian Coordinates of 4.**
Appendix 4 **Optimized Cartesian Coordinates for [Co$^{III}$ (Corrole$^{•2-}$) Py$_2$]$^{+}$ (S=1/2) of 3.**
Appendix 5 **Optimized Cartesian Coordinates for [Co$^{III}$ (Corrole$^{•2-}$) Py$_2$]$^{+}$ (S=1/2) of 4.**
Appendix 6 **Optimized Cartesian Coordinates for [Co$^{III}$ (Corrole$^{1-}$) Py$_2$]$^{2+}$ (S=0) of 4.**
Appendix 7 **Optimized Cartesian Coordinates for [Co$^{III}$ (Corrole$^{•4-}$) Py$_2$]$^{-}$ (S=1/2) of 4.**
Appendix 8 **Optimized Cartesian Coordinates for [Co$^{II}$ (Corrole$^{3-}$) Py]$^{-}$ (S=1/2) of 4.**
Appendix 9 **Optimized Cartesian Coordinates of 3a.**
Appendix 10 **Optimized Cartesian Coordinates of 3b.**
Appendix 11 **Optimized Cartesian Coordinates of 3c.**
Appendix 12 **Optimized Cartesian Coordinates of 3d.**
Appendix 13 **Optimized Cartesian Coordinates of 3e.**
Appendix 14 **Optimized Cartesian Coordinates of 3f.**

**Appendix 15 Optimized Cartesian Coordinates of 4a.**

**Appendix 16 Optimized Cartesian Coordinates of 4b.**

**Appendix 17 Optimized Cartesian Coordinates of 4c.**

**Appendix 18 Optimized Cartesian Coordinates of 4d.**

**Appendix 19 Optimized Cartesian Coordinates of 4e.**

**Appendix 20 Optimized Cartesian Coordinates of 4f.**

**Appendix 21 Optimized Cartesian Coordinates of 3c-T.**

**Appendix 22 Optimized Cartesian Coordinates of 3d-T.**

**Appendix 23 Optimized Cartesian Coordinates of 3e-T.**

**Appendix 24 Optimized Cartesian Coordinates of 3f-T.**

**Appendix 25 Optimized Cartesian Coordinates of TS(3-TS).**

**Appendix 26 Optimized Cartesian Coordinates of TS(4-TS$_{C}$).**

**Appendix 27 Optimized Cartesian Coordinates of TS(3T-TS).**

**Appendix 28 Optimized Cartesian Coordinates of TS(4-TSa).**

**Appendix 29 Optimized Cartesian Coordinates of TS(4-TSb).**

**Computational Details for Gibbs Free energy calculation**

**Table S1** UV–Vis. data for **1,3** and **4.**

| Compound | UV–vis. Data [a,b] $\lambda_{max}$ / nm ($\varepsilon$ / $10^5 M^{-1}cm^{-1}$) | Electrochemical data [b] |
|---|---|---|
| | | Oxidation $E^0$,V ($\Delta E_p$,V) Vs (Fc/$Fc^+$) |
| **1**[a] | 410(83786), 563(13701), 611(8582), 638(4338) | - |
| **3**[b] | 379(44527), 434(21237), 540(7190), 584(6358), 611(7368) | -0.68 (80) , -0.15(80), +0.41(80) |
| **4**[b] | 378(43893), 428(23271), 546(6745), 583(6361), 612(7606) | -0.59 (80) , +0.11(80) , +0.66(80) |
| **3**[c] | 440(63642), 451(sh)(52114),510(4080), 542(4951), 562(6853), 589(17093), 613(25848) | |
| **4**[c] | 435(71952), 448(sh)(57914),507(4278), 539(5432), 563(8194), 584(17159), 611(31352) | |

[a]In dichloromethane.

[b]In Acetonitrile.

[c]In Pyridin

**Table S2** Crystallographic Data for **1**,**3** and **4.**

| compound code | **1** | **3** | **4** |
|---|---|---|---|
| molecular formula | $\mathbf{C_{51}H_{38}F_{10}N_6O_2}$ | $\mathbf{C_{64.5}H_{44.75}CoF_{10}N_9O_{1.25}}$ | $\mathbf{C_{108}H_{78}Co_2F_{19.97}N_{12}}$ |
| Fw | 956.87 | 1214.77 | 2041.17 |
| Radiation | Cu Kα (λ = 1.54184) | Mo Kα (λ = 0.71073) | Mo Kα (λ = 0.71073) |
| diffractometer | Rigaku Oxford | Rigaku Oxford | Rigaku Oxford |
| crystal system | triclinic | monoclinic | triclinic |
| space group | P-1 | $P2_1/c$ | P-1 |
| $a$(Å) | 11.4554(2) | 21.5204(6) | 14.3457(3) |
| $b$(Å) | 14.6770(3) | 15.0216(4) | 16.6438(4) |
| $c$(Å) | 15.3467(3) | 17.9534(5) | 20.9055(6) |
| α (°) | 117.434(2) | 90 | 102.642(2) |
| β (°) | 103.340(2) | 108.800(3) | 103.348(2) |
| γ (°) | 99.822(2) | 90 | 96.554(2) |
| $V$(Å$^3$) | 2110.65(8) | 5494.2(3) | 4667.1(2) |
| Ranges (h, k, l) | $-14 \le h \le 14$ | $-28 \le h \le 26$ | $-19 \le h \le 17$ |
| | $-18 \le k \le 17$ | $-18 \le k \le 20$ | $-21 \le k \le 22$ |
| | $-14 \le l \le 19$ | $-22 \le l \le 22$ | $-27 \le l \le 28$ |
| Z | 2 | 4 | 2 |
| Colour | dark purple | dark purple | dark purple |
| Crystal dimen (mm) | 0.2× 0.2 × 0.1 | 0.2× 0.2 × 0.1 | 0.2× 0.2 × 0.1 |
| $\mu$ (mm$^{-1}$) | 1.065 | 0.401 | 0.453 |
| $T$(K) | 99.97(10) | 293(2) | 100.00(10) |
| $D_{calcd}$ (g cm$^{-3}$) | 1.506 | 1.469 | 1.452 |
| $2\theta$ range (deg) | 6.9 to 155.36 | 3.618 to 61.034 | 3.666 to 60.238 |
| $e$ data ($R_{int}$) | 8775 (0.0596) | 13573(0.0544) | 21848(0.0921) |
| Parameters | 638 | 847 | 21848 |
| Restraints | 0 | 0 | 0 |
| $R_1$ ($I>2\sigma(I)$) | 0.0529 | 0.0598 | 0.0659 |
| $WR_2$ (all data) | 0.1532 | 0.1558 | 0.1700 |
| GOOF | 1.060 | 1.026 | 1.031 |
| $\Delta\rho_{max}$, $\Delta\rho_{min}$ (e Å$^{-3}$) | 0.43, -0.29 | 1.27, -0.96 | 0.78, -0.51 |

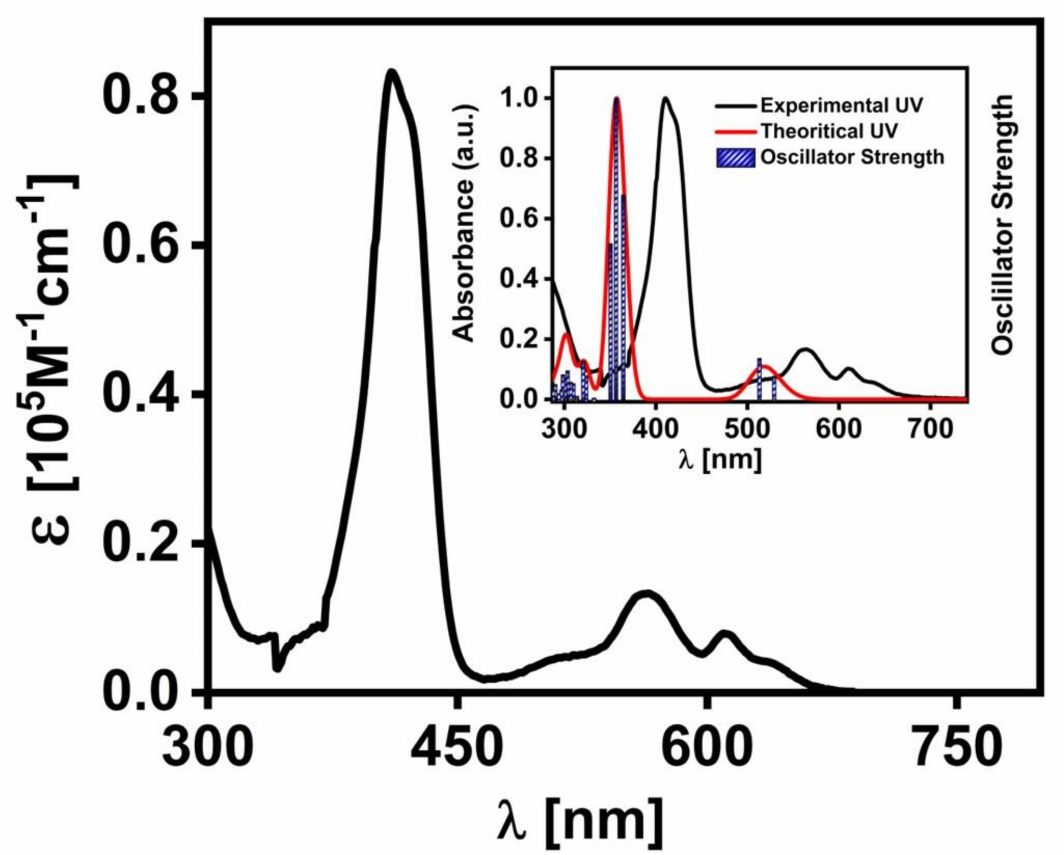


**Figure S1** Electronic absorption spectrum of **1** in dichloromethane. Inset FB Corrole, **1** in dichloromethane overlaid with calculated vertical excitation energies (blue lines) obtained from TD-DFT (B3LYP/6-311G(d,p) for atoms; solvent = $CH_2Cl_2$, PCM model).

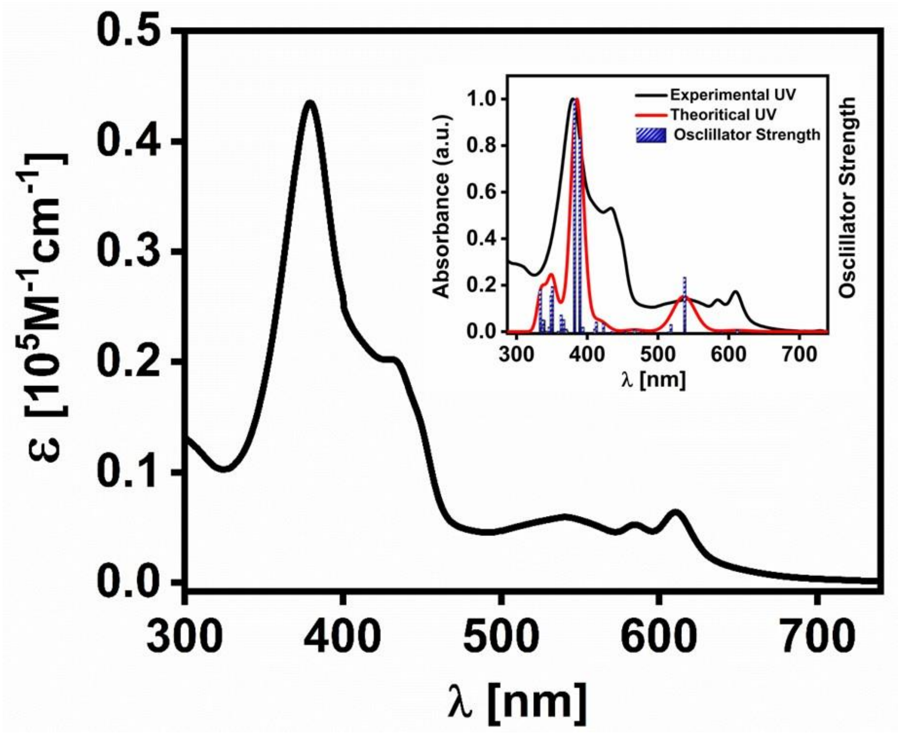


**Figure S2** Electronic absorption spectrum of **3** in Acetonitrile. Inset Corrolato cobalt (III) complex, **3** in $CH_3CN$ overlaid with calculated vertical excitation energies (blue lines) obtained from TD-DFT (B3LYP/6-311G(d,p) for light atoms and LANL2DZ for Co; solvent $CH_3CN$, PCM model).

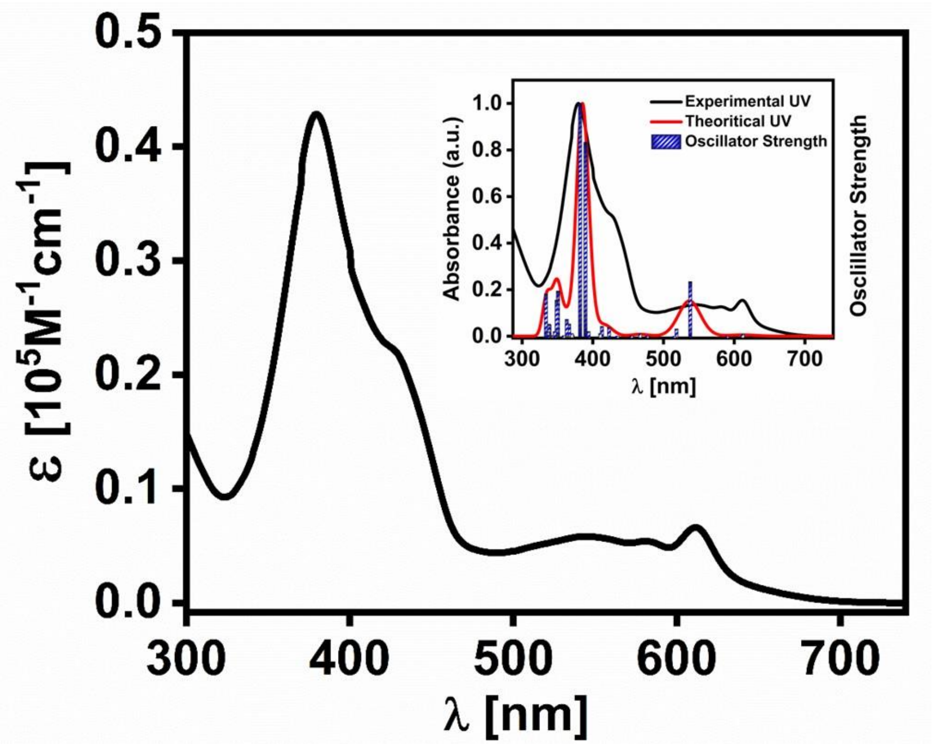


**Figure S3** Electronic absorption spectrum of **4** in Acetonitrile. Inset Corrolato cobalt (III) complex, **4** in $CH_3CN$ overlaid with calculated vertical excitation energies (blue lines) obtained from TD-DFT (B3LYP/6-311G(d,p) for light atoms and LANL2DZ for Co; solvent $CH_3CN$, PCM model).

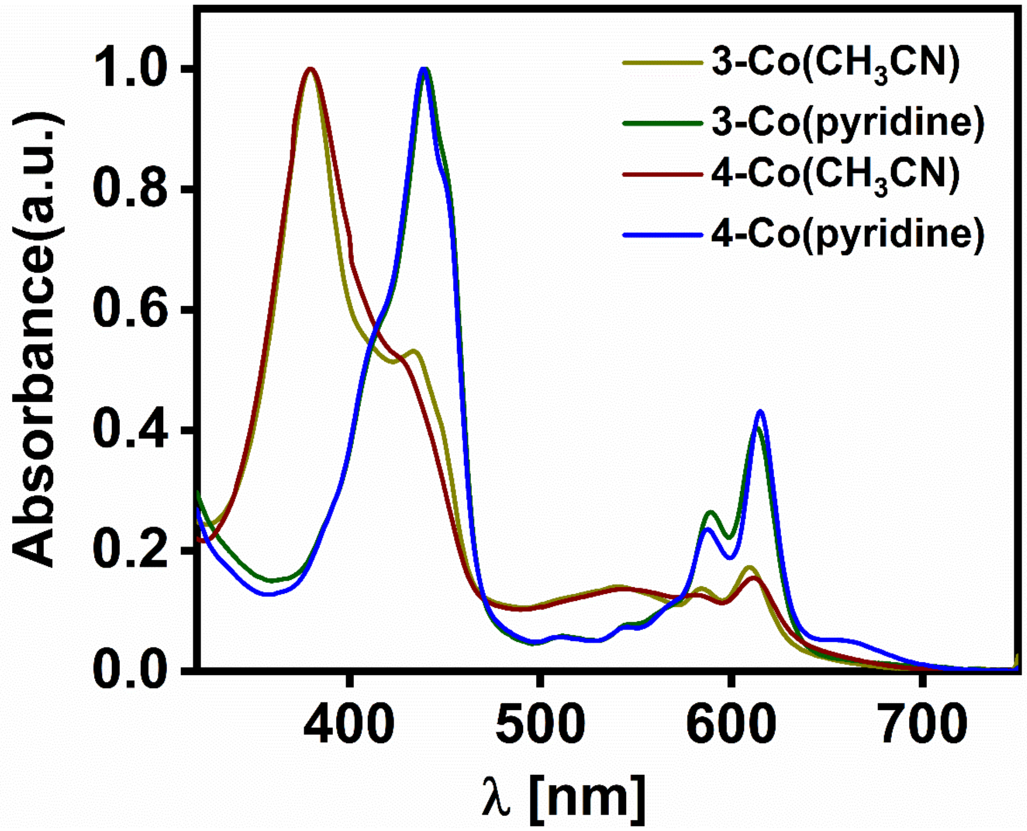


**Figure S4** Overlap of UV-spectrum of compound **3** and **4** in acetonitrile and pyridine.

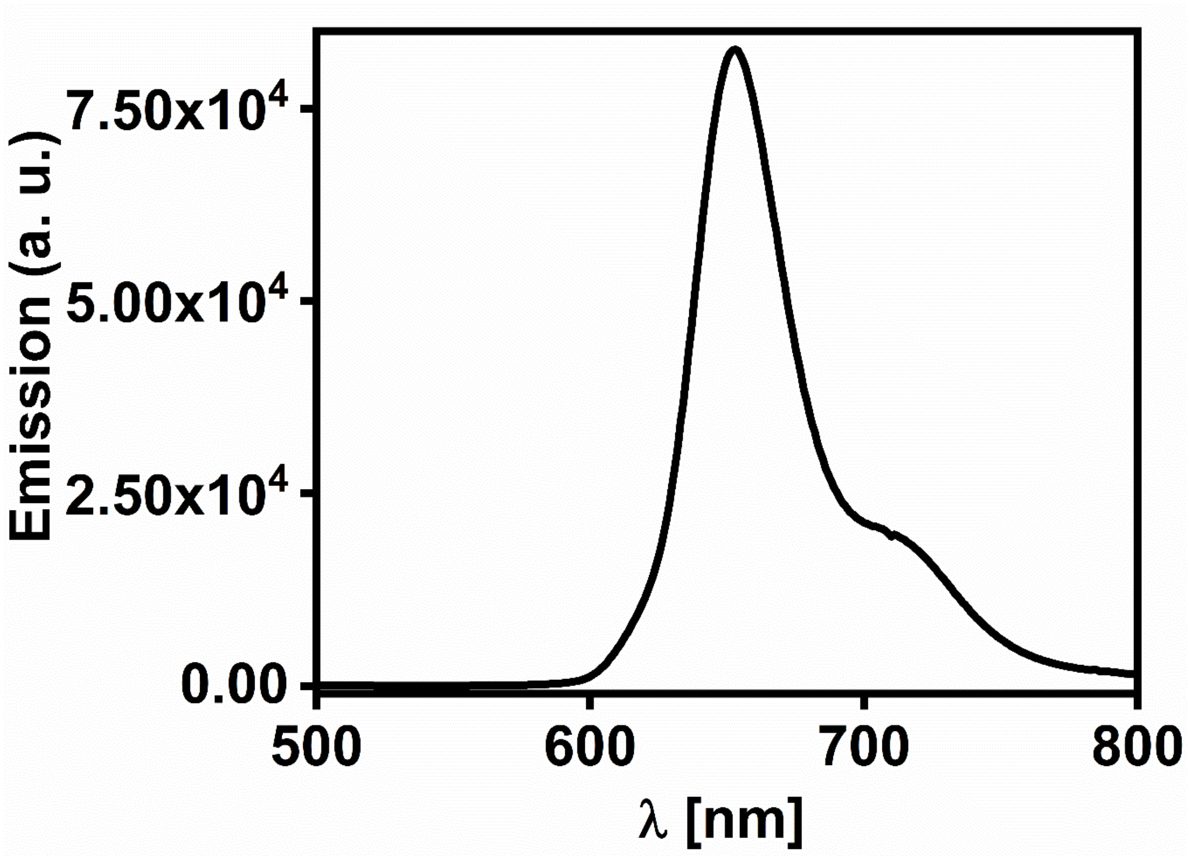


**Figure S5** Electronic emission spectrum (excited at 410 nm) of **1** in dichloromethane.

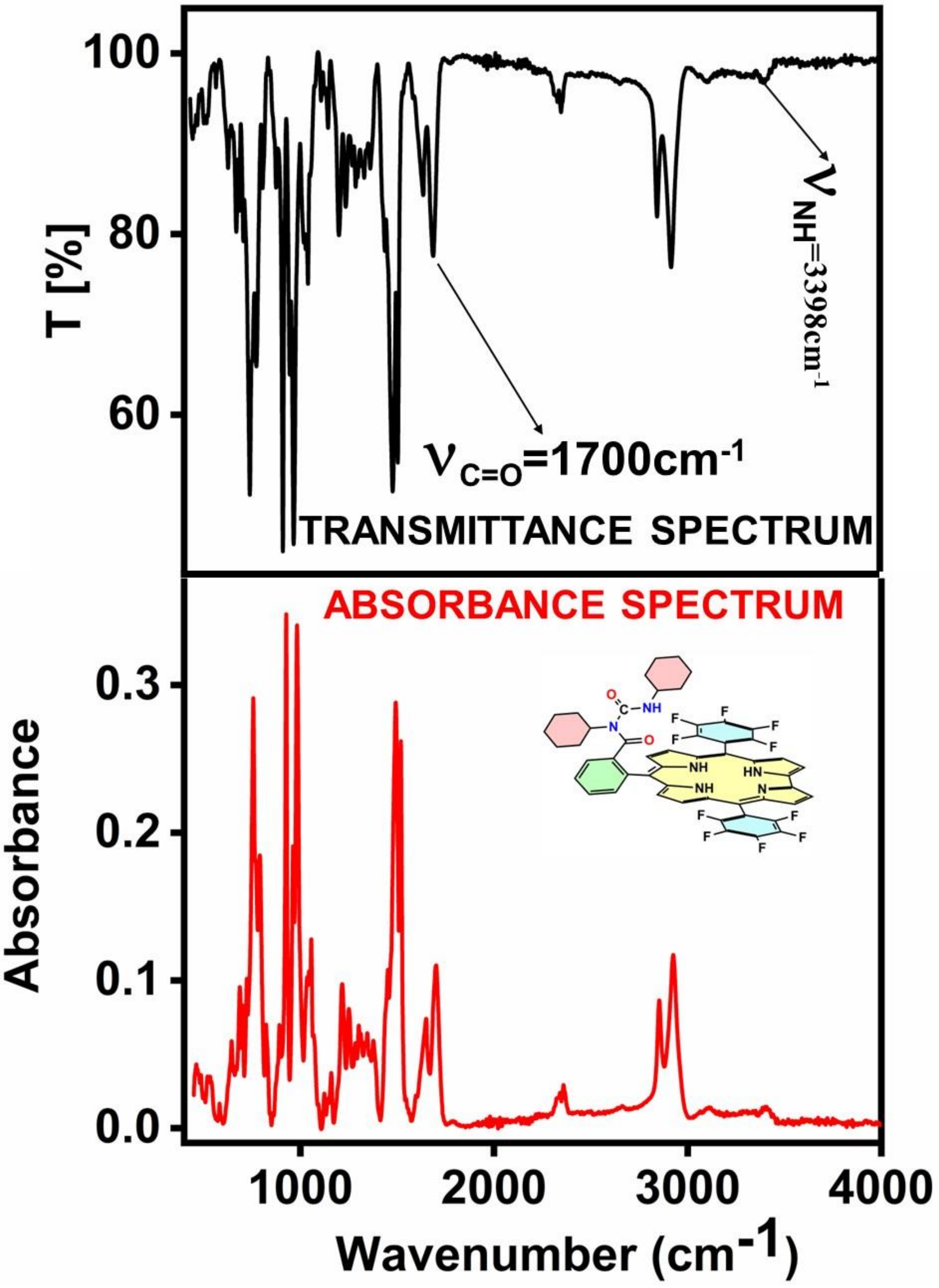


**Figure S6** FT-IR spectrum of **1** as a KBr pellet.

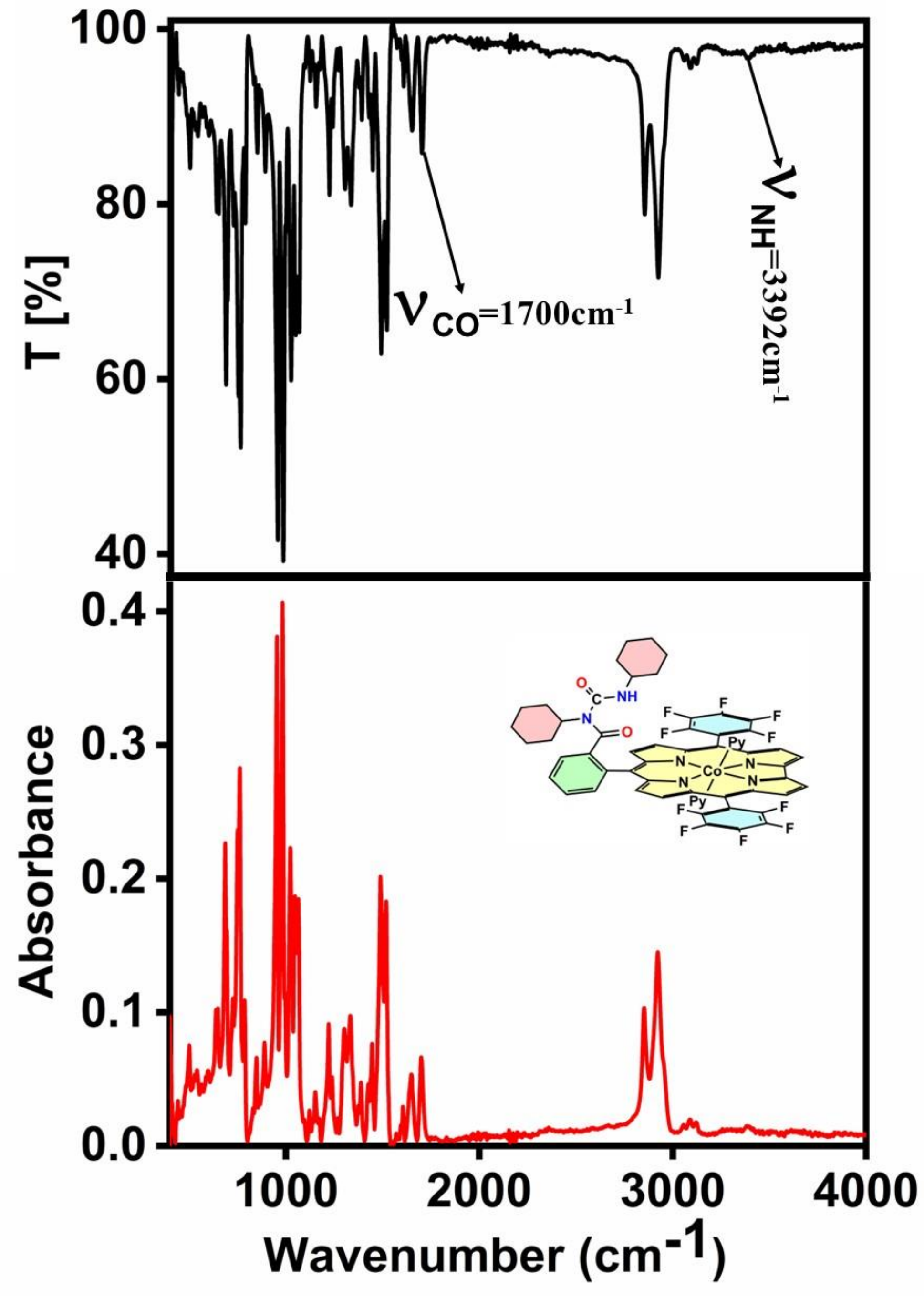


**Figure S7** FT-IR spectrum of **3** as a KBr pellet.

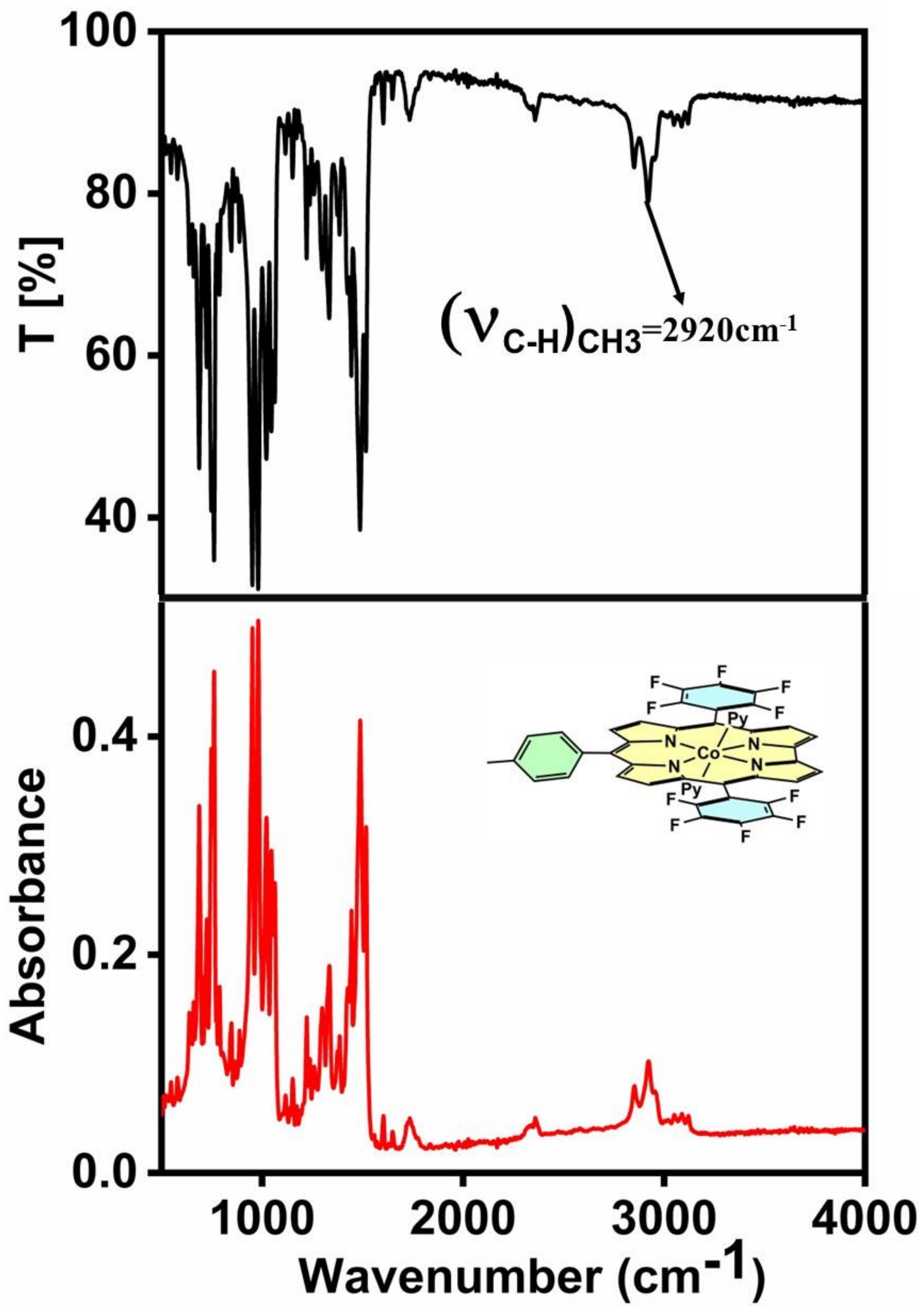


**Figure S8** FT-IR spectrum of **4** as a KBr pellet.

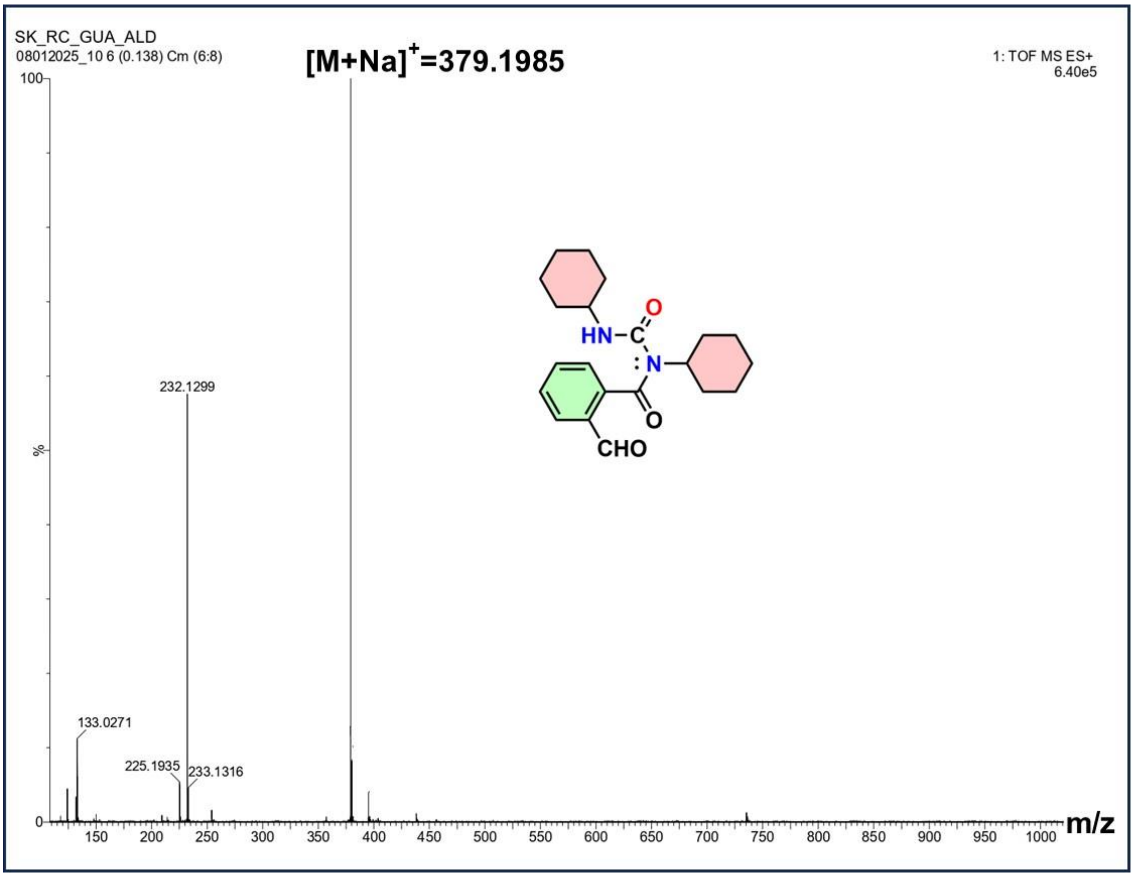


**Figure S9** **(a)** ESI- MS spectrum of N-cyclohexyl-N-(cyclohexylcarbamoyl)-2-formylbenzamide in $CH_3CN$ shows the measured spectrum.

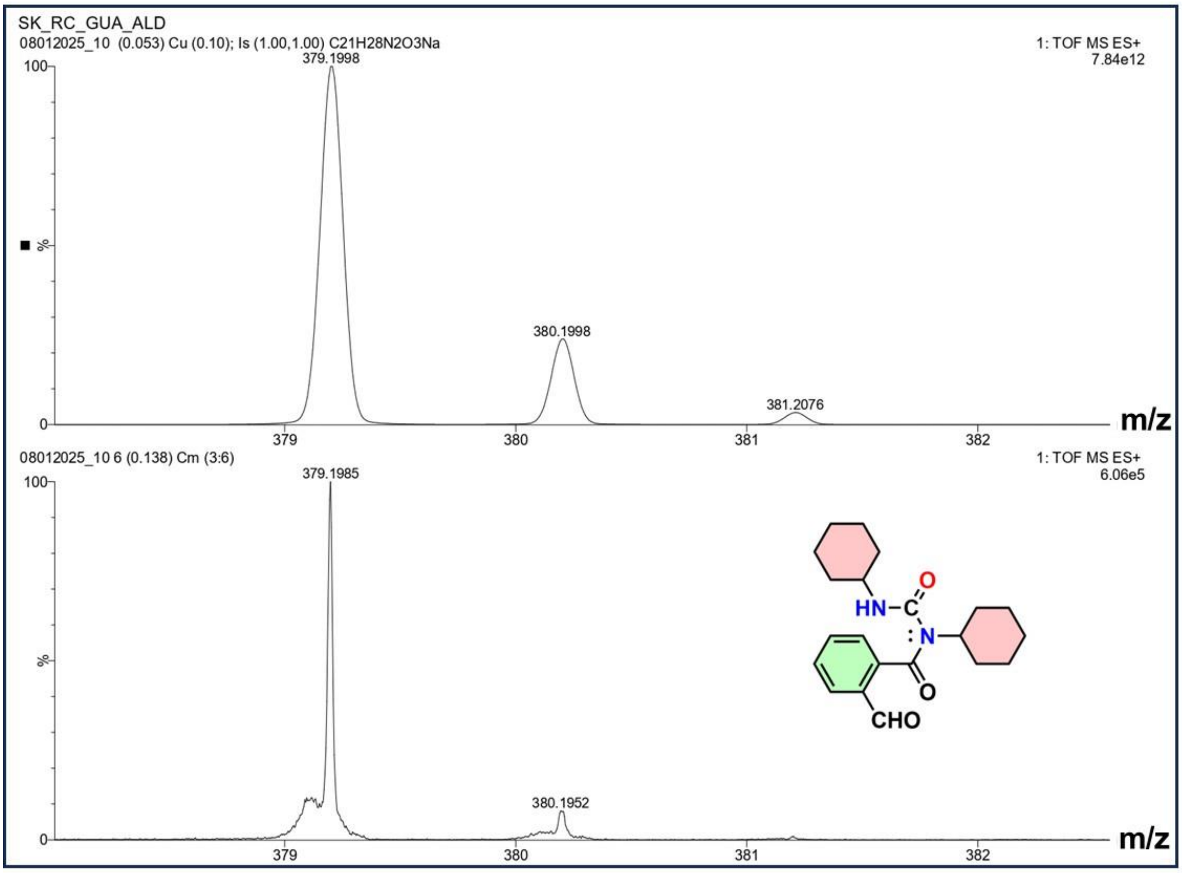


**Figure S9** **(b)** ESI- MS spectrum of N-cyclohexyl-N-(cyclohexylcarbamoyl)-2-formylbenzamide in $CH_3CN$ shows the measured spectrum with isotopic distribution pattern.

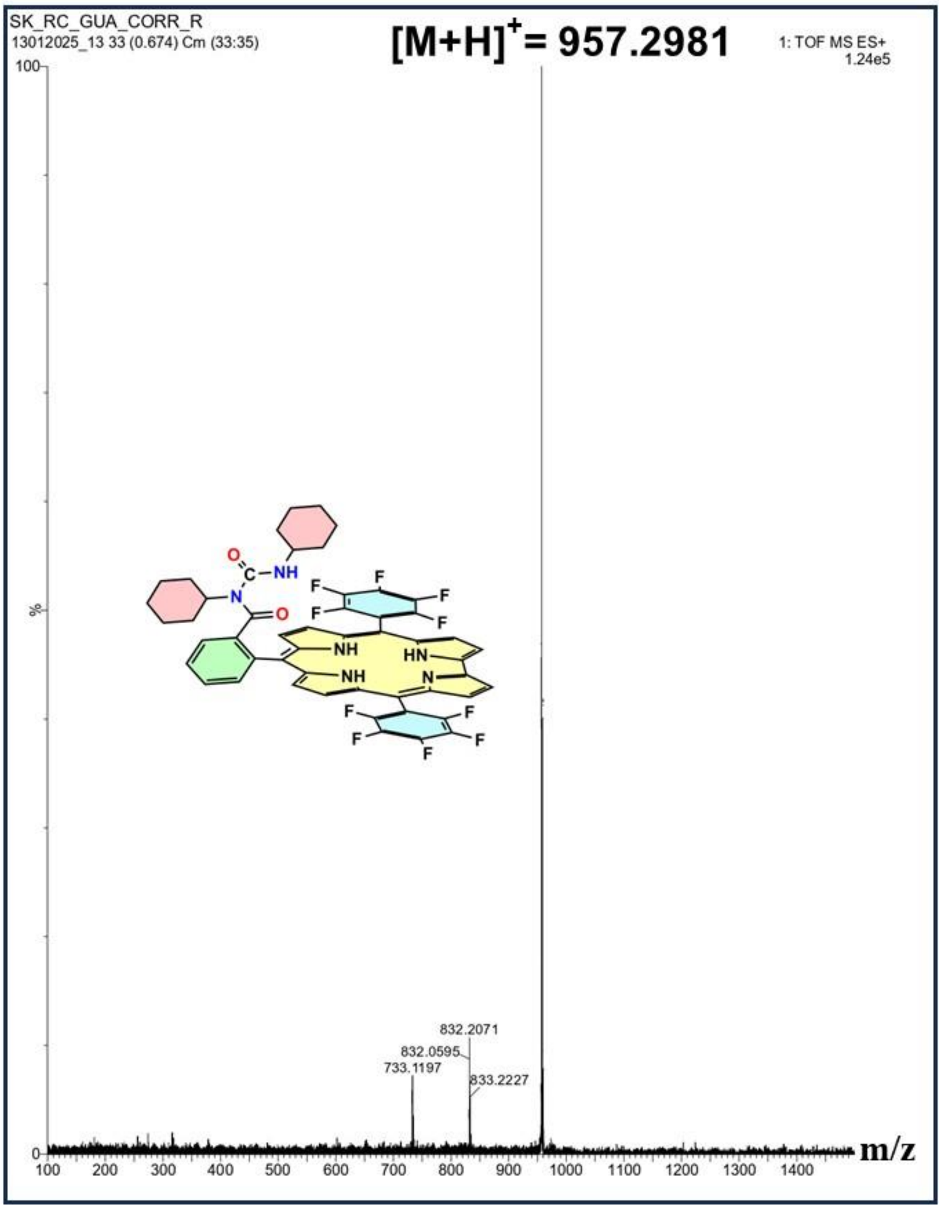


**Figure S10** **(a)** ESI- MS spectrum of **1** in $CH_3CN$ shows the measured spectrum.

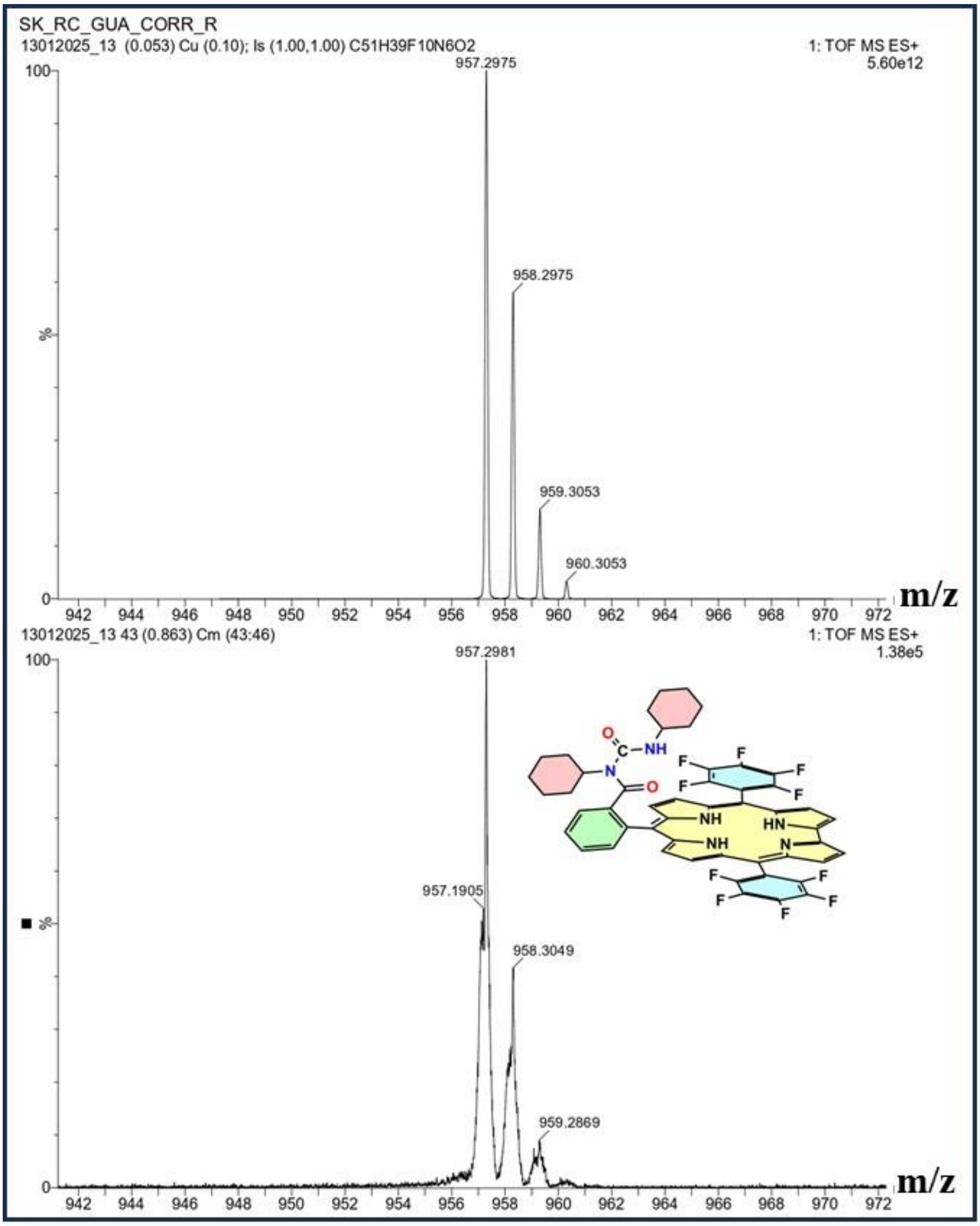


**Figure S10** **(b)** ESI- MS spectrum of **1** in $CH_3CN$ shows the measured spectrum with isotopic distribution pattern.

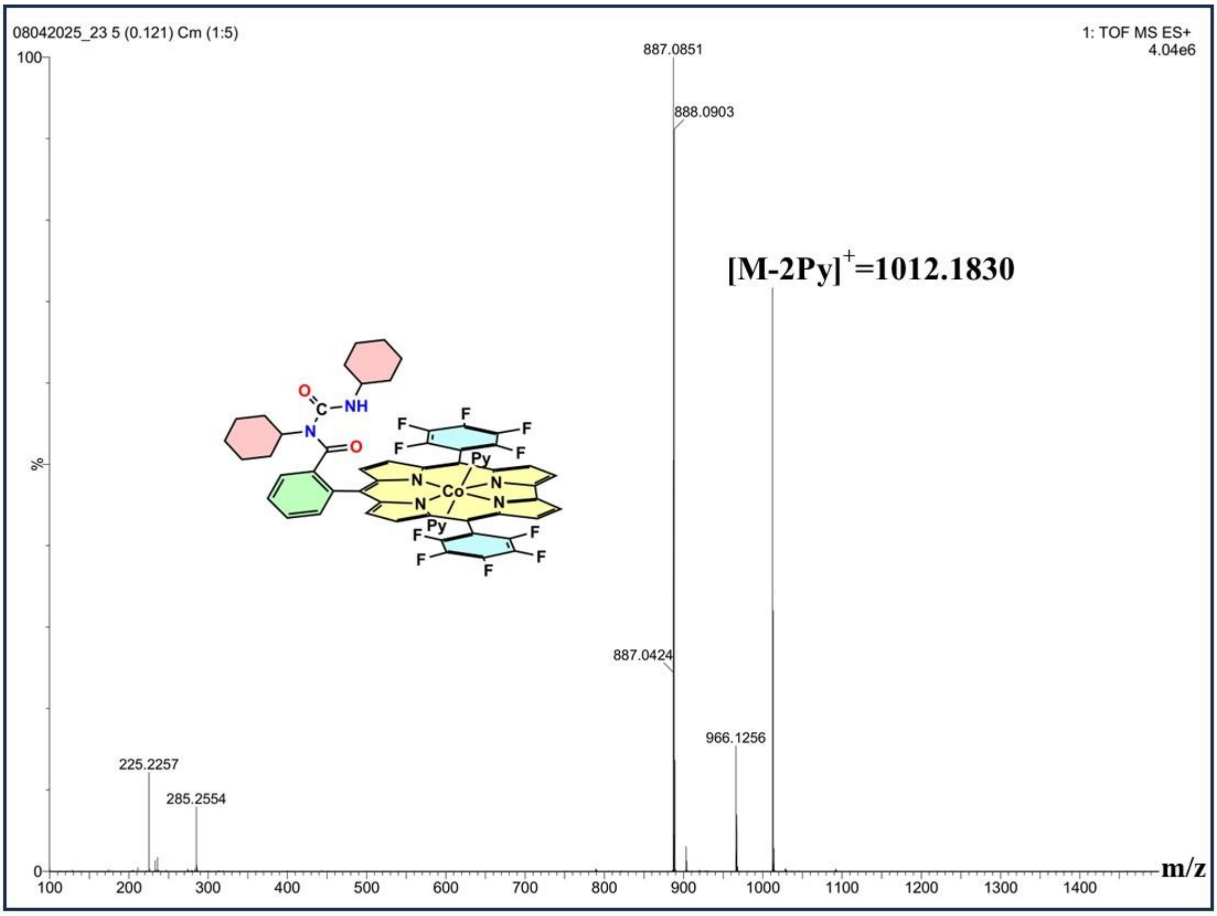


**Figure S11** **(a)** ESI- MS spectrum of **3** in $CH_3CN$ shows the measured spectrum.

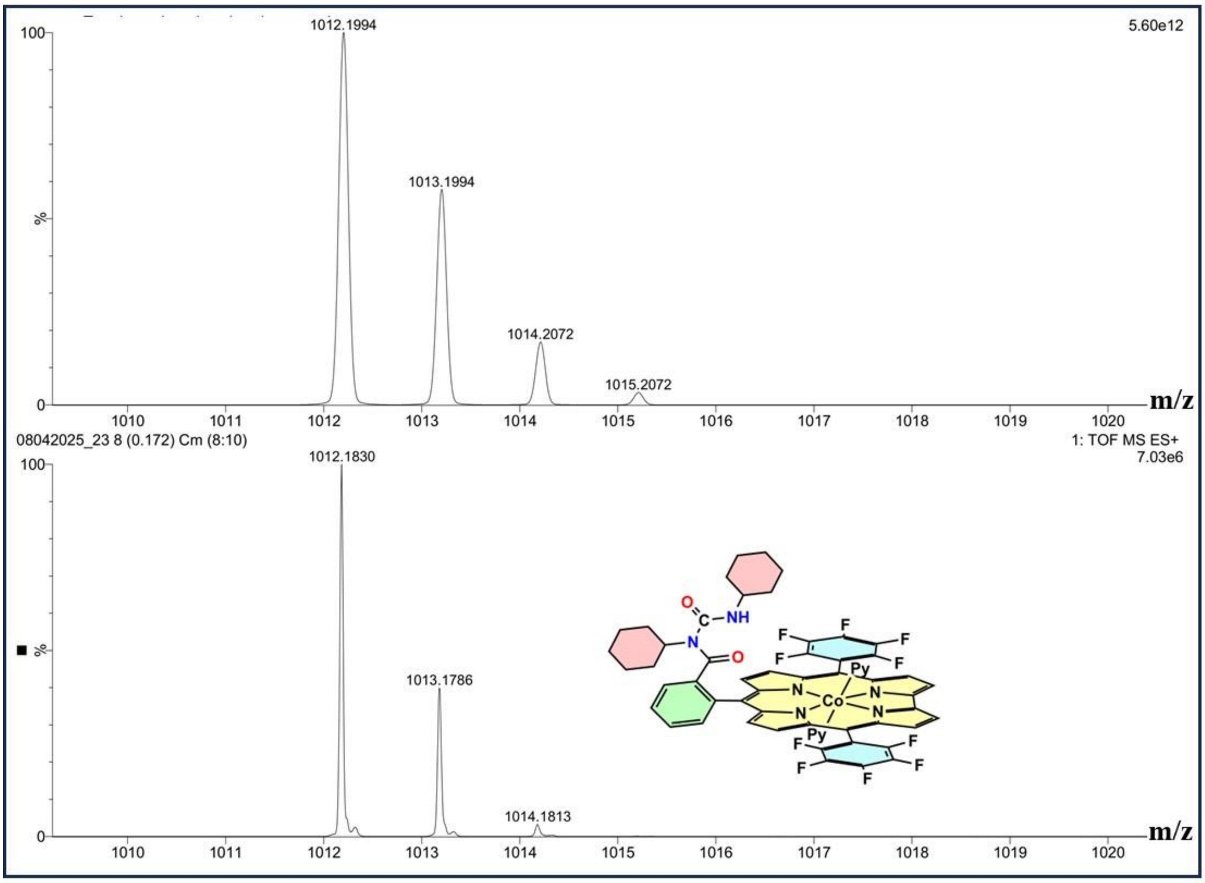


**Figure S11** **(b)** ESI- MS spectrum of **3** in $CH_3CN$ shows the measured spectrum with isotopic distribution pattern.

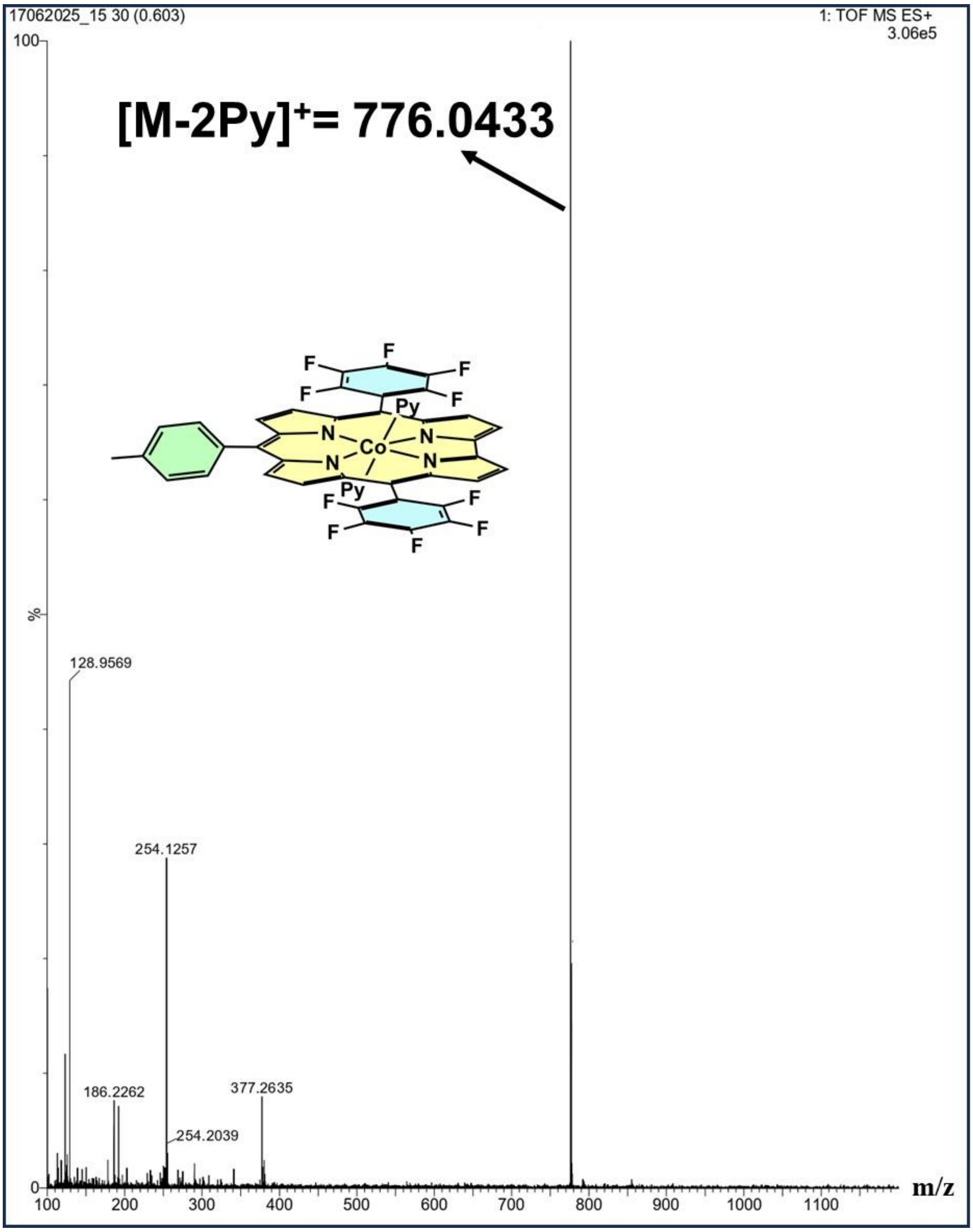


**Figure S12** **(a)** ESI- MS spectrum of **4** in $CH_3CN$ shows the measured spectrum.

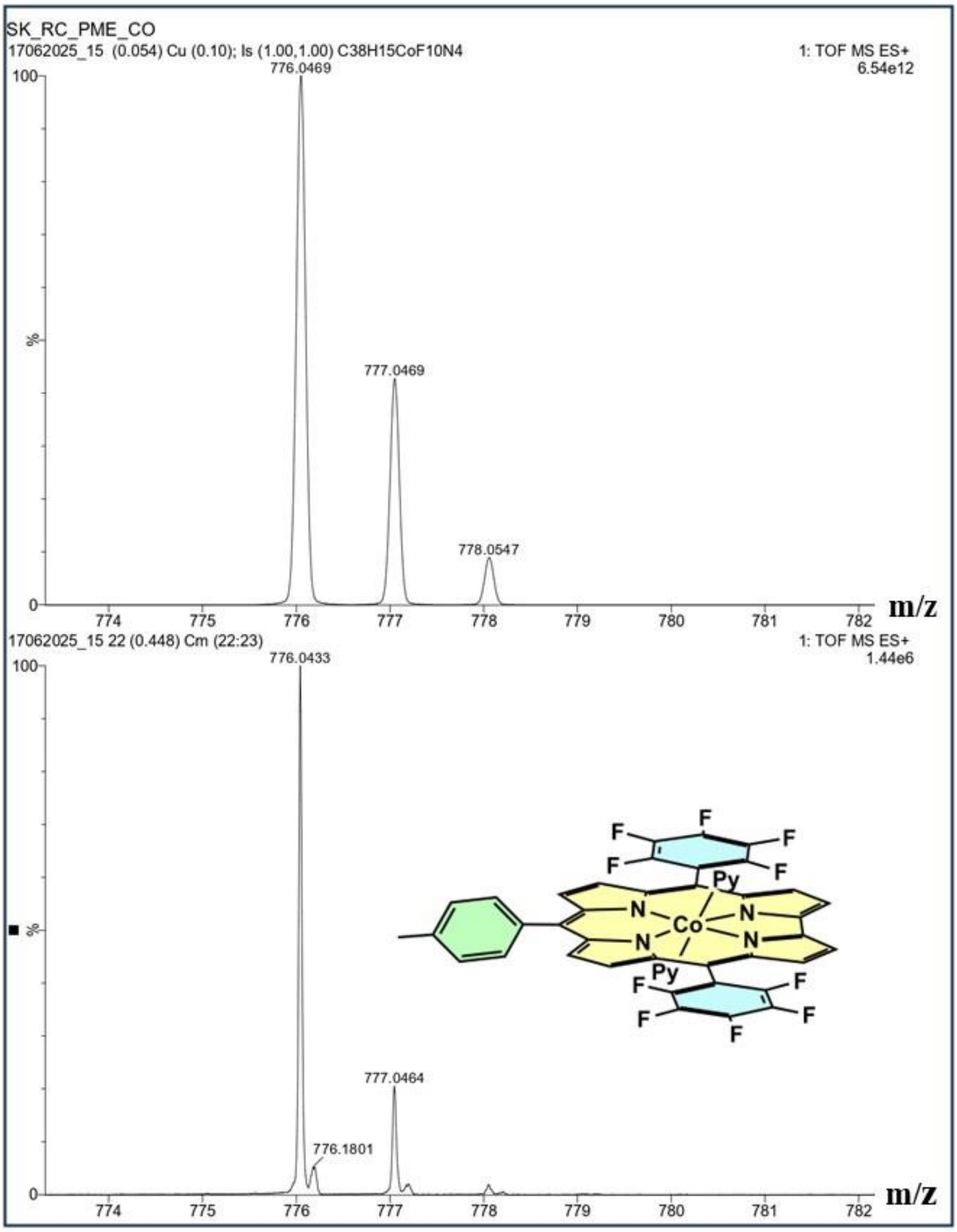


**Figure S12** **(b)** ESI- MS spectrum of **4** in $CH_3CN$ shows the measured spectrum with isotopic distribution pattern.

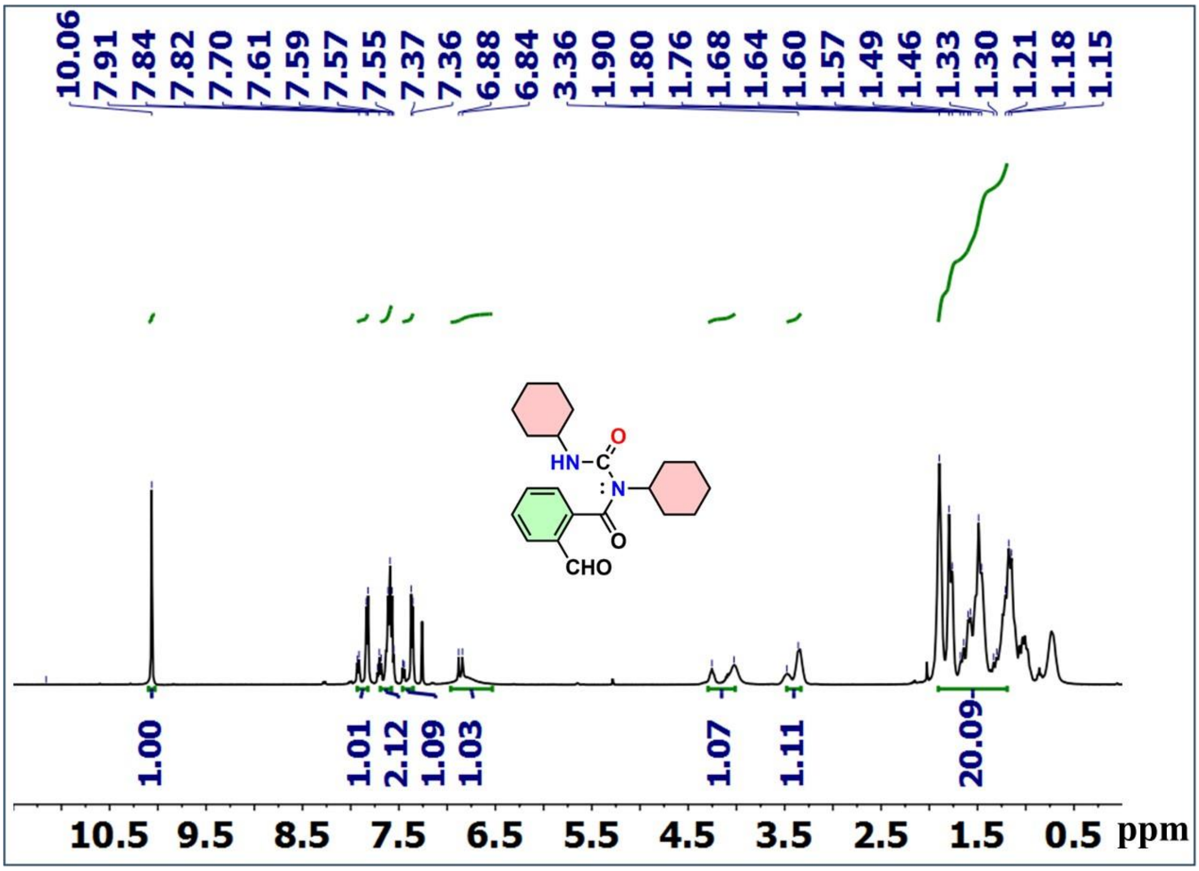


**Figure S13** $^{1}$H NMR (400 MHz) spectrum of N-cyclohexyl-N-(cyclohexylcarbamoyl)-2-formylbenzamide in $CDCl_3$.

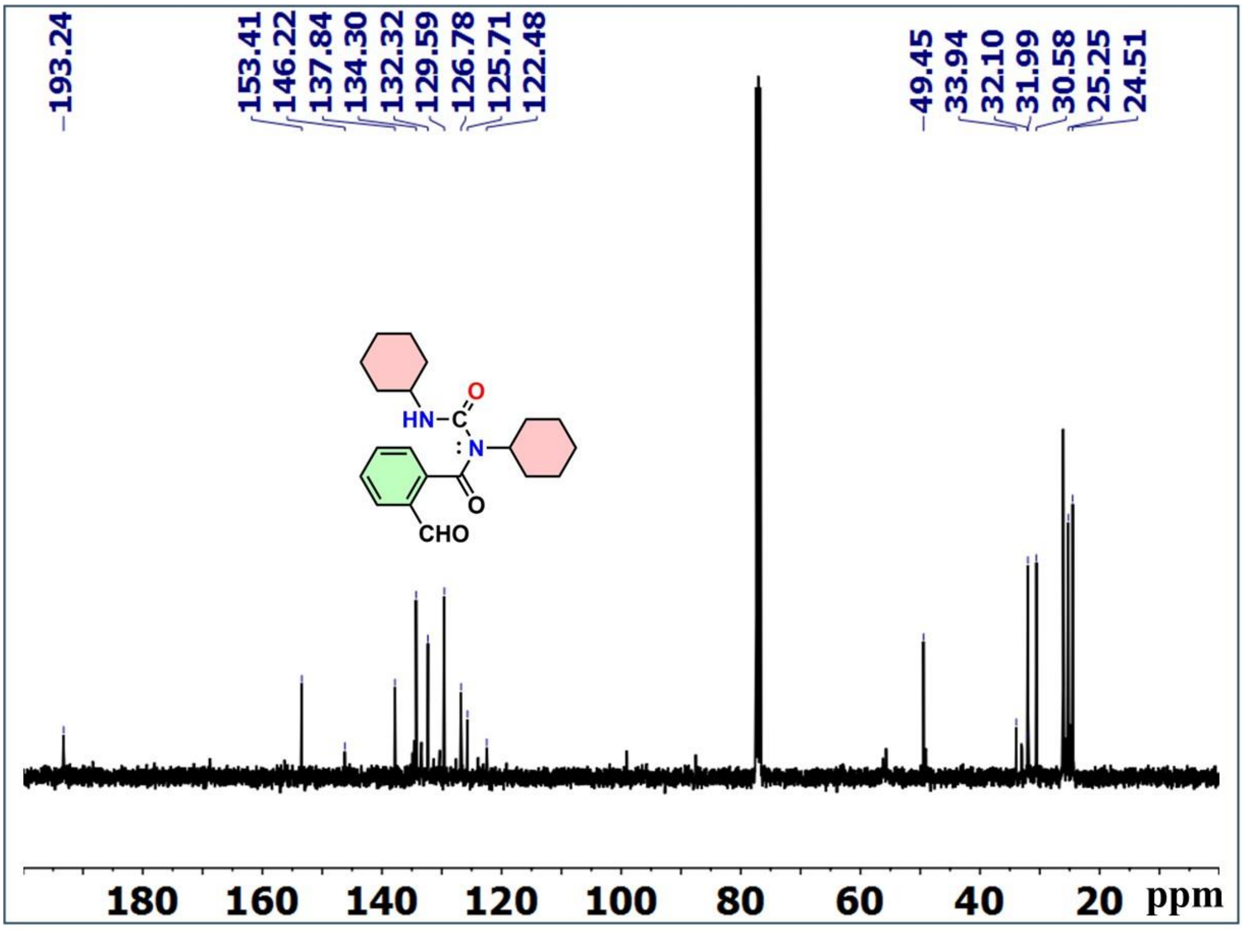


**Figure S14** $^{13}$C NMR (101 MHz) spectrum of N-cyclohexyl-N-(cyclohexylcarbamoyl)-2-formylbenzamide in $CDCl_3$.

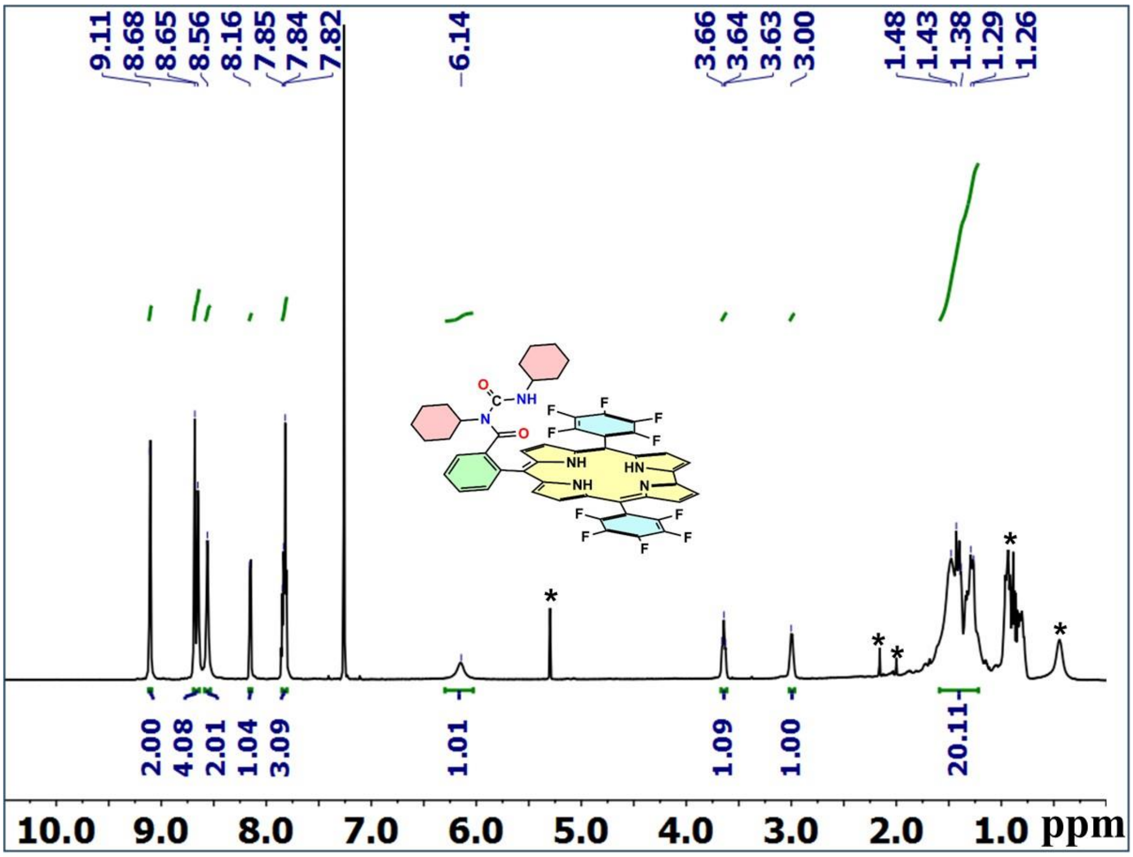


**Figure S15** $^1$H NMR (700 MHz) spectrum of **1** in $CDCl_3$.

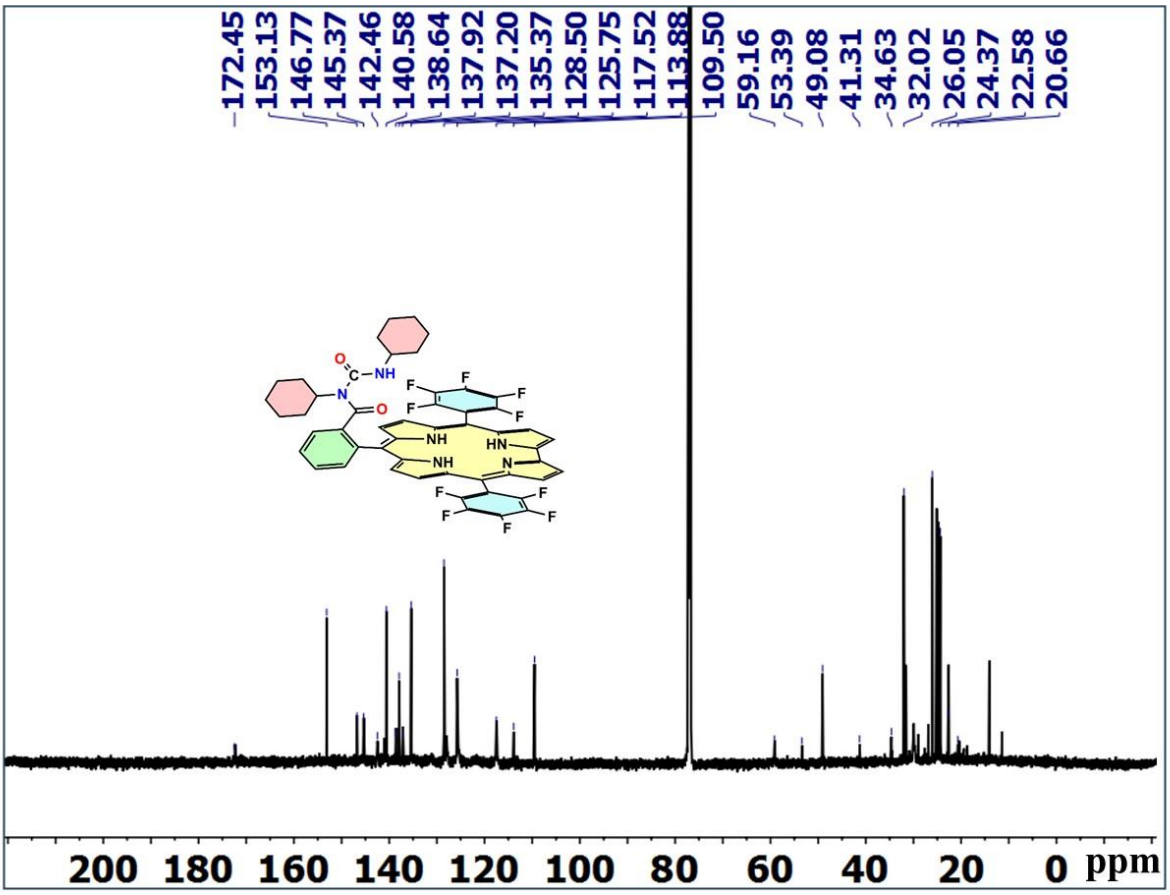


**Figure S16** $^{13}$C NMR (176 MHz) spectrum of **1** in $CDCl_3$.

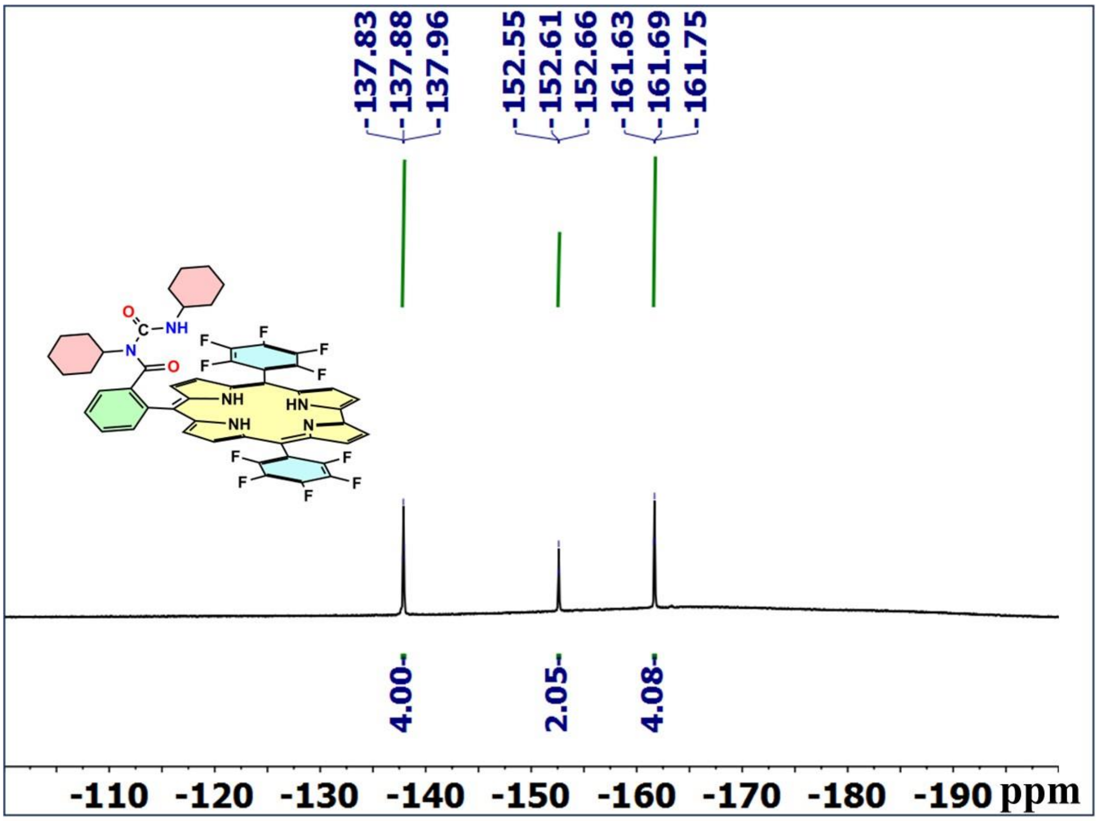


**Figure S17** $^{19}$F NMR (377 MHz) spectrum of **1** in $CDCl_3$.

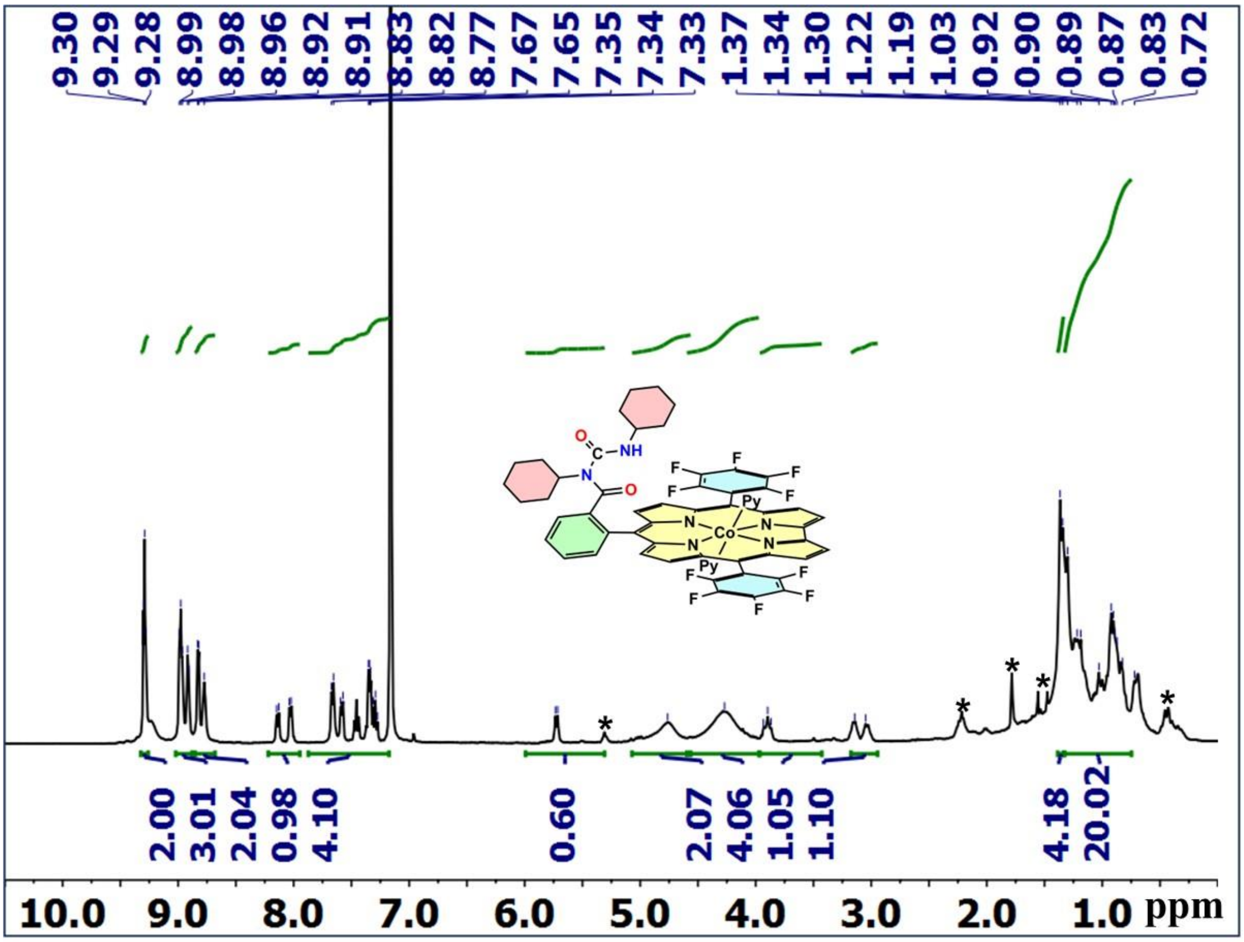


**Figure S18** $^1$H NMR (400 MHz) spectrum of **3** in $C_6D_6$.

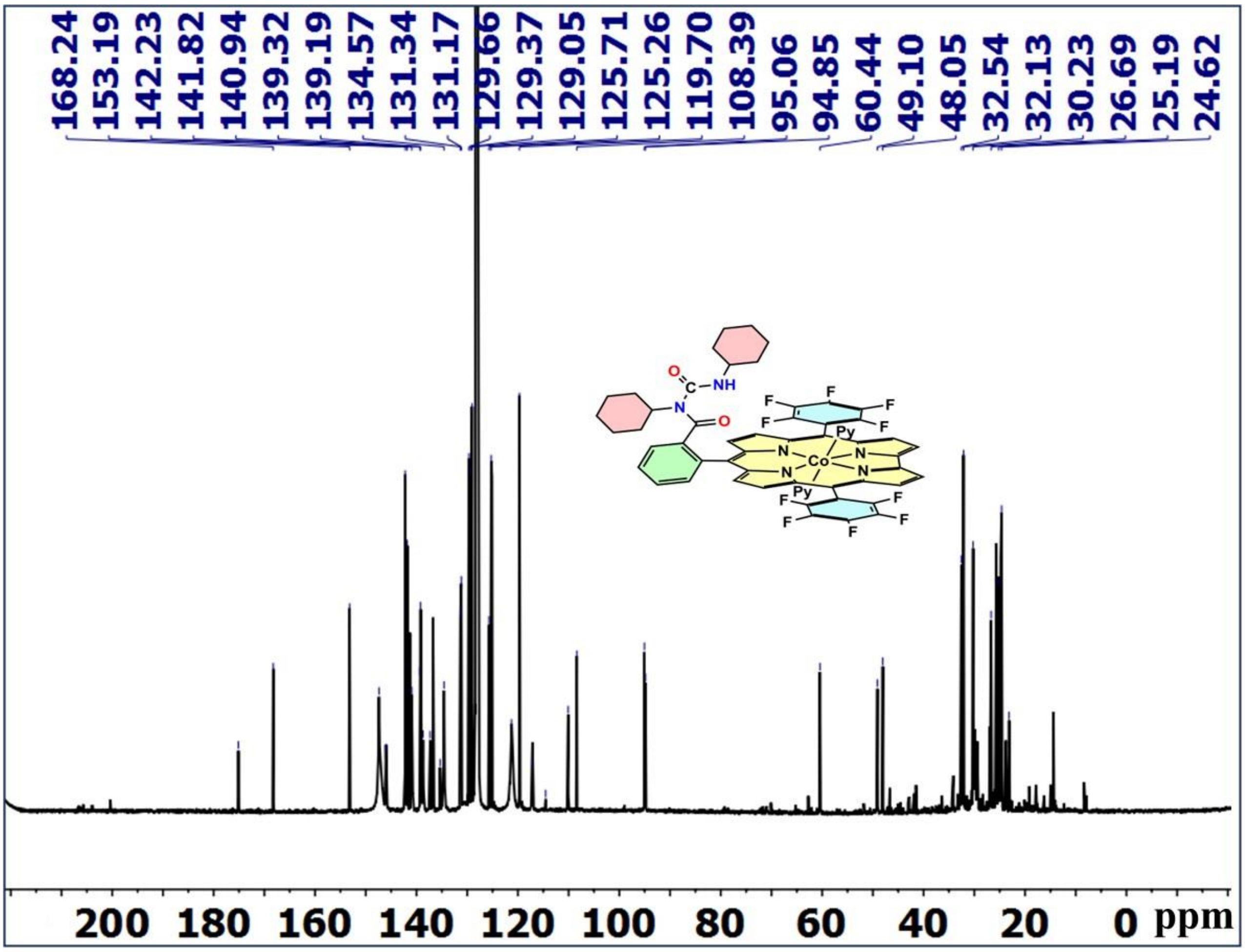


**Figure S19** $^{13}C$ NMR (176 MHz) spectrum of **3** in $C_6D_6$.

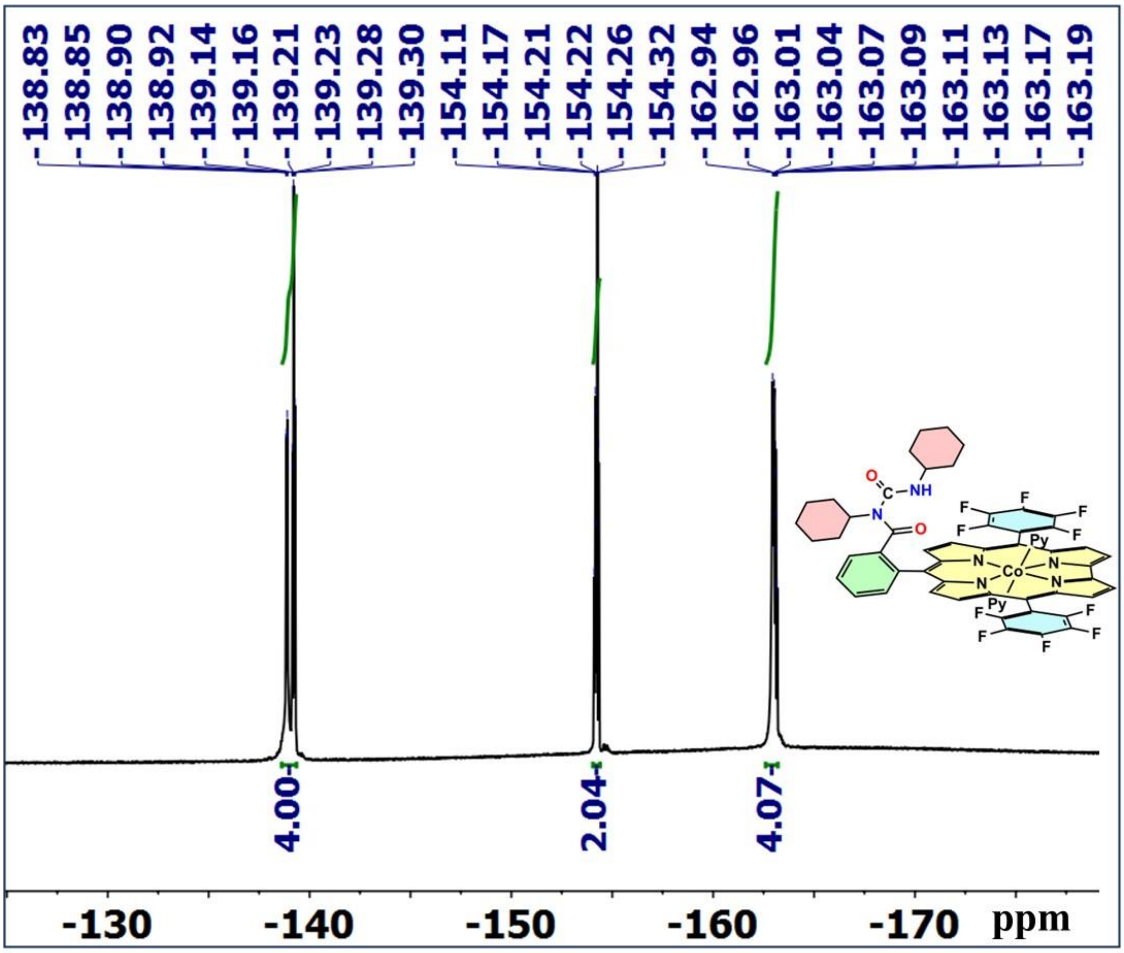


**Figure S20** $^{19}$F NMR (377 MHz) spectrum of **3** in $C_6D_6$.

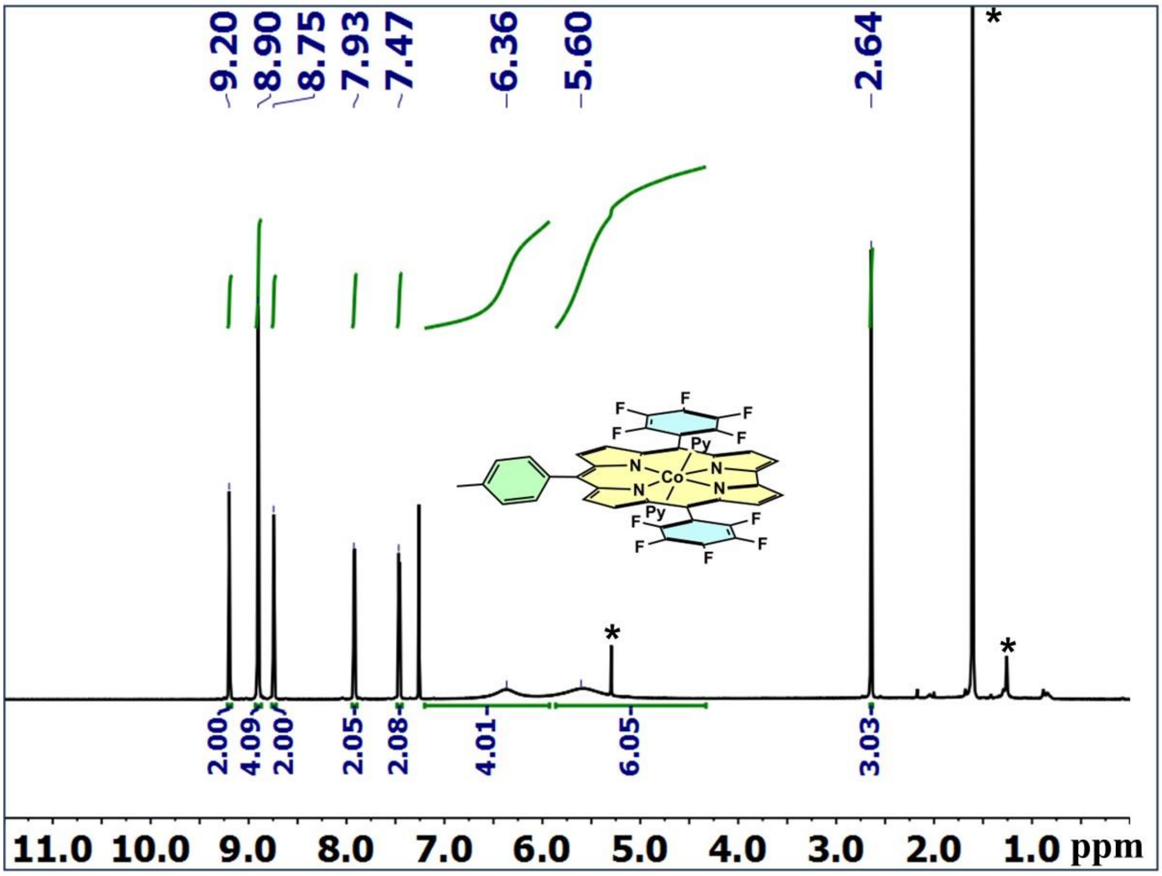


**Figure S21** $^{1}$H NMR (700 MHz) spectrum of **4** in $CDCl_3$.

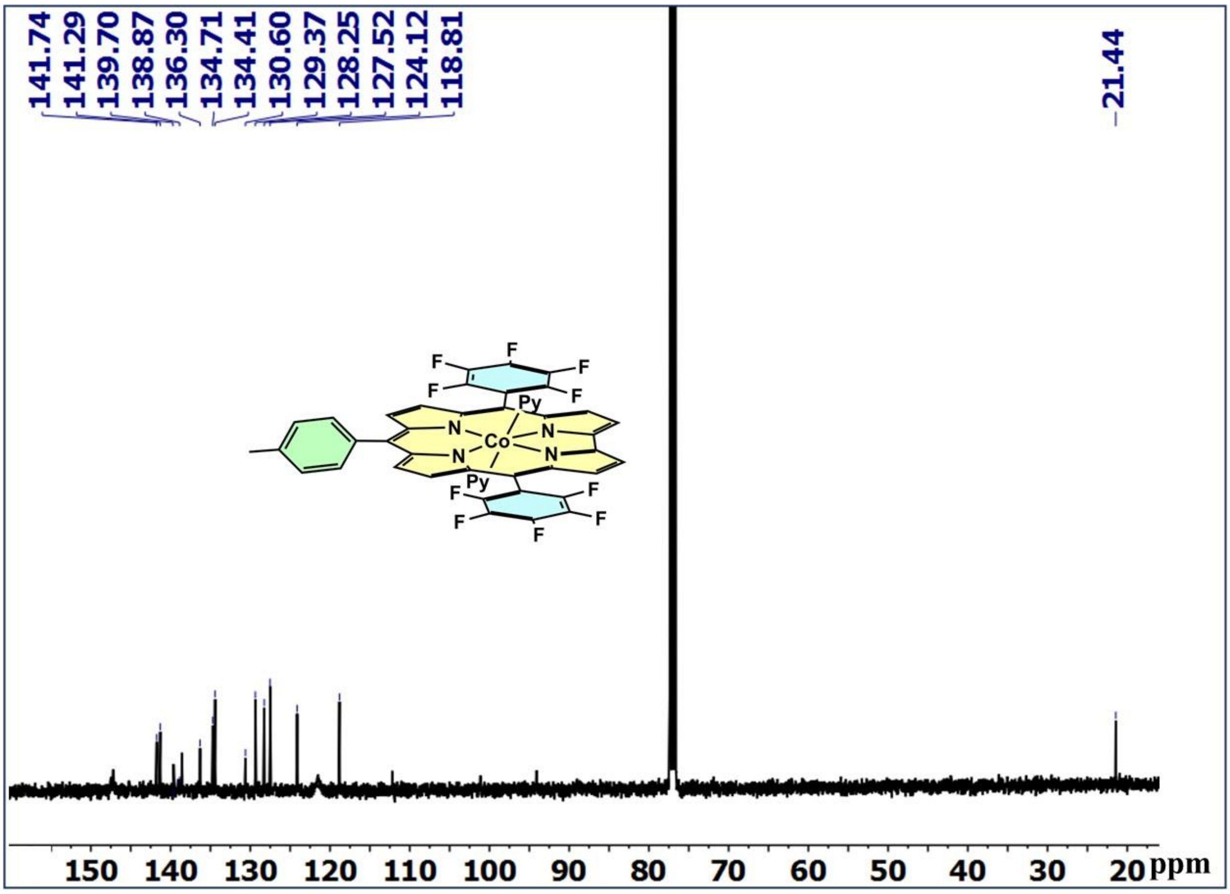


**Figure S22** $^{13}C$ NMR (101 MHz) spectrum of **4** in $CDCl_3$.

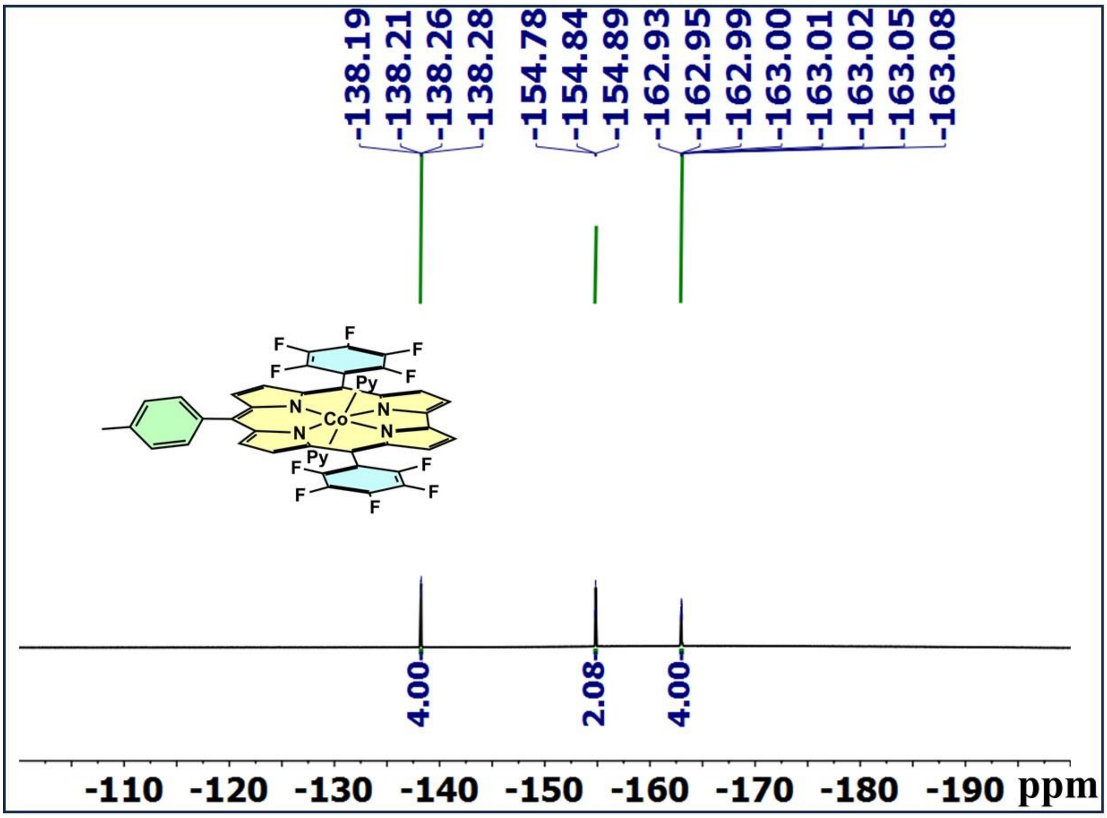


**Figure S23** $^{19}F$ NMR (377 MHz) spectrum of **4** in $CDCl_3$.

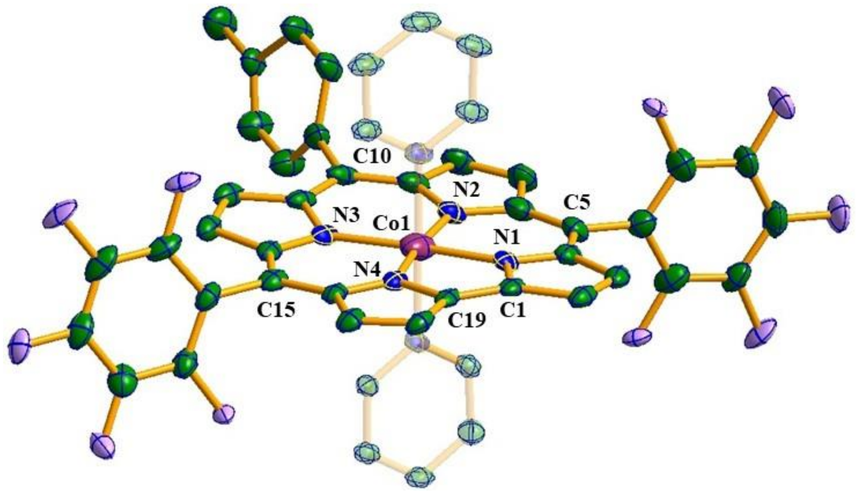


**Figure S24** Single-crystal X-ray structure of compound **4**. Hydrogen atoms are omitted for clarity. Thermal ellipsoids are drawn at the 50% probability level.

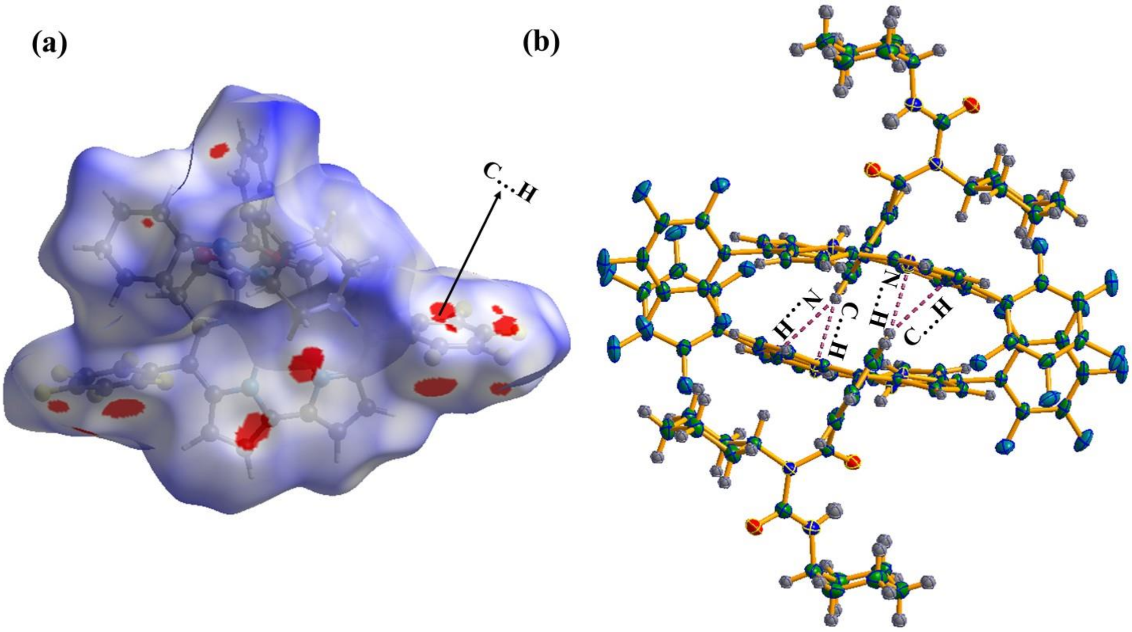


**Figure S25** (a) Hirshfeld surface, and (b) Hydrogen bonding interactions in **1**.

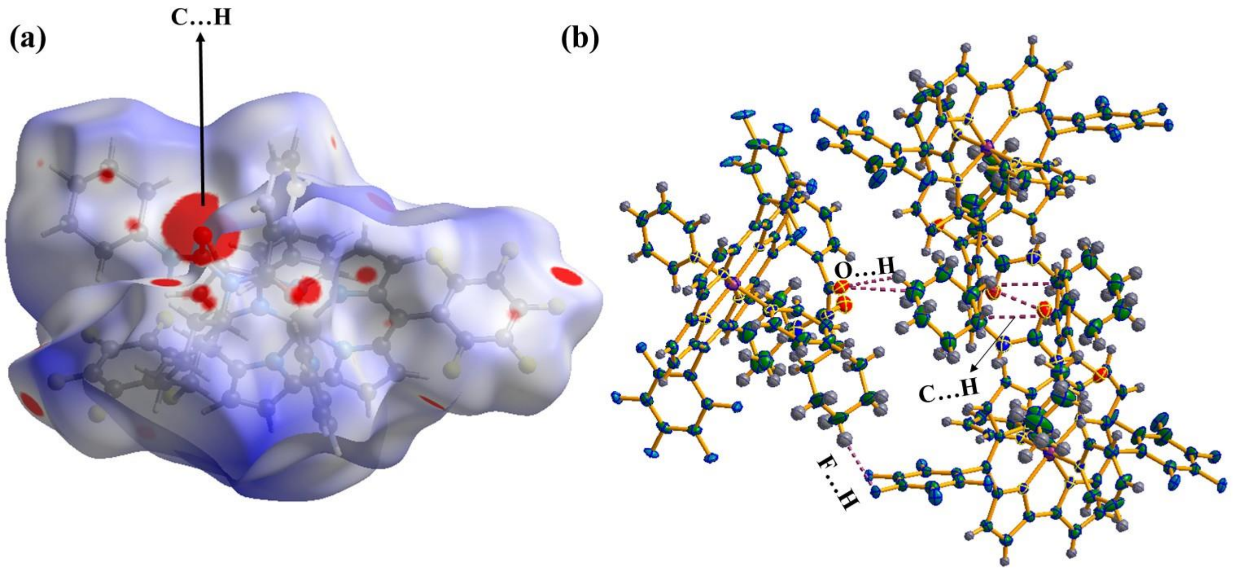


**Figure S26** (a) Hirshfeld surface, and (b) Hydrogen bonding interactions in **3**.

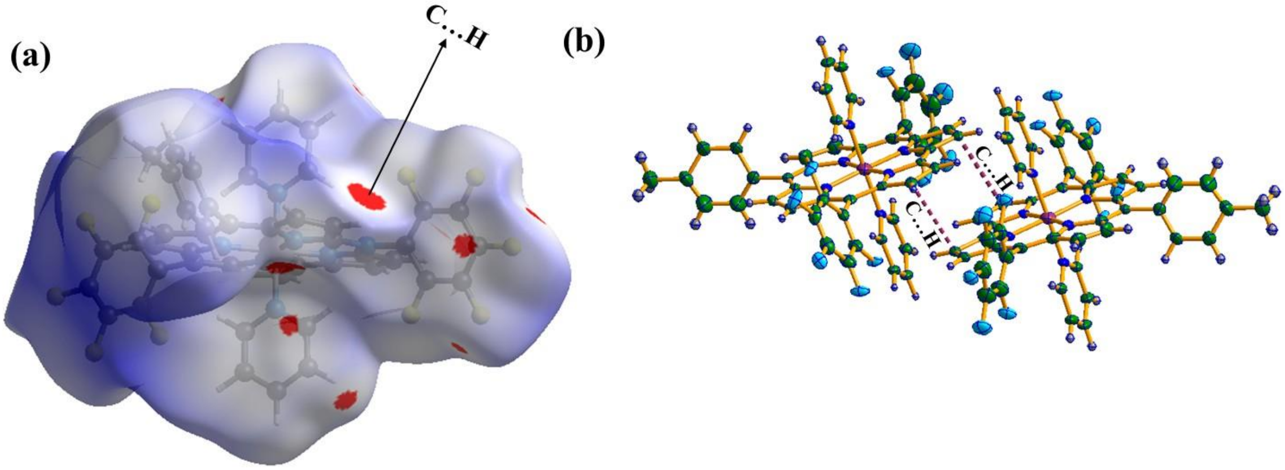


**Figure S27** (a) Hirshfeld surface, and (b) Hydrogen bonding interactions in **4**.

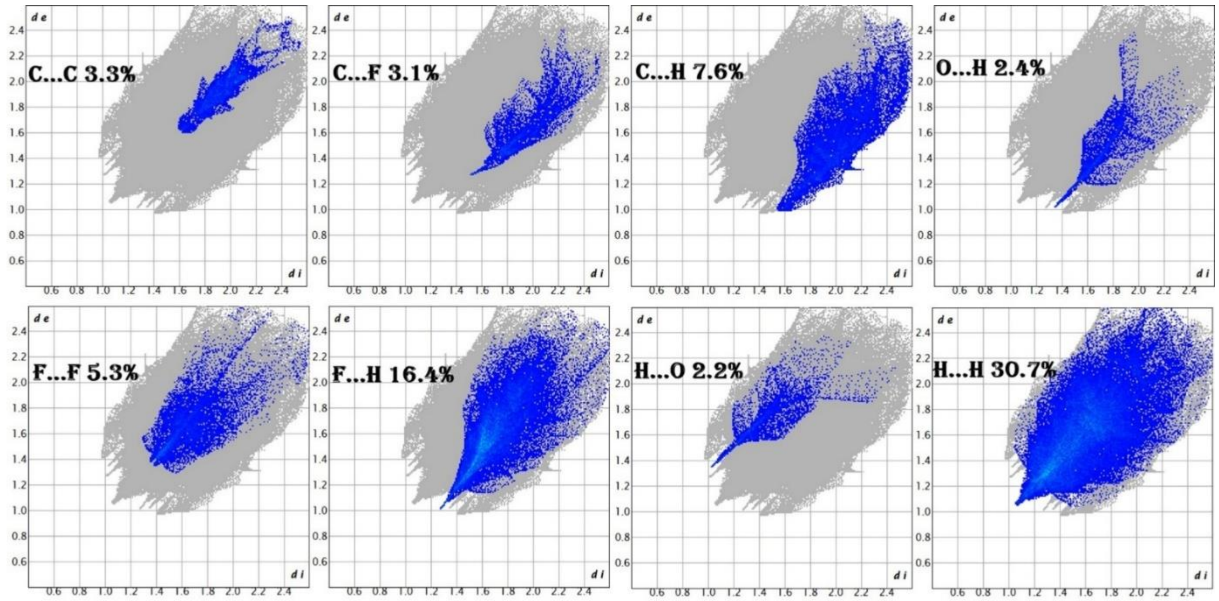


**Figure S28** Fingerprint plots of **1** with different interactions are highlighted in colour.

**Table S3** Contributions of various interactions in percentage to Hirshfeld surface area in **1.**

| **Relative interactions** | **Present in 1.** |
|---|---|
| **C···C** | **3.3 %** |
| **C···F** | **3.1 %** |
| **C···H** | **7.6 %** |
| **O···H** | **2.4 %** |
| **F···F** | **5.3 %** |
| **F···H** | **16.4 %** |
| **H···O** | **2.2 %** |
| **H···H** | **30.7 %** |

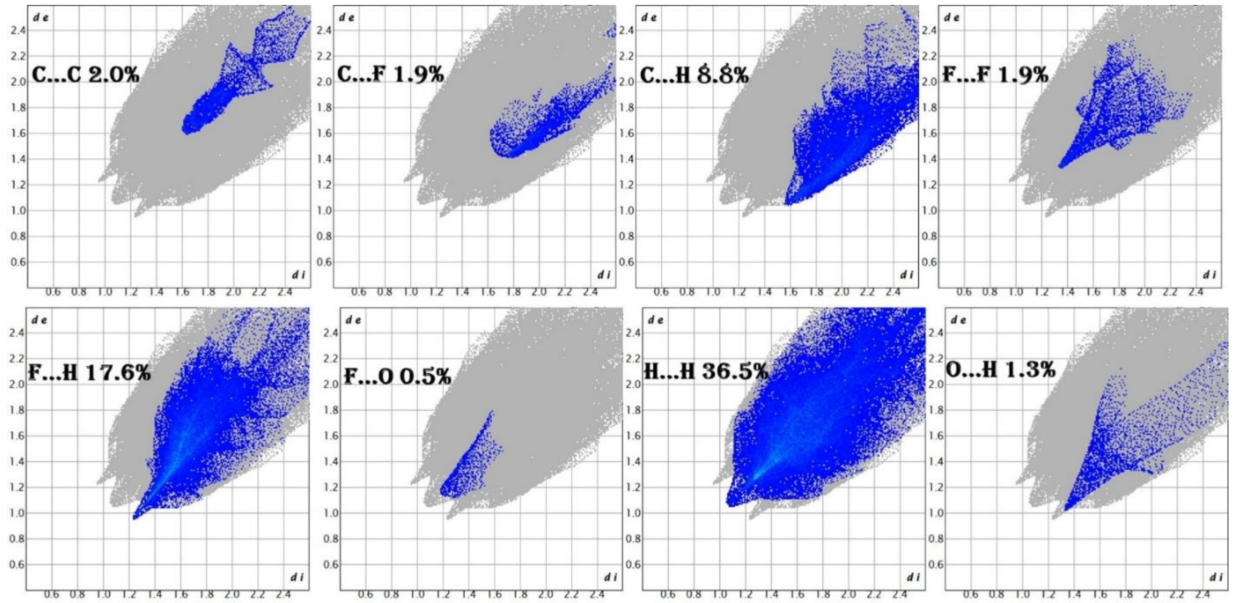


**Figure S29** Fingerprint plots of **3** with different interactions are highlighted in colour.

**Table S4** Contributions of various interactions in percentage to Hirshfeld surface area in **3.**

| **Relative interactions** | **Present in 3.** |
|---|---|
| **C···C** | **2.0 %** |
| **H···H** | **36.5 %** |
| **C···H** | **8.8 %** |
| **O···H** | **1.3 %** |
| **F···F** | **1.9 %** |
| **F···O** | **0.5 %** |
| **C···F** | **1.9 %** |
| **F···H** | **17.6 %** |

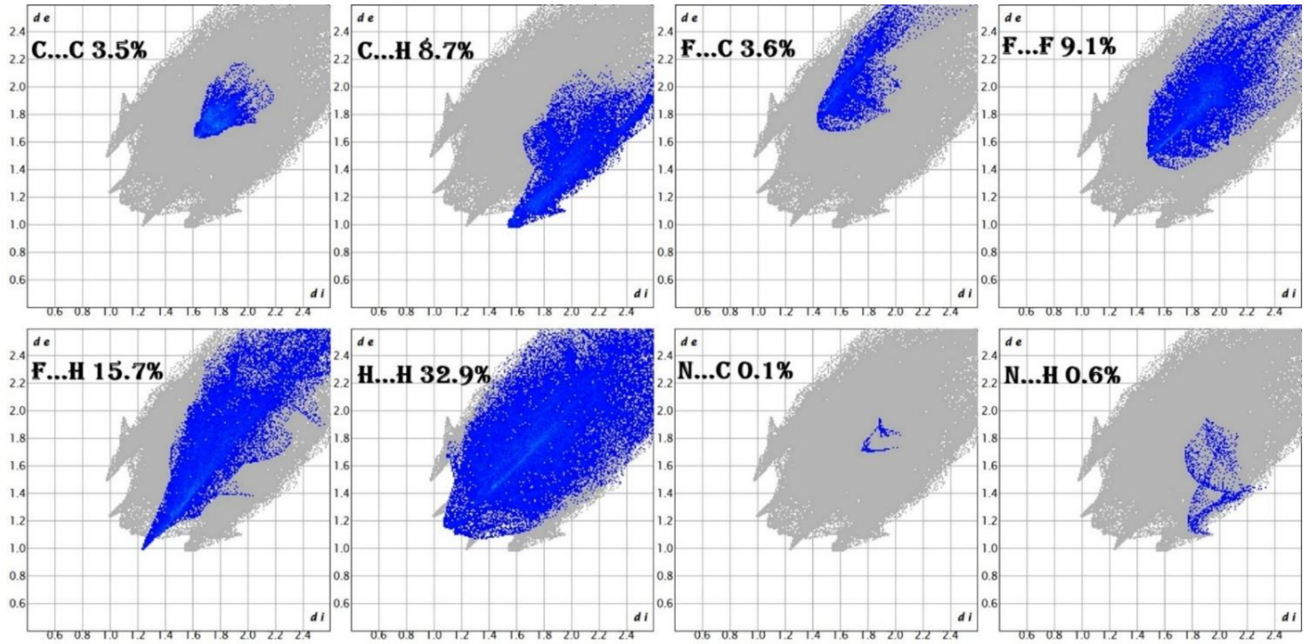


**Figure S30** Fingerprint plots of **4** with different interactions are highlighted in colour.

**Table S5** Contributions of various interactions in percentage to Hirshfeld surface area in **4.**

| **Relative interactions** | **Present in 4.** |
|---|---|
| **C···C** | **3.5 %** |
| **H···H** | **32.9%** |
| **C···H** | **8.7 %** |
| **N···H** | **0.6 %** |
| **F···F** | **9.1 %** |
| **F···C** | **3.6 %** |
| **F···H** | **15.7 %** |
| **N···C** | **0.1 %** |

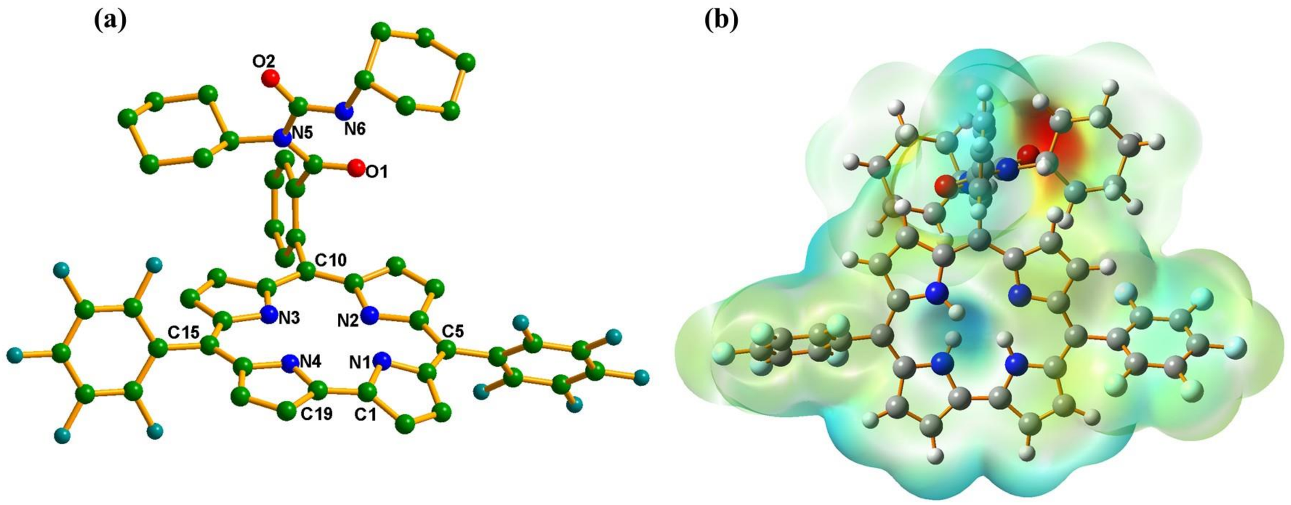


**Figure S31** (a) DFT optimized structure, and (b) Potential mapping of **1.**

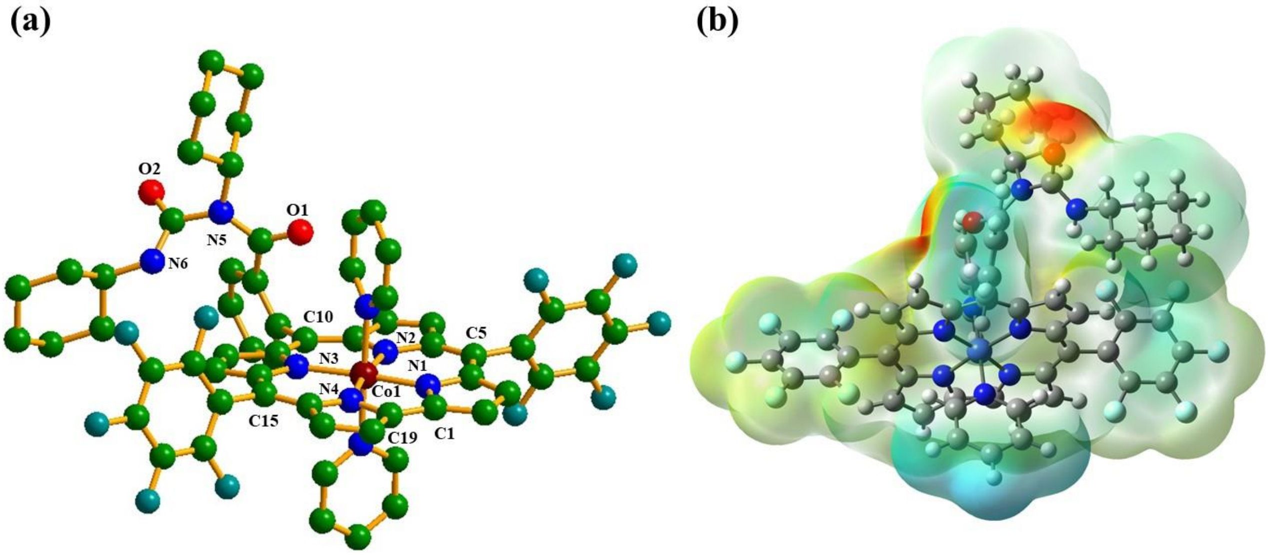


**Figure S32** (a) DFT optimized structure, and (b) Potential mapping of **3.**

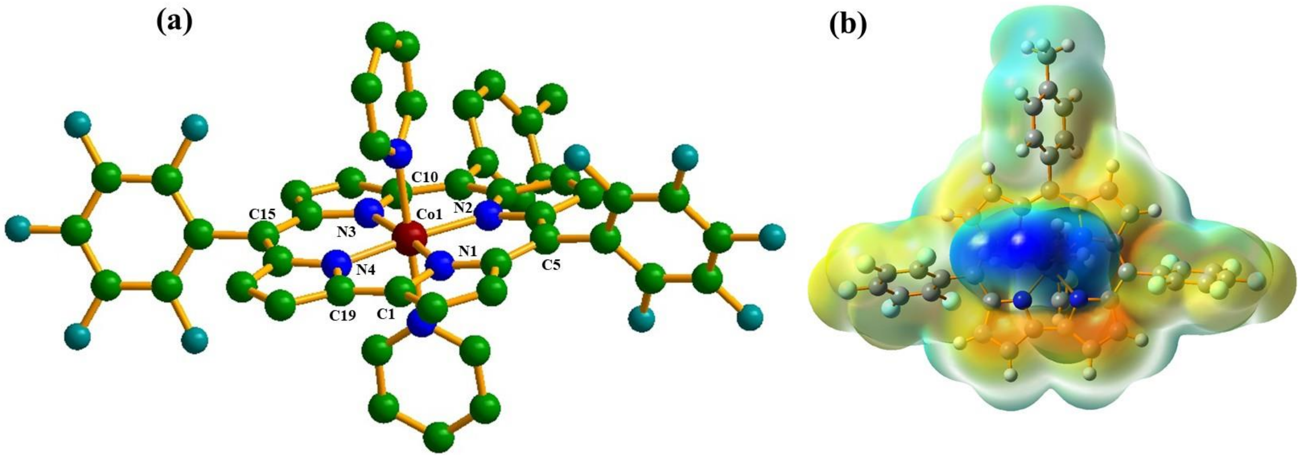


**Figure S33** (a) DFT optimized structure, and (b) Potential mapping of **4.**

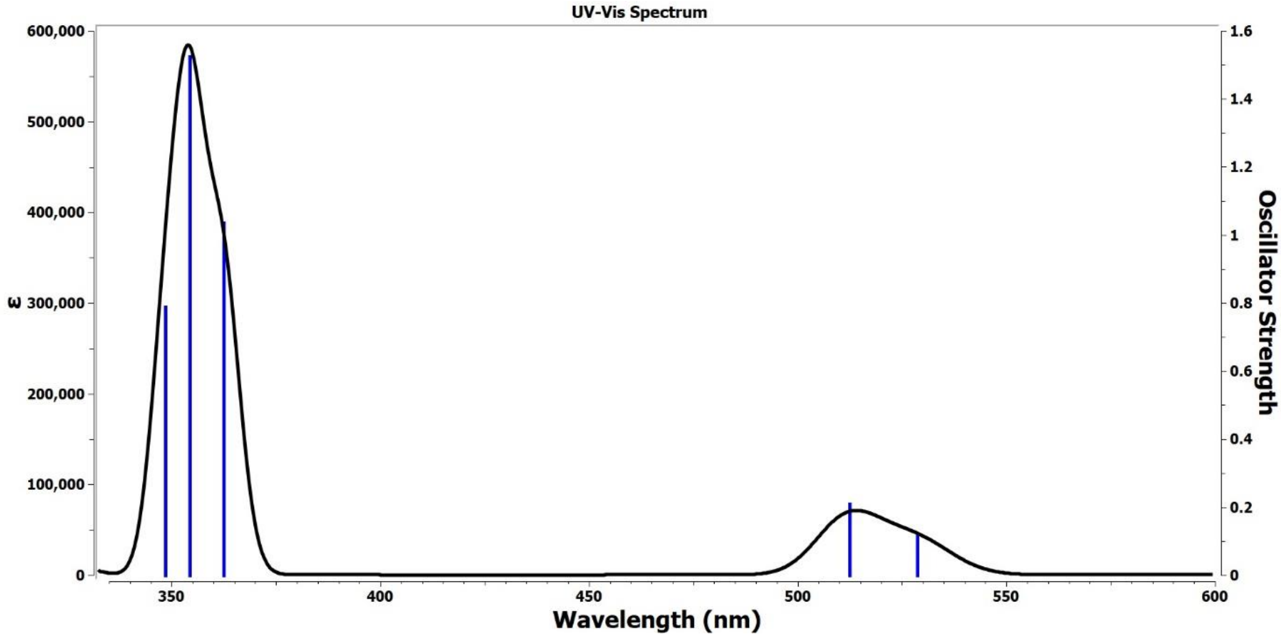


**Figure S34** TD-DFT-based electronic absorption spectra of **1**.

**Table S6** TD-DFT Calculated Electronic Transitions for **1**.

| State | Energy (eV) | Wavelength (nm) | Oscillator Strength | Orbital contributions[a] |
|---|---|---|---|---|
| S1 | 2.34 | 529.27 | 0.119 | HOMO-1->LUMO (35%), HOMO-1-> LUMO+1 (10%), HOMO->LUMO (28%), HOMO-> LUMO+1 (25%) |
| S2 | 2.41 | 513.15 | 0.209 | HOMO-1->LUMO (23%), HOMO-1-> LUMO+1 (16%), HOMO->LUMO (43%) |
| S3 | 3.40 | 364.43 | 1.034 | HOMO-2->LUMO (28%), HOMO-1-> LUMO+1 (35%), HOMO->LUMO (10%) |
| S4 | 3.47 | 356.49 | 1.524 | HOMO-1->LUMO (25%), HOMO-> LUMO+1 (40%) |
| S5 | 3.53 | 350.58 | 0.788 | HOMO-2->LUMO (65%), HOMO -1-> LUMO+1 (19%) |
| S6 | 3.72 | 332.70 | 0.008 | HOMO-3->LUMO (39%), HOMO-> LUMO+2 (49%) |
| S7 | 3.73 | 332.33 | 0.0081 | HOMO-3->LUMO (58%), HOMO-> LUMO+2 (31%) |
| S8 | 3.82 | 323.86 | 0.120 | HOMO-> LUMO+3 (62%), HOMO-> LUMO+4 (22%) |
| S9 | 3.83 | 323.24 | 0.006 | HOMO-4->LUMO (89%) |

| | | | | |
|---|---|---|---|---|
| S10 | 3.86 | 320.65 | 0.194 | HOMO-> LUMO+3 (13%),<br>HOMO-> LUMO+4 (52%) |
| S11 | 3.94 | 313.92 | 0.017 | HOMO-8->LUMO (52%),<br>HOMO-6->LUMO (15%) |
| S12 | 3.96 | 312.93 | 0.016 | HOMO-1-> LUMO+2 (78%) |
| S13 | 3.97 | 312.15 | 0.002 | HOMO-6->LUMO (61%),<br>HOMO -5->LUMO (29%) |
| S14 | 3.99 | 310.40 | 0.004 | HOMO-7->LUMO (77%),<br>HOMO-> LUMO+5 (13%) |
| S15 | 4.00 | 309.76 | 0.081 | HOMO-2-> LUMO+1 (46%),<br>HOMO-1-> LUMO+3 (33%) |
| S16 | 4.02 | 307.97 | 0.012 | HOMO-8->LUMO (12%),<br>HOMO-6->LUMO (13%),<br>HOMO-5->LUMO (44%),<br>HOMO-1-> LUMO+3 (10%) |
| S17 | 4.04 | 306.88 | 0.088 | HOMO-5->LUMO (13%),<br>HOMO-1-> LUMO+3 (28%),<br>HOMO-1-> LUMO+4 (30%) |
| S18 | 4.08 | 303.54 | 0.146 | HOMO-1-> LUMO+4 (40%),<br>HOMO-> LUMO+7 (32%) |
| S19 | 4.10 | 301.94 | 0.018 | HOMO-> LUMO+5 (51%) |
| S20 | 4.11 | 301.23 | 0.071 | HOMO-10->LUMO (14%),<br>HOMO-> LUMO+5 (32%),<br>HOMO-> LUMO+6 (10%) |
| S21 | 4.12 | 300.72 | 0.107 | HOMO -10->LUMO (12%),<br>HOMO->LUMO+6 (18%),<br>HOMO->LUMO+7 (36%) |

| | | | | |
|---|---|---|---|---|
| S22 | 4.15 | 298.46 | 0.126 | HOMO-> LUMO+6 (62%),<br>HOMO-> LUMO+7 (21%) |
| S23 | 4.17 | 296.99 | 0.049 | HOMO-13->LUMO (17%),<br>HOMO-11->LUMO (48%),<br>HOMO-9->LUMO (20%) |
| S24 | 4.21 | 294.04 | 0.031 | HOMO-16->LUMO (11%),<br>HOMO-13->LUMO (14%),<br>HOMO-9->LUMO (65%) |
| S25 | 4.22 | 293.29 | 0.003 | HOMO-4-> LUMO+1 (39%),<br>HOMO-3-> LUMO+1 (57%) |
| S26 | 4.27 | 290.17 | 0.076 | HOMO-16->LUMO (13%),<br>HOMO-14->LUMO (59%),<br>HOMO-10->LUMO (13%) |
| S27 | 4.28 | 289.37 | 0.015 | HOMO-14->LUMO (14%),<br>HOMO-4-> LUMO+1 (34%),<br>HOMO-3-> LUMO+1 (31%) |
| S28 | 4.29 | 288.81 | 0.018 | HOMO-16->LUMO (19%),<br>HOMO-14->LUMO (19%),<br>HOMO-11->LUMO (10%),<br>HOMO-10->LUMO (15%),<br>HOMO-4-> LUMO+1 (19%) |
| S29 | 4.30 | 287.87 | 0.049 | HOMO-12->LUMO (57%) |
| S30 | 4.34 | 285.61 | 0.0014 | HOMO-1-> LUMO+5 (70%),<br>HOMO-1-> LUMO+6 (21%) |

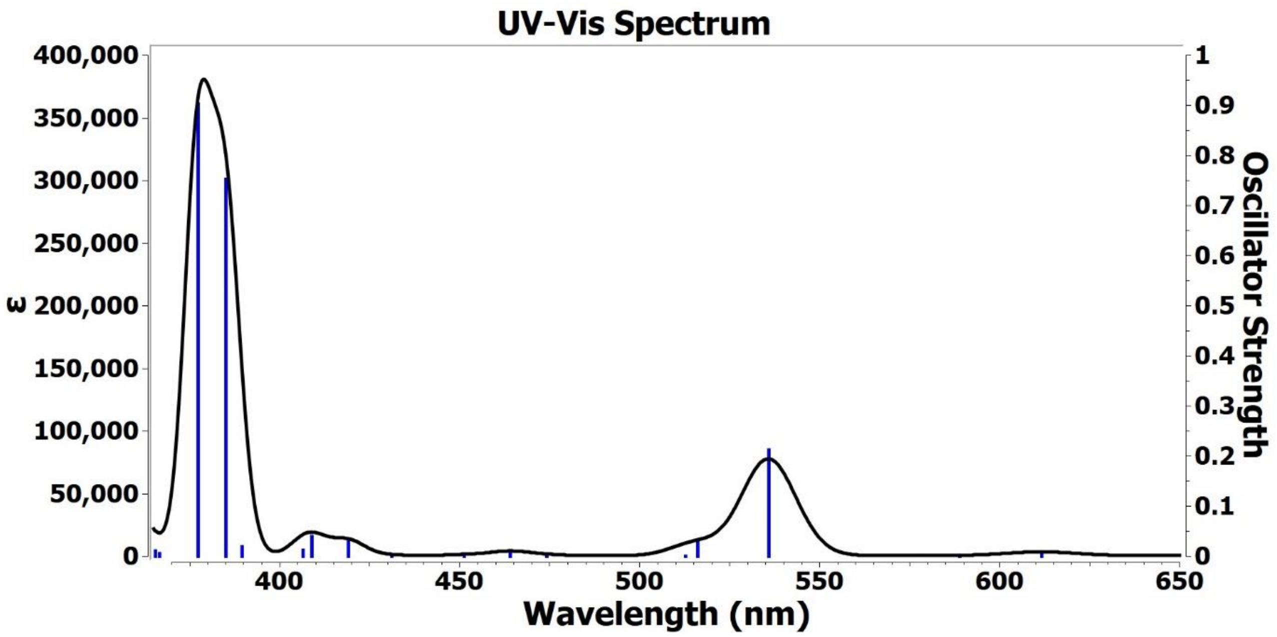


**Figure S35** TD-DFT-based electronic absorption spectra of **3**.

**Table S7** TD-DFT Calculated Electronic Transitions for **3**.

| State | Energy (eV) | Wavelength (nm) | Oscillator Strength | Orbital contributions[a] |
|---|---|---|---|---|
| S1 | 2.02 | 612.26 | 0.0084 | HOMO->LUMO+1 (94%) |
| S2 | 2.10 | 589.91 | 0.0004 | HOMO-2->LUMO+1 (21%), HOMO-1->LUMO+1 (53%) |
| S3 | 2.30 | 537.84 | 0.2124 | HOMO-1->LUMO+2 (19%), HOMO->LUMO (75%) |
| S4 | 2.39 | 518.41 | 0.0291 | HOMO-1->LUMO (54%), HOMO->LUMO+2 (41%) |
| S5 | 2.40 | 515.15 | 0.0005 | HOMO-34->LUMO+1 (12%), HOMO-22->LUMO+1 (11%), HOMO-3->LUMO+1 (46%) |
| S6 | 2.59 | 477.09 | 0.0018 | HOMO-2->LUMO+1 (27%), HOMO-1->LUMO+1 (44%) |
| S7 | 2.65 | 467.12 | 0.0099 | HOMO->LUMO+3 (96%) |
| S8 | 2.65 | 467.12 | 0.0099 | HOMO->LUMO+3 (96%) |
| S9 | 2.85 | 434.86 | 0.0015 | HOMO->LUMO+6 (85%) |
| S10 | 2.72 | 454.63 | 0.0025 | HOMO-> LUMO+5 (32%), HOMO-> LUMO+8 (35%) |
| S11 | 3.00 | 412.96 | 0.0391 | HOMO->LUMO+5 (91%) |

| | | | | |
|---|---|---|---|---|
| S12 | 3.01 | 410.59 | 0.0117 | HOMO-1->LUMO+4 (93%) |
| S13 | 3.14 | 394.08 | 0.019 | HOMO-1->LUMO+6 (81%) |
| S14 | 3.18 | 389.57 | 0.7522 | HOMO-2->LUMO (10%), HOMO-1->LUMO (23%), HOMO->LUMO+2 (34%) |
| S15 | 3.24 | 382.03 | 0.9021 | HOMO-1->LUMO+2 (45%), HOMO->LUMO (10%), HOMO->LUMO+8 (11%) |
| S16 | 3.33 | 371.40 | 0.005 | HOMO->LUMO+6 (11%), HOMO->LUMO+9 (66%) |
| S17 | 3.34 | 370.32 | 0.0114 | HOMO-1->LUMO+5 (85%) |
| S18 | 3.38 | 366.64 | 0.048 | HOMO-2->LUMO (12%), HOMO->LUMO+7 (63%) |
| S19 | 3.41 | 363.17 | 0.066 | HOMO->LUMO+8 (67%) |
| S20 | 3.45 | 358.83 | 0.0027 | HOMO->LUMO+10 (86%) |
| S21 | 3.53 | 350.77 | 0.176 | HOMO-3->LUMO (37%), HOMO-2->LUMO (38%) |
| S22 | 3.55 | 348.89 | 0.1404 | HOMO-3->LUMO (47%), HOMO-2->LUMO (24%) |
| S23 | 3.58 | 346.23 | 0.0188 | HOMO-22->LUMO+6 (19%), HOMO-20->LUMO+6 (26%), HOMO-3->LUMO (11%) |
| S24 | 3.62 | 341.68 | 0.0033 | HOMO-1->LUMO+6 (13%), HOMO-1->LUMO+9 (65%) |
| S25 | 3.64 | 340.12 | 0.0019 | HOMO->LUMO+11 (77%) |

| | | | | |
|---|---|---|---|---|
| S26 | 3.65 | 338.78 | 0.0451 | HOMO-22->LUMO+1 (10%), HOMO-20->LUMO+1 (18%), HOMO-1->LUMO+7 (26%), HOMO-1->LUMO+8 (10%), HOMO->LUMO+13 (15%) |
| S27 | 3.66 | 338.25 | 0.0054 | HOMO-20->LUMO+1 (11%), HOMO->LUMO+11 (12%), HOMO->LUMO+12 (39%), HOMO->LUMO+13 (18%) |
| S28 | 3.67 | 337.55 | 0.0477 | HOMO-22->LUMO+1 (10%), HOMO-20->LUMO+1 (16%), HOMO-1->LUMO+7 (41%) |
| S29 | 3.68 | 336.50 | 0.0183 | HOMO-1->LUMO+8 (39%), HOMO->LUMO+12 (34%), HOMO->LUMO+13 (12%) |
| S30 | 3.71 | 333.75 | 0.1645 | HOMO-1->LUMO+8 (31%), HOMO->LUMO+13 (41%) |

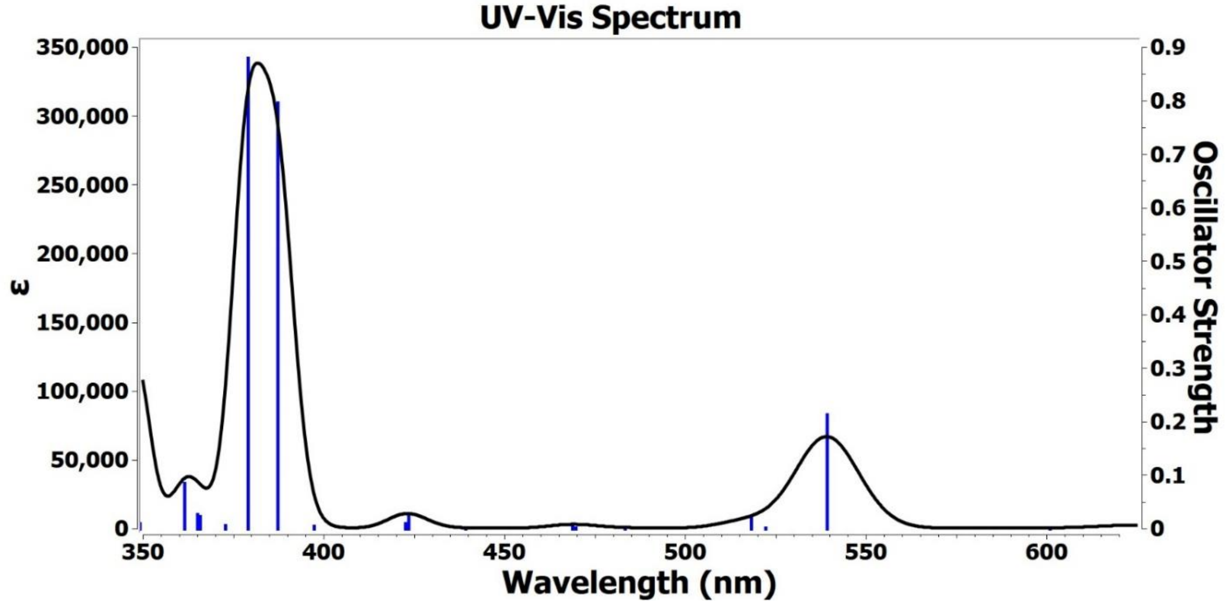


**Figure S36** TD-DFT-based electronic absorption spectra of **4**.

**Table S8** TD-DFT Calculated Electronic Transitions for **4**.

| State | Energy (eV) | Wavelength (nm) | Oscillator Strength | Orbital contributions[a] |
|---|---|---|---|---|
| **S1** | 1.99 | 621.31 | 0.0075 | HOMO->LUMO+1 (97%) |
| **S2** | 2.07 | 596.56 | 0.0002 | HOMO-2->LUMO+1 (21%), HOMO-1->LUMO+1 (56%) |
| **S3** | 2.31 | 535.98 | 0.2132 | HOMO-1->LUMO+2 (20%), HOMO->LUMO (75%) |
| **S4** | 2.38 | 519.17 | 0.0007 | HOMO-24->LUMO+1 (13%), HOMO-18->LUMO+1 (10%), HOMO-3->LUMO+1 (42%) |
| **S5** | 2.40 | 515.39 | 0.0194 | HOMO-1->LUMO (54%), HOMO->LUMO+2 (42%) |
| **S6** | 2.57 | 481.05 | 0.0009 | HOMO-2->LUMO+1 (29%), HOMO-1->LUMO+1 (43%) |
| **S7** | 2.65 | 467.63 | 0.0017 | HOMO->LUMO+3 (98%) |
| **S8** | 2.65 | 466.85 | 0.0074 | HOMO->LUMO+4 (95%) |
| **S9** | 2.83 | 437.62 | 0.0005 | HOMO->LUMO+5 (87%) |

| | | | | |
|---|---|---|---|---|
| **S10** | 2.93 | 422.18 | 0.0244 | HOMO-1->LUMO+4 (85%) |
| **S11** | 2.94 | 421.38 | 0.0101 | HOMO-1->LUMO+3 (93%) |
| **S12** | 3.12 | 396.48 | 0.005 | HOMO-1->LOMO+5 (83%) |
| **S13** | 3.20 | 386.61 | 0.7959 | HOMO-2->LUMO (13%), HOMO-1->LUMO (25%), HOMO->LUMO+2 (37%) |
| **S14** | 3.27 | 378.47 | 0.8792 | HOMO-1->LUMO+2 (47%), HOMO->LUMO (10%), HOMO->LUMO+7 (17%) |
| **S15** | 3.32 | 372.44 | 0.0063 | HOMO->LUMO+5 (12%), HOMO->LUMO+8 (47%), HOMO->LUMO+9 (34%) |
| **S16** | 3.39 | 365.54 | 0.0224 | HOMO->LUMO+6 (24%), HOMO->LUMO+8 (38%), HOMO->LUMO+9 (29%) |
| **S17** | 3.39 | 364.77 | 0.0263 | HOMO-2->LUMO (11%), HOMO->LUMO+6 (44%), HOMO->LUMO+7 (18%), HOMO->LUMO+9 (23%) |
| **S18** | 3.43 | 361.23 | 0.0854 | HOMO-1->LUMO+2 (10%), HOMO->LUMO+7 (54%) |
| **S19** | 3.55 | 349.19 | 0.0101 | HOMO-3->LUMO (82%) |
| **S20** | 3.56 | 348.24 | 0.3588 | HOMO-2->LUMO (62%) |
| **S21** | 3.58 | 345.96 | 0.0216 | HOMO-18->LUMO+5 (24%), HOMO-16->LUMO+5 (24%) |
| **S22** | 3.62 | 342.46 | 0.0029 | HOMO-1->LUMO+5 (13%), HOMO-1->LUMO+8 (44%), HOMO-1->LUMO+9 (33%) |

| | | | | |
|---|---|---|---|---|
| **S23** | 3.64 | 339.82 | 0.0006 | HOMO-18->LUMO+1 (31%),<br>HOMO-16->LUMO+1 (42%) |
| **S24** | 3.67 | 337.20 | 0.0188 | HOMO-1->LUMO+6 (35%),<br>HOMO-1->LUMO+7 (15%),<br>HOMO-1->LUMO+8 (11%),<br>HOMO->LUMO+11 (24%) |
| **S25** | 3.68 | 336.68 | 0.0085 | HOMO->LUMO+10 (89%) |
| **S26** | 3.69 | 335.77 | 0.027 | HOMO-1->LUMO+8 (21%),<br>HOMO-1->LUMO+9 (22%),<br>HOMO->LUMO+11 (49%) |
| **S27** | 3.69 | 335.65 | 0.0268 | HOMO-1->LUMO+6 (46%),<br>HOMO-1->LUMO+7 (23%),<br>HOMO-1->LUMO+9 (20%) |
| **S28** | 3.71 | 333.60 | 0.2102 | HOMO-1->LUMO+7 (48%),<br>HOMO-1->LUMO+8 (13%),<br>HOMO-1->LUMO+9 (11%),<br>HOMO->LUMO+11 (15%) |
| **S29** | 3.82 | 324.32 | 0.0004 | HOMO-5->LUMO+1 (41%),<br>HOMO-3->LUMO+5 (13%) |
| **S30** | 3.84 | 322.68 | 0.0193 | HOMO-5->LUMO (84%) |

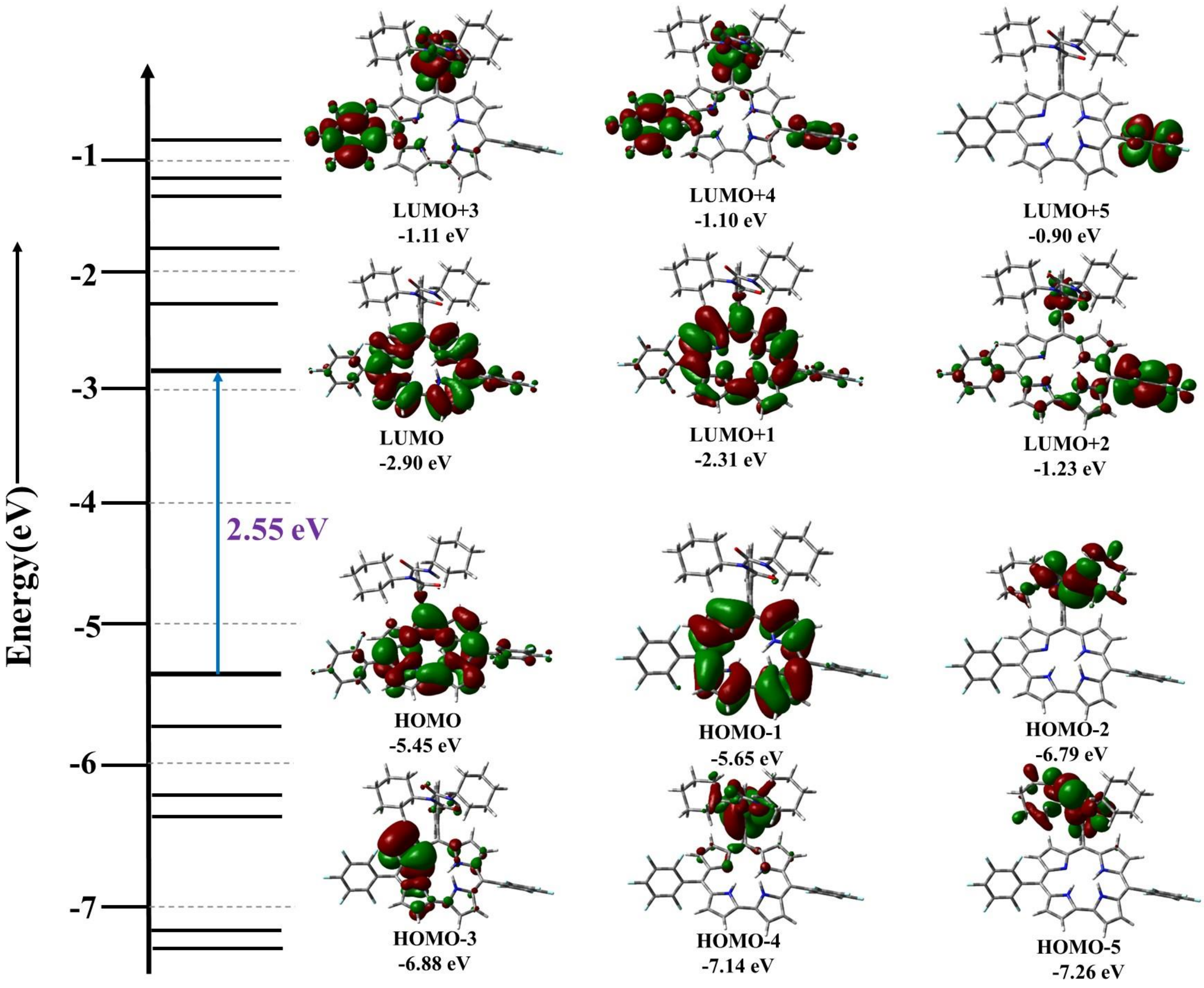


**Figure S37** Composition and Energies of Selected Kohn-Sham orbital energy level diagram and Molecular Orbitals of FB corrole, **1** (iso-value of 0.02).

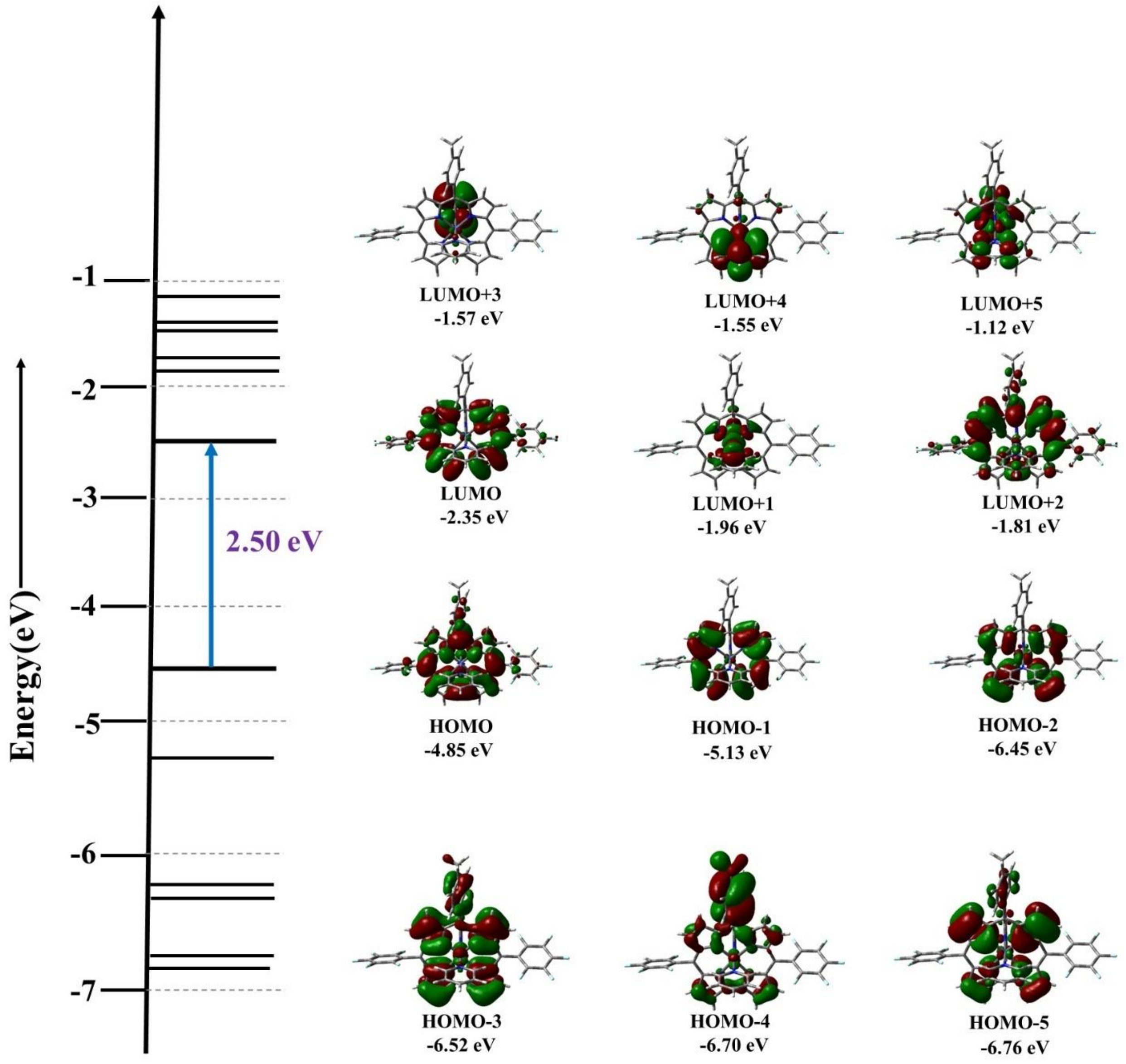


**Figure S38** Selected Kohn-Sham orbital energy level diagram of corrolato coblat(III) complex, **4** (iso-value of 0.02).

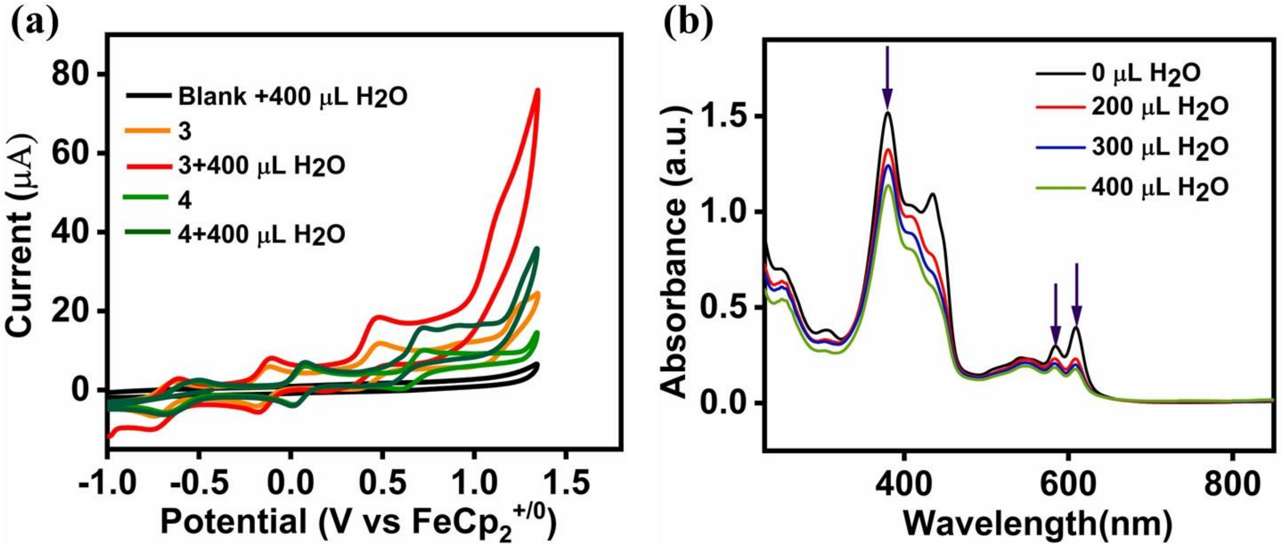


**Figure S39** (a) Comparative CV of compound **3** (light green trace) and with 400 μL of water (deep green trace), compound **4** (orange trace) with 400 μL of water (red trace). (b) UV-Vis spectrum of compound **3** with different water concentrations.

**Experimental Protocol for Rinse Test**:

To evaluate the homogeneous nature of compound **3** in catalytic water oxidation, a rinse test was performed in anhydrous MeCN in presence of 400μL $H_2O$ over six consecutive electrochemical runs. In the first experiment, a complete cyclic voltammetric scan of the complex exhibited pronounced catalytic activity for water oxidation (purple trace, run 1, as 1$^{st}$ cycle, Figure S40). A gradual decrease in catalytic current density was observed upon successive cycling. After completion of this six consecutive cycles, without further polishing, the electrode was then rinsed solely with water without polishing in alumina and placed in a fresh MeCN solution devoid of any metal catalyst. The third scan, displayed almost no water oxidation activity (dark purple trace, run 3, Rinse, Figure S40). The absence of appreciable catalytic current in this final run strongly supports that the water oxidation proceeds via a homogeneous pathway, with no significant contribution from electrode-bound species.

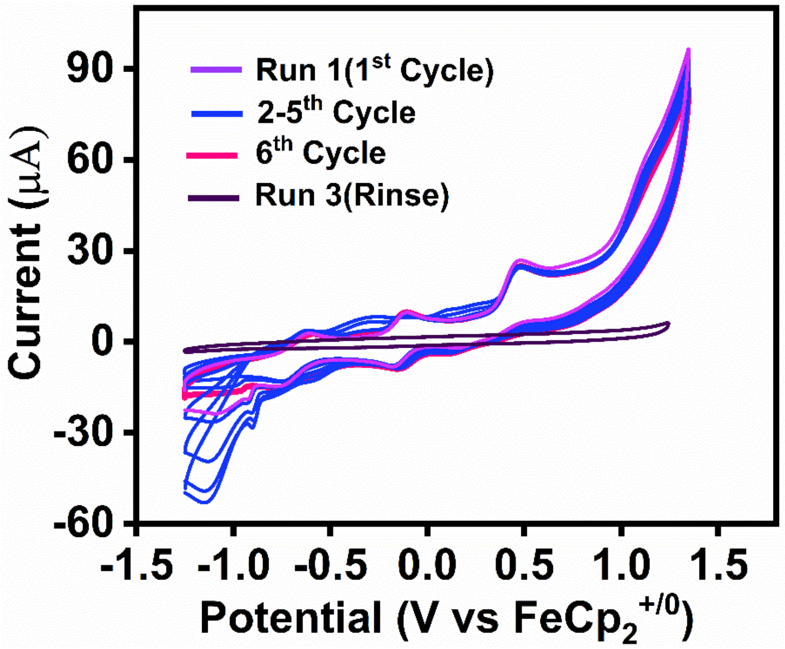


**Figure S40** Rinse test for the compound **3** in dry MeCN. Conditions: 0.5 mM complex in a 0.1 M $TBAPF_6$ solution in MeCN, 50 mV/s scan rate. The data was recorded with a Glassy carbon working electrode, a Pt wire counter electrode, and Ag/AgCl (in 3M KCl) reference electrode in the presence of 0.1 M $TBAPF_6$.

**Faradaic efficiency calculation:**

**For Compound 3:**

The amount of gaseous sample injected in GC =100 μL

The area of $O_2$ Peak for standard gas mixture is 390025 units which corresponds to 1.023mol% of oxygen (This calibration was used for mol % conversion.)

Hence, area 390025 unit = 1.023 mol% of Oxygen

The area of $O_2$ Peak after CPE= 23420

Area 23420 unit = 0.06154 mol% oxygen

The area of $O_2$ peak before CPE=16364

Area 16364 unit= 0.043 mol% oxygen

Hence net mol% oxygen produced during 1hour CPE= (0.06154-0.043) = 0.01854 mol%

$$x_{\mathrm{O_2,net}} = 0.06154\% - 0.043\% = 0.01854\%$$

Charge accumulation during CPE= $6.9619\times10^{-2}$ Columb

Now,

1) **Total moles of gas in Headspace**

   The total amount of gas in the 20 mL headspace (1 atm, 298 K) was determined from the ideal gas law.

$$n_{\mathrm{total}} = \frac{PV}{RT} = 8.18 \times 10^{-4}\ \mathrm{mol}$$

2) **Total moles of Oxygen gas in Headspace**

   Thus, the amount of $O_2$ formed during CPE is

$$n_{\mathrm{O_2}} = n_{\mathrm{total}} \times \frac{0.01854}{100} = 1.52 \times 10^{-7}\ \mathrm{mol}$$

3) **Charge Equivalent to that Oxygen**

   The charge associated with $O_2$ formation (4 $e^-$ per $O_2$) is given by:

$$Q_{\mathrm{O_2,experimental}} = 4Fn_{\mathrm{O_2}} = 1.52 \times 10^{-7} \times 4 \times 96485.33212 = \ 0.05847\ \mathrm{C}$$

4) **Faradic Efficency**

The Faradaic efficiency is therefore:

$FE = \frac{Q_{O_2,\text{experimental}}}{Q_{O_2,\text{theoritical}}} \times 100 =$ 0.05847/0.069619 × 100%

= 83.95 %

**For Compound 4:**

The amount of gaseous sample injected in GC =100 μL

The area of $O_2$ Peak for standard gas mixture is 390025 units which corresponds to 1.023mol% of oxygen

Hence, area 390025 unit = 1.023 mol% of Oxygen

The area of $O_2$ Peak after CPE= 50796

Area 50796 unit = 0.1332 mol% oxygen

The area of $O_2$ peak before CPE=44885

Area 44885 unit= 0.1177 mol% oxygen

Hence net mol% oxygen produced during 1hour CPE= (0.1332-0.1177) = 0.015 mol%

$$x_{O_2,\text{net}} = 0.1332\% - 0.1177\% = 0.015\%$$

Charge accumulation during CPE= $7.940\times10^{-2}$ Columb

Now,

1) **Total moles of gas in Headspace**

The total amount of gas in the 20 mL headspace (1 atm, 298 K) was determined from the ideal gas law.

$$n_{\text{total}} = \frac{PV}{RT} = 8.18 \times 10^{-4} \text{ mol}$$

2) **Total moles of Oxygen gas in Headspace**

Thus, the amount of $O_2$ formed during CPE is

$n_{O_2} = n_{\text{total}} \times \frac{0.015}{100} = 1.23 \times 10^{-7}$ mol.

**3) Charge Equivalent to that Oxygen**

The charge associated with $O_2$ formation (4 $e^-$ per $O_2$) is given by:

$$Q_{O_2,\text{experimental}} = 4Fn_{O_2} = 1.23 \times 10^{-7} \times 4 \times 96485.33212 = 0.04735\ \text{C}$$

**4) Faradic Efficency**

The Faradaic efficiency is therefore:

$$\text{FE} = \frac{Q_{O_2,\text{experimental}}}{Q_{O_2,\text{theoritical}}} \times 100 = 0.04735/0.07940 \times 100\%$$

$$= 59.63\ \%$$

**TOF($K_{obs}$) Calculation**

The kinetic constant $k_{obs}$ (TOF) for catalytic water oxidation was calculated using the following relations.

$$i_c = n_c FAC^0_{cat}\sqrt{Dk_{obs}}$$

$$i_p = 0.4463 n_p FAC^0_{cat}\sqrt{\frac{n_p F\upsilon D}{\text{RT}}}$$

$$\frac{i_c}{i_p} = \frac{n_c}{0.4463} \times \sqrt{(\frac{RT}{F\upsilon})k_{obs}}$$

$$k_{obs} = 0.02424 \times (\frac{i_c}{i_p})^2$$

In this framework, **Ip** corresponds to the peak current of the reversible, non-catalytic redox transition of the catalyst, while **Ic** denotes the current associated with the catalytic process. The catalytic pathway involves **nc = 4** electron transfers, whereas the reversible redox couple of the catalyst involves **np = 1** electron. Since **np** has already been substituted into the expression, the equation is simplified accordingly. The other parameters include the potential scan rate (**υ**, in $V \cdot s^{-1}$), the diffusion coefficient of the catalyst species (**D**), Faraday's constant (**F**), the bulk concentration of the catalyst (**$C^{o}_{cat}$**), and the effective electroactive surface area of the electrode (**A**). The relation is applicable in the case of a water nucleophilic attack (WNA) mechanism for oxygen evolution catalysis. **$k_{obs}$**, also referred to as the turnover frequency, **TOF** was determined by calculating the ratio of the catalytic current (**Ic**) observed at the third oxidation wave to the peak current (**Ip**) corresponding to the first oxidation process.

Now,

nc= 4

R= 8.314 $Jmol^{-1}K^{-1}$

T=298K

F= 96485 Columb/mol

υ= 0.05 V/s

**<u>For Co-Complex 3.</u>**

$i_c$ = 45.68 μA

$i_p$ = 6.51 μA

$$k_{obs} = 0.02424 \times (\frac{45.68}{6.51})^2$$

$k_{obs}$ = $(TOF)_{max}$ = 1.19 $S^{-1}$ @ 1.12 V (vs. $FeCp_2^{+/0}$)

**<u>For Co-Complex 4.</u>**

$i_c$ = 27.96 μA

$i_p$ = 6.37 μA

$$k_{obs} = 0.02424 \times (\frac{27.96}{6.37})^2$$

$k_{obs}$ = $(TOF)_{max}$ = 0.47 $S^{-1}$ @ 1.21 V (vs. $FeCp_2^{+/0}$)

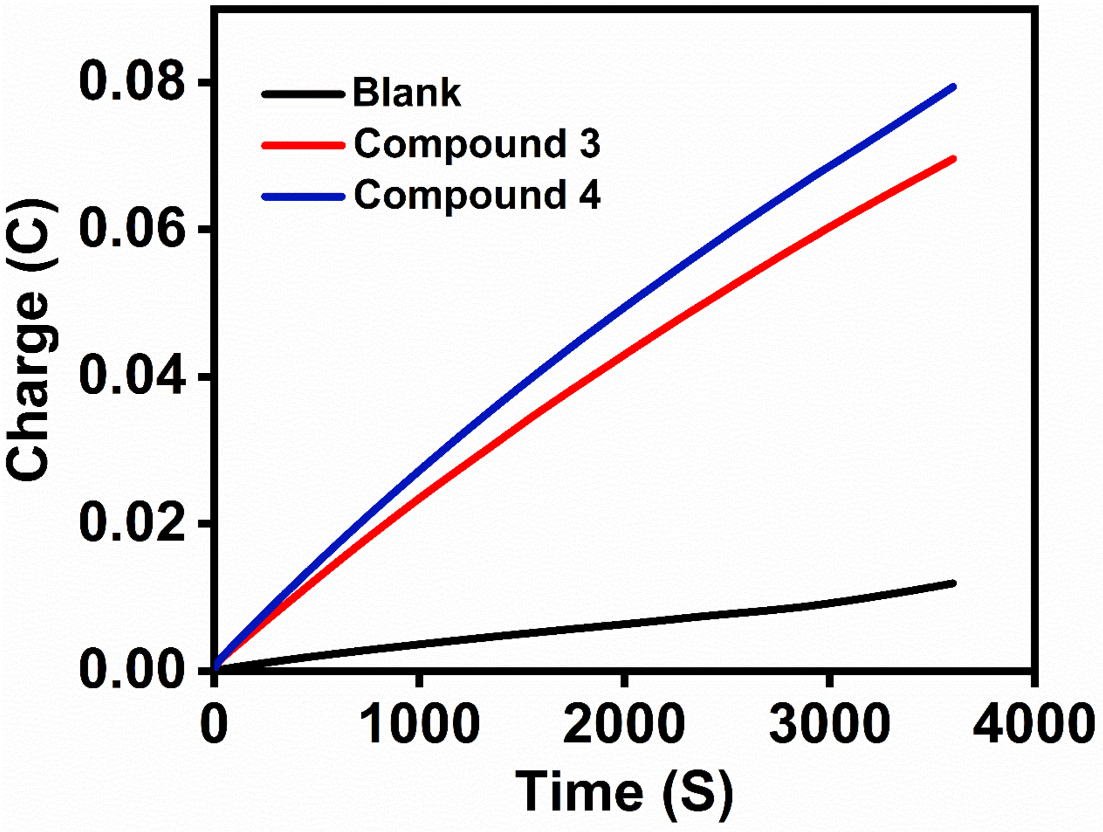


**Figure S41** Bulk electrolysis (or chronocoulometry) experiment for compound **3**(Red line) and compound **4** (blue line) with blank (Blank line) was performed by holding the constant potential 1.78V (vs. Ag/AgCl couple) in an Ar atmosphere. The data were recorded with a 0.5 cm × 0.5 cm carbon working electrode, Pt wire counter electrode, and Ag/AgCl (3M KCl) reference electrode in the presence of 0.1 M $TBAPF_6$ electrolyte.

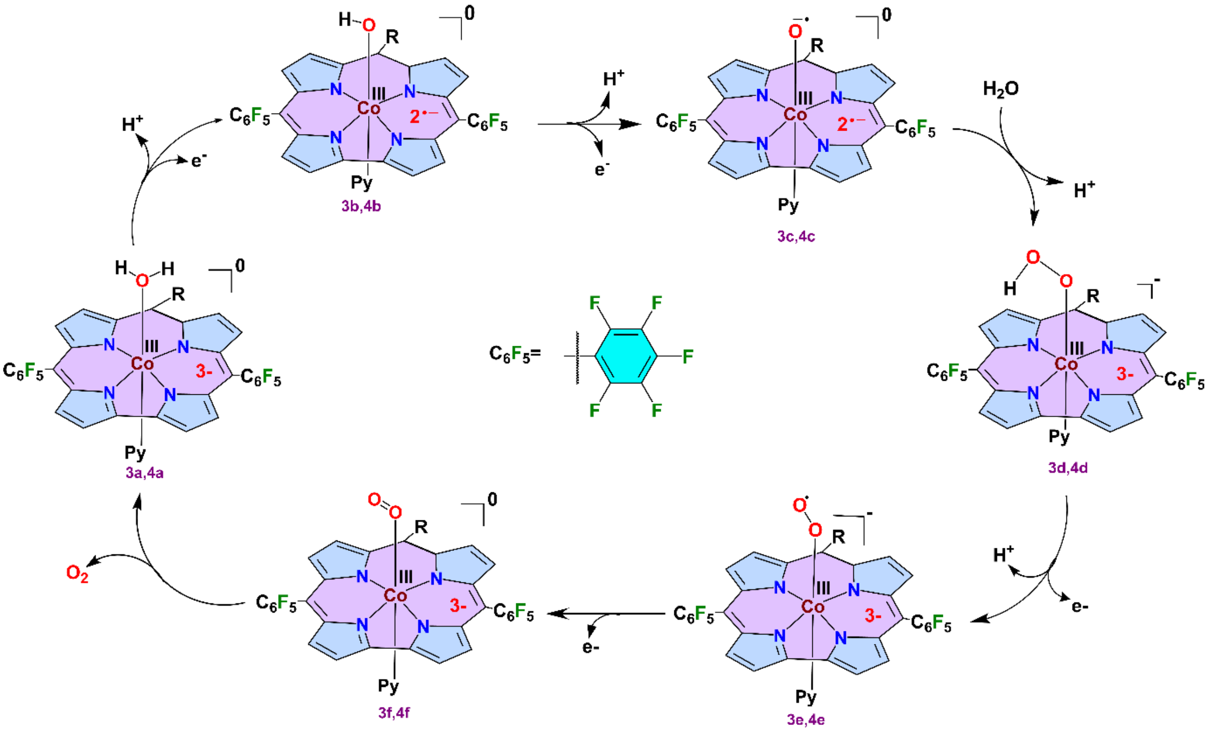


**Figure S42** Proposed catalytic cycle for water oxidation via the water nucleophilic attack (WNA) pathway in Co (III) corroles bearing a single apical pyridine (Py) ligand. The mono-pyridine species is generated under catalytic conditions by ligand exchange from the bis-pyridine Co (III) precursor. In this pathway, the R group denotes N-benzoyl-DCU (benzoyl-urea) derivative for **3a-3f** and 4-methylphenyl derivative for **4a-4f**.

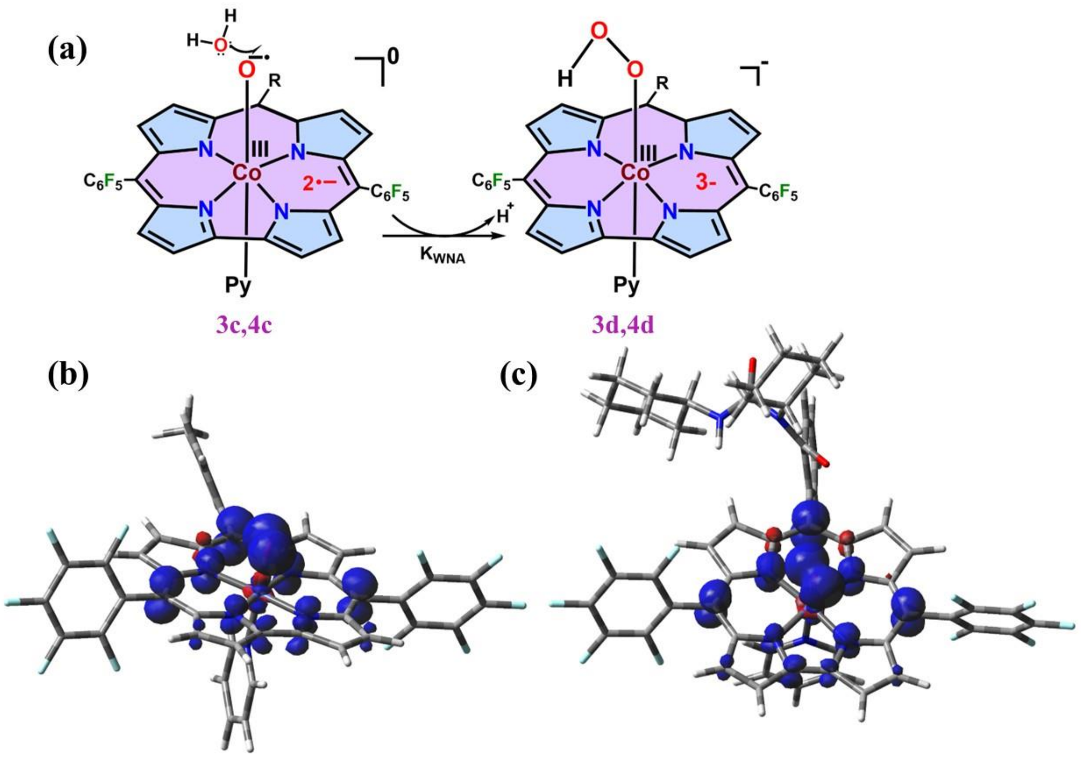


**Figure S43** (a) O-O bond formation step *via* water nucleophilic attack (WNA) on the doubly oxidized cobalt corrole complex. Spin density plots (iso-value = 0.005) of $[Co^{III}(corrole^{\bullet 2-})(O^{\bullet -})Py]^0$ for (b) intermediate **4c** and (c) intermediate **3c**.

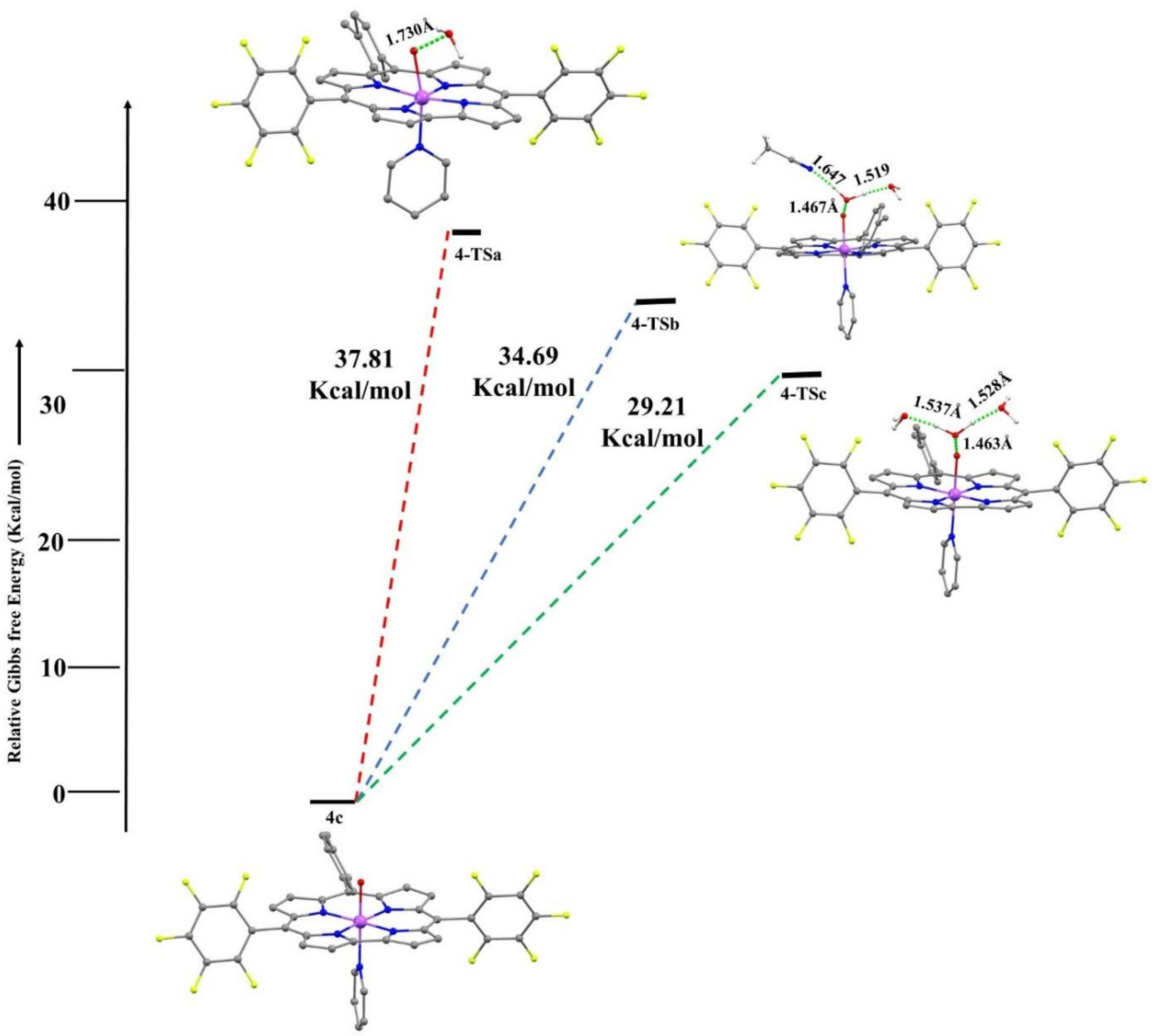


**Figure S44** Free-energy profile for the WNA step showing transition-state stabilization arising from hydrogen-bonding interactions between the incoming water nucleophile and two additional water molecules.

**Detailed theoretical calculations for cobalt complex 4 used to construct the energy profile diagram.**

| Intermediate | Electronic Energy **(H)** | Thermal correction to Gibbs Free Energy **(H)** | Thermal correction to Gibbs Free Energy **(Kcal/mol)** | Sum of electronic and thermal free energies **(H)** | Sum of electronic and thermal free energies **(Kcal/mol)** |
|---|---|---|---|---|---|
| **4a** | -3145.078 | 0.473577 | 297.174118 | -3144.6044 | -1973269.4745 |
| **4b** | -3144.4402 | 0.460234 | 288.801257 | -3143.98 | -1972877.66 |
| **4c** | -3143.7795 | 0.45263 | 284.02967 | -3143.3161 | -1972461.0538 |
| **4d** | -3219.7544 | 0.462102 | 289.973445 | -3219.2925 | -2020136.9748 |
| **4e** | -3219.1362 | 0.449572 | 282.11075 | -3218.6867 | -2019756.8295 |
| **4f** | -3218.9463 | 0.452188 | 283.752315 | -3218.4941 | -2019635.9711 |
| **$H_2O$** | -76.45447 | 0.003618 | 2.27032976 | -76.450852 | -47973.644172 |
| **4-TSc** | -3373.1392 | 0.517118 | 324.496513 | -3372.6221 | -2116352.772 |
| **$H^+$** | Reference: *J. Am. Chem. Soc.* **2018**, *140*, 1557−1565 | | | | -272.2 |
| **Ferrocenium ($F_c^+$) (In ACN)** | -510.30688 | 0.122904 | 77.1234409 | -510.18398 | -320145.3491 |
| **Ferrocene (Fc)(In ACN)** | -510.45303 | 0.122946 | 77.1497963 | -510.33009 | -320237.0347 |
| **$F_c^+$-$F_c$ (In ACN)** | | | | | 91.69 |

**1) 4a** -> **4b** + $H^+$+ $e^-$

$$\boldsymbol{\Delta Ga - b} = [(\boldsymbol{\Delta Gb} + \boldsymbol{\Delta GH^+} + \boldsymbol{\Delta Gelectron}) - \boldsymbol{\Delta Ga}]$$

**= [(-1972877.66-272.2-142.65) +1973269.4745**

**= -23.04 Kcal/mol**

**2) 4b** -> **4c** + $H^+$+ $e^-$

$$\boldsymbol{\Delta Gb - c} = [(\boldsymbol{\Delta Gc} + \boldsymbol{\Delta GH^+} + \boldsymbol{\Delta Gelectron}) - \boldsymbol{\Delta Gb}]$$

**= [(-1972461.0538-272.2-142.65) +1972877.66]**

**= 1.76 Kcal/mol**

**3) 4d** -> **4e** + $H^+$+ $e^-$

$$\boldsymbol{\Delta Gd - e} = [(\boldsymbol{\Delta Ge} + \boldsymbol{\Delta GH^+} + \boldsymbol{\Delta Gelectron}) - \boldsymbol{\Delta Gd}]$$

**= [(-2019756.8295-272.2-142.65) +2020136.9748]**

**= -34.70 Kcal/mol**

**4) 4e** -> **4f** + $e^-$

$$\boldsymbol{\Delta Ge - f} = [(\boldsymbol{\Delta Gf} + \boldsymbol{\Delta Gelectron}) - \boldsymbol{\Delta Ge}]$$

**= [(-2019635.9711-142.65) +2019756.8295]**

**= -21.79 Kcal/mol**

**<u>Including TS</u>**

**1) 4c** + $3H_2O$ -> **4-TSc**

$$\boldsymbol{\Delta Gc - TS} = [\boldsymbol{\Delta GTS} - (\boldsymbol{\Delta Gc} + (\boldsymbol{\Delta GH2O} \times 3)]$$

**= [(-2116352.772) - ( -1972461.0538+(-47973.644172×3))]**

**= 29.21 Kcal/mol**

**2) 4-TSc -> 4d** + $\mathbf{H^+}$+ $2H_2O$

$$\Delta GTS - d = [(\Delta Gd + \Delta GH^+ + (\Delta GH2O \times 2)) - \Delta GTS]$$

**= [(-2020136.9748-272.2+ (-47973.644172×2) +2116352.772]**

**= -3.69 Kcal/mol**

In these calculations, the free energy of the electron $\Delta G_{electron}$ was taken as -142.65 kcalmol$^{-1}$. The final electron-release step corresponds to the formation of the Co-$O_2$ adduct, followed by $O_2$ evolution and regeneration of the catalyst. The potential associated with this step can therefore be considered representative of the water-oxidation process. To estimate this potential theoretically, the corresponding free-energy change was evaluated as described below.

$[GFc^+ - GFc]$ **= (320237.0347-320145.3491) = 91.69 Kcal/mol**

$\Delta Gredox = \Delta G4f - \Delta G4e$ **= [(-2019635.9711) -(-2019756.8295)] = 120.86 Kcal/mol**

$\Delta Gabsolute, redox = Gredox - GFc/Fc^+$ **= 120.86-91.69= 29.17 Kcal/mol**

$$\Delta G^{o} = -nFE^{o}$$

$E^{o} = -\Delta G^{o}/F$ **[F=23.06 Kcal/mol/V]**

**= 1.26V** (Vs $FeCp_2$) (taking only the magnitude )

**=1.90V** (Vs NHE) **[$Evs\ NHE = Evs\ FeCp2 + 0.64$]**

Accordingly, the theoretically estimated potential is 1.26 V vs $FeCp_2$(taking only the magnitude as calculated value is reported as an oxidation potential). The free energy of the electron (-142.65 kcal mol$^{-1}$), obtained from theoretical calculations, was used consistently to convert computed free-energy changes into electrode potentials. Potentials referenced to the $FeCp_2$/$FeCp_{2+}$ couple were subsequently converted to the NHE scale using the established literature offset.

**Detailed theoretical calculations for cobalt complex 3 used to construct the energy profile diagram.**

| Intermediate | Electronic Energy **(H)** | Thermal correction to Gibbs Free Energy **(H)** | Thermal correction to Gibbs Free Energy **(Kcal/mol)** | Sum of electronic and thermal free energies **(H)** | Sum of electronic and thermal free energies **(Kcal/mol)** |
|---|---|---|---|---|---|
| **3a** | -3912.725 | 0.782274 | 490.884451 | -3911.9427 | -2454781.6303 |
| **3b** | -3912.0327 | 0.764652 | 479.826477 | -3911.268 | -2454358.25 |
| **3c** | -3911.407685 | 0.755894 | 474.330784 | -3910.65179 | -2453971.5782 |
| **3d** | -3987.3964 | 0.768103 | 481.992012 | -3986.6283 | -2501647.5619 |
| **3e** | -3986.7787 | 0.754902 | 473.708258 | -3986.0238 | -2501268.2323 |
| **3f** | -3986.5851 | 0.757931 | 475.608985 | -3985.8271 | -2501144.8012 |
| **$H_2O$** | -76.45447 | 0.003618 | 2.27032976 | -76.450852 | -47973.644172 |
| **3TS** | -3987.8279 | 0.783024 | 491.355083 | -3987.0449 | -2501908.9824 |
| **$H^+$** | Reference: *J. Am. Chem. Soc.* **2018**, *140*, 1557−1565 | | | | -272.2 |

**Calculation of ΔG :**

**1) 3a -> 3b** + $H^+$+ $e^-$

$$\Delta Ga - b = [(\Delta Gb + \Delta GH^+ + \Delta Gelectron) - \Delta Ga]$$

**= [(-2454358.25-272.2 -145.00) + 2454781.6303]**

**= 6.18 Kcal/mol.**

**2) 3b -> 3c** + H$^+$+ e$^-$

$$\Delta Gb - c = [(\Delta Gc + \Delta GH^+ + \Delta Gelectron) - \Delta Gb]$$

**= [(-2453971.5782-272.2-145.00) + 2454358.25]**

**= -30.52 Kcal/mol**

**3) 3d -> 3e** + H$^+$+ e$^-$

$$\Delta Gd - e = [(\Delta Ge + \Delta GH^+ + \Delta Gelectron) - \Delta Gd]$$

**= [(-2501268.2323-272.2 -145.00) + 2501647.5619]**

**= -37.87 Kcal/mol**

**4) 3e -> 3f** + e$^-$

$$\Delta Ge - f = [(\Delta Gf + \Delta Gelectron) - \Delta Ge]$$

**= [(-2501144.8012-145.00) +2501268.2323]**

**= -21.57 Kcal/mol**

**<u>Including TS</u>**

**1) 3c** + $H_2O$ -> **3-TS**

$$TS - \Delta Gc = [\Delta GTS - (\Delta Gc + \Delta GH2O)]$$

**= [(-2501908.9824) -( -2453971.5782-47973.644172)]**

**= 36.23 Kcal/mol**

**2) 3-TS -> 3d + H$^+$**

$$d - \Delta GTS = [(\Delta Gd + \Delta GH^+) - \Delta GTS]$$

**= [(-2501647.5619-272.2)- (-2501908.9824)]**

**= -10.77 Kcal/mol**

Here during the calculation ΔGelectron is taken = -145.00 Kcal/mol.

$[GFc^{+} - GFc]$ **= (320237.0347-320145.3491) = 91.69 Kcal/mol**

$\Delta Gredox = \Delta G4f - \Delta G4e$ **= [(-2501144.8012)- (-2501268.2323)] = 123.4311 Kcal/mol**

$\Delta Gabsolute, redox = Gredox - GFc/Fc^{+}$ **= 123.43-91.69= 31.74 Kcal/mol**

$$\Delta G^{o} = -nFE^{o}$$

$$E^{o} = -\Delta G^{o}/F \; [F = 23.06 \; Kcal/mol/V]$$

**= 1.37V** (Vs $FeCp_2$) (taking only the magnitude)

**=2.01V** (Vs NHE) **[$Evs\ NHE = Evs\ FeCp2 + 0.64$]**

Accordingly, the theoretically estimated potential is 1.37 V vs $FeCp_2$(taking only the magnitude as calculated value is reported as an oxidation potential). The free energy of the electron (-145.00 kcal mol$^{-1}$), obtained from theoretical calculations, was used consistently to convert computed free-energy changes into electrode potentials. Potentials referenced to the $FeCp_2$/$FeCp_{2+}$ couple were subsequently converted to the NHE scale using the established literature offset.

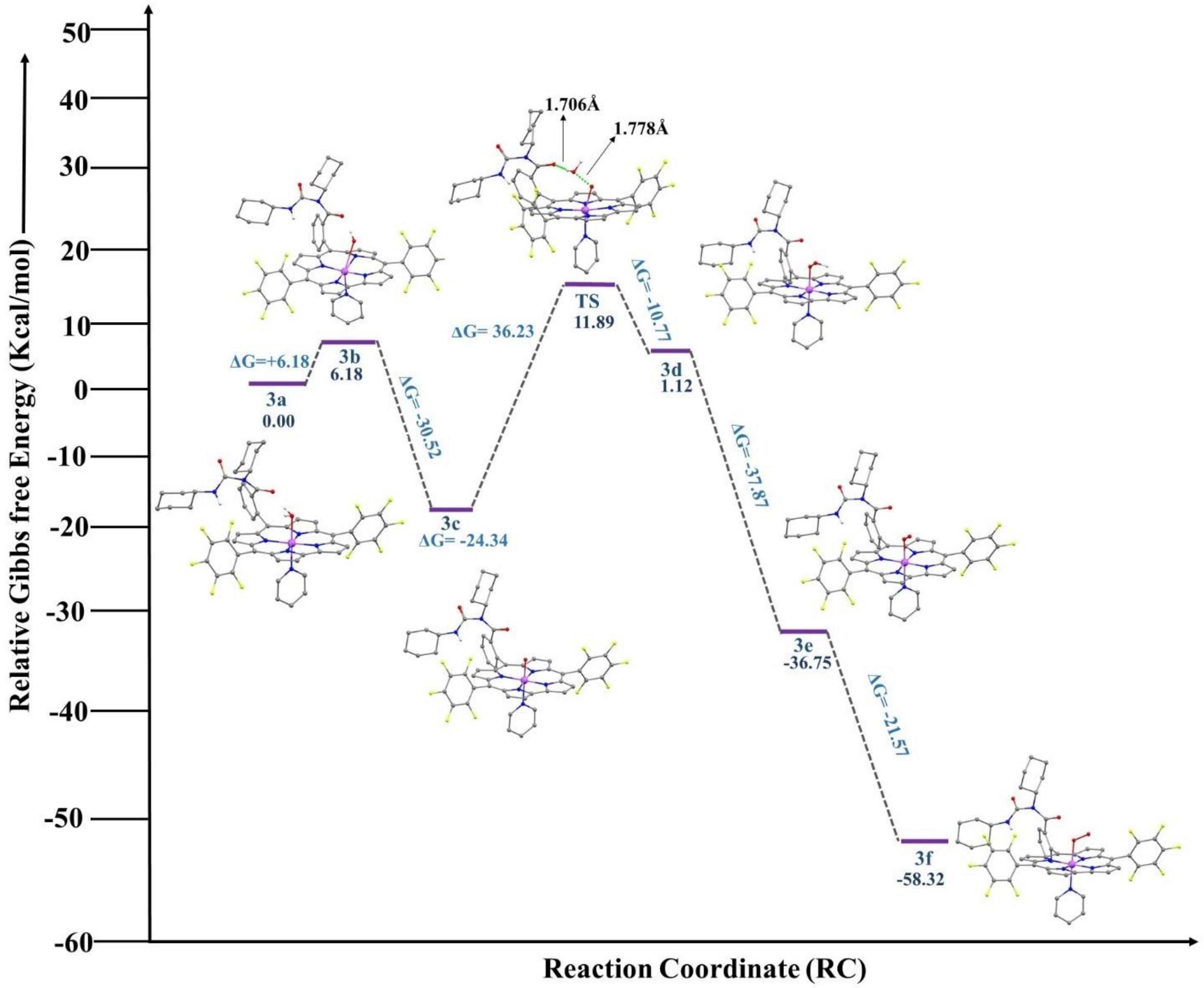


**Figure S45** Free-energy profile (ΔG, kcal mol$^{-1}$) for O-O bond formation in the corrolato cobalt (III) complex [Co$^{III}$(corrole$^{\bullet 2-}$)(O$^{\bullet -}$)(Py)]$^{0}$, (**3c**), proceeding through a water nucleophilic attack (WNA) transition state. The cobalt center is shown in violet, oxygen atoms in red, and selected atoms of the secondary coordination sphere are highlighted.

**Detailed theoretical calculations for Truncated version of cobalt complex 3 used to construct the energy profile diagram.**

| Intermediate | Electronic Energy **(H)** | Thermal correction to Gibbs Free Energy **(H)** | Thermal correction to Gibbs Free Energy **(Kcal/mol)** | Sum of electronic and thermal free energies **(H)** | Sum of electronic and thermal free energies **(Kcal/mol)** |
|---|---|---|---|---|---|
| **3c-T** | -3837.3586 | 0.773097 | 485.125795 | -3836.5855 | -2407494.2633 |
| **3d-T** | -3913.3488 | 0.786899 | 493.786683 | -3912.5619 | -2455170.1843 |
| **3e-T** | -3912.7311 | 0.775149 | 486.413445 | -3911.956 | -2454789.976 |
| **3f-T** | -3912.5392 | 0.777193 | 487.696075 | -3911.762 | -2454668.239 |
| **3T-TS** | -3913.7729 | 0.800171 | 502.114991 | -3912.9728 | -2455428.028 |
| **$H^+$** | Reference: *J. Am. Chem. Soc.* **2018**, *140*, 1557–1565 | | | | -272.2 |
| **$H_2O$** | -76.45447 | 0.003618 | 2.27032976 | -76.450852 | -47973.644172 |

To quantify the effect of intramolecular hydrogen bonding from the pendant motif, we also constructed a truncated model in which the carbonyl group was replaced by two hydrogen atoms, thereby preventing hydrogen-bond formation. The energies of intermediates **3c-T**, the corresponding **TS(3TS-T)**, and **3d-T** were recalculated using this model. The truncated system shows a significantly higher TS barrier, confirming that hydrogen bonding plays a key role in lowering the activation energy for the WNA step. The results of these calculations are provided below. Here, **T** denotes the truncated model employed to isolate the effect of hydrogen bonding.

In these calculations, the free energy of the electron $\Delta G_{electron}$ was taken as -143.39 kcal $mol^{-1}$.

$[GFc^{+} - GFc]$ = **(320237.0347-320145.3491) = 91.69 Kcal/mol**

$\Delta Gredox = \Delta G3fT - \Delta G3eT$**=[(-2454668.239)- (-2454789.976)] = 121.737 Kcal/mol**

$$\Delta Gabsolute, redox = Gredox - GFc/Fc^{+} = 121.737 - 91.69$$
$$= 30.047\ Kcal/mol$$
$$\Delta G^{o} = -nFE^{o}$$
$$E^{o} = -\Delta G^{o}/F\ [F = 23.06\ Kcal/mol/V]$$

= **1.3V** (Vs $FeCp_2$) (taking only the magnitude)

=**1.94V** (Vs NHE) $[Evs\ NHE = Evs\ FeCp2 + 0.64]$

Accordingly, the theoretically estimated potential is 1.3 V vs $FeCp_2$(taking only the magnitude as calculated value is reported as an oxidation potential). The free energy of the electron (-143.39 kcal $mol^{-1}$), obtained from theoretical calculations, was used consistently to convert computed free-energy changes into electrode potentials. Potentials referenced to the $FeCp_2$ /$FeCp_{2+}$ couple were subsequently converted to the NHE scale using the established literature offset.

**1) 3c-T** + $3H_2O$ -> **3T-TS**

$$\Delta Gc - TS = [\Delta GTS - (\Delta Gc + \Delta GH2O)]$$

**= [(-2455428.028) -( -2407494.2633-47973.644172)]**

**= 39.87 Kcal/mol**

**2) 3T-TS -> 3d-T + H$^{+}$**

$$\Delta GTS - d = [(\Delta Gd + \Delta GH^{+}) - (\Delta GTS)]$$

**= [(-2455170.1843-272.2)- (-2455428.028)]**

**= -14.36 Kcal/mol**

**3) 3d-T -> 3e-T** + $H^{+}$+ $e^{-}$

$$\Delta Gd - e = [(\Delta Ge + \Delta GH^{+} + \Delta Gelectron) - \Delta Gd]$$

**= [(-2454789.976-272.2 -143.39) + 2455170.1843]**

**= -35.38 Kcal/mol**

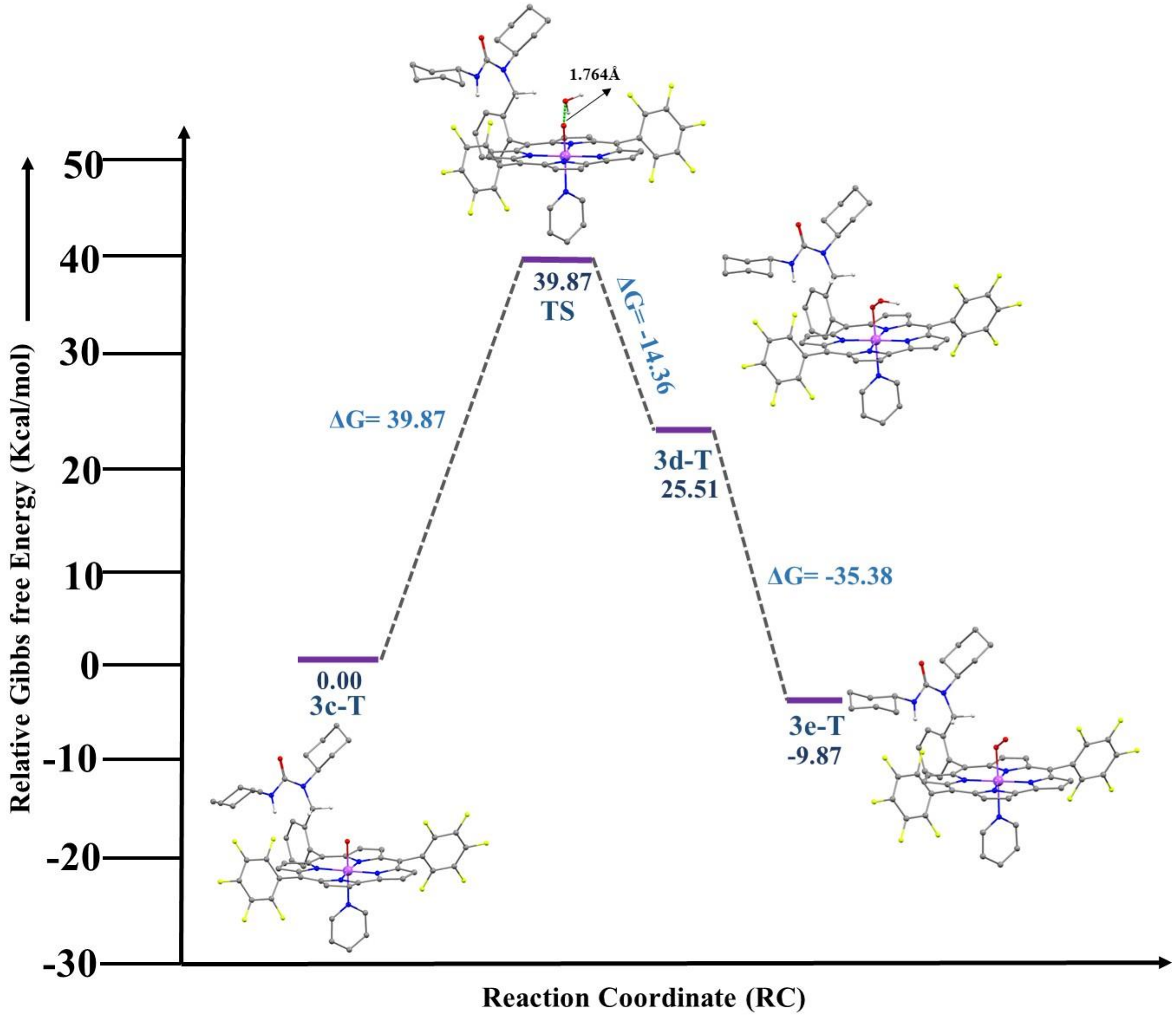


**Figure S46** Free-energy profile (ΔG, kcal $mol^{-1}$) for O-O bond formation in the corrolato cobalt (III) complex $[Co^{III}(corrole^{\bullet 2-})(O^{\bullet -})(Py)]^0$, (**3c-T**), proceeding through a water nucleophilic attack (WNA) transition state. The cobalt center is shown in violet, oxygen atoms in red, and selected atoms of the secondary coordination sphere are highlighted.

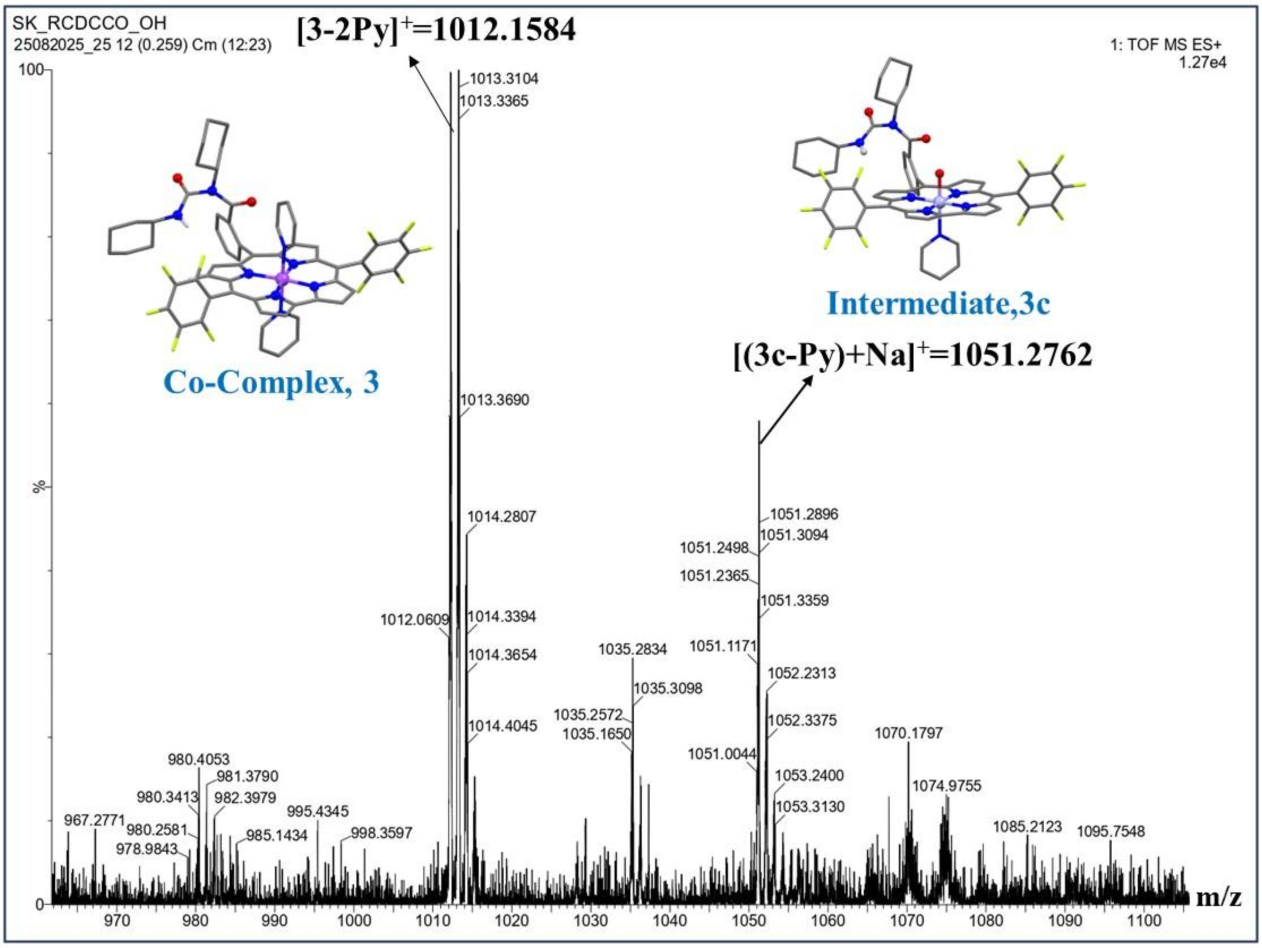


**Figure S47** ESI-MS data for water Oxidation intermediate for complex **3**.

## Appendix 1 Optimized Cartesian Coordinates of 1.

Cartesian coordinates of the optimized structure (in Å). Corrole, **1** was optimized at the B3LYP level of theory and 6-311G (d, p) basis set.

FB Corrole, **1**

(E = -3444.5077 hartrees)

| | | | |
|---|---|---|---|
| F | -5.57843 | -1.51255 | -2.09516 |
| F | -5.27887 | -2.11905 | 2.59831 |
| F | 4.81187 | -0.84644 | -1.43068 |
| F | -7.96412 | -2.13025 | 2.75612 |
| F | 6.78368 | -5.50176 | 0.58315 |
| F | 4.14326 | -4.99380 | 0.76284 |
| F | 7.45064 | -1.35852 | -1.57524 |
| F | -9.47267 | -1.85846 | 0.50492 |
| O | -0.98699 | 3.31408 | 0.45957 |
| N | -1.73200 | -0.79685 | 0.97287 |
| F | -8.26391 | -1.55867 | -1.91736 |
| F | 8.45185 | -3.68912 | -0.57913 |
| N | 1.15633 | -1.18811 | 0.74897 |
| N | 1.22509 | 3.80364 | 0.02576 |
| N | -1.89204 | -2.73979 | -0.96199 |
| O | 1.95883 | 4.88179 | -1.88604 |
| N | -0.19396 | 4.17307 | -1.86068 |
| N | 0.70744 | -3.12321 | -0.96351 |
| C | 0.00524 | 0.62417 | 1.99540 |
| C | 2.50025 | 0.14473 | 2.06105 |
| H | 2.78482 | 0.90878 | 2.76693 |
| C | -6.13556 | -1.67813 | -0.88881 |
| C | 0.89475 | -4.94092 | -2.23729 |

| | | | |
|---|---|---|---|
| H | 0.64273 | -5.77628 | -2.87168 |
| C | -0.03301 | -4.03802 | -1.65381 |
| C | -1.44190 | -3.89821 | -1.56498 |
| C | 4.38289 | -2.90115 | -0.32494 |
| C | -3.58705 | 0.37113 | 1.45828 |
| H | -4.61874 | 0.68493 | 1.47673 |
| C | 0.17964 | 3.40023 | 0.83569 |
| C | -2.51543 | 1.06037 | 1.96093 |
| H | -2.53242 | 2.02398 | 2.44118 |
| C | 0.71890 | 4.04429 | 4.53018 |
| H | 0.89271 | 4.90041 | 5.17179 |
| C | -3.65696 | -4.08512 | -1.10460 |
| H | -4.64605 | -4.49407 | -0.97061 |
| C | 0.43398 | 3.12273 | 2.30284 |
| C | -3.22682 | -2.82232 | -0.63233 |
| C | 2.04175 | -3.39793 | -1.01776 |
| C | 1.14637 | -0.12979 | 1.60621 |
| C | -7.52222 | -1.69577 | -0.81382 |
| C | -3.11538 | -0.82494 | 0.82690 |
| C | 7.14428 | -3.43629 | -0.49884 |
| C | -1.32161 | 0.32309 | 1.67270 |
| C | 3.31434 | -0.75419 | 1.44925 |
| H | 4.38345 | -0.84704 | 1.56213 |
| C | 0.66306 | 4.21239 | 3.15074 |
| H | 0.78337 | 5.20130 | 2.72411 |
| C | -8.14166 | -1.84387 | 0.42134 |
| C | 2.47222 | -1.57989 | 0.60751 |
| C | -2.56369 | -4.74205 | -1.67282 |
| H | -2.55042 | -5.75375 | -2.04738 |
| C | 2.17034 | -4.54539 | -1.85448 |
| H | 3.09680 | -5.02596 | -2.12660 |

| | | | |
|---|---|---|---|
| C | -0.51283 | 4.72341 | -3.18287 |
| H | 0.39319 | 4.62831 | -3.78385 |
| C | 1.02840 | 4.33591 | -1.31678 |
| C | 3.49594 | 2.79706 | -0.42337 |
| H | 3.01186 | 1.82189 | -0.52648 |
| H | 3.55779 | 3.24687 | -1.41494 |
| C | -3.84657 | -1.80887 | 0.14281 |
| C | 0.30682 | 1.69021 | 4.24000 |
| H | 0.16841 | 0.70139 | 4.66219 |
| C | 4.91009 | 2.62408 | 0.15270 |
| H | 4.85589 | 2.06538 | 1.09568 |
| H | 5.50639 | 2.01414 | -0.53243 |
| C | 0.24244 | 1.83943 | 2.84999 |
| C | -5.98383 | -1.96382 | 1.47055 |
| C | 6.63171 | -2.24783 | -1.00449 |
| C | -0.89614 | 6.21550 | -3.10246 |
| H | -0.09350 | 6.76211 | -2.60105 |
| H | -0.95335 | 6.60661 | -4.12603 |
| C | 2.92435 | -2.61547 | -0.24032 |
| C | -5.32655 | -1.81012 | 0.24513 |
| C | 2.64588 | 3.69485 | 0.49450 |
| H | 2.57507 | 3.18077 | 1.45340 |
| C | 0.54267 | 2.77681 | 5.07740 |
| H | 0.58314 | 2.63307 | 6.15103 |
| C | 5.58981 | 3.97592 | 0.40714 |
| H | 6.57994 | 3.82499 | 0.84950 |
| H | 5.74667 | 4.48945 | -0.54931 |
| C | -3.00438 | 4.12471 | -3.13614 |
| H | -2.98900 | 3.70626 | -2.12185 |
| H | -3.78084 | 3.57364 | -3.67610 |
| C | 4.93134 | -4.08457 | 0.17368 |

| | | | |
|---|---|---|---|
| C | 3.31654 | 5.05591 | 0.75008 |
| H | 2.70827 | 5.64176 | 1.44652 |
| H | 3.36429 | 5.61071 | -0.18702 |
| C | 4.73031 | 4.86071 | 1.32055 |
| H | 5.20422 | 5.83703 | 1.46260 |
| H | 4.66706 | 4.39864 | 2.31502 |
| C | -7.36949 | -1.97684 | 1.56884 |
| C | -1.64556 | 3.90185 | -3.82094 |
| H | -1.72248 | 4.20292 | -4.87197 |
| H | -1.37692 | 2.84106 | -3.81429 |
| C | -3.36121 | 5.61713 | -3.06403 |
| H | -3.52544 | 5.99742 | -4.08092 |
| H | -4.30395 | 5.75328 | -2.52457 |
| C | 5.26788 | -1.99813 | -0.91938 |
| C | 6.29086 | -4.36030 | 0.09137 |
| C | -2.24191 | 6.43042 | -2.39660 |
| H | -2.15315 | 6.13054 | -1.34539 |
| H | -2.49604 | 7.49499 | -2.39414 |
| H | 0.41507 | -2.37042 | -0.32753 |
| H | -0.90827 | 3.75929 | -1.27095 |
| H | -1.41346 | -1.85570 | -1.04416 |
| H | -1.11739 | -1.59198 | 0.89082 |

**Appendix 2 Optimized Cartesian Coordinates of 3.**

Cartesian coordinates of the optimized structure (in Å). Corrolato-Cobalt, **3** was optimized at the B3LYP level of theory and 6-311G (d, p) basis set. The LANL2DZ pseudopotential was used for the Co atom.

**Corrolato-Cobalt (III), 3**

(E = -4084.6003 hartrees)

| | | | |
|---|---|---|---|
| Co | -1.46812 | 1.29807 | 0.01979 |
| F | 4.30234 | 6.79610 | -0.71863 |
| F | 2.04520 | 5.37212 | -1.09586 |
| F | -5.82602 | -1.03190 | 2.35514 |
| F | 6.09713 | 6.04035 | 1.18782 |
| F | -8.28257 | -2.14381 | 2.35643 |
| F | 3.34511 | 2.42444 | 2.38097 |
| F | -9.64207 | -2.46408 | 0.01797 |
| F | 5.60840 | 3.84593 | 2.72621 |
| N | -2.12308 | -0.38555 | -0.61280 |
| N | 0.37062 | 0.94773 | -0.38681 |
| F | -6.07845 | -0.51912 | -2.34900 |
| O | 0.59800 | -3.28913 | 0.66362 |
| N | -3.20640 | 1.85346 | 0.48489 |
| N | -1.21056 | 0.50765 | 1.87444 |
| N | -1.04698 | 3.02601 | 0.64764 |
| N | -1.74077 | 2.08395 | -1.85055 |
| N | 2.82045 | -3.74852 | 0.53836 |
| F | -8.52648 | -1.65007 | -2.33119 |
| C | 2.60380 | 1.20784 | -0.74025 |
| H | 3.58648 | 1.65467 | -0.75062 |
| C | 1.42925 | 1.80372 | -0.15929 |

| | | | |
|---|---|---|---|
| C | 2.22943 | 0.02121 | -1.31009 |
| H | 2.83898 | -0.64921 | -1.89438 |
| C | 0.81994 | -0.15142 | -1.07079 |
| C | 0.02810 | -1.25359 | -1.45040 |
| C | 1.48469 | -3.37065 | -1.53543 |
| C | 0.13091 | 3.67594 | 0.82967 |
| C | 1.59459 | -3.43115 | -0.03125 |
| C | 1.35178 | 3.07757 | 0.44484 |
| C | 0.69280 | -2.38915 | -2.17277 |
| C | 0.51957 | -2.47698 | -3.55907 |
| H | -0.07374 | -1.71541 | -4.05174 |
| C | -1.35671 | -1.35304 | -1.21061 |
| C | -4.40462 | 1.21885 | 0.47024 |
| C | -2.09366 | 3.80457 | 1.05561 |
| C | -4.51410 | -0.10947 | -0.00047 |
| C | 2.89607 | 4.98019 | -0.13369 |
| C | 4.05666 | 5.72304 | 0.04249 |
| C | 3.55357 | 3.49272 | 1.59957 |
| C | -3.42198 | -0.84218 | -0.50706 |
| C | -3.34309 | 3.12859 | 0.95512 |
| C | -7.73223 | -1.74283 | 1.20462 |
| C | -0.55227 | -1.33748 | 3.25122 |
| H | -0.16657 | -2.34753 | 3.28323 |
| C | 4.72492 | 4.21561 | 1.79133 |
| C | 4.97553 | 5.33718 | 1.01058 |
| C | -6.46736 | -1.16686 | 1.18717 |
| C | -5.38087 | 2.15524 | 0.95672 |
| H | -6.43839 | 1.96491 | 1.06208 |
| C | 2.60831 | 3.85125 | 0.63604 |
| C | -0.72402 | -0.73690 | 2.01037 |
| H | -0.46783 | -1.28059 | 1.11607 |

| | | | |
|---|---|---|---|
| C | -0.85021 | 2.94177 | -2.37420 |
| H | 0.00999 | 3.18150 | -1.76911 |
| C | 2.82872 | -4.19460 | 1.96080 |
| H | 1.94974 | -3.71309 | 2.39146 |
| C | 2.03654 | -4.41353 | -2.29198 |
| H | 2.61585 | -5.18384 | -1.79822 |
| C | -3.46010 | -2.17515 | -1.05584 |
| H | -4.33554 | -2.80469 | -1.10036 |
| C | 1.83531 | -4.48900 | -3.66610 |
| H | 2.26230 | -5.31105 | -4.22897 |
| C | -8.42696 | -1.90972 | 0.01316 |
| C | -0.18252 | 4.96052 | 1.39391 |
| H | 0.53312 | 5.72333 | 1.66147 |
| C | -1.55860 | 5.03773 | 1.52999 |
| H | -2.12423 | 5.87047 | 1.92030 |
| C | -2.20387 | -2.48577 | -1.48784 |
| H | -1.87506 | -3.41031 | -1.93470 |
| C | 1.07949 | -3.51115 | -4.30425 |
| H | 0.91834 | -3.55409 | -5.37566 |
| C | -2.83013 | 1.77493 | -2.57286 |
| H | -3.53212 | 1.08788 | -2.12712 |
| C | -1.01220 | 3.51063 | -3.63074 |
| H | -0.26160 | 4.19766 | -4.00004 |
| C | -5.86182 | -0.74288 | 0.00302 |
| C | 4.06658 | -3.69570 | 2.71885 |
| H | 4.13422 | -2.60592 | 2.64110 |
| H | 4.97199 | -4.11459 | 2.26735 |
| C | -2.13740 | 3.18782 | -4.38005 |
| H | -2.29204 | 3.61758 | -5.36304 |
| C | -1.53249 | 1.19150 | 2.98568 |
| H | -1.92015 | 2.18850 | 2.84783 |

| | | | |
|---|---|---|---|
| C | -3.06160 | 2.30361 | -3.83601 |
| H | -3.95840 | 2.01846 | -4.37104 |
| C | -0.88786 | -0.63250 | 4.39942 |
| H | -0.76774 | -1.07636 | 5.38107 |
| C | -4.72096 | 3.33538 | 1.25618 |
| H | -5.16029 | 4.24196 | 1.64447 |
| C | -6.59128 | -0.91964 | -1.17363 |
| C | 2.65694 | -5.71736 | 2.08964 |
| H | 1.73970 | -6.01918 | 1.57515 |
| H | 3.49234 | -6.20968 | 1.58466 |
| C | -7.85450 | -1.49716 | -1.18378 |
| C | -1.38450 | 0.65866 | 4.25834 |
| H | -1.66262 | 1.25650 | 5.11690 |
| C | 4.00054 | -4.12045 | 4.19491 |
| H | 3.15765 | -3.61290 | 4.68162 |
| H | 4.90441 | -3.78599 | 4.71331 |
| C | 2.60261 | -6.13756 | 3.56630 |
| H | 1.69367 | -5.73057 | 4.02859 |
| H | 2.52400 | -7.22679 | 3.63743 |
| C | 3.83017 | -5.63889 | 4.34102 |
| H | 4.72747 | -6.14083 | 3.95791 |
| H | 3.74769 | -5.90882 | 5.39876 |
| O | 4.68521 | -4.90704 | -0.22903 |
| C | 4.06983 | -3.85773 | -0.17687 |
| N | 4.49761 | -2.68692 | -0.70517 |
| H | 3.96691 | -1.85229 | -0.49967 |
| C | 6.79479 | -1.75948 | -0.68613 |
| H | 6.97528 | -2.23572 | 0.28175 |
| H | 6.39226 | -0.75882 | -0.47893 |
| C | 6.81245 | -1.85085 | -3.64902 |
| H | 7.20233 | -2.85624 | -3.85229 |

| | | | |
|---|---|---|---|
| H | 6.61776 | -1.39108 | -4.62272 |
| C | 5.74417 | -2.56981 | -1.46450 |
| H | 6.10615 | -3.59454 | -1.57603 |
| C | 8.10415 | -1.63214 | -1.47921 |
| H | 8.81659 | -1.01606 | -0.92225 |
| H | 8.56180 | -2.62461 | -1.57690 |
| C | 7.86658 | -1.04494 | -2.87734 |
| H | 7.52765 | -0.00531 | -2.78100 |
| H | 8.80546 | -1.01514 | -3.43889 |
| C | 5.50063 | -1.96990 | -2.85865 |
| H | 5.05607 | -0.97178 | -2.74947 |
| H | 4.77523 | -2.58419 | -3.39918 |

**Appendix 3 Optimized Cartesian Coordinates of 4.**

Cartesian coordinates of the optimized structure (in Å). Corrolato-Cobalt, **4** was optimized at the B3LYP level of theory and 6-311G (d, p) basis set. The LANL2DZ pseudopotential was used for the Co atom.

**Corrolato-Cobalt (III), 4**

(E = -3316.9808 hartrees)

| | | | |
|---|---|---|---|
| Co | 0.00268 | -0.49383 | 0.11997 |
| F | 7.66901 | -1.62779 | 2.15299 |
| F | 4.98883 | -1.37712 | 2.28521 |
| F | -4.71313 | -1.89685 | -2.23429 |
| F | 8.96401 | -1.27510 | -0.21948 |
| F | -7.39260 | -2.16740 | -2.35574 |
| F | 4.86473 | -0.44477 | -2.36171 |
| F | -8.94955 | -1.33983 | -0.27757 |
| F | 7.54737 | -0.67755 | -2.46958 |
| N | -1.41638 | 0.78183 | -0.03042 |
| N | 1.41789 | 0.78502 | -0.04779 |
| F | -5.11938 | 0.01656 | 2.08394 |
| N | -1.22640 | -1.91098 | 0.30690 |
| N | 0.00250 | -0.73303 | -1.90778 |
| N | 1.23554 | -1.90730 | 0.31964 |
| N | 0.00077 | -0.24107 | 2.14375 |
| F | -7.79831 | -0.24491 | 1.93683 |
| C | 3.47968 | 1.74944 | -0.13083 |
| H | 4.55380 | 1.85581 | -0.14888 |
| C | 2.76850 | 0.49741 | -0.07087 |
| C | 2.55524 | 2.75531 | -0.12669 |
| H | 2.74814 | 3.81594 | -0.14891 |
| C | 1.24765 | 2.14579 | -0.08586 |

| | | | |
|---|---|---|---|
| C | -0.00094 | 2.80777 | -0.10609 |
| C | 0.48169 | 5.00156 | -1.26622 |
| C | 2.58885 | -1.96200 | 0.23499 |
| C | 3.34647 | -0.78558 | 0.03100 |
| C | -0.01014 | 4.30287 | -0.15511 |
| C | -0.51448 | 5.05391 | 0.91497 |
| H | -0.89216 | 4.54074 | 1.79249 |
| C | -1.24694 | 2.13999 | -0.11122 |
| C | -2.57949 | -1.96645 | 0.22553 |
| C | 0.71736 | -3.15352 | 0.53338 |
| C | -3.33877 | -0.79462 | 0.00390 |
| C | 5.58976 | -1.21072 | 1.09462 |
| C | 6.97107 | -1.33950 | 1.04613 |
| C | 5.52836 | -0.73109 | -1.22956 |
| C | -2.76424 | 0.48827 | -0.10932 |
| C | -0.70772 | -3.15429 | 0.53378 |
| C | -6.82509 | -1.63060 | -1.26709 |
| C | -0.01351 | 0.24991 | -4.09377 |
| H | -0.02772 | 1.15440 | -4.68778 |
| C | 6.90963 | -0.84902 | -1.30368 |
| C | 7.63364 | -1.15652 | -0.15991 |
| C | -5.44664 | -1.48535 | -1.18711 |
| C | -2.95721 | -3.33907 | 0.42791 |
| H | -3.96752 | -3.72061 | 0.43443 |
| C | 4.82906 | -0.90537 | -0.03464 |
| C | -0.01381 | 0.34657 | -2.70881 |
| H | -0.02780 | 1.31057 | -2.22547 |
| C | 1.15336 | -0.18115 | 2.83233 |
| H | 2.06602 | -0.26584 | 2.26366 |
| C | 0.46984 | 6.39367 | -1.30308 |
| H | 0.85735 | 6.90509 | -2.17869 |

| | | | |
|---|---|---|---|
| C | -3.47193 | 1.73316 | -0.27171 |
| H | -4.54136 | 1.83304 | -0.37989 |
| C | -0.03581 | 7.14337 | -0.23502 |
| C | -7.62081 | -1.20773 | -0.21123 |
| C | 2.96610 | -3.33808 | 0.41317 |
| H | 3.97507 | -3.72275 | 0.39846 |
| C | 1.80512 | -4.07260 | 0.60044 |
| H | 1.73096 | -5.13797 | 0.76059 |
| C | -2.54978 | 2.74120 | -0.26689 |
| H | -2.73914 | 3.79763 | -0.37182 |
| C | -0.52550 | 6.44633 | 0.87466 |
| H | -0.91494 | 6.99936 | 1.72363 |
| C | -1.15425 | -0.14874 | 2.82492 |
| H | -2.06517 | -0.20370 | 2.24986 |
| C | 1.19142 | -0.02155 | 4.21118 |
| H | 2.14935 | 0.02101 | 4.71290 |
| C | -4.82048 | -0.92588 | -0.07307 |
| C | -0.00361 | 0.07794 | 4.91501 |
| H | -0.00531 | 0.20271 | 5.99107 |
| C | 0.02053 | -1.94834 | -2.48091 |
| H | 0.03369 | -2.79902 | -1.81771 |
| C | -1.19648 | 0.01092 | 4.20359 |
| H | -2.15602 | 0.07989 | 4.69928 |
| C | 0.00531 | -1.00827 | -4.68549 |
| H | 0.00637 | -1.11514 | -5.76345 |
| C | -1.79559 | -4.07205 | 0.61796 |
| H | -1.72119 | -5.13499 | 0.79331 |
| C | -5.65096 | -0.51799 | 0.97112 |
| C | -7.03191 | -0.64728 | 0.91407 |
| C | 0.02280 | -2.12545 | -3.85825 |
| H | 0.03796 | -3.12896 | -4.26300 |

| | | | |
|---|---|---|---|
| H | 0.87330 | 4.44794 | -2.11240 |
| C | -0.07498 | 8.65158 | -0.29055 |
| H | 0.77055 | 9.05156 | -0.85496 |
| H | -0.98919 | 9.00214 | -0.78208 |
| H | -0.05556 | 9.08713 | 0.71079 |

**Appendix 4 Optimized Cartesian Coordinates for [Co$^{III}$(Corrole$^{\bullet 2-}$) Py$_2$]$^+$ (S=1/2) of 3.**

| | | | |
|---|---|---|---|
| Co | -1.54022 | 1.30626 | -0.08167 |
| F | 4.13640 | 6.93975 | -0.72938 |
| F | 1.90684 | 5.48679 | -1.17795 |
| F | -5.69975 | -1.11525 | 2.39480 |
| F | 5.85098 | 6.23297 | 1.26460 |
| F | -8.08762 | -2.37487 | 2.51278 |
| F | 3.08984 | 2.59871 | 2.38412 |
| F | -9.49034 | -2.85783 | 0.22952 |
| F | 5.32089 | 4.05843 | 2.81558 |
| N | -2.11397 | -0.43856 | -0.61257 |
| N | 0.31839 | 1.00286 | -0.42011 |
| F | -6.10523 | -0.81142 | -2.31113 |
| O | 0.74311 | -3.06668 | 0.69395 |
| N | -3.30588 | 1.82270 | 0.31083 |
| N | -1.29075 | 0.64383 | 1.83244 |
| N | -1.20416 | 3.08139 | 0.45008 |
| N | -1.79093 | 1.95673 | -2.00019 |
| N | 2.93538 | -3.65152 | 0.58523 |
| F | -8.49334 | -2.07243 | -2.17972 |
| C | 2.55080 | 1.32444 | -0.71672 |
| H | 3.51880 | 1.80012 | -0.70510 |
| C | 1.33682 | 1.89006 | -0.19836 |
| C | 2.23461 | 0.10680 | -1.25954 |
| H | 2.89357 | -0.56971 | -1.77868 |
| C | 0.82993 | -0.10078 | -1.05573 |
| C | 0.07656 | -1.24194 | -1.41118 |
| C | 1.60327 | -3.30456 | -1.49656 |
| C | -0.04627 | 3.77598 | 0.65975 |

| | | | |
|---|---|---|---|
| C | 1.73216 | -3.30992 | 0.00584 |
| C | 1.19196 | 3.19960 | 0.36300 |
| C | 0.77431 | -2.35480 | -2.13313 |
| C | 0.57481 | -2.44501 | -3.51446 |
| H | -0.04692 | -1.70516 | -4.00447 |
| C | -1.31243 | -1.39629 | -1.17462 |
| C | -4.48048 | 1.12853 | 0.34236 |
| C | -2.26975 | 3.83494 | 0.79351 |
| C | -4.52014 | -0.22114 | -0.02661 |
| C | 2.72640 | 5.11995 | -0.18100 |
| C | 3.87105 | 5.87482 | 0.03432 |
| C | 3.32810 | 3.66203 | 1.60243 |
| C | -3.38313 | -0.94605 | -0.49359 |
| C | -3.50614 | 3.09836 | 0.70432 |
| C | -7.59534 | -1.98765 | 1.33134 |
| C | -0.49434 | -1.04144 | 3.33755 |
| H | -0.00631 | -1.99991 | 3.45133 |
| C | 4.47783 | 4.40256 | 1.83650 |
| C | 4.74849 | 5.51387 | 1.04832 |
| C | -6.37031 | -1.33910 | 1.25552 |
| C | -5.50224 | 2.05070 | 0.77295 |
| H | -6.54999 | 1.81923 | 0.88854 |
| C | 2.42544 | 3.99988 | 0.59388 |
| C | -0.68652 | -0.53684 | 2.05879 |
| H | -0.34718 | -1.10446 | 1.20809 |
| C | -0.90656 | 2.79670 | -2.56694 |
| H | -0.06157 | 3.09838 | -1.96997 |
| C | 2.94729 | -3.97033 | 2.04462 |
| H | 2.11035 | -3.39936 | 2.44778 |
| C | 2.15776 | -4.34164 | -2.25722 |
| H | 2.75064 | -5.10509 | -1.77142 |

| | | | |
|---|---|---|---|
| C | -3.37486 | -2.29620 | -0.98434 |
| H | -4.22448 | -2.96039 | -1.00503 |
| C | 1.93725 | -4.42571 | -3.62859 |
| H | 2.37087 | -5.24210 | -4.19349 |
| C | -8.31142 | -2.23621 | 0.16779 |
| C | -0.42265 | 5.07439 | 1.16720 |
| H | 0.25864 | 5.86630 | 1.43762 |
| C | -1.79868 | 5.11044 | 1.24376 |
| H | -2.41179 | 5.93066 | 1.58327 |
| C | -2.10329 | -2.57011 | -1.41145 |
| H | -1.73921 | -3.49355 | -1.83157 |
| C | 1.15446 | -3.46673 | -4.26241 |
| H | 0.98122 | -3.51671 | -5.33081 |
| C | -2.85914 | 1.57711 | -2.72434 |
| H | -3.56105 | 0.90812 | -2.25387 |
| C | -1.05245 | 3.27341 | -3.86167 |
| H | -0.30669 | 3.94651 | -4.26333 |
| C | -5.83056 | -0.92874 | 0.03781 |
| C | 4.23022 | -3.48562 | 2.73286 |
| H | 4.36313 | -2.41373 | 2.55866 |
| H | 5.10005 | -3.99731 | 2.30688 |
| C | -2.15454 | 2.87609 | -4.61004 |
| H | -2.29496 | 3.23203 | -5.62319 |
| C | -1.71688 | 1.35191 | 2.89379 |
| H | -2.19945 | 2.29534 | 2.69754 |
| C | -3.07306 | 2.01250 | -4.02421 |
| H | -3.95106 | 1.67205 | -4.55691 |
| C | -0.93536 | -0.30890 | 4.43289 |
| H | -0.79909 | -0.67900 | 5.44164 |
| C | -4.89638 | 3.27054 | 0.99627 |
| H | -5.36820 | 4.18197 | 1.32891 |

| | | | |
|---|---|---|---|
| C | -6.57334 | -1.19132 | -1.11193 |
| C | 2.68791 | -5.46268 | 2.30856 |
| H | 1.74670 | -5.75583 | 1.83384 |
| H | 3.48456 | -6.04803 | 1.84078 |
| C | -7.80048 | -1.83697 | -1.06061 |
| C | -1.55766 | 0.91226 | 4.19986 |
| H | -1.92225 | 1.52790 | 5.01147 |
| C | 4.16974 | -3.77508 | 4.24160 |
| H | 3.37041 | -3.17453 | 4.69343 |
| H | 5.10452 | -3.45475 | 4.71115 |
| C | 2.63825 | -5.74812 | 3.81732 |
| H | 1.76599 | -5.24510 | 4.25386 |
| H | 2.49549 | -6.82013 | 3.98390 |
| C | 3.90982 | -5.26091 | 4.52539 |
| H | 4.76686 | -5.85070 | 4.17643 |
| H | 3.83071 | -5.42931 | 5.60391 |
| O | 4.68374 | -4.98000 | -0.19273 |
| C | 4.17376 | -3.87246 | -0.13472 |
| N | 4.69635 | -2.74971 | -0.66414 |
| H | 4.26833 | -1.86975 | -0.41487 |
| C | 7.10560 | -2.16069 | -0.58693 |
| H | 7.22916 | -2.76673 | 0.31517 |
| H | 6.84311 | -1.14648 | -0.26002 |
| C | 7.08249 | -1.89990 | -3.53942 |
| H | 7.33218 | -2.91572 | -3.87033 |
| H | 6.93982 | -1.30271 | -4.44490 |
| C | 5.94907 | -2.72604 | -1.42889 |
| H | 6.16514 | -3.77007 | -1.66585 |
| C | 8.41222 | -2.11858 | -1.39348 |
| H | 9.20586 | -1.67444 | -0.78528 |
| H | 8.72898 | -3.14435 | -1.62016 |

| | | | |
|---|---|---|---|
| C | 8.24214 | -1.33929 | -2.70472 |
| H | 8.04601 | -0.28410 | -2.47567 |
| H | 9.17051 | -1.36758 | -3.28343 |
| C | 5.77317 | -1.94019 | -2.73685 |
| H | 5.46319 | -0.91411 | -2.49992 |
| H | 4.97182 | -2.39059 | -3.32932 |

**Appendix 5 Optimized Cartesian Coordinates for [Co$^{III}$ (Corrole$^{•2-}$) Py$_2$]$^+$ (S=1/2) of 4.**

| | | | |
|---|---|---|---|
| Co | 0.00324 | -0.47690 | 0.14950 |
| F | 7.67154 | -1.69787 | 2.09789 |
| F | 4.99008 | -1.41896 | 2.26396 |
| F | -4.64302 | -1.99324 | -2.19743 |
| F | 8.93898 | -1.32254 | -0.28411 |
| F | -7.32256 | -2.29740 | -2.35774 |
| F | 4.82462 | -0.40194 | -2.35870 |
| F | -8.92127 | -1.40312 | -0.34185 |
| F | 7.50513 | -0.67129 | -2.50575 |
| N | -1.40765 | 0.78766 | -0.03031 |
| N | 1.40918 | 0.79288 | -0.03792 |
| F | -5.14679 | 0.10005 | 2.02018 |
| N | -1.22201 | -1.88436 | 0.36082 |
| N | 0.00852 | -0.75662 | -1.87816 |
| N | 1.23303 | -1.87961 | 0.37755 |
| N | -0.00410 | -0.18058 | 2.17120 |
| F | -7.82290 | -0.20410 | 1.84280 |
| C | 3.47987 | 1.76535 | -0.15039 |
| H | 4.55340 | 1.86763 | -0.17520 |
| C | 2.76288 | 0.50091 | -0.06755 |
| C | 2.56257 | 2.76125 | -0.14667 |
| H | 2.74810 | 3.82249 | -0.17602 |
| C | 1.24190 | 2.14357 | -0.08986 |

| | | | |
|---|---|---|---|
| C | -0.00219 | 2.81309 | -0.11661 |
| C | 0.46448 | 4.99429 | -1.30018 |
| C | 2.56384 | -1.94401 | 0.28410 |
| C | 3.34020 | -0.76707 | 0.04552 |
| C | -0.01317 | 4.30722 | -0.17660 |
| C | -0.50653 | 5.06023 | 0.89612 |
| H | -0.87104 | 4.55270 | 1.78241 |
| C | -1.24173 | 2.13568 | -0.12688 |
| C | -2.55252 | -1.95002 | 0.26716 |
| C | 0.70464 | -3.13545 | 0.62446 |
| C | -3.33054 | -0.78052 | 0.00465 |
| C | 5.58678 | -1.23670 | 1.07468 |
| C | 6.96489 | -1.38240 | 1.00674 |
| C | 5.50198 | -0.71806 | -1.24461 |
| C | -2.75692 | 0.48748 | -0.12343 |
| C | -0.69331 | -3.13632 | 0.62308 |
| C | -6.78059 | -1.70909 | -1.28543 |
| C | 0.00874 | 0.18659 | -4.08231 |
| H | 0.00470 | 1.08091 | -4.69125 |
| C | 6.88036 | -0.85365 | -1.33689 |
| C | 7.61330 | -1.18837 | -0.20606 |
| C | -5.40604 | -1.54411 | -1.18908 |
| C | -2.93892 | -3.33463 | 0.50268 |
| H | -3.95063 | -3.71004 | 0.50700 |
| C | 4.81930 | -0.90250 | -0.04163 |

| | | | |
|---|---|---|---|
| C | 0.00468 | 0.30826 | -2.70023 |
| H | -0.00184 | 1.28334 | -2.24112 |
| C | 1.14674 | -0.10484 | 2.86279 |
| H | 2.06402 | -0.20128 | 2.30464 |
| C | 0.44943 | 6.38563 | -1.34554 |
| H | 0.82528 | 6.89170 | -2.22887 |
| C | -3.46988 | 1.74186 | -0.31949 |
| H | -4.53653 | 1.83410 | -0.45105 |
| C | -0.04481 | 7.14091 | -0.27581 |
| C | -7.59764 | -1.25239 | -0.25946 |
| C | 2.94955 | -3.33255 | 0.49337 |
| H | 3.95932 | -3.71235 | 0.47404 |
| C | 1.80434 | -4.05727 | 0.70701 |
| H | 1.72820 | -5.11744 | 0.89219 |
| C | -2.55655 | 2.74138 | -0.31129 |
| H | -2.73813 | 3.79677 | -0.43364 |
| C | -0.52014 | 6.45199 | 0.84511 |
| H | -0.90035 | 7.01044 | 1.69426 |
| C | -1.16091 | -0.07071 | 2.84838 |
| H | -2.07352 | -0.13643 | 2.27829 |
| C | 1.18106 | 0.08679 | 4.23672 |
| H | 2.13801 | 0.14049 | 4.73870 |
| C | -4.80894 | -0.92951 | -0.08829 |
| C | -0.01559 | 0.20357 | 4.93466 |
| H | -0.02007 | 0.35351 | 6.00724 |

| | | | |
|---|---|---|---|
| C | 0.01757 | -1.98243 | -2.43117 |
| H | 0.02073 | -2.82617 | -1.76011 |
| C | -1.20643 | 0.12116 | 4.22192 |
| H | -2.16753 | 0.20276 | 4.71210 |
| C | 0.01823 | -1.08186 | -4.65134 |
| H | 0.02193 | -1.20806 | -5.72695 |
| C | -1.79344 | -4.05701 | 0.72142 |
| H | -1.71711 | -5.11389 | 0.92441 |
| C | -5.65787 | -0.48865 | 0.92702 |
| C | -7.03570 | -0.63870 | 0.85225 |
| C | 0.02294 | -2.18386 | -3.80429 |
| H | 0.03046 | -3.19481 | -4.18966 |
| H | 0.84445 | 4.43549 | -2.14824 |
| C | -0.08658 | 8.64807 | -0.34152 |
| H | 0.75713 | 9.04516 | -0.91035 |
| H | -1.00242 | 8.99144 | -0.83473 |
| H | -0.06712 | 9.09029 | 0.65663 |

**Appendix 6 Optimized Cartesian Coordinates for $[Co^{III}(Corrole^{1-})Py_2]^{2+}$ (S=0) of 4.**

| | | | |
|---|---|---|---|
| Co | 0.00413 | -0.47315 | 0.22352 |
| F | 7.76106 | -1.76362 | 1.91632 |
| F | 5.08734 | -1.56898 | 2.21315 |
| F | -4.54247 | -2.19618 | -2.09756 |
| F | 8.90975 | -1.23822 | -0.49265 |
| F | -7.21784 | -2.45542 | -2.36687 |
| F | 4.69065 | -0.30140 | -2.33207 |
| F | -8.88629 | -1.36991 | -0.51184 |
| F | 7.37097 | -0.50838 | -2.61407 |
| N | -1.40932 | 0.78479 | -0.00469 |
| N | 1.41739 | 0.78322 | -0.04496 |
| F | -5.19423 | 0.25641 | 1.89720 |
| N | -1.21790 | -1.87654 | 0.50660 |
| N | -0.00675 | -0.84561 | -1.79205 |
| N | 1.22947 | -1.87723 | 0.51563 |
| N | 0.00882 | -0.08093 | 2.21904 |
| F | -7.87003 | -0.01857 | 1.61996 |
| C | 3.48349 | 1.72611 | -0.06983 |
| H | 4.55711 | 1.81851 | -0.04864 |
| C | 2.74789 | 0.50212 | -0.03878 |
| C | 2.56848 | 2.74866 | -0.07737 |
| H | 2.78269 | 3.80337 | -0.05979 |
| C | 1.26631 | 2.15171 | -0.08994 |

| | | | |
|---|---|---|---|
| C | 0.00860 | 2.81129 | -0.14585 |
| C | 0.74563 | 4.97046 | -1.19017 |
| C | 2.60162 | -1.95507 | 0.37795 |
| C | 3.33509 | -0.82370 | 0.08903 |
| C | -0.01281 | 4.27765 | -0.22551 |
| C | -0.80322 | 5.02687 | 0.66863 |
| H | -1.37293 | 4.51899 | 1.43655 |
| C | -1.25423 | 2.13448 | -0.16531 |
| C | -2.58858 | -1.94659 | 0.39411 |
| C | 0.73708 | -3.08130 | 0.78903 |
| C | -3.32001 | -0.82034 | 0.05703 |
| C | 5.62463 | -1.31323 | 1.01344 |
| C | 7.00169 | -1.41218 | 0.87689 |
| C | 5.43030 | -0.65845 | -1.27483 |
| C | -2.73861 | 0.48715 | -0.13268 |
| C | -0.72899 | -3.08048 | 0.80002 |
| C | -6.71554 | -1.79373 | -1.32227 |
| C | -0.00409 | 0.00717 | -4.03292 |
| H | -0.00036 | 0.87717 | -4.67570 |
| C | 6.80344 | -0.76224 | -1.43348 |
| C | 7.59051 | -1.13893 | -0.35140 |
| C | -5.34315 | -1.65501 | -1.17110 |
| C | -2.96333 | -3.31489 | 0.67800 |
| H | -3.97106 | -3.69906 | 0.69505 |
| C | 4.80886 | -0.93388 | -0.05456 |

| | | | |
|---|---|---|---|
| C | -0.00136 | 0.18503 | -2.65812 |
| H | 0.00575 | 1.17927 | -2.24497 |
| C | 1.16157 | 0.03152 | 2.90422 |
| H | 2.08009 | -0.08099 | 2.35326 |
| C | 0.70125 | 6.35366 | -1.25905 |
| H | 1.27010 | 6.86373 | -2.02829 |
| C | -3.45176 | 1.68889 | -0.42356 |
| H | -4.51121 | 1.76764 | -0.60525 |
| C | -0.06667 | 7.10322 | -0.35563 |
| C | -7.56861 | -1.23730 | -0.37764 |
| C | 2.96515 | -3.34247 | 0.59941 |
| H | 3.96497 | -3.74455 | 0.55913 |
| C | 1.81334 | -4.03554 | 0.86388 |
| H | 1.71196 | -5.08807 | 1.07458 |
| C | -2.53410 | 2.71066 | -0.44499 |
| H | -2.72636 | 3.74762 | -0.66177 |
| C | -0.80752 | 6.41196 | 0.61351 |
| H | -1.39409 | 6.96853 | 1.33556 |
| C | -1.14727 | 0.05102 | 2.89616 |
| H | -2.06314 | -0.04226 | 2.33732 |
| C | 1.19829 | 0.28197 | 4.26743 |
| H | 2.15703 | 0.36293 | 4.76170 |
| C | -4.79398 | -0.95740 | -0.09502 |
| C | 0.00345 | 0.42082 | 4.96374 |
| H | 0.00136 | 0.61616 | 6.02874 |

| | | | |
|---|---|---|---|
| C | -0.01351 | -2.09260 | -2.29607 |
| H | -0.01850 | -2.91516 | -1.60042 |
| C | -1.18875 | 0.30076 | 4.25922 |
| H | -2.14938 | 0.39696 | 4.74712 |
| C | -0.01046 | -1.28304 | -4.55005 |
| H | -0.01185 | -1.45309 | -5.61937 |
| C | -1.80934 | -4.01731 | 0.92834 |
| H | -1.71603 | -5.06356 | 1.17137 |
| C | -5.67447 | -0.40824 | 0.83736 |
| C | -7.04852 | -0.54356 | 0.70864 |
| C | -0.01482 | -2.34937 | -3.65906 |
| H | -0.01977 | -3.37570 | -4.00069 |
| H | 1.32824 | 4.41718 | -1.91550 |
| C | -0.11584 | 8.60393 | -0.44481 |
| H | 0.83601 | 9.01311 | -0.78906 |
| H | -0.88320 | 8.91652 | -1.16174 |
| H | -0.36402 | 9.05282 | 0.51828 |

**Appendix 7 Optimized Cartesian Coordinates for [Co$^{III}$ (Corrole$^{•4-}$) Py$_2$]$^{-}$ (S=1/2) of 4.**

| | | | |
|---|---|---|---|
| Co | 0.00171 | -0.50844 | 0.09347 |
| F | 7.64936 | -2.00769 | 2.06729 |
| F | 4.98306 | -1.74315 | 2.20334 |
| F | -4.77025 | -2.15003 | -2.11624 |
| F | 9.00201 | -1.25876 | -0.18298 |
| F | -7.43955 | -2.42985 | -2.17718 |
| F | 4.95277 | -0.05284 | -2.24097 |
| F | -8.99136 | -1.32096 | -0.22431 |
| F | 7.62656 | -0.26557 | -2.32211 |
| N | -1.42688 | 0.78074 | -0.02265 |
| N | 1.42774 | 0.78263 | -0.03813 |
| F | -5.14715 | 0.28959 | 1.94609 |
| N | -1.23919 | -1.93497 | 0.22136 |
| N | 0.01510 | -0.68922 | -1.93699 |
| N | 1.24408 | -1.93063 | 0.26616 |
| N | -0.01226 | -0.31608 | 2.11751 |
| F | -7.81742 | 0.04986 | 1.82413 |
| C | 3.48201 | 1.76027 | -0.04199 |
| H | 4.55559 | 1.87517 | -0.02333 |
| C | 2.77896 | 0.50320 | -0.03980 |
| C | 2.54807 | 2.76618 | -0.03430 |
| H | 2.73881 | 3.82811 | -0.02242 |
| C | 1.25056 | 2.15627 | -0.04111 |

| | | | |
|---|---|---|---|
| C | -0.00021 | 2.81198 | -0.06910 |
| C | 0.50353 | 5.02112 | -1.19335 |
| C | 2.60184 | -1.98553 | 0.19463 |
| C | 3.36973 | -0.78274 | 0.03560 |
| C | -0.00760 | 4.30761 | -0.09986 |
| C | -0.52829 | 5.05027 | 0.96893 |
| H | -0.92210 | 4.52751 | 1.83364 |
| C | -1.24833 | 2.15163 | -0.09455 |
| C | -2.59650 | -1.98770 | 0.15310 |
| C | 0.72225 | -3.19157 | 0.42887 |
| C | -3.36378 | -0.78804 | -0.02957 |
| C | 5.59388 | -1.39648 | 1.05433 |
| C | 6.97397 | -1.53111 | 1.00817 |
| C | 5.58254 | -0.52062 | -1.14640 |
| C | -2.77530 | 0.49690 | -0.11145 |
| C | -0.72091 | -3.19134 | 0.42201 |
| C | -6.86380 | -1.76026 | -1.16488 |
| C | -0.00660 | 0.35068 | -4.09610 |
| H | -0.03578 | 1.26980 | -4.66710 |
| C | 6.96431 | -0.62880 | -1.21101 |
| C | 7.66715 | -1.14229 | -0.12988 |
| C | -5.48541 | -1.61021 | -1.11194 |
| C | -2.97465 | -3.34664 | 0.33217 |
| H | -3.98553 | -3.72817 | 0.34542 |
| C | 4.84296 | -0.89350 | -0.01628 |

| | | | |
|---|---|---|---|
| C | -0.01791 | 0.41052 | -2.70845 |
| H | -0.05541 | 1.35885 | -2.19514 |
| C | 1.13655 | -0.27857 | 2.81288 |
| H | 2.04966 | -0.35339 | 2.24199 |
| C | 0.49425 | 6.41407 | -1.21551 |
| H | 0.89703 | 6.93480 | -2.07899 |
| C | -3.47125 | 1.74745 | -0.27708 |
| H | -4.53835 | 1.85715 | -0.40040 |
| C | -0.02844 | 7.15307 | -0.14825 |
| C | -7.65768 | -1.19107 | -0.17861 |
| C | 2.97461 | -3.35222 | 0.31615 |
| H | 3.98286 | -3.74008 | 0.29675 |
| C | 1.79177 | -4.10446 | 0.46593 |
| H | 1.71758 | -5.17685 | 0.57931 |
| C | -2.53918 | 2.75446 | -0.25968 |
| H | -2.72442 | 3.81246 | -0.36353 |
| C | -0.53727 | 6.44355 | 0.94485 |
| H | -0.93996 | 6.98761 | 1.79380 |
| C | -1.17157 | -0.23999 | 2.79224 |
| H | -2.07607 | -0.27780 | 2.20448 |
| C | 1.16641 | -0.15579 | 4.19646 |
| H | 2.12092 | -0.12912 | 4.70622 |
| C | -4.83689 | -0.91037 | -0.08672 |
| C | -0.03298 | -0.07250 | 4.89546 |
| H | -0.04104 | 0.02324 | 5.97469 |

| | | | |
|---|---|---|---|
| C | 0.06171 | -1.88835 | -2.54057 |
| H | 0.08750 | -2.75222 | -1.89398 |
| C | -1.22187 | -0.11812 | 4.17528 |
| H | -2.18395 | -0.06093 | 4.66803 |
| C | 0.04258 | -0.89102 | -4.72079 |
| H | 0.05340 | -0.96934 | -5.80137 |
| C | -1.79337 | -4.09878 | 0.49545 |
| H | -1.72234 | -5.16686 | 0.64558 |
| C | -5.67516 | -0.36558 | 0.89410 |
| C | -7.05699 | -0.48629 | 0.85512 |
| C | 0.07747 | -2.02912 | -3.92280 |
| H | 0.11632 | -3.02101 | -4.35438 |
| H | 0.91050 | 4.47605 | -2.03784 |
| C | -0.06541 | 8.66226 | -0.18758 |
| H | 0.78738 | 9.06777 | -0.73721 |
| H | -0.97312 | 9.02103 | -0.68552 |
| H | -0.05679 | 9.08691 | 0.81878 |

**Appendix 8 Optimized Cartesian Coordinates for [Co$^{II}$ (Corrole$^{3-}$) Py]$^{-}$ (S=1/2) of 4.**

| | | | |
|---|---|---|---|
| Co | 0.00376 | -0.53763 | -0.22358 |
| F | -7.51136 | -1.86892 | -2.77171 |
| F | -4.83119 | -1.62196 | -2.74127 |
| F | 4.89729 | -1.80433 | 1.78220 |
| F | -8.96817 | -1.31851 | -0.53367 |
| F | 7.57681 | -2.04822 | 1.72588 |
| F | -5.02585 | -0.30107 | 1.81185 |
| F | 8.98144 | -1.27788 | -0.48082 |
| F | -7.70676 | -0.52909 | 1.75122 |
| N | 1.41456 | 0.75639 | -0.31441 |
| N | -1.41958 | 0.74071 | -0.29256 |
| F | 4.98593 | -0.04087 | -2.62200 |
| N | 1.24349 | -1.95768 | -0.39973 |
| N | 0.00294 | -0.64404 | 2.17135 |
| N | -1.22011 | -1.96939 | -0.42441 |
| F | 7.66764 | -0.26807 | -2.64598 |
| C | -3.48801 | 1.70767 | -0.41171 |
| H | -4.55995 | 1.81314 | -0.49187 |
| C | -2.76988 | 0.45632 | -0.36943 |
| C | -2.56679 | 2.71595 | -0.36322 |
| H | -2.76151 | 3.77661 | -0.39440 |
| C | -1.25931 | 2.10385 | -0.28398 |
| C | -0.01248 | 2.77073 | -0.24261 |

| | | | |
|---|---|---|---|
| C | -0.53622 | 4.95269 | 0.91438 |
| C | -2.57674 | -2.02584 | -0.47357 |
| C | -3.33965 | -0.83556 | -0.43067 |
| C | -0.02372 | 4.26628 | -0.19549 |
| C | 0.46943 | 5.03460 | -1.25933 |
| H | 0.85921 | 4.53385 | -2.13876 |
| C | 1.24159 | 2.11716 | -0.26150 |
| C | 2.60054 | -1.99857 | -0.44914 |
| C | -0.69610 | -3.22544 | -0.52649 |
| C | 3.35212 | -0.80116 | -0.39455 |
| C | -5.50821 | -1.35577 | -1.61135 |
| C | -6.89030 | -1.48469 | -1.64691 |
| C | -5.60692 | -0.68161 | 0.66010 |
| C | 2.76958 | 0.48475 | -0.33121 |
| C | 0.73345 | -3.21808 | -0.52042 |
| C | 6.93019 | -1.55547 | 0.65928 |
| C | 1.18739 | -0.90671 | 4.24402 |
| H | 2.13507 | -1.05750 | 4.74648 |
| C | -6.99036 | -0.79616 | 0.64941 |
| C | -7.63526 | -1.20141 | -0.51082 |
| C | 5.54800 | -1.42578 | 0.66775 |
| C | 2.98854 | -3.37753 | -0.59727 |
| H | 4.00005 | -3.74889 | -0.67755 |
| C | -4.82409 | -0.95301 | -0.46335 |
| C | 1.13993 | -0.82963 | 2.85449 |

| | | | |
|---|---|---|---|
| H | 2.04272 | -0.92181 | 2.26072 |
| C | -0.55197 | 6.34495 | 0.95863 |
| H | -0.95533 | 6.84448 | 1.83418 |
| C | 3.47742 | 1.74092 | -0.26542 |
| H | 4.55114 | 1.85639 | -0.24851 |
| C | -0.05448 | 7.10984 | -0.10216 |
| C | 7.64848 | -1.16081 | -0.46074 |
| C | -2.94941 | -3.41176 | -0.59506 |
| H | -3.95676 | -3.79761 | -0.65624 |
| C | -1.77693 | -4.15287 | -0.63253 |
| H | -1.69416 | -5.22621 | -0.72801 |
| C | 2.54605 | 2.73977 | -0.21934 |
| H | 2.73356 | 3.80061 | -0.16315 |
| C | 0.45372 | 6.42694 | -1.21259 |
| H | 0.83569 | 6.99148 | -2.05767 |
| C | 4.83708 | -0.91111 | -0.41778 |
| C | -1.13578 | -0.53090 | 2.86732 |
| H | -2.03767 | -0.38303 | 2.28344 |
| C | -0.00018 | -0.78734 | 4.96082 |
| H | -0.00135 | -0.84279 | 6.04330 |
| C | 1.82436 | -4.13141 | -0.64248 |
| H | 1.75326 | -5.20374 | -0.75689 |
| C | 5.59337 | -0.53008 | -1.52755 |
| C | 6.97684 | -0.64306 | -1.55933 |
| C | -1.18634 | -0.59623 | 4.25730 |

| | | | |
|---|---|---|---|
| H | -2.13536 | -0.49932 | 4.77040 |
| H | -0.92380 | 4.38669 | 1.75436 |
| C | -0.04314 | 8.61854 | -0.03969 |
| H | -0.87453 | 8.99892 | 0.55827 |
| H | 0.88204 | 8.98625 | 0.41788 |
| H | -0.10955 | 9.05876 | -1.03724 |

**Appendix 9 Optimized Cartesian Coordinates of 3a.**

**ΔGsoln= -3911.9427**

| | | | |
|---|---|---|---|
| Co | -1.39360 | 1.18324 | -0.05317 |
| F | 4.71069 | 6.31621 | -0.86214 |
| F | 2.30454 | 5.12157 | -1.09605 |
| F | -5.68217 | -1.07893 | 2.55296 |
| F | 6.65860 | 5.18618 | 0.67243 |
| F | -8.21530 | -1.98701 | 2.70657 |
| F | 3.76793 | 1.65017 | 1.78185 |
| F | -9.82962 | -1.92514 | 0.51180 |
| F | 6.17519 | 2.84713 | 1.98508 |
| N | -2.26242 | -0.37386 | -0.74591 |
| N | 0.35501 | 0.75925 | -0.70640 |
| F | -6.36226 | -0.00606 | -2.01429 |
| O | 0.15321 | -2.52454 | 0.95563 |
| N | -2.98425 | 1.74461 | 0.78978 |
| N | -0.71254 | 2.70569 | 0.83266 |
| N | -1.83005 | 2.24513 | -1.66650 |
| N | 2.33847 | -3.11266 | 1.05785 |
| F | -8.88748 | -0.93463 | -1.84597 |
| C | 2.57080 | 0.82486 | -1.23406 |
| H | 3.58443 | 1.18433 | -1.32663 |
| C | 1.50842 | 1.50268 | -0.53801 |
| C | 2.05285 | -0.32999 | -1.75653 |

| | | | |
|---|---|---|---|
| H | 2.55917 | -1.05675 | -2.37281 |
| C | 0.65741 | -0.37869 | -1.40339 |
| C | -0.23677 | -1.43521 | -1.65913 |
| C | 1.04281 | -3.51067 | -1.02884 |
| C | 0.52404 | 3.27186 | 0.89185 |
| C | 1.14479 | -2.99638 | 0.38957 |
| C | 1.61902 | 2.68843 | 0.21817 |
| C | 0.35428 | -2.76236 | -2.01494 |
| C | 0.21139 | -3.30772 | -3.29271 |
| H | -0.29622 | -2.72668 | -4.05397 |
| C | -1.61550 | -1.40504 | -1.37533 |
| C | -4.23786 | 1.22687 | 0.83239 |
| C | -1.60966 | 3.44882 | 1.54884 |
| C | -4.54014 | 0.01322 | 0.17670 |
| C | 3.23669 | 4.54097 | -0.32161 |
| C | 4.47044 | 5.16848 | -0.21466 |
| C | 3.97352 | 2.79625 | 1.11000 |
| C | -3.58061 | -0.73383 | -0.53629 |
| C | -2.91842 | 2.89431 | 1.52220 |
| C | -7.75177 | -1.49677 | 1.54912 |
| C | 5.21772 | 3.40005 | 1.22881 |
| C | 5.46553 | 4.59344 | 0.56398 |
| C | -6.45041 | -1.02258 | 1.45323 |
| C | -5.03606 | 2.11760 | 1.63174 |
| H | -6.08542 | 1.99505 | 1.85513 |

| | | | |
|---|---|---|---|
| C | 2.95173 | 3.34208 | 0.33214 |
| C | -0.93879 | 3.10642 | -2.18935 |
| H | 0.01524 | 3.18746 | -1.69406 |
| C | 2.36254 | -2.82912 | 2.52472 |
| H | 1.55047 | -2.11690 | 2.67369 |
| C | 1.51397 | -4.79159 | -1.33987 |
| H | 2.00736 | -5.38370 | -0.57962 |
| C | -3.77315 | -2.04114 | -1.11017 |
| H | -4.70234 | -2.59047 | -1.10915 |
| C | 1.34101 | -5.32615 | -2.61442 |
| H | 1.70552 | -6.32274 | -2.83360 |
| C | -8.57531 | -1.46970 | 0.43198 |
| C | 0.40977 | 4.44859 | 1.70884 |
| H | 1.21752 | 5.11823 | 1.96422 |
| C | -0.91180 | 4.55581 | 2.11206 |
| H | -1.33771 | 5.32493 | 2.73900 |
| C | -2.57126 | -2.45993 | -1.60530 |
| H | -2.35644 | -3.40381 | -2.08056 |
| C | 0.70025 | -4.57766 | -3.59678 |
| H | 0.57204 | -4.98085 | -4.59473 |
| C | -3.03272 | 2.12852 | -2.25848 |
| H | -3.73063 | 1.43441 | -1.81916 |
| C | -1.21569 | 3.86829 | -3.31584 |
| H | -0.46007 | 4.54492 | -3.69267 |
| C | -5.93149 | -0.50947 | 0.26404 |

| | | | |
|---|---|---|---|
| C | 3.67040 | -2.15581 | 2.96252 |
| H | 3.83406 | -1.24490 | 2.37943 |
| H | 4.51763 | -2.82382 | 2.77307 |
| C | -2.45614 | 3.74570 | -3.93124 |
| H | -2.69911 | 4.32801 | -4.81161 |
| C | -3.37779 | 2.85836 | -3.38716 |
| H | -4.35936 | 2.72303 | -3.82190 |
| C | -4.21467 | 3.14721 | 2.06040 |
| H | -4.49270 | 3.98077 | 2.68803 |
| C | -6.78835 | -0.49543 | -0.83707 |
| C | 2.06115 | -4.08596 | 3.35724 |
| H | 1.10483 | -4.51012 | 3.03749 |
| H | 2.83306 | -4.83500 | 3.15875 |
| C | -8.09182 | -0.96748 | -0.76855 |
| C | 3.62224 | -1.82297 | 4.46283 |
| H | 2.84881 | -1.06529 | 4.64019 |
| H | 4.57327 | -1.37537 | 4.76639 |
| C | 2.02375 | -3.74910 | 4.85563 |
| H | 1.17380 | -3.08487 | 5.05755 |
| H | 1.85005 | -4.66208 | 5.43332 |
| C | 3.31974 | -3.06534 | 5.31198 |
| H | 4.15290 | -3.77416 | 5.22447 |
| H | 3.25076 | -2.79032 | 6.36910 |
| O | 4.02625 | -4.70470 | 0.85840 |
| C | 3.56624 | -3.63728 | 0.48490 |

| | | | |
|---|---|---|---|
| N | 4.13643 | -2.81477 | -0.41225 |
| H | 3.70843 | -1.91096 | -0.56487 |
| C | 6.56617 | -2.33131 | -0.51636 |
| H | 6.66122 | -2.56000 | 0.54904 |
| H | 6.34291 | -1.25997 | -0.59607 |
| C | 6.56620 | -3.19812 | -3.35075 |
| H | 6.77473 | -4.27234 | -3.27076 |
| H | 6.45239 | -2.98071 | -4.41692 |
| C | 5.39016 | -3.12795 | -1.10735 |
| H | 5.56461 | -4.19193 | -0.93208 |
| C | 7.87672 | -2.64240 | -1.25444 |
| H | 8.68551 | -2.03163 | -0.84221 |
| H | 8.15199 | -3.68905 | -1.07347 |
| C | 7.74469 | -2.40785 | -2.76549 |
| H | 7.59142 | -1.33756 | -2.95365 |
| H | 8.67358 | -2.68587 | -3.27296 |
| C | 5.25321 | -2.88419 | -2.61769 |
| H | 4.98773 | -1.83309 | -2.78892 |
| H | 4.43522 | -3.49216 | -3.01433 |
| O | -0.95887 | 0.02826 | 1.60474 |
| H | -0.27526 | 0.48474 | 2.11240 |
| H | -0.56918 | -0.83327 | 1.34199 |

**Appendix 10 Optimized Cartesian Coordinates of 3b.**

**ΔGsoln= -3911.268 Hartree**

| | | | |
|---|---|---|---|
| Co | -1.26618 | 1.47324 | 0.18582 |
| F | 4.39585 | 7.06716 | -0.41770 |
| F | 2.12461 | 5.64882 | -0.74131 |
| F | -5.05311 | -2.56854 | 1.46282 |
| F | 6.34944 | 6.16172 | 1.25239 |
| F | -7.56705 | -3.52986 | 1.36623 |
| F | 3.74087 | 2.39618 | 2.30170 |
| F | -9.55427 | -2.06005 | 0.22048 |
| F | 6.01042 | 3.82099 | 2.60519 |
| N | -1.87965 | -0.36400 | -0.21287 |
| N | 0.55007 | 1.16200 | -0.36419 |
| F | -6.49172 | 1.36074 | -0.76963 |
| O | -0.08866 | -3.32868 | 0.28196 |
| N | -2.89703 | 1.82080 | 1.09685 |
| N | -0.75294 | 3.04426 | 1.10199 |
| N | -1.92906 | 2.40571 | -1.80468 |
| N | 2.06055 | -4.02108 | 0.44706 |
| F | -9.00121 | 0.38777 | -0.84094 |
| C | 2.79075 | 1.39173 | -0.73791 |
| H | 3.78417 | 1.81363 | -0.74323 |
| C | 1.62969 | 1.98642 | -0.13079 |
| C | 2.39600 | 0.20967 | -1.30841 |

| | | | |
|---|---|---|---|
| H | 3.00660 | -0.48247 | -1.86592 |
| C | 0.98493 | 0.07350 | -1.06391 |
| C | 0.14373 | -0.97362 | -1.53442 |
| C | 1.17142 | -3.28552 | -1.74836 |
| C | 0.43232 | 3.69807 | 1.21990 |
| C | 0.99976 | -3.52582 | -0.26695 |
| C | 1.59089 | 3.20080 | 0.60127 |
| C | 0.75578 | -2.07511 | -2.34781 |
| C | 0.90329 | -1.92083 | -3.72829 |
| H | 0.60361 | -0.98409 | -4.18370 |
| C | -1.21963 | -1.07984 | -1.30516 |
| C | -4.07095 | 1.15374 | 1.19158 |
| C | -1.68819 | 3.63366 | 1.88814 |
| C | -4.29826 | -0.02096 | 0.41727 |
| C | 3.06358 | 5.17589 | 0.09357 |
| C | 4.22709 | 5.91741 | 0.24788 |
| C | 3.87760 | 3.54025 | 1.61274 |
| C | -3.32242 | -0.65480 | -0.34235 |
| C | -2.92811 | 2.92149 | 1.89883 |
| C | -7.29348 | -2.32799 | 0.84383 |
| C | 5.05083 | 4.26264 | 1.78201 |
| C | 5.22485 | 5.45720 | 1.09623 |
| C | -6.00278 | -1.81864 | 0.88396 |
| C | -4.91830 | 1.87626 | 2.10066 |
| H | -5.91895 | 1.59731 | 2.39504 |

| | | | |
|---|---|---|---|
| C | 2.85657 | 3.97193 | 0.76639 |
| C | -1.01708 | 2.76912 | -2.71916 |
| H | 0.01457 | 2.54512 | -2.47685 |
| C | 1.81789 | -4.48751 | 1.84608 |
| H | 0.98001 | -3.87915 | 2.18847 |
| C | 1.66526 | -4.31774 | -2.55543 |
| H | 1.96170 | -5.25617 | -2.10428 |
| C | -3.44265 | -1.61447 | -1.36574 |
| H | -4.37942 | -2.03106 | -1.70514 |
| C | 1.77403 | -4.15748 | -3.93416 |
| H | 2.14716 | -4.97288 | -4.54233 |
| C | -8.30879 | -1.57968 | 0.26389 |
| C | 0.22308 | 4.81375 | 2.11635 |
| H | 0.97065 | 5.53288 | 2.41660 |
| C | -1.09149 | 4.77041 | 2.53016 |
| H | -1.57981 | 5.44430 | 3.21792 |
| C | -2.20282 | -1.87244 | -1.92739 |
| H | -2.01627 | -2.52998 | -2.76250 |
| C | 1.40546 | -2.95118 | -4.52129 |
| H | 1.49663 | -2.81268 | -5.59227 |
| C | -3.21798 | 2.66903 | -2.06823 |
| H | -3.93268 | 2.36943 | -1.31177 |
| C | -1.34915 | 3.39857 | -3.91420 |
| H | -0.56989 | 3.67124 | -4.61486 |
| C | -5.68272 | -0.56634 | 0.35636 |

| | | | |
|---|---|---|---|
| C | 3.01641 | -4.20970 | 2.76275 |
| H | 3.26724 | -3.14530 | 2.73485 |
| H | 3.89382 | -4.76299 | 2.41054 |
| C | -2.68890 | 3.66529 | -4.18134 |
| H | -2.98345 | 4.15383 | -5.10266 |
| C | -3.64142 | 3.29184 | -3.23793 |
| H | -4.69626 | 3.47673 | -3.39815 |
| C | -4.20113 | 2.97746 | 2.54133 |
| H | -4.53380 | 3.73023 | 3.24043 |
| C | -6.72711 | 0.15715 | -0.22188 |
| C | 1.39488 | -5.96490 | 1.89311 |
| H | 0.51792 | -6.11035 | 1.25556 |
| H | 2.20256 | -6.57726 | 1.48211 |
| C | -8.02527 | -0.33075 | -0.27219 |
| C | 2.69698 | -4.64274 | 4.20302 |
| H | 1.89177 | -4.01192 | 4.59958 |
| H | 3.57139 | -4.46918 | 4.83720 |
| C | 1.08615 | -6.39527 | 3.33523 |
| H | 0.20290 | -5.85250 | 3.69515 |
| H | 0.82796 | -7.45848 | 3.35386 |
| C | 2.26891 | -6.11509 | 4.27239 |
| H | 3.11584 | -6.75153 | 3.98626 |
| H | 2.00826 | -6.38251 | 5.30118 |
| O | 3.77376 | -5.44289 | -0.23321 |
| C | 3.37499 | -4.29641 | -0.10876 |

| | | | |
|---|---|---|---|
| N | 4.08458 | -3.19081 | -0.39734 |
| H | 3.69126 | -2.29347 | -0.15073 |
| C | 6.49103 | -2.91444 | 0.13813 |
| H | 6.39393 | -3.63026 | 0.95941 |
| H | 6.28005 | -1.92015 | 0.55151 |
| C | 7.00637 | -2.31865 | -2.71933 |
| H | 7.20338 | -3.31308 | -3.13893 |
| H | 7.08445 | -1.60891 | -3.54824 |
| C | 5.44601 | -3.23331 | -0.94484 |
| H | 5.59255 | -4.26195 | -1.28164 |
| C | 7.91484 | -2.94248 | -0.43756 |
| H | 8.63268 | -2.67185 | 0.34253 |
| H | 8.15876 | -3.96711 | -0.74515 |
| C | 8.05584 | -2.00457 | -1.64429 |
| H | 7.93314 | -0.96597 | -1.31176 |
| H | 9.06242 | -2.08211 | -2.06670 |
| C | 5.58134 | -2.28702 | -2.14656 |
| H | 5.34532 | -1.26489 | -1.82442 |
| H | 4.85143 | -2.56153 | -2.91337 |
| O | -1.42992 | -0.97839 | 1.04853 |
| H | -1.05776 | -1.85123 | 0.78958 |

**Appendix 11 Optimized Cartesian Coordinates of 3c.**

**ΔGsoln= -3910.65179 Hartree**

| | | | |
|---|---|---|---|
| Co | -1.50854 | 1.32590 | 0.10995 |
| F | 4.31786 | 6.80279 | -0.44425 |
| F | 2.00934 | 5.46466 | -0.84277 |
| F | -5.69113 | -1.09019 | 2.65949 |
| F | 6.16198 | 5.85394 | 1.32185 |
| F | -8.12700 | -2.24527 | 2.77833 |
| F | 3.37122 | 2.20540 | 2.31350 |
| F | -9.62345 | -2.51810 | 0.51913 |
| F | 5.67948 | 3.55179 | 2.69466 |
| N | -2.20144 | -0.34880 | -0.52341 |
| N | 0.30915 | 0.97917 | -0.37743 |
| F | -6.23616 | -0.45337 | -2.00197 |
| O | 0.43152 | -3.10417 | 0.76061 |
| N | -3.20924 | 1.84969 | 0.73299 |
| N | -1.04840 | 3.02361 | 0.80320 |
| N | -1.88729 | 2.19766 | -1.76395 |
| N | 2.62852 | -3.68323 | 0.71346 |
| F | -8.66847 | -1.61892 | -1.86828 |
| C | 2.53957 | 1.20567 | -0.76376 |
| H | 3.52771 | 1.63792 | -0.78685 |
| C | 1.37883 | 1.80387 | -0.16401 |
| C | 2.14412 | 0.01607 | -1.31777 |

| | | | |
|---|---|---|---|
| H | 2.74554 | -0.67460 | -1.88615 |
| C | 0.74075 | -0.13235 | -1.05077 |
| C | -0.07422 | -1.23729 | -1.38202 |
| C | 1.34661 | -3.36535 | -1.40527 |
| C | 0.15453 | 3.63927 | 0.99469 |
| C | 1.43551 | -3.34963 | 0.10113 |
| C | 1.33354 | 3.06111 | 0.51971 |
| C | 0.57369 | -2.39517 | -2.07974 |
| C | 0.40026 | -2.50850 | -3.46248 |
| H | -0.17863 | -1.75405 | -3.98215 |
| C | -1.46172 | -1.32975 | -1.11966 |
| C | -4.40968 | 1.20474 | 0.78114 |
| C | -2.02656 | 3.72254 | 1.40495 |
| C | -4.54675 | -0.08476 | 0.25128 |
| C | 2.89279 | 4.97562 | 0.04163 |
| C | 4.07709 | 5.67396 | 0.23237 |
| C | 3.58229 | 3.33275 | 1.61858 |
| C | -3.47993 | -0.81034 | -0.35651 |
| C | -3.29770 | 3.04477 | 1.34972 |
| C | -7.65621 | -1.80183 | 1.60716 |
| C | 4.77310 | 4.01442 | 1.82613 |
| C | 5.01989 | 5.19029 | 1.12954 |
| C | -6.40568 | -1.20345 | 1.53051 |
| C | -5.33594 | 2.08378 | 1.45185 |
| H | -6.37942 | 1.87884 | 1.63620 |

| | | | |
|---|---|---|---|
| C | 2.61402 | 3.79249 | 0.72562 |
| C | -1.03059 | 3.08395 | -2.29846 |
| H | -0.14342 | 3.30940 | -1.72628 |
| C | 2.60404 | -3.96930 | 2.17834 |
| H | 1.74379 | -3.40591 | 2.54192 |
| C | 1.87966 | -4.43856 | -2.12969 |
| H | 2.43642 | -5.20963 | -1.61310 |
| C | -3.55379 | -2.14589 | -0.88943 |
| H | -4.43210 | -2.77240 | -0.89391 |
| C | 1.68565 | -4.54427 | -3.50390 |
| H | 2.10075 | -5.38777 | -4.04250 |
| C | -8.41983 | -1.94401 | 0.45646 |
| C | -0.10128 | 4.84734 | 1.74740 |
| H | 0.64161 | 5.56555 | 2.05918 |
| C | -1.45433 | 4.89687 | 2.00048 |
| H | -1.98875 | 5.65857 | 2.54716 |
| C | -2.31066 | -2.46543 | -1.36108 |
| H | -1.99970 | -3.39442 | -1.81104 |
| C | 0.95288 | -3.57059 | -4.17475 |
| H | 0.80030 | -3.63857 | -5.24545 |
| C | -3.00373 | 1.90938 | -2.45369 |
| H | -3.68097 | 1.19676 | -2.00779 |
| C | -1.24984 | 3.70006 | -3.52353 |
| H | -0.52151 | 4.40517 | -3.90255 |
| C | -5.88547 | -0.73577 | 0.32447 |

| | | | |
|---|---|---|---|
| C | 3.85626 | -3.44224 | 2.89156 |
| H | 3.97347 | -2.37276 | 2.69277 |
| H | 4.74815 | -3.94743 | 2.50471 |
| C | -2.40616 | 3.39649 | -4.23399 |
| H | -2.60702 | 3.86110 | -5.19173 |
| C | -3.29898 | 2.48342 | -3.68341 |
| H | -4.21540 | 2.21188 | -4.19108 |
| C | -4.64248 | 3.22324 | 1.80768 |
| H | -5.02855 | 4.08462 | 2.33070 |
| C | -6.67803 | -0.89176 | -0.81211 |
| C | 2.36475 | -5.45917 | 2.47373 |
| H | 1.44415 | -5.78260 | 1.97847 |
| H | 3.18589 | -6.04169 | 2.04658 |
| C | -7.93020 | -1.48719 | -0.76024 |
| C | 3.75576 | -3.69360 | 4.40492 |
| H | 2.93170 | -3.09718 | 4.81585 |
| H | 4.66956 | -3.34348 | 4.89433 |
| C | 2.27346 | -5.70557 | 3.98748 |
| H | 1.37982 | -5.20675 | 4.38368 |
| H | 2.14453 | -6.77525 | 4.17927 |
| C | 3.51416 | -5.17631 | 4.71994 |
| H | 4.39187 | -5.75882 | 4.41215 |
| H | 3.40565 | -5.31836 | 5.79978 |
| O | 4.40200 | -5.02134 | 0.01762 |
| C | 3.87948 | -3.91706 | 0.02652 |

| | | | |
|---|---|---|---|
| N | 4.40594 | -2.81151 | -0.53583 |
| H | 3.96582 | -1.92470 | -0.33483 |
| C | 6.83093 | -2.29163 | -0.40887 |
| H | 6.92022 | -2.90912 | 0.48947 |
| H | 6.59216 | -1.27329 | -0.07714 |
| C | 6.86845 | -2.00475 | -3.35855 |
| H | 7.09364 | -3.02459 | -3.69466 |
| H | 6.75955 | -1.39543 | -4.26067 |
| C | 5.67304 | -2.81529 | -1.27614 |
| H | 5.86561 | -3.86254 | -1.51993 |
| C | 8.15247 | -2.28196 | -1.19180 |
| H | 8.94843 | -1.86745 | -0.56587 |
| H | 8.44220 | -3.31483 | -1.42262 |
| C | 8.02929 | -1.48645 | -2.49849 |
| H | 7.86037 | -0.42797 | -2.26302 |
| H | 8.96655 | -1.53721 | -3.06119 |
| C | 5.54462 | -2.01282 | -2.57908 |
| H | 5.26304 | -0.97963 | -2.33828 |
| H | 4.74101 | -2.43296 | -3.19058 |
| O | -1.18071 | 0.57268 | 1.73966 |

**Appendix 12 Optimized Cartesian Coordinates of 3d.**

**ΔGsoln= -3986.6283 Hartree**

| | | | |
|---|---|---|---|
| Co | 1.41101 | 1.27097 | -0.12004 |
| F | -4.46690 | 6.66892 | 0.56658 |
| F | -2.14695 | 5.34586 | 0.90704 |
| F | 5.76133 | -0.97947 | -2.56626 |
| F | -6.36523 | 5.69895 | -1.13319 |
| F | 8.24855 | -2.00884 | -2.63948 |
| F | -3.58653 | 2.06321 | -2.20054 |
| F | 9.71159 | -2.22994 | -0.34987 |
| F | -5.91100 | 3.38727 | -2.50474 |
| N | 2.16528 | -0.35564 | 0.56939 |
| N | -0.38488 | 0.89267 | 0.42137 |
| F | 6.18718 | -0.33543 | 2.11413 |
| O | -0.27981 | -3.35144 | -0.73333 |
| N | 3.10091 | 1.83662 | -0.74476 |
| N | 0.89476 | 2.94132 | -0.82886 |
| N | 1.78694 | 2.21274 | 1.74251 |
| N | -2.51823 | -3.72934 | -0.72494 |
| F | 8.66216 | -1.39203 | 2.02278 |
| C | -2.62249 | 1.05655 | 0.82182 |
| H | -3.62413 | 1.45831 | 0.85547 |
| C | -1.48506 | 1.69369 | 0.20473 |
| C | -2.18623 | -0.11587 | 1.37699 |

| | | | |
|---|---|---|---|
| H | -2.75900 | -0.81534 | 1.96480 |
| C | -0.77379 | -0.22730 | 1.10120 |
| C | 0.06905 | -1.30332 | 1.44888 |
| C | -1.26597 | -3.48876 | 1.42030 |
| C | -0.31163 | 3.54125 | -0.97721 |
| C | -1.31228 | -3.48988 | -0.08892 |
| C | -1.48461 | 2.93594 | -0.46919 |
| C | -0.55214 | -2.49192 | 2.12355 |
| C | -0.42873 | -2.62288 | 3.51158 |
| H | 0.10304 | -1.85243 | 4.05824 |
| C | 1.45189 | -1.34692 | 1.18466 |
| C | 4.32524 | 1.25259 | -0.74928 |
| C | 1.87331 | 3.68890 | -1.40813 |
| C | 4.51169 | -0.03208 | -0.18226 |
| C | -3.04835 | 4.84218 | 0.04567 |
| C | -4.24044 | 5.53552 | -0.11296 |
| C | -3.77452 | 3.19150 | -1.49626 |
| C | 3.47195 | -0.76968 | 0.42254 |
| C | 3.15253 | 3.06085 | -1.34585 |
| C | 7.73274 | -1.59426 | -1.47355 |
| C | -4.97783 | 3.86253 | -1.66761 |
| C | -5.21069 | 5.04200 | -0.97406 |
| C | 6.45329 | -1.05800 | -1.41747 |
| C | 5.23075 | 2.17138 | -1.38607 |
| H | 6.28759 | 2.01245 | -1.54329 |

| | | | |
|---|---|---|---|
| C | -2.77638 | 3.65525 | -0.63716 |
| C | 0.89725 | 3.06368 | 2.27801 |
| H | -0.00913 | 3.23115 | 1.71488 |
| C | -2.48675 | -3.99135 | -2.19304 |
| H | -1.60452 | -3.45010 | -2.53795 |
| C | -1.79574 | -4.58451 | 2.11422 |
| H | -2.31766 | -5.36147 | 1.56931 |
| C | 3.58275 | -2.09199 | 0.99563 |
| H | 4.48350 | -2.68666 | 1.02722 |
| C | -1.64553 | -4.70131 | 3.49315 |
| H | -2.05457 | -5.56181 | 4.00970 |
| C | 8.47886 | -1.71165 | -0.30917 |
| C | -0.08933 | 4.77274 | -1.69078 |
| H | -0.84852 | 5.48783 | -1.97211 |
| C | 1.26852 | 4.86160 | -1.95439 |
| H | 1.77611 | 5.65940 | -2.47690 |
| C | 2.34957 | -2.44469 | 1.46221 |
| H | 2.07077 | -3.37365 | 1.93484 |
| C | -0.96458 | -3.71205 | 4.19607 |
| H | -0.84496 | -3.78700 | 5.27095 |
| C | 2.92837 | 1.99030 | 2.41342 |
| H | 3.62759 | 1.30298 | 1.96037 |
| C | 1.10935 | 3.71202 | 3.48914 |
| H | 0.35513 | 4.38767 | 3.87212 |
| C | 5.87810 | -0.62128 | -0.22290 |

| | | | |
|---|---|---|---|
| C | -3.71303 | -3.40985 | -2.90929 |
| H | -3.79801 | -2.34165 | -2.68872 |
| H | -4.62518 | -3.89388 | -2.54298 |
| C | 2.29260 | 3.47755 | 4.18178 |
| H | 2.48860 | 3.96808 | 5.12785 |
| C | 3.21874 | 2.59940 | 3.62872 |
| H | 4.15670 | 2.38228 | 4.12379 |
| C | 4.49855 | 3.29119 | -1.75679 |
| H | 4.87209 | 4.17023 | -2.26161 |
| C | 6.66117 | -0.74906 | 0.92547 |
| C | -2.29144 | -5.48157 | -2.51802 |
| H | -1.38719 | -5.84376 | -2.01984 |
| H | -3.13573 | -6.04600 | -2.11235 |
| C | 7.94022 | -1.28785 | 0.89778 |
| C | -3.60293 | -3.63253 | -4.42634 |
| H | -2.75542 | -3.05448 | -4.81524 |
| H | -4.49918 | -3.24317 | -4.91878 |
| C | -2.19036 | -5.69951 | -4.03547 |
| H | -1.27685 | -5.22129 | -4.41129 |
| H | -2.09327 | -6.76854 | -4.24911 |
| C | -3.40493 | -5.11538 | -4.77017 |
| H | -4.30432 | -5.67570 | -4.48420 |
| H | -3.28875 | -5.23850 | -5.85164 |
| O | -4.34230 | -5.02452 | -0.08807 |
| C | -3.78514 | -3.93640 | -0.05943 |

| | | | |
|---|---|---|---|
| N | -4.29024 | -2.82727 | 0.51352 |
| H | -3.78952 | -1.95861 | 0.38257 |
| C | -6.60394 | -1.95063 | 0.44836 |
| H | -6.72432 | -2.35189 | -0.56213 |
| H | -6.21348 | -0.93032 | 0.34356 |
| C | -6.77186 | -2.27824 | 3.38996 |
| H | -7.14779 | -3.30407 | 3.49207 |
| H | -6.63690 | -1.89277 | 4.40503 |
| C | -5.57738 | -2.79996 | 1.21661 |
| H | -5.92038 | -3.83666 | 1.23188 |
| C | -7.95476 | -1.91309 | 1.17881 |
| H | -8.65043 | -1.26771 | 0.63404 |
| H | -8.39316 | -2.91903 | 1.17496 |
| C | -7.80299 | -1.43353 | 2.62898 |
| H | -7.48260 | -0.38389 | 2.63159 |
| H | -8.77000 | -1.46769 | 3.14040 |
| C | -5.41843 | -2.30716 | 2.66383 |
| H | -4.99385 | -1.29550 | 2.65493 |
| H | -4.70935 | -2.94944 | 3.19363 |
| O | 1.03405 | 0.31473 | -1.72767 |
| O | 1.44371 | 0.97051 | -2.94825 |
| H | 2.40789 | 0.93300 | -2.88630 |

**Appendix 13 Optimized Cartesian Coordinates of 3e.**

**ΔGsoln= -3986.0238 Hartree**

| | | | |
|---|---|---|---|
| Co | 1.42126 | 1.28885 | -0.10931 |
| F | -4.47001 | 6.67996 | 0.52496 |
| F | -2.14721 | 5.36318 | 0.87128 |
| F | 5.75459 | -1.00757 | -2.57254 |
| F | -6.37471 | 5.68771 | -1.15464 |
| F | 8.24399 | -2.03275 | -2.64174 |
| F | -3.59718 | 2.04203 | -2.19028 |
| F | 9.72113 | -2.20779 | -0.35735 |
| F | -5.92421 | 3.36005 | -2.50011 |
| N | 2.17316 | -0.34579 | 0.55916 |
| N | -0.38107 | 0.90049 | 0.41734 |
| F | 6.20607 | -0.27752 | 2.09230 |
| O | -0.26480 | -3.35202 | -0.72042 |
| N | 3.10281 | 1.84824 | -0.75070 |
| N | 0.88970 | 2.94005 | -0.85024 |
| N | 1.79401 | 2.23729 | 1.74317 |
| N | -2.50147 | -3.73972 | -0.70945 |
| F | 8.68375 | -1.32919 | 2.00582 |
| C | -2.61667 | 1.06353 | 0.83061 |
| H | -3.61875 | 1.46386 | 0.86799 |
| C | -1.48430 | 1.70031 | 0.20545 |
| C | -2.17634 | -0.10772 | 1.38559 |

| | | | |
|---|---|---|---|
| H | -2.74624 | -0.80726 | 1.97610 |
| C | -0.76598 | -0.21925 | 1.10277 |
| C | 0.07933 | -1.29271 | 1.44987 |
| C | -1.25003 | -3.48159 | 1.43417 |
| C | -0.31931 | 3.53613 | -1.00112 |
| C | -1.29649 | -3.49136 | -0.07491 |
| C | -1.48906 | 2.93731 | -0.47782 |
| C | -0.53837 | -2.47921 | 2.13159 |
| C | -0.41523 | -2.60119 | 3.52041 |
| H | 0.11452 | -1.82621 | 4.06259 |
| C | 1.46209 | -1.33557 | 1.18229 |
| C | 4.32284 | 1.25812 | -0.77387 |
| C | 1.86256 | 3.67532 | -1.45723 |
| C | 4.51726 | -0.01920 | -0.19672 |
| C | -3.05219 | 4.84806 | 0.02062 |
| C | -4.24552 | 5.53847 | -0.14148 |
| C | -3.78353 | 3.17816 | -1.49808 |
| C | 3.48230 | -0.75651 | 0.41741 |
| C | 3.14176 | 3.04970 | -1.39173 |
| C | 7.73397 | -1.59809 | -1.48064 |
| C | -4.98809 | 3.84607 | -1.67254 |
| C | -5.21902 | 5.03369 | -0.99235 |
| C | 6.45346 | -1.06384 | -1.42696 |
| C | 5.21710 | 2.15772 | -1.45478 |
| H | 6.26979 | 1.99131 | -1.63096 |

| | | | |
|---|---|---|---|
| C | -2.78227 | 3.65290 | -0.64857 |
| C | 0.90603 | 3.09460 | 2.27105 |
| H | 0.00141 | 3.26186 | 1.70467 |
| C | -2.46807 | -4.01445 | -2.17524 |
| H | -1.58689 | -3.47401 | -2.52404 |
| C | -1.77791 | -4.57411 | 2.13460 |
| H | -2.29827 | -5.35532 | 1.59431 |
| C | 3.59668 | -2.07249 | 1.00049 |
| H | 4.49934 | -2.66392 | 1.03706 |
| C | -1.62794 | -4.68210 | 3.51426 |
| H | -2.03549 | -5.54016 | 4.03598 |
| C | 8.48740 | -1.69182 | -0.31884 |
| C | -0.10410 | 4.75115 | -1.74320 |
| H | -0.86668 | 5.45875 | -2.03411 |
| C | 1.25126 | 4.83434 | -2.02309 |
| H | 1.75262 | 5.61965 | -2.56989 |
| C | 2.36324 | -2.42664 | 1.46791 |
| H | 2.08739 | -3.35341 | 1.94648 |
| C | -0.94923 | -3.68718 | 4.21135 |
| H | -0.83006 | -3.75523 | 5.28674 |
| C | 2.93331 | 2.01301 | 2.41688 |
| H | 3.63054 | 1.32069 | 1.96789 |
| C | 1.11858 | 3.74883 | 3.47889 |
| H | 0.36667 | 4.42998 | 3.85658 |
| C | 5.88508 | -0.60599 | -0.23731 |

| | | | |
|---|---|---|---|
| C | -3.69494 | -3.44259 | -2.89814 |
| H | -3.78292 | -2.37260 | -2.68747 |
| H | -4.60633 | -3.92550 | -2.52857 |
| C | 2.29944 | 3.51287 | 4.17515 |
| H | 2.49554 | 4.00793 | 5.11882 |
| C | 3.22325 | 2.62754 | 3.62948 |
| H | 4.15904 | 2.40936 | 4.12813 |
| C | 4.47930 | 3.26714 | -1.83955 |
| H | 4.84143 | 4.13103 | -2.37773 |
| C | 6.67492 | -0.71082 | 0.90862 |
| C | -2.26843 | -5.50703 | -2.48648 |
| H | -1.36385 | -5.86231 | -1.98393 |
| H | -3.11173 | -6.06991 | -2.07664 |
| C | 7.95506 | -1.24705 | 0.88338 |
| C | -3.58230 | -3.67881 | -4.41299 |
| H | -2.73571 | -3.10223 | -4.80609 |
| H | -4.47887 | -3.29622 | -4.91011 |
| C | -2.16493 | -5.73853 | -4.00173 |
| H | -1.25214 | -5.26151 | -4.38085 |
| H | -2.06490 | -6.80923 | -4.20545 |
| C | -3.38010 | -5.16422 | -4.74312 |
| H | -4.27839 | -5.72422 | -4.45313 |
| H | -3.26231 | -5.29683 | -5.82329 |
| O | -4.32536 | -5.03145 | -0.06533 |
| C | -3.76863 | -3.94301 | -0.04341 |

| | | | |
|---|---|---|---|
| N | -4.27474 | -2.83027 | 0.52185 |
| H | -3.77498 | -1.96199 | 0.38503 |
| C | -6.58966 | -1.95846 | 0.44076 |
| H | -6.70612 | -2.36997 | -0.56607 |
| H | -6.20041 | -0.93863 | 0.32709 |
| C | -6.76648 | -2.25693 | 3.38481 |
| H | -7.14125 | -3.28224 | 3.49594 |
| H | -6.63537 | -1.86114 | 4.39640 |
| C | -5.56414 | -2.79845 | 1.22070 |
| H | -5.90534 | -3.83556 | 1.24503 |
| C | -7.94288 | -1.91576 | 1.16639 |
| H | -8.63777 | -1.27696 | 0.61294 |
| H | -8.37972 | -2.92237 | 1.17121 |
| C | -7.79642 | -1.42143 | 2.61213 |
| H | -7.47760 | -0.37133 | 2.60518 |
| H | -8.76499 | -1.45190 | 3.12083 |
| C | -5.41064 | -2.29112 | 2.66341 |
| H | -4.98743 | -1.27900 | 2.64586 |
| H | -4.70244 | -2.92712 | 3.20191 |
| O | 1.02854 | 0.31826 | -1.74986 |
| O | 1.33114 | 0.84116 | -2.88846 |

**Appendix 14 Optimized Cartesian Coordinates of 3f.**

**ΔGsoln= -3985.8271 Hartree**

| | | | |
|---|---|---|---|
| Co | 1.50343 | 1.36655 | 0.04850 |
| F | -4.52538 | 6.60929 | 0.42808 |
| F | -2.19727 | 5.32633 | 0.89294 |
| F | 5.61401 | -0.89166 | -2.63533 |
| F | -6.25888 | 5.66145 | -1.44739 |
| F | 8.02539 | -2.07367 | -2.85666 |
| F | -3.31814 | 2.12565 | -2.41464 |
| F | 9.54861 | -2.50541 | -0.64072 |
| F | -5.64608 | 3.41604 | -2.86190 |
| N | 2.19498 | -0.29547 | 0.69794 |
| N | -0.32844 | 0.94046 | 0.43716 |
| F | 6.23037 | -0.54412 | 2.04889 |
| O | -0.38417 | -3.08402 | -0.72474 |
| N | 3.20013 | 1.95641 | -0.50680 |
| N | 1.00275 | 3.01054 | -0.72813 |
| N | 1.74549 | 2.24830 | 1.87673 |
| N | -2.59103 | -3.62101 | -0.72322 |
| F | 8.64163 | -1.73598 | 1.81055 |
| C | -2.58106 | 1.11670 | 0.74717 |
| H | -3.58229 | 1.51822 | 0.72717 |
| C | -1.42097 | 1.73869 | 0.17446 |
| C | -2.16764 | -0.04720 | 1.34154 |

| | | | |
|---|---|---|---|
| H | -2.76390 | -0.74294 | 1.90924 |
| C | -0.75401 | -0.16129 | 1.12089 |
| C | 0.07332 | -1.25190 | 1.49610 |
| C | -1.33475 | -3.38753 | 1.42311 |
| C | -0.20647 | 3.60337 | -0.93735 |
| C | -1.40056 | -3.32778 | -0.08434 |
| C | -1.38872 | 2.98427 | -0.50613 |
| C | -0.57844 | -2.43610 | 2.14436 |
| C | -0.42625 | -2.60195 | 3.52436 |
| H | 0.13953 | -1.86303 | 4.07969 |
| C | 1.44675 | -1.31373 | 1.25681 |
| C | 4.39762 | 1.30888 | -0.58484 |
| C | 1.98935 | 3.77837 | -1.24361 |
| C | 4.51734 | -0.01047 | -0.14466 |
| C | -3.02541 | 4.83906 | -0.04493 |
| C | -4.22027 | 5.50876 | -0.26926 |
| C | -3.59274 | 3.22595 | -1.69849 |
| C | 3.45048 | -0.75906 | 0.45348 |
| C | 3.27673 | 3.15236 | -1.12456 |
| C | 7.57782 | -1.69466 | -1.65379 |
| C | -4.79332 | 3.87898 | -1.94050 |
| C | -5.10670 | 5.02555 | -1.22246 |
| C | 6.33775 | -1.08283 | -1.52381 |
| C | 5.31815 | 2.20021 | -1.24829 |
| H | 6.36059 | 1.99936 | -1.44308 |

| | | | |
|---|---|---|---|
| C | -2.68035 | 3.68596 | -0.74905 |
| C | 0.84143 | 3.11009 | 2.37151 |
| H | -0.02575 | 3.31271 | 1.76196 |
| C | -2.55236 | -3.84440 | -2.19852 |
| H | -1.68612 | -3.26979 | -2.52898 |
| C | -1.87282 | -4.48912 | 2.09953 |
| H | -2.41697 | -5.24382 | 1.54638 |
| C | 3.52881 | -2.12655 | 0.91505 |
| H | 4.40040 | -2.75930 | 0.85211 |
| C | -1.69955 | -4.64474 | 3.47210 |
| H | -2.11849 | -5.50960 | 3.97264 |
| C | 8.35511 | -1.91798 | -0.52570 |
| C | 0.04039 | 4.84487 | -1.62595 |
| H | -0.71187 | 5.55329 | -1.93783 |
| C | 1.40484 | 4.95273 | -1.81133 |
| H | 1.93518 | 5.75702 | -2.29766 |
| C | 2.30610 | -2.45953 | 1.41784 |
| H | 2.00285 | -3.41057 | 1.82519 |
| C | -0.98211 | -3.69307 | 4.18917 |
| H | -0.84467 | -3.80015 | 5.25879 |
| C | 2.84091 | 1.98345 | 2.61007 |
| H | 3.55207 | 1.28980 | 2.18876 |
| C | 0.99434 | 3.72708 | 3.60583 |
| H | 0.23258 | 4.41194 | 3.95464 |
| C | 5.84167 | -0.67971 | -0.28441 |

| | | | |
|---|---|---|---|
| C | -3.79503 | -3.28074 | -2.90058 |
| H | -3.91034 | -2.22067 | -2.65542 |
| H | -4.69295 | -3.79895 | -2.54609 |
| C | 2.12661 | 3.45045 | 4.36409 |
| H | 2.27438 | 3.91698 | 5.33041 |
| C | 3.06596 | 2.56206 | 3.85154 |
| H | 3.96628 | 2.31283 | 4.39771 |
| C | 4.62053 | 3.34290 | -1.58230 |
| H | 4.99874 | 4.20874 | -2.10382 |
| C | 6.64864 | -0.91702 | 0.82847 |
| C | -2.31585 | -5.32070 | -2.55750 |
| H | -1.40209 | -5.66969 | -2.06707 |
| H | -3.14395 | -5.91834 | -2.16602 |
| C | 7.88992 | -1.52740 | 0.72322 |
| C | -3.67926 | -3.46448 | -4.42254 |
| H | -2.84878 | -2.85345 | -4.79760 |
| H | -4.58654 | -3.08933 | -4.90553 |
| C | -2.20858 | -5.49921 | -4.07970 |
| H | -1.30888 | -4.98656 | -4.44321 |
| H | -2.08128 | -6.55965 | -4.31829 |
| C | -3.43920 | -4.93252 | -4.80115 |
| H | -4.32231 | -5.52475 | -4.52950 |
| H | -3.31925 | -5.02637 | -5.88506 |
| O | -4.36830 | -4.98978 | -0.10175 |
| C | -3.85007 | -3.88402 | -0.06161 |

| | | | |
|---|---|---|---|
| N | -4.38885 | -2.80178 | 0.53261 |
| H | -3.94178 | -1.90899 | 0.37838 |
| C | -6.80043 | -2.22926 | 0.40546 |
| H | -6.88343 | -2.77841 | -0.53685 |
| H | -6.53921 | -1.19394 | 0.15242 |
| C | -6.88671 | -2.15616 | 3.36699 |
| H | -7.13384 | -3.19388 | 3.62363 |
| H | -6.78491 | -1.61624 | 4.31309 |
| C | -5.66731 | -2.83452 | 1.25193 |
| H | -5.88137 | -3.89284 | 1.41675 |
| C | -8.13573 | -2.25287 | 1.16427 |
| H | -8.91316 | -1.77983 | 0.55682 |
| H | -8.44653 | -3.29450 | 1.31403 |
| C | -8.02358 | -1.55701 | 2.52770 |
| H | -7.83397 | -0.48716 | 2.37332 |
| H | -8.97159 | -1.63282 | 3.06920 |
| C | -5.54930 | -2.12978 | 2.61149 |
| H | -5.24750 | -1.08676 | 2.45085 |
| H | -4.76311 | -2.60609 | 3.20412 |
| O | 1.30045 | 0.30252 | -1.56607 |
| O | 2.35801 | -0.05939 | -2.18370 |

**Appendix 15 Optimized Cartesian Coordinates of 4a.**

**ΔGsoln= -3144.6044 Hartree**

| | | | |
|---|---|---|---|
| Co | -0.00020 | -0.52853 | -0.24917 |
| F | -7.60463 | -1.69404 | -2.55942 |
| F | -4.92073 | -1.45264 | -2.61122 |
| F | 4.80044 | -1.88088 | 1.91078 |
| F | -8.97331 | -1.28841 | -0.23767 |
| F | 7.48230 | -2.14077 | 1.94911 |
| F | -4.94154 | -0.41934 | 2.01489 |
| F | 8.97026 | -1.31952 | -0.18177 |
| F | -7.62740 | -0.64660 | 2.04363 |
| N | 1.41682 | 0.76201 | -0.25786 |
| N | -1.41948 | 0.75712 | -0.23270 |
| F | 5.06320 | 0.00721 | -2.42998 |
| N | 1.23317 | -1.93961 | -0.43111 |
| N | 0.00124 | -0.66337 | 1.71707 |
| N | -1.22959 | -1.94205 | -0.44911 |
| F | 7.74518 | -0.24176 | -2.36481 |
| C | -3.48288 | 1.72460 | -0.22524 |
| H | -4.55682 | 1.83182 | -0.24549 |
| C | -2.77405 | 0.47224 | -0.23993 |
| C | -2.55784 | 2.73050 | -0.21691 |
| H | -2.75000 | 3.79142 | -0.22253 |

| | | | |
|---|---|---|---|
| C | -1.25135 | 2.12231 | -0.21072 |
| C | -0.00484 | 2.78687 | -0.18450 |
| C | -0.49381 | 4.97880 | 0.97218 |
| C | -2.58664 | -1.99148 | -0.44178 |
| C | -3.34992 | -0.81143 | -0.31735 |
| C | -0.00437 | 4.28248 | -0.14115 |
| C | 0.48448 | 5.03428 | -1.21753 |
| H | 0.85895 | 4.52257 | -2.09728 |
| C | 1.24324 | 2.12553 | -0.18660 |
| C | 2.58982 | -1.98216 | -0.42969 |
| C | -0.70715 | -3.19157 | -0.62901 |
| C | 3.35071 | -0.80170 | -0.28921 |
| C | -5.55893 | -1.25663 | -1.44582 |
| C | -6.94157 | -1.38228 | -1.43767 |
| C | -5.57052 | -0.72910 | 0.86925 |
| C | 2.77179 | 0.47946 | -0.20312 |
| C | 0.71620 | -3.18884 | -0.62772 |
| C | 6.87900 | -1.61314 | 0.87536 |
| C | 0.01978 | 0.41294 | 3.85505 |
| H | 0.03561 | 1.34235 | 4.40880 |
| C | -6.95358 | -0.84355 | 0.90238 |
| C | -7.64158 | -1.17302 | -0.25742 |
| C | 5.49827 | -1.47399 | 0.83771 |
| C | 2.96864 | -3.35455 | -0.63148 |
| H | 3.98081 | -3.72649 | -0.68889 |

| | | | |
|---|---|---|---|
| C | -4.83473 | -0.92878 | -0.29930 |
| C | 0.01945 | 0.45264 | 2.46808 |
| H | 0.03446 | 1.39495 | 1.94394 |
| C | -0.49266 | 6.37098 | 1.00606 |
| H | -0.87767 | 6.88122 | 1.88339 |
| C | 3.47280 | 1.73004 | -0.08038 |
| H | 4.54418 | 1.83877 | -0.00587 |
| C | -0.00168 | 7.12215 | -0.06777 |
| C | 7.63964 | -1.19319 | -0.20715 |
| C | -2.95889 | -3.36944 | -0.61631 |
| H | -3.96882 | -3.75016 | -0.65003 |
| C | -1.79321 | -4.10909 | -0.73475 |
| H | -1.71402 | -5.17550 | -0.88464 |
| C | 2.54474 | 2.73328 | -0.07294 |
| H | 2.73139 | 3.79219 | 0.00812 |
| C | 0.48405 | 6.42680 | -1.18031 |
| H | 0.86153 | 6.98115 | -2.03374 |
| C | 4.83459 | -0.92365 | -0.25952 |
| C | -0.01814 | -1.85601 | 2.33889 |
| H | -0.03277 | -2.73347 | 1.71233 |
| C | -0.00048 | -0.81865 | 4.49963 |
| H | -0.00112 | -0.87904 | 5.58104 |
| C | 1.80618 | -4.09895 | -0.75329 |
| H | 1.73160 | -5.16308 | -0.92072 |
| C | 5.63048 | -0.51776 | -1.33152 |

| | | | |
|---|---|---|---|
| C | 7.01312 | -0.64117 | -1.31606 |
| C | -0.01996 | -1.97068 | 3.72175 |
| H | -0.03632 | -2.95593 | 4.16861 |
| H | -0.87595 | 4.42366 | 1.82172 |
| C | 0.02611 | 8.63065 | -0.01574 |
| H | -0.81477 | 9.02495 | 0.55942 |
| H | 0.94421 | 8.98916 | 0.46261 |
| H | -0.01029 | 9.06401 | -1.01754 |
| O | 0.00520 | -0.38121 | -2.33079 |
| H | -0.79055 | 0.11485 | -2.56968 |
| H | 0.74532 | 0.20797 | -2.53635 |

**Appendix 16  Optimized Cartesian Coordinates of 4b.**

**ΔGsoln= -3143.98 Hartree**

| | | | |
|---|---|---|---|
| Co | 0.00506 | -0.51659 | -0.27473 |
| F | -7.56310 | -1.68611 | -2.62052 |
| F | -4.87448 | -1.47183 | -2.63929 |
| F | 4.83627 | -1.87409 | 1.88798 |
| F | -8.95495 | -1.25240 | -0.31955 |
| F | 7.52469 | -2.10280 | 1.87636 |
| F | -4.94282 | -0.39513 | 1.97296 |
| F | 8.95922 | -1.26912 | -0.28440 |
| F | -7.63407 | -0.60579 | 1.97348 |
| N | 1.42078 | 0.76730 | -0.16414 |
| N | -1.42158 | 0.76141 | -0.16066 |
| F | 4.99300 | 0.02279 | -2.44904 |
| N | 1.23476 | -1.94079 | -0.41573 |
| N | -0.00664 | -0.71309 | 1.81846 |
| N | -1.21909 | -1.94034 | -0.46088 |
| F | 7.68237 | -0.20583 | -2.44270 |
| C | -3.48324 | 1.72539 | -0.24998 |
| H | -4.55572 | 1.82642 | -0.31008 |
| C | -2.75954 | 0.48289 | -0.22475 |
| C | -2.56076 | 2.73521 | -0.22056 |
| H | -2.75456 | 3.79479 | -0.25029 |

| | | | |
|---|---|---|---|
| C | -1.25996 | 2.12130 | -0.15977 |
| C | -0.00444 | 2.78241 | -0.13032 |
| C | -0.58298 | 4.98581 | 0.94352 |
| C | -2.58258 | -1.99301 | -0.45803 |
| C | -3.33470 | -0.82049 | -0.32770 |
| C | -0.00512 | 4.27247 | -0.11604 |
| C | 0.56871 | 5.00006 | -1.16789 |
| H | 1.00695 | 4.47190 | -2.00673 |
| C | 1.25301 | 2.12446 | -0.11450 |
| C | 2.59628 | -1.97880 | -0.42939 |
| C | -0.70806 | -3.17351 | -0.65136 |
| C | 3.34099 | -0.80545 | -0.27869 |
| C | -5.52831 | -1.26078 | -1.48717 |
| C | -6.91219 | -1.37177 | -1.49444 |
| C | -5.56522 | -0.71050 | 0.82687 |
| C | 2.75959 | 0.49305 | -0.15318 |
| C | 0.73462 | -3.16926 | -0.64101 |
| C | 6.89560 | -1.58172 | 0.81629 |
| C | -0.01865 | 0.31313 | 3.98786 |
| H | -0.01859 | 1.22926 | 4.56403 |
| C | -6.94873 | -0.81594 | 0.84353 |
| C | -7.62394 | -1.14794 | -0.32368 |
| C | 5.51286 | -1.45912 | 0.80616 |
| C | 2.98262 | -3.35081 | -0.66562 |
| H | 3.99506 | -3.71841 | -0.73592 |

| | | | |
|---|---|---|---|
| C | -4.82067 | -0.92919 | -0.33219 |
| C | -0.00784 | 0.37904 | 2.60082 |
| H | -0.00011 | 1.33497 | 2.10026 |
| C | -0.57766 | 6.37722 | 0.95134 |
| H | -1.02438 | 6.90418 | 1.78811 |
| C | 3.47801 | 1.73490 | -0.04539 |
| H | 4.55047 | 1.83809 | 0.01069 |
| C | -0.00873 | 7.10690 | -0.09884 |
| C | 7.62885 | -1.15550 | -0.28279 |
| C | -2.95518 | -3.37453 | -0.64562 |
| H | -3.96365 | -3.75749 | -0.68176 |
| C | -1.79037 | -4.10427 | -0.77057 |
| H | -1.70143 | -5.16861 | -0.92529 |
| C | 2.55139 | 2.74084 | -0.02027 |
| H | 2.74024 | 3.79864 | 0.06137 |
| C | 0.55825 | 6.39155 | -1.15964 |
| H | 0.99464 | 6.92959 | -1.99475 |
| C | 4.82758 | -0.91503 | -0.27954 |
| C | -0.01561 | -1.91433 | 2.41884 |
| H | -0.01341 | -2.78048 | 1.77457 |
| C | -0.02962 | -0.93337 | 4.60432 |
| H | -0.03886 | -1.01883 | 5.68416 |
| C | 1.82467 | -4.08818 | -0.79780 |
| H | 1.74354 | -5.14772 | -0.98627 |
| C | 5.59278 | -0.49833 | -1.36840 |

| | | | |
|---|---|---|---|
| C | 6.97603 | -0.61082 | -1.38074 |
| C | -0.02839 | -2.06710 | 3.79930 |
| H | -0.03634 | -3.06247 | 4.22407 |
| H | -1.02798 | 4.44730 | 1.77223 |
| C | 0.01445 | 8.61528 | -0.07551 |
| H | -0.85001 | 9.01838 | 0.45646 |
| H | 0.91194 | 8.98266 | 0.43403 |
| H | 0.02082 | 9.02865 | -1.08620 |
| O | 0.06050 | -0.28623 | -2.15198 |
| H | -0.79468 | -0.60488 | -2.46081 |

**Appendix 17 Optimized Cartesian Coordinates of 4c.**

**ΔGsoln= -3143.3161 Hartree**

| | | | |
|---|---|---|---|
| Co | -0.00196 | -0.52320 | -0.25927 |
| F | -7.53196 | -1.66975 | -2.67187 |
| F | -4.84260 | -1.46022 | -2.65133 |
| F | 4.84156 | -1.85453 | 1.86698 |
| F | -8.95485 | -1.25156 | -0.38721 |
| F | 7.53040 | -2.08006 | 1.84340 |
| F | -4.97309 | -0.41800 | 1.96685 |
| F | 8.95245 | -1.26273 | -0.33169 |
| F | -7.66499 | -0.62492 | 1.92900 |
| N | 1.41293 | 0.76799 | -0.17664 |
| N | -1.42329 | 0.75806 | -0.14454 |
| F | 4.97326 | 0.00725 | -2.48571 |
| N | 1.22825 | -1.94660 | -0.41759 |
| N | 0.01825 | -0.70262 | 1.84399 |
| N | -1.22254 | -1.95759 | -0.40575 |
| F | 7.66324 | -0.21864 | -2.49190 |
| C | -3.48609 | 1.71499 | -0.23873 |
| H | -4.55878 | 1.81282 | -0.29850 |
| C | -2.75981 | 0.47495 | -0.21154 |
| C | -2.56713 | 2.72836 | -0.20981 |
| H | -2.76442 | 3.78720 | -0.24122 |
| C | -1.26499 | 2.11952 | -0.14841 |

| | | | |
|---|---|---|---|
| C | -0.01097 | 2.78356 | -0.13026 |
| C | -0.59260 | 4.98748 | 0.93895 |
| C | -2.58456 | -2.00262 | -0.42602 |
| C | -3.33434 | -0.82866 | -0.31462 |
| C | -0.01314 | 4.27308 | -0.11915 |
| C | 0.56100 | 4.99938 | -1.17189 |
| H | 0.99985 | 4.47042 | -2.00989 |
| C | 1.24707 | 2.12593 | -0.12396 |
| C | 2.59053 | -1.98134 | -0.43887 |
| C | -0.71426 | -3.18806 | -0.60732 |
| C | 3.33424 | -0.80554 | -0.30058 |
| C | -5.51239 | -1.25604 | -1.50744 |
| C | -6.89626 | -1.36480 | -1.53463 |
| C | -5.58060 | -0.72445 | 0.81037 |
| C | 2.75287 | 0.49211 | -0.17625 |
| C | 0.72859 | -3.18182 | -0.61674 |
| C | 6.89520 | -1.56859 | 0.78245 |
| C | 0.04618 | 0.33865 | 4.00660 |
| H | 0.06176 | 1.25876 | 4.57620 |
| C | -6.96438 | -0.82764 | 0.80711 |
| C | -7.62376 | -1.14918 | -0.37205 |
| C | 5.51229 | -1.44773 | 0.77856 |
| C | 2.97694 | -3.35508 | -0.65561 |
| H | 3.98964 | -3.72168 | -0.72652 |
| C | -4.82080 | -0.93488 | -0.34003 |

| | | | |
|---|---|---|---|
| C | 0.03715 | 0.39447 | 2.61906 |
| H | 0.04589 | 1.34733 | 2.11222 |
| C | -0.58818 | 6.37881 | 0.94458 |
| H | -1.03569 | 6.90673 | 1.78028 |
| C | 3.47166 | 1.73333 | -0.07391 |
| H | 4.54449 | 1.83559 | -0.02500 |
| C | -0.01905 | 7.10723 | -0.10639 |
| C | 7.62210 | -1.15067 | -0.32406 |
| C | -2.96112 | -3.38269 | -0.62463 |
| H | -3.97079 | -3.76103 | -0.67300 |
| C | -1.79875 | -4.11577 | -0.74217 |
| H | -1.71142 | -5.17912 | -0.90424 |
| C | 2.54618 | 2.74029 | -0.04029 |
| H | 2.73657 | 3.79780 | 0.04097 |
| C | 0.54920 | 6.39072 | -1.16582 |
| H | 0.98544 | 6.92781 | -2.00158 |
| C | 4.82095 | -0.91361 | -0.30823 |
| C | 0.00741 | -1.89922 | 2.45344 |
| H | -0.00851 | -2.77022 | 1.81568 |
| C | 0.03499 | -0.90344 | 4.63190 |
| H | 0.04148 | -0.98134 | 5.71234 |
| C | 1.81957 | -4.09828 | -0.76644 |
| H | 1.74042 | -5.16112 | -0.93582 |
| C | 5.57969 | -0.50484 | -1.40453 |
| C | 6.96299 | -0.61582 | -1.42313 |

| | | | |
|---|---|---|---|
| C | 0.01523 | -2.04273 | 3.83496 |
| H | 0.00570 | -3.03504 | 4.26682 |
| H | -1.03727 | 4.44994 | 1.76839 |
| C | 0.00311 | 8.61556 | -0.08541 |
| H | -0.86032 | 9.01886 | 0.44802 |
| H | 0.90163 | 8.98410 | 0.42145 |
| H | 0.00694 | 9.02740 | -1.09674 |
| O | -0.02846 | -0.34431 | -2.07485 |

**Appendix 18 Optimized Cartesian Coordinates of 4d.**

**ΔGsoln= -3219.2925 Hartree**

| | | | |
|---|---|---|---|
| Co | -0.02154 | -0.50607 | -0.26524 |
| F | -7.58441 | -1.76431 | -2.55046 |
| F | -4.90378 | -1.51910 | -2.56660 |
| F | 4.77361 | -1.82898 | 1.96985 |
| F | -8.99446 | -1.28624 | -0.26666 |
| F | 7.45352 | -2.08686 | 2.01501 |
| F | -5.00346 | -0.34619 | 2.02864 |
| F | 8.94605 | -1.28923 | -0.12284 |
| F | -7.68635 | -0.57145 | 2.01631 |
| N | 1.39017 | 0.78229 | -0.17135 |
| N | -1.44648 | 0.76992 | -0.13645 |
| F | 5.04269 | 0.00424 | -2.39619 |
| N | 1.21345 | -1.92921 | -0.41563 |
| N | 0.00710 | -0.67772 | 1.84979 |
| N | -1.24907 | -1.94206 | -0.37744 |
| F | 7.72281 | -0.23888 | -2.32112 |
| C | -3.51296 | 1.73171 | -0.18537 |
| H | -4.58709 | 1.83529 | -0.22425 |
| C | -2.79358 | 0.48048 | -0.17727 |
| C | -2.59146 | 2.73971 | -0.16940 |
| H | -2.78647 | 3.80032 | -0.18731 |

| | | | |
|---|---|---|---|
| C | -1.28171 | 2.12883 | -0.13777 |
| C | -0.03534 | 2.79745 | -0.12345 |
| C | -0.55022 | 5.00839 | 0.98881 |
| C | -2.60260 | -1.99073 | -0.38230 |
| C | -3.36619 | -0.80585 | -0.26524 |
| C | -0.03823 | 4.29301 | -0.10292 |
| C | 0.46910 | 5.03260 | -1.18043 |
| H | 0.85896 | 4.50819 | -2.04581 |
| C | 1.21582 | 2.13738 | -0.12465 |
| C | 2.56933 | -1.97130 | -0.38640 |
| C | -0.72808 | -3.18824 | -0.56087 |
| C | 3.32150 | -0.78080 | -0.24614 |
| C | -5.55774 | -1.28950 | -1.41572 |
| C | -6.94029 | -1.41692 | -1.42697 |
| C | -5.60935 | -0.68904 | 0.87895 |
| C | 2.73854 | 0.50022 | -0.15881 |
| C | 0.69952 | -3.18420 | -0.57648 |
| C | 6.85138 | -1.57254 | 0.93288 |
| C | 0.02845 | 0.38273 | 4.00265 |
| H | 0.03541 | 1.30720 | 4.56596 |
| C | -6.99283 | -0.80304 | 0.89223 |
| C | -7.66151 | -1.17030 | -0.26716 |
| C | 5.47061 | -1.43448 | 0.89061 |
| C | 2.95410 | -3.34761 | -0.54509 |
| H | 3.96683 | -3.72235 | -0.57644 |

| | | | |
|---|---|---|---|
| C | -4.85054 | -0.92417 | -0.26892 |
| C | 0.01587 | 0.42660 | 2.61351 |
| H | 0.01409 | 1.37190 | 2.09110 |
| C | -0.55299 | 6.40130 | 1.00164 |
| H | -0.95637 | 6.92397 | 1.86351 |
| C | 3.44882 | 1.75374 | -0.05996 |
| H | 4.52174 | 1.86283 | -0.00571 |
| C | -0.04188 | 7.13763 | -0.07288 |
| C | 7.61425 | -1.16469 | -0.15240 |
| C | -2.97982 | -3.36967 | -0.55189 |
| H | -3.98984 | -3.75124 | -0.58523 |
| C | -1.81245 | -4.10990 | -0.66672 |
| H | -1.73332 | -5.17792 | -0.80857 |
| C | 2.52116 | 2.75542 | -0.04156 |
| H | 2.70801 | 3.81540 | 0.02783 |
| C | 0.46667 | 6.42554 | -1.16457 |
| H | 0.85937 | 6.96732 | -2.01948 |
| C | 4.80598 | -0.89831 | -0.21347 |
| C | 0.00933 | -1.86916 | 2.46802 |
| H | 0.00038 | -2.74124 | 1.83048 |
| C | 0.03144 | -0.85427 | 4.63872 |
| H | 0.04097 | -0.92270 | 5.72005 |
| C | 1.79097 | -4.09733 | -0.66321 |
| H | 1.72122 | -5.16719 | -0.79558 |
| C | 5.60548 | -0.50590 | -1.28811 |

| | | | |
|---|---|---|---|
| C | 6.98857 | -0.62664 | -1.26829 |
| C | 0.02182 | -2.00101 | 3.85177 |
| H | 0.02344 | -2.98922 | 4.29378 |
| H | -0.94749 | 4.46585 | 1.83962 |
| C | -0.01681 | 8.64708 | -0.04390 |
| H | -0.85603 | 9.04876 | 0.52866 |
| H | 0.90273 | 9.01595 | 0.42397 |
| H | -0.05896 | 9.06513 | -1.05217 |
| O | -0.17008 | -0.33836 | -2.16018 |
| O | 1.09549 | -0.35666 | -2.85917 |
| H | 1.40582 | -1.25741 | -2.69294 |

**Appendix 19 Optimized Cartesian Coordinates of 4e.**

**ΔGsoln= -3218.6867 Hartree**

| | | | |
|---|---|---|---|
| Co | -0.00988 | -0.51619 | -0.23117 |
| F | -7.56562 | -1.77794 | -2.57835 |
| F | -4.88469 | -1.53396 | -2.58459 |
| F | 4.80747 | -1.86179 | 1.94761 |
| F | -8.98499 | -1.28630 | -0.30327 |
| F | 7.48650 | -2.12371 | 1.95801 |
| F | -5.00331 | -0.33466 | 2.00327 |
| F | 8.95575 | -1.30085 | -0.18665 |
| F | -7.68617 | -0.55908 | 1.98107 |
| N | 1.39977 | 0.77757 | -0.17508 |
| N | -1.43552 | 0.76690 | -0.14839 |
| F | 5.02910 | 0.02572 | -2.39885 |
| N | 1.22141 | -1.93982 | -0.35261 |
| N | -0.00774 | -0.65847 | 1.88146 |
| N | -1.23889 | -1.94716 | -0.37028 |
| F | 7.70832 | -0.22079 | -2.35688 |
| C | -3.50157 | 1.72918 | -0.21202 |
| H | -4.57536 | 1.83294 | -0.25867 |
| C | -2.78333 | 0.47791 | -0.19635 |
| C | -2.58011 | 2.73732 | -0.19190 |
| H | -2.77476 | 3.79790 | -0.21463 |

| | | | |
|---|---|---|---|
| C | -1.27096 | 2.12704 | -0.15086 |
| C | -0.02506 | 2.79554 | -0.13441 |
| C | -0.53928 | 5.00378 | 0.98111 |
| C | -2.59327 | -1.99471 | -0.38435 |
| C | -3.35663 | -0.80836 | -0.28034 |
| C | -0.02803 | 4.29110 | -0.11261 |
| C | 0.47814 | 5.03294 | -1.18909 |
| H | 0.86757 | 4.51060 | -2.05594 |
| C | 1.22605 | 2.13499 | -0.13343 |
| C | 2.57524 | -1.97580 | -0.35826 |
| C | -0.71672 | -3.19708 | -0.53443 |
| C | 3.33016 | -0.78657 | -0.23925 |
| C | -5.54352 | -1.29733 | -1.43791 |
| C | -6.92607 | -1.42435 | -1.45421 |
| C | -5.60454 | -0.68384 | 0.85310 |
| C | 2.74947 | 0.49614 | -0.16093 |
| C | 0.71046 | -3.19137 | -0.52985 |
| C | 6.87275 | -1.59533 | 0.88922 |
| C | 0.03796 | 0.42241 | 4.02283 |
| H | 0.08038 | 1.35127 | 4.57724 |
| C | -6.98808 | -0.79725 | 0.86124 |
| C | -7.65204 | -1.17086 | -0.29885 |
| C | 5.49179 | -1.45528 | 0.86465 |
| C | 2.96375 | -3.34970 | -0.53857 |
| H | 3.97747 | -3.71926 | -0.59193 |

| | | | |
|---|---|---|---|
| C | -4.84100 | -0.92570 | -0.29029 |
| C | 0.04201 | 0.45268 | 2.63326 |
| H | 0.08781 | 1.39152 | 2.10083 |
| C | -0.54215 | 6.39669 | 0.99712 |
| H | -0.94483 | 6.91737 | 1.86050 |
| C | 3.45855 | 1.74926 | -0.06960 |
| H | 4.53148 | 1.85903 | -0.01783 |
| C | -0.03200 | 7.13538 | -0.07612 |
| C | 7.62383 | -1.17440 | -0.19922 |
| C | -2.96902 | -3.37431 | -0.54411 |
| H | -3.97895 | -3.75560 | -0.58285 |
| C | -1.80109 | -4.11728 | -0.64123 |
| H | -1.72245 | -5.18658 | -0.77309 |
| C | 2.53066 | 2.75173 | -0.05459 |
| H | 2.71808 | 3.81199 | 0.00833 |
| C | 0.47550 | 6.42580 | -1.17004 |
| H | 0.86735 | 6.96961 | -2.02406 |
| C | 4.81469 | -0.90449 | -0.22486 |
| C | -0.06579 | -1.84291 | 2.51019 |
| H | -0.10673 | -2.71975 | 1.88025 |
| C | -0.02109 | -0.80711 | 4.67061 |
| H | -0.02587 | -0.86482 | 5.75258 |
| C | 1.80298 | -4.10166 | -0.64646 |
| H | 1.73331 | -5.16955 | -0.79379 |
| C | 5.60287 | -0.49848 | -1.30318 |

| | | | |
|---|---|---|---|
| C | 6.98589 | -0.62106 | -1.30054 |
| C | -0.07402 | -1.96062 | 3.89520 |
| H | -0.12148 | -2.94318 | 4.34715 |
| H | -0.93584 | 4.45911 | 1.83090 |
| C | -0.00645 | 8.64474 | -0.04373 |
| H | -0.83647 | 9.04489 | 0.54305 |
| H | 0.92051 | 9.01231 | 0.41030 |
| H | -0.06443 | 9.06543 | -1.05015 |
| O | -0.13671 | -0.34230 | -2.16676 |
| O | 0.93300 | -0.46443 | -2.87651 |

**Appendix 20 Optimized Cartesian Coordinates of 4f.**

**ΔGsoln= -3218.4941 Hartree**

| | | | |
|---|---|---|---|
| Co | -0.00486 | -0.47617 | -0.26738 |
| F | -7.66836 | -1.45829 | -2.48299 |
| F | -4.98633 | -1.20446 | -2.58381 |
| F | 4.70523 | -1.97865 | 1.89963 |
| F | -8.97163 | -1.28781 | -0.09696 |
| F | 7.38912 | -2.26007 | 2.00949 |
| F | -4.88077 | -0.62116 | 2.11726 |
| F | 8.94182 | -1.36425 | -0.04199 |
| F | -7.56632 | -0.86418 | 2.19819 |
| N | 1.42234 | 0.76194 | -0.03612 |
| N | -1.44006 | 0.76737 | -0.00229 |
| F | 5.10843 | 0.09933 | -2.33514 |
| N | 1.22135 | -1.83213 | -0.71198 |
| N | 0.03618 | -0.96476 | 1.70966 |
| N | -1.23963 | -1.84884 | -0.66406 |
| F | 7.79172 | -0.18463 | -2.21095 |
| C | -3.50185 | 1.73865 | -0.00794 |
| H | -4.57492 | 1.84832 | -0.03730 |
| C | -2.79267 | 0.48792 | -0.05668 |
| C | -2.57441 | 2.74151 | 0.01891 |
| H | -2.76361 | 3.80230 | 0.02179 |
| C | -1.27261 | 2.12129 | 0.02757 |

| | | | |
|---|---|---|---|
| C | -0.00761 | 2.76840 | 0.02234 |
| C | -0.54677 | 4.98051 | 1.10275 |
| C | -2.59723 | -1.92264 | -0.60167 |
| C | -3.35882 | -0.78493 | -0.27495 |
| C | 0.00745 | 4.25895 | 0.03606 |
| C | 0.58585 | 4.97710 | -1.01892 |
| H | 1.00742 | 4.44016 | -1.86051 |
| C | 1.25192 | 2.10915 | 0.07655 |
| C | 2.58227 | -1.90518 | -0.64333 |
| C | -0.72774 | -3.03720 | -1.06313 |
| C | 3.33962 | -0.78652 | -0.28345 |
| C | -5.59387 | -1.12798 | -1.39019 |
| C | -6.97570 | -1.25625 | -1.35574 |
| C | -5.54339 | -0.82293 | 0.96793 |
| C | 2.76696 | 0.48490 | -0.02151 |
| C | 0.71088 | -3.02738 | -1.08062 |
| C | 6.82177 | -1.68098 | 0.94448 |
| C | 0.02791 | -0.23788 | 3.99279 |
| H | 0.00850 | 0.59144 | 4.68758 |
| C | -6.92444 | -0.94704 | 1.02658 |
| C | -7.64257 | -1.16570 | -0.14147 |
| C | 5.44363 | -1.52970 | 0.87309 |
| C | 2.95521 | -3.24765 | -1.01561 |
| H | 3.96397 | -3.62816 | -1.06973 |
| C | -4.84252 | -0.91090 | -0.23510 |

| | | | |
|---|---|---|---|
| C | 0.01274 | 0.01657 | 2.62867 |
| H | -0.01845 | 1.02926 | 2.25706 |
| C | -0.51426 | 6.37147 | 1.11339 |
| H | -0.94234 | 6.90506 | 1.95573 |
| C | 3.47541 | 1.71829 | 0.17857 |
| H | 4.54657 | 1.82048 | 0.25849 |
| C | 0.05890 | 7.09237 | 0.05923 |
| C | 7.61501 | -1.22425 | -0.09946 |
| C | -2.96871 | -3.26152 | -0.97249 |
| H | -3.97529 | -3.64827 | -1.01959 |
| C | -1.80509 | -3.95084 | -1.26572 |
| H | -1.71967 | -4.97819 | -1.58526 |
| C | 2.54393 | 2.72180 | 0.22871 |
| H | 2.72709 | 3.77520 | 0.36410 |
| C | 0.60267 | 6.36864 | -1.00781 |
| H | 1.04266 | 6.90002 | -1.84527 |
| C | 4.82308 | -0.92781 | -0.22004 |
| C | 0.07400 | -2.23709 | 2.13764 |
| H | 0.09044 | -3.00678 | 1.38085 |
| C | 0.06762 | -1.55628 | 4.43423 |
| H | 0.07982 | -1.78623 | 5.49262 |
| C | 1.79392 | -3.94156 | -1.28751 |
| H | 1.70657 | -4.97148 | -1.59785 |
| C | 5.64643 | -0.48220 | -1.25264 |
| C | 7.02670 | -0.62148 | -1.20344 |

| | | | |
|---|---|---|---|
| C | 0.09075 | -2.57190 | 3.48501 |
| H | 0.12111 | -3.61479 | 3.77200 |
| H | -0.99265 | 4.44920 | 1.93566 |
| C | 0.11041 | 8.60001 | 0.08627 |
| H | -0.76239 | 9.01895 | 0.59202 |
| H | 0.99799 | 8.94927 | 0.62506 |
| H | 0.15605 | 9.01470 | -0.92274 |
| O | -0.31811 | 0.16678 | -2.08748 |
| O | 0.21764 | 1.27421 | -2.43187 |

**Appendix 21 Optimized Cartesian Coordinates of 3c-T.**

**ΔGsoln= -3836.5855 Hartree**

| | | | |
|---|---|---|---|
| Co | 0.71081 | 1.74117 | -0.05650 |
| F | -6.42886 | 5.41162 | 0.46232 |
| F | -3.80084 | 4.88593 | 0.76495 |
| F | 5.11619 | 0.16755 | -2.86789 |
| F | -8.02237 | 3.68153 | -0.91106 |
| F | 7.74974 | -0.32524 | -3.19264 |
| F | -4.33158 | 0.89160 | -1.72846 |
| F | 9.48609 | 0.11049 | -1.14033 |
| F | -6.96223 | 1.41602 | -1.99413 |
| N | 1.89506 | 0.39807 | 0.63859 |
| N | -0.88644 | 1.00087 | 0.69138 |
| F | 5.93874 | 1.55399 | 1.58504 |
| N | 2.13499 | 2.59978 | -0.94441 |
| N | -0.26253 | 3.15390 | -0.85329 |
| N | 1.01589 | 2.91622 | 1.65334 |
| N | -0.76060 | -4.77953 | -0.00009 |
| F | 8.57083 | 1.05055 | 1.24690 |
| C | -3.05622 | 0.63737 | 1.26932 |
| H | -4.12362 | 0.77284 | 1.34523 |
| C | -2.16029 | 1.46109 | 0.50508 |
| C | -2.30135 | -0.32458 | 1.88685 |
| H | -2.64912 | -1.10390 | 2.54596 |

| | | | |
|---|---|---|---|
| C | -0.93388 | -0.09969 | 1.50542 |
| C | 0.18306 | -0.89996 | 1.84010 |
| C | -0.33957 | -3.36998 | 2.06773 |
| C | -1.60224 | 3.38747 | -0.97429 |
| C | -0.26883 | -3.51640 | 0.54784 |
| C | -2.52788 | 2.57652 | -0.31326 |
| C | -0.08210 | -2.12130 | 2.66985 |
| C | -0.10050 | -2.00587 | 4.06313 |
| H | 0.09767 | -1.04062 | 4.51532 |
| C | 1.50895 | -0.66053 | 1.41456 |
| C | 3.45671 | 2.28900 | -1.08143 |
| C | 0.42486 | 3.99187 | -1.64912 |
| C | 3.99330 | 1.16345 | -0.44773 |
| C | -4.55396 | 4.02147 | 0.06757 |
| C | -5.90578 | 4.30365 | -0.07469 |
| C | -4.82370 | 2.00975 | -1.17282 |
| C | 3.22754 | 0.26944 | 0.36251 |
| C | 1.83230 | 3.68226 | -1.68621 |
| C | 7.29781 | 0.13784 | -2.02172 |
| C | -6.17810 | 2.26834 | -1.32325 |
| C | -6.72082 | 3.42173 | -0.77181 |
| C | 5.94615 | 0.39793 | -1.84020 |
| C | 4.03624 | 3.28227 | -1.95446 |
| H | 5.07279 | 3.33346 | -2.25079 |
| C | -3.97813 | 2.87178 | -0.47307 |

| | | | |
|---|---|---|---|
| C | 0.00074 | 3.59921 | 2.20854 |
| H | -0.96643 | 3.50010 | 1.73910 |
| C | 0.26929 | -5.78048 | -0.37699 |
| H | 1.14137 | -5.50620 | 0.22632 |
| C | -0.62430 | -4.46294 | 2.88798 |
| H | -0.84353 | -5.41734 | 2.42632 |
| C | 3.71607 | -0.91874 | 1.01265 |
| H | 4.73083 | -1.28297 | 0.97456 |
| C | -0.64318 | -4.33994 | 4.27606 |
| H | -0.86721 | -5.20470 | 4.89023 |
| C | 8.18452 | 0.35712 | -0.97613 |
| C | -1.76025 | 4.49679 | -1.88782 |
| H | -2.69839 | 4.93591 | -2.19099 |
| C | -0.50166 | 4.87069 | -2.30431 |
| H | -0.25058 | 5.65943 | -2.99671 |
| C | 2.65803 | -1.48975 | 1.66571 |
| H | 2.66163 | -2.39245 | 2.25607 |
| C | -0.37542 | -3.10942 | 4.86808 |
| H | -0.38596 | -3.00315 | 5.94660 |
| C | 2.23142 | 3.03082 | 2.21384 |
| H | 3.03445 | 2.47611 | 1.75250 |
| C | 0.15818 | 4.40392 | 3.32923 |
| H | -0.69819 | 4.92887 | 3.73214 |
| C | 5.44419 | 0.87647 | -0.63069 |
| C | 0.68324 | -5.68462 | -1.85759 |

| | | | |
|---|---|---|---|
| H | 0.97750 | -4.65508 | -2.08793 |
| H | -0.18688 | -5.92028 | -2.47564 |
| C | 1.41831 | 4.51735 | 3.90675 |
| H | 1.57423 | 5.13781 | 4.78092 |
| C | 2.47355 | 3.81622 | 3.33332 |
| H | 3.47533 | 3.86918 | 3.73943 |
| C | 3.02620 | 4.14245 | -2.33164 |
| H | 3.10760 | 4.99682 | -2.98583 |
| C | 6.36057 | 1.08705 | 0.39887 |
| C | -0.08681 | -7.22463 | 0.01443 |
| H | -0.32635 | -7.26601 | 1.08182 |
| H | -0.97848 | -7.53567 | -0.53176 |
| C | 7.71568 | 0.83414 | 0.24108 |
| C | 1.83553 | -6.64926 | -2.17719 |
| H | 2.73669 | -6.33143 | -1.63651 |
| H | 2.07809 | -6.59599 | -3.24332 |
| C | 1.07916 | -8.17496 | -0.30225 |
| H | 1.94158 | -7.92145 | 0.32825 |
| H | 0.79643 | -9.20055 | -0.04450 |
| C | 1.49274 | -8.09117 | -1.77819 |
| H | 0.66799 | -8.45352 | -2.40502 |
| H | 2.34611 | -8.74975 | -1.96999 |
| O | -2.39722 | -5.79466 | -1.25534 |
| C | -2.05624 | -4.89682 | -0.47942 |
| N | -2.95524 | -3.96682 | -0.02185 |

| | | | |
|---|---|---|---|
| H | -2.67096 | -3.28139 | 0.65884 |
| C | -4.39891 | -2.79646 | -1.67287 |
| H | -3.72159 | -3.07979 | -2.48443 |
| H | -4.04318 | -1.83650 | -1.27973 |
| C | -6.73461 | -3.37572 | 0.05079 |
| H | -7.08333 | -4.34006 | -0.34003 |
| H | -7.40500 | -3.11273 | 0.87496 |
| C | -4.31299 | -3.85057 | -0.55321 |
| H | -4.55700 | -4.82829 | -0.97525 |
| C | -5.83282 | -2.63511 | -2.19845 |
| H | -5.86315 | -1.84451 | -2.95460 |
| H | -6.14259 | -3.56101 | -2.69995 |
| C | -6.81588 | -2.32175 | -1.06231 |
| H | -6.58112 | -1.33374 | -0.64682 |
| H | -7.83751 | -2.26246 | -1.45118 |
| C | -5.30135 | -3.53630 | 0.57945 |
| H | -4.99291 | -2.60572 | 1.07462 |
| H | -5.25515 | -4.32850 | 1.33306 |
| O | 0.43955 | 0.71947 | -1.54550 |
| H | 0.77493 | -3.40923 | 0.24017 |
| H | -0.78653 | -2.68032 | 0.07062 |

**Appendix 22 Optimized Cartesian Coordinates of 3d-T.**

**ΔGsoln= -3912.5619 Hartree**

| | | | |
|---|---|---|---|
| Co | -0.73137 | 1.70667 | 0.06962 |
| F | 6.20569 | 5.73246 | -0.58431 |
| F | 3.62006 | 5.04096 | -0.87439 |
| F | -5.22307 | 0.12794 | 2.79067 |
| F | 7.90480 | 4.13896 | 0.83207 |
| F | -7.85447 | -0.38561 | 3.04267 |
| F | 4.39008 | 1.15717 | 1.74079 |
| F | -9.52408 | -0.07982 | 0.90965 |
| F | 6.97972 | 1.84297 | 1.97841 |
| N | -1.86187 | 0.32967 | -0.64618 |
| N | 0.89316 | 1.03791 | -0.69183 |
| F | -5.90879 | 1.30827 | -1.75224 |
| N | -2.20364 | 2.52971 | 0.91873 |
| N | 0.18159 | 3.16566 | 0.84404 |
| N | -1.06800 | 2.87757 | -1.65966 |
| N | 0.82894 | -4.77145 | 0.08599 |
| F | -8.53271 | 0.76409 | -1.48569 |
| C | 3.08634 | 0.73450 | -1.23385 |
| H | 4.15128 | 0.89958 | -1.29854 |
| C | 2.15538 | 1.55334 | -0.49537 |
| C | 2.37221 | -0.26356 | -1.83574 |
| H | 2.75474 | -1.04875 | -2.46943 |

| | | | |
|---|---|---|---|
| C | 0.98470 | -0.07665 | -1.47798 |
| C | -0.09346 | -0.91993 | -1.82347 |
| C | 0.47586 | -3.38756 | -2.01288 |
| C | 1.49731 | 3.47834 | 0.93511 |
| C | 0.34708 | -3.51674 | -0.49592 |
| C | 2.47279 | 2.69088 | 0.27942 |
| C | 0.22588 | -2.14430 | -2.63306 |
| C | 0.31083 | -2.05251 | -4.02653 |
| H | 0.11885 | -1.09493 | -4.49832 |
| C | -1.42520 | -0.71802 | -1.40936 |
| C | -3.51658 | 2.20129 | 1.01030 |
| C | -0.56477 | 4.02820 | 1.58703 |
| C | -4.01494 | 1.04032 | 0.37059 |
| C | 4.41773 | 4.23802 | -0.14864 |
| C | 5.74933 | 4.60772 | -0.01493 |
| C | 4.80595 | 2.28721 | 1.14399 |
| C | -3.20796 | 0.17151 | -0.39544 |
| C | -1.94664 | 3.67463 | 1.61577 |
| C | -7.36647 | 0.03176 | 1.86583 |
| C | 6.14411 | 2.62836 | 1.28230 |
| C | 6.61818 | 3.79680 | 0.70215 |
| C | -6.01304 | 0.30426 | 1.71845 |
| C | -4.15165 | 3.21406 | 1.81112 |
| H | -5.19983 | 3.25206 | 2.06953 |
| C | 3.90403 | 3.07216 | 0.42196 |

| | | | |
|---|---|---|---|
| C | -0.06928 | 3.57490 | -2.22404 |
| H | 0.89920 | 3.49654 | -1.75202 |
| C | -0.20310 | -5.78751 | 0.40847 |
| H | -1.04690 | -5.52583 | -0.23899 |
| C | 0.81460 | -4.48671 | -2.80507 |
| H | 1.02606 | -5.43332 | -2.32343 |
| C | -3.64023 | -1.03882 | -1.05540 |
| H | -4.64482 | -1.43449 | -1.04151 |
| C | 0.89781 | -4.37975 | -4.19261 |
| H | 1.16288 | -5.24785 | -4.78595 |
| C | -8.21869 | 0.18298 | 0.78088 |
| C | 1.60122 | 4.64208 | 1.77704 |
| H | 2.51615 | 5.14784 | 2.04892 |
| C | 0.31864 | 4.98165 | 2.17797 |
| H | 0.03653 | 5.80360 | 2.81979 |
| C | -2.55273 | -1.58081 | -1.67996 |
| H | -2.51944 | -2.48622 | -2.26696 |
| C | 0.64054 | -3.15863 | -4.80842 |
| H | 0.70010 | -3.06297 | -5.88683 |
| C | -2.28696 | 2.95878 | -2.21610 |
| H | -3.07247 | 2.38911 | -1.74153 |
| C | -0.24761 | 4.36612 | -3.35277 |
| H | 0.59527 | 4.90632 | -3.76476 |
| C | -5.46527 | 0.73441 | 0.50875 |
| C | -0.69410 | -5.69597 | 1.86578 |

| | | | |
|---|---|---|---|
| H | -1.01519 | -4.67059 | 2.07765 |
| H | 0.14711 | -5.91643 | 2.52804 |
| C | -1.51186 | 4.44626 | -3.92758 |
| H | -1.68395 | 5.05462 | -4.80757 |
| C | -2.54994 | 3.72757 | -3.34398 |
| H | -3.55361 | 3.75516 | -3.74880 |
| C | -3.17391 | 4.12446 | 2.18757 |
| H | -3.31068 | 5.00553 | 2.79754 |
| C | -6.35422 | 0.88124 | -0.55732 |
| C | 0.19231 | -7.22735 | 0.03873 |
| H | 0.48554 | -7.26709 | -1.01522 |
| H | 1.06010 | -7.52466 | 0.62920 |
| C | -7.71021 | 0.60899 | -0.43836 |
| C | -1.84630 | -6.67731 | 2.12969 |
| H | -2.72379 | -6.37386 | 1.54370 |
| H | -2.14304 | -6.62602 | 3.18218 |
| C | -0.97436 | -8.19393 | 0.29841 |
| H | -1.80775 | -7.95336 | -0.37480 |
| H | -0.66478 | -9.21581 | 0.05692 |
| C | -1.46268 | -8.11453 | 1.75161 |
| H | -0.66501 | -8.46366 | 2.41972 |
| H | -2.31471 | -8.78564 | 1.90183 |
| O | 2.42062 | -5.76804 | 1.41182 |
| C | 2.09670 | -4.86663 | 0.63165 |
| N | 2.98357 | -3.88220 | 0.26338 |

| | | | |
|---|---|---|---|
| H | 2.77525 | -3.30864 | -0.53946 |
| C | 4.79925 | -2.41855 | 1.03938 |
| H | 4.12616 | -1.98776 | 1.78624 |
| H | 4.68701 | -1.81408 | 0.13029 |
| C | 6.78698 | -4.46378 | 0.23170 |
| H | 6.88971 | -5.06330 | 1.14539 |
| H | 7.45161 | -4.91443 | -0.51220 |
| C | 4.36693 | -3.86125 | 0.73657 |
| H | 4.37256 | -4.43766 | 1.66427 |
| C | 6.25609 | -2.34735 | 1.51945 |
| H | 6.53939 | -1.30226 | 1.67872 |
| H | 6.33978 | -2.84620 | 2.49350 |
| C | 7.21447 | -3.01901 | 0.52606 |
| H | 7.22335 | -2.44726 | -0.41095 |
| H | 8.23753 | -2.99894 | 0.91527 |
| C | 5.33221 | -4.53328 | -0.25678 |
| H | 5.24730 | -4.02815 | -1.22800 |
| H | 5.02593 | -5.57276 | -0.40889 |
| O | -0.44932 | 0.53715 | 1.55223 |
| O | -0.44111 | 1.17508 | 2.84901 |
| H | -1.36435 | 1.44560 | 2.94858 |
| H | 0.84276 | -2.67191 | -0.01321 |
| H | -0.70769 | -3.41348 | -0.22734 |

**Appendix 23 Optimized Cartesian Coordinates of 3e-T.**

**ΔGsoln= -3911.956 Hartree**

| | | | |
|---|---|---|---|
| Co | -0.71255 | -1.72510 | -0.06355 |
| F | 6.26184 | -5.67972 | 0.56638 |
| F | 3.66859 | -5.01110 | 0.84588 |
| F | -5.21071 | -0.12070 | -2.78234 |
| F | 7.95816 | -4.05746 | -0.82016 |
| F | -7.84867 | 0.36125 | -3.03129 |
| F | 4.42701 | -1.09135 | -1.71760 |
| F | -9.52211 | -0.03086 | -0.91573 |
| F | 7.02372 | -1.75569 | -1.94675 |
| N | -1.85859 | -0.35898 | 0.64687 |
| N | 0.90765 | -1.03448 | 0.69502 |
| F | -5.89688 | -1.43946 | 1.72188 |
| N | -2.16789 | -2.54582 | -0.93698 |
| N | 0.22925 | -3.13988 | -0.88243 |
| N | -1.03590 | -2.92324 | 1.64216 |
| N | 0.75191 | 4.76783 | -0.06973 |
| F | -8.52820 | -0.92795 | 1.45913 |
| C | 3.09383 | -0.71355 | 1.25453 |
| H | 4.16006 | -0.86771 | 1.32453 |
| C | 2.17749 | -1.53517 | 0.50238 |
| C | 2.36554 | 0.27314 | 1.85893 |
| H | 2.73637 | 1.05788 | 2.50004 |

| | | | |
|---|---|---|---|
| C | 0.98283 | 0.07585 | 1.49140 |
| C | -0.10623 | 0.90363 | 1.83818 |
| C | 0.42550 | 3.37832 | 2.03010 |
| C | 1.54947 | -3.43855 | -0.96905 |
| C | 0.28712 | 3.50752 | 0.51400 |
| C | 2.51206 | -2.65725 | -0.28748 |
| C | 0.19739 | 2.13055 | 2.64986 |
| C | 0.29071 | 2.03796 | 4.04268 |
| H | 0.11587 | 1.07693 | 4.51409 |
| C | -1.43547 | 0.68756 | 1.42145 |
| C | -3.48114 | -2.22515 | -1.04076 |
| C | -0.50230 | -3.98859 | -1.65740 |
| C | -3.99987 | -1.08564 | -0.38207 |
| C | 4.46568 | -4.19359 | 0.13607 |
| C | 5.80089 | -4.55180 | 0.00725 |
| C | 4.84796 | -2.22500 | -1.13121 |
| C | -3.20799 | -0.21715 | 0.40038 |
| C | -1.88760 | -3.65350 | -1.67875 |
| C | -7.35920 | -0.08246 | -1.86481 |
| C | 6.18948 | -2.55471 | -1.26477 |
| C | 6.66831 | -3.72623 | -0.69476 |
| C | -6.00236 | -0.33902 | -1.71939 |
| C | -4.09407 | -3.21360 | -1.88952 |
| H | -5.13808 | -3.25254 | -2.16404 |
| C | 3.94741 | -3.02481 | -0.42418 |

| | | | |
|---|---|---|---|
| C | -0.02978 | -3.61942 | 2.19454 |
| H | 0.93732 | -3.52550 | 1.72220 |
| C | -0.29369 | 5.77162 | -0.38729 |
| H | -1.13001 | 5.50198 | 0.26657 |
| C | 0.75166 | 4.48136 | 2.82207 |
| H | 0.94679 | 5.43160 | 2.34066 |
| C | -3.65630 | 0.97716 | 1.07545 |
| H | -4.66666 | 1.35801 | 1.06794 |
| C | 0.84343 | 4.37367 | 4.20903 |
| H | 1.09845 | 5.24481 | 4.80232 |
| C | -8.21352 | -0.27782 | -0.78861 |
| C | 1.67163 | -4.57618 | -1.84280 |
| H | 2.59413 | -5.06416 | -2.12152 |
| C | 0.39592 | -4.91547 | -2.26651 |
| H | 0.12817 | -5.72008 | -2.93580 |
| C | -2.57470 | 1.52909 | 1.70327 |
| H | -2.55374 | 2.42862 | 2.29975 |
| C | 0.60765 | 3.14798 | 4.82438 |
| H | 0.67418 | 3.05183 | 5.90233 |
| C | -2.25369 | -3.02199 | 2.19815 |
| H | -3.04452 | -2.45292 | 1.73136 |
| C | -0.19997 | -4.42781 | 3.31229 |
| H | 0.64790 | -4.96671 | 3.71557 |
| C | -5.45388 | -0.79641 | -0.52027 |
| C | -0.79371 | 5.67144 | -1.84096 |

| | | | |
|---|---|---|---|
| H | -1.10482 | 4.64210 | -2.04856 |
| H | 0.04031 | 5.89990 | -2.50956 |
| C | -1.46274 | -4.52620 | 3.88756 |
| H | -1.62830 | -5.14805 | 4.75930 |
| C | -2.50806 | -3.80841 | 3.31571 |
| H | -3.51072 | -3.85030 | 3.72176 |
| C | -3.10106 | -4.09628 | -2.28610 |
| H | -3.21703 | -4.95400 | -2.93255 |
| C | -6.34451 | -0.98648 | 0.53738 |
| C | 0.08826 | 7.21648 | -0.02325 |
| H | 0.38827 | 7.26162 | 1.02858 |
| H | 0.94866 | 7.52208 | -0.62025 |
| C | -7.70392 | -0.73123 | 0.42027 |
| C | -1.95864 | 6.63926 | -2.09878 |
| H | -2.82854 | 6.32733 | -1.50596 |
| H | -2.26220 | 6.58237 | -3.14902 |
| C | -1.09078 | 8.16956 | -0.27690 |
| H | -1.91673 | 7.92138 | 0.40267 |
| H | -0.79081 | 9.19534 | -0.03987 |
| C | -1.58837 | 8.08148 | -1.72646 |
| H | -0.79936 | 8.43794 | -2.40094 |
| H | -2.44884 | 8.74274 | -1.87213 |
| O | 2.32260 | 5.77886 | -1.40973 |
| C | 2.01438 | 4.87553 | -0.62550 |
| N | 2.91432 | 3.90076 | -0.26287 |

| | | | |
|---|---|---|---|
| H | 2.71978 | 3.32896 | 0.54474 |
| C | 4.74078 | 2.45325 | -1.04339 |
| H | 4.06618 | 2.00843 | -1.78055 |
| H | 4.64455 | 1.85471 | -0.12848 |
| C | 6.71061 | 4.52983 | -0.27244 |
| H | 6.79651 | 5.12293 | -1.19204 |
| H | 7.37689 | 4.99506 | 0.46092 |
| C | 4.29369 | 3.89307 | -0.74831 |
| H | 4.28400 | 4.46161 | -1.68084 |
| C | 6.19367 | 2.39605 | -1.53711 |
| H | 6.48835 | 1.35327 | -1.69019 |
| H | 6.26147 | 2.88750 | -2.51615 |
| C | 7.15336 | 3.08814 | -0.55915 |
| H | 7.17895 | 2.52451 | 0.38246 |
| H | 8.17260 | 3.07763 | -0.95862 |
| C | 5.26004 | 4.58504 | 0.23017 |
| H | 5.19116 | 4.08659 | 1.20610 |
| H | 4.94239 | 5.62179 | 0.37718 |
| O | -0.39770 | -0.51096 | -1.55420 |
| O | -0.40000 | -0.96359 | -2.76147 |
| H | 0.78958 | 2.66867 | 0.02794 |
| H | -0.76796 | 3.39275 | 0.25126 |

**Appendix 24 Optimized Cartesian Coordinates of 3f-T.**

**ΔGsoln= -3911.762 Hartree**

| | | | |
|---|---|---|---|
| Co | -0.76328 | -1.81564 | 0.04223 |
| F | 6.25243 | -5.74336 | 0.36718 |
| F | 3.65095 | -5.12734 | 0.71549 |
| F | -5.06647 | 0.24242 | -2.65900 |
| F | 7.89880 | -4.03234 | -0.96838 |
| F | -7.67892 | 0.85481 | -2.97520 |
| F | 4.31194 | -1.07630 | -1.65412 |
| F | -9.47609 | 0.21866 | -1.03027 |
| F | 6.91678 | -1.69577 | -1.96890 |
| N | -1.87598 | -0.37538 | 0.65663 |
| N | 0.87586 | -1.08459 | 0.69864 |
| F | -6.03549 | -1.65893 | 1.56636 |
| N | -2.24028 | -2.61460 | -0.82752 |
| N | 0.15715 | -3.25881 | -0.74822 |
| N | -1.06535 | -2.86246 | 1.75955 |
| N | 0.84283 | 4.74153 | -0.07008 |
| F | -8.64587 | -1.03878 | 1.23923 |
| C | 3.05159 | -0.82649 | 1.32026 |
| H | 4.10954 | -1.01639 | 1.41211 |
| C | 2.12601 | -1.61222 | 0.54815 |
| C | 2.34044 | 0.18320 | 1.90652 |
| H | 2.71765 | 0.95822 | 2.55452 |

| | | | |
|---|---|---|---|
| C | 0.96702 | 0.02644 | 1.50622 |
| C | -0.10281 | 0.87957 | 1.82253 |
| C | 0.47528 | 3.34240 | 2.01463 |
| C | 1.49057 | -3.49349 | -0.92930 |
| C | 0.37560 | 3.47565 | 0.49543 |
| C | 2.45584 | -2.73863 | -0.26932 |
| C | 0.21128 | 2.10281 | 2.63396 |
| C | 0.26724 | 2.00036 | 4.02721 |
| H | 0.06298 | 1.04290 | 4.49319 |
| C | -1.44141 | 0.68017 | 1.41398 |
| C | -3.54134 | -2.21255 | -1.00701 |
| C | -0.55468 | -3.96687 | -1.64947 |
| C | -4.03308 | -1.08237 | -0.35802 |
| C | 4.42948 | -4.26803 | 0.03928 |
| C | 5.76758 | -4.59811 | -0.12741 |
| C | 4.76462 | -2.22939 | -1.13740 |
| C | -3.22037 | -0.21670 | 0.42750 |
| C | -1.94820 | -3.61765 | -1.67625 |
| C | -7.26800 | 0.23625 | -1.86189 |
| C | 6.10641 | -2.53536 | -1.31168 |
| C | 6.60963 | -3.72632 | -0.80445 |
| C | -5.92886 | -0.08559 | -1.68551 |
| C | -4.12193 | -3.09472 | -1.99061 |
| H | -5.14281 | -3.06080 | -2.33903 |
| C | 3.89134 | -3.07952 | -0.45597 |

| | | | |
|---|---|---|---|
| C | -0.05797 | -3.53241 | 2.34522 |
| H | 0.91143 | -3.46485 | 1.87572 |
| C | -0.20430 | 5.74204 | -0.39523 |
| H | -1.04233 | 5.47333 | 0.25701 |
| C | 0.80374 | 4.43631 | 2.81742 |
| H | 1.02715 | 5.38300 | 2.34189 |
| C | -3.65468 | 0.98496 | 1.08400 |
| H | -4.66211 | 1.37136 | 1.07862 |
| C | 0.85967 | 4.32484 | 4.20577 |
| H | 1.11695 | 5.19045 | 4.80562 |
| C | -8.18571 | -0.08618 | -0.87112 |
| C | 1.60911 | -4.51492 | -1.95349 |
| H | 2.53456 | -4.94309 | -2.30748 |
| C | 0.34490 | -4.80108 | -2.40066 |
| H | 0.06596 | -5.49690 | -3.17688 |
| C | -2.55965 | 1.53792 | 1.69247 |
| H | -2.52092 | 2.44699 | 2.27180 |
| C | 0.58581 | 3.10458 | 4.81538 |
| H | 0.62460 | 3.00663 | 5.89409 |
| C | -2.28744 | -2.93696 | 2.31437 |
| H | -3.08133 | -2.39631 | 1.82254 |
| C | -0.23308 | -4.28601 | 3.49772 |
| H | 0.61471 | -4.80431 | 3.92618 |
| C | -5.47105 | -0.72899 | -0.53749 |
| C | -0.69858 | 5.63736 | -1.85042 |

| | | | |
|---|---|---|---|
| H | -1.00841 | 4.60739 | -2.05727 |
| H | 0.13780 | 5.86501 | -2.51619 |
| C | -1.49689 | -4.35809 | 4.07346 |
| H | -1.66427 | -4.93837 | 4.97264 |
| C | -2.54126 | -3.67011 | 3.46550 |
| H | -3.54535 | -3.69340 | 3.86823 |
| C | -3.13232 | -3.95213 | -2.41614 |
| H | -3.21724 | -4.72712 | -3.16250 |
| C | -6.41688 | -1.03932 | 0.43761 |
| C | 0.17641 | 7.18756 | -0.03350 |
| H | 0.47270 | 7.23542 | 1.01925 |
| H | 1.03866 | 7.49224 | -0.62843 |
| C | -7.76045 | -0.72652 | 0.28547 |
| C | -1.86317 | 6.60394 | -2.11451 |
| H | -2.73493 | 6.29332 | -1.52378 |
| H | -2.16299 | 6.54401 | -3.16560 |
| C | -1.00179 | 8.13981 | -0.29406 |
| H | -1.82987 | 7.89381 | 0.38363 |
| H | -0.70227 | 9.16613 | -0.05921 |
| C | -1.49441 | 8.04728 | -1.74502 |
| H | -0.70331 | 8.40199 | -2.41790 |
| H | -2.35454 | 8.70780 | -1.89538 |
| O | 2.41563 | 5.74274 | -1.41611 |
| C | 2.11076 | 4.85359 | -0.61651 |
| N | 3.01482 | 3.89417 | -0.22317 |

| | | | |
|---|---|---|---|
| H | 2.82986 | 3.36929 | 0.61793 |
| C | 4.85501 | 2.43747 | -0.95099 |
| H | 4.18650 | 1.96585 | -1.67694 |
| H | 4.76001 | 1.86683 | -0.01790 |
| C | 6.80755 | 4.54772 | -0.23088 |
| H | 6.89531 | 5.11528 | -1.16620 |
| H | 7.46662 | 5.03740 | 0.49292 |
| C | 4.39729 | 3.88256 | -0.70095 |
| H | 4.38819 | 4.42304 | -1.65002 |
| C | 6.31023 | 2.37600 | -1.43705 |
| H | 6.61238 | 1.33152 | -1.56108 |
| H | 6.37847 | 2.84110 | -2.42880 |
| C | 7.26075 | 3.10133 | -0.47445 |
| H | 7.28552 | 2.56450 | 0.48272 |
| H | 8.28186 | 3.08629 | -0.86879 |
| C | 5.35379 | 4.60885 | 0.26190 |
| H | 5.28246 | 4.13864 | 1.25164 |
| H | 5.02950 | 5.64746 | 0.37699 |
| O | -0.73557 | -0.88260 | -1.68425 |
| O | -0.26832 | -1.51689 | -2.69306 |
| H | 0.90372 | 2.64696 | 0.01867 |
| H | -0.67140 | 3.35150 | 0.20510 |

**Appendix 25 Optimized Cartesian Coordinates of TS(3-TS).**

**ΔGsoln= -3987.0449 Hartree**

| | | | |
|---|---|---|---|
| Co | 1.44054 | 1.25018 | 0.05625 |
| F | -4.61833 | 6.46094 | 0.73969 |
| F | -2.23433 | 5.24090 | 1.04244 |
| F | 5.74637 | -0.98527 | -2.58626 |
| F | -6.53006 | 5.35824 | -0.85959 |
| F | 8.25771 | -1.95389 | -2.71110 |
| F | -3.64415 | 1.79797 | -1.89800 |
| F | 9.79540 | -2.07275 | -0.46441 |
| F | -6.03031 | 3.02019 | -2.16745 |
| N | 2.25381 | -0.36622 | 0.70327 |
| N | -0.33894 | 0.79707 | 0.57547 |
| F | 6.29610 | -0.20934 | 2.05851 |
| O | -0.27175 | -2.71633 | -0.95931 |
| N | 3.03750 | 1.76704 | -0.79264 |
| N | 0.83416 | 2.87741 | -0.70095 |
| N | 1.81047 | 2.18292 | 1.81284 |
| N | -2.47403 | -3.26639 | -0.98142 |
| F | 8.79853 | -1.19980 | 1.91731 |
| C | -2.55504 | 0.91831 | 1.09506 |
| H | -3.55856 | 1.30551 | 1.18491 |
| C | -1.46431 | 1.57921 | 0.42623 |
| C | -2.07440 | -0.25645 | 1.60657 |

| | | | |
|---|---|---|---|
| H | -2.60384 | -0.97298 | 2.21488 |
| C | -0.67774 | -0.33760 | 1.26484 |
| C | 0.19126 | -1.40766 | 1.54384 |
| C | -1.14463 | -3.51450 | 1.10325 |
| C | -0.39879 | 3.42602 | -0.83565 |
| C | -1.27075 | -3.12131 | -0.34730 |
| C | -1.53045 | 2.80983 | -0.26761 |
| C | -0.41573 | -2.69729 | 2.00014 |
| C | -0.24824 | -3.13404 | 3.31657 |
| H | 0.29115 | -2.50055 | 4.01107 |
| C | 1.57383 | -1.40419 | 1.27832 |
| C | 4.29746 | 1.22667 | -0.80793 |
| C | 1.75059 | 3.59833 | -1.40631 |
| C | 4.55035 | -0.00987 | -0.18561 |
| C | -3.14617 | 4.67419 | 0.23438 |
| C | -4.36982 | 5.31432 | 0.09315 |
| C | -3.85748 | 2.94177 | -1.22583 |
| C | 3.55547 | -0.75974 | 0.48085 |
| C | 3.03088 | 2.99175 | -1.42803 |
| C | 7.76775 | -1.52136 | -1.54181 |
| C | -5.09090 | 3.55950 | -1.37924 |
| C | -5.34686 | 4.75288 | -0.71748 |
| C | 6.47705 | -1.01656 | -1.46031 |
| C | 5.13219 | 2.15337 | -1.50785 |
| H | 6.18912 | 2.03232 | -1.69261 |

| | | | |
|---|---|---|---|
| C | -2.85270 | 3.47498 | -0.41634 |
| C | 0.91116 | 3.00926 | 2.37453 |
| H | -0.02833 | 3.13050 | 1.85773 |
| C | -2.53394 | -3.11545 | -2.46774 |
| H | -1.70985 | -2.44492 | -2.70769 |
| C | -1.63060 | -4.75399 | 1.53603 |
| H | -2.15306 | -5.39966 | 0.84204 |
| C | 3.70896 | -2.09762 | 0.99720 |
| H | 4.62050 | -2.67510 | 0.97284 |
| C | -1.43272 | -5.17981 | 2.84704 |
| H | -1.80825 | -6.14643 | 3.16103 |
| C | 8.55211 | -1.58650 | -0.39836 |
| C | -0.25238 | 4.61219 | -1.64512 |
| H | -1.05037 | 5.27937 | -1.93515 |
| C | 1.07860 | 4.71571 | -1.99633 |
| H | 1.53184 | 5.47946 | -2.61048 |
| C | 2.49425 | -2.49804 | 1.47364 |
| H | 2.24563 | -3.45482 | 1.90457 |
| C | -0.75149 | -4.36221 | 3.74305 |
| H | -0.60187 | -4.67968 | 4.76853 |
| C | 2.99634 | 2.02122 | 2.42626 |
| H | 3.70149 | 1.35404 | 1.95476 |
| C | 1.15788 | 3.68599 | 3.56140 |
| H | 0.39450 | 4.33530 | 3.96979 |
| C | 5.93044 | -0.56267 | -0.25920 |

| | | | |
|---|---|---|---|
| C | -3.83369 | -2.44069 | -2.92681 |
| H | -3.94789 | -1.47481 | -2.42616 |
| H | -4.69519 | -3.05841 | -2.65071 |
| C | 2.38358 | 3.51601 | 4.19591 |
| H | 2.60536 | 4.03275 | 5.12177 |
| C | 3.31849 | 2.66869 | 3.61144 |
| H | 4.28954 | 2.50226 | 4.05916 |
| C | 4.34804 | 3.23686 | -1.89101 |
| H | 4.67918 | 4.11082 | -2.43197 |
| C | 6.74886 | -0.63960 | 0.86852 |
| C | -2.29603 | -4.45220 | -3.18849 |
| H | -1.34284 | -4.87583 | -2.85875 |
| H | -3.08241 | -5.15489 | -2.89921 |
| C | 8.04061 | -1.14438 | 0.81416 |
| C | -3.82359 | -2.24915 | -4.45231 |
| H | -3.03372 | -1.53694 | -4.72144 |
| H | -4.76987 | -1.79995 | -4.76773 |
| C | -2.29697 | -4.25410 | -4.71191 |
| H | -1.43487 | -3.63868 | -4.99894 |
| H | -2.16859 | -5.22116 | -5.20741 |
| C | -3.58640 | -3.57348 | -5.19098 |
| H | -4.43690 | -4.24382 | -5.01368 |
| H | -3.54345 | -3.39894 | -6.27052 |
| O | -4.17479 | -4.80995 | -0.58913 |
| C | -3.70050 | -3.71506 | -0.33564 |

| | | | |
|---|---|---|---|
| N | -4.24489 | -2.79286 | 0.47525 |
| H | -3.79596 | -1.88822 | 0.52970 |
| C | -6.60258 | -2.07836 | 0.71124 |
| H | -6.76685 | -2.26093 | -0.35466 |
| H | -6.27046 | -1.03725 | 0.81140 |
| C | -6.56725 | -3.03287 | 3.51811 |
| H | -6.88680 | -4.07765 | 3.41716 |
| H | -6.38600 | -2.86334 | 4.58364 |
| C | -5.48907 | -3.00640 | 1.22429 |
| H | -5.77313 | -4.04235 | 1.02742 |
| C | -7.90419 | -2.28080 | 1.50142 |
| H | -8.66376 | -1.57894 | 1.14438 |
| H | -8.29175 | -3.28816 | 1.30418 |
| C | -7.68364 | -2.10993 | 3.01068 |
| H | -7.41459 | -1.06709 | 3.22157 |
| H | -8.61331 | -2.30990 | 3.55230 |
| C | -5.26255 | -2.82702 | 2.73387 |
| H | -4.88466 | -1.81384 | 2.92119 |
| H | -4.49278 | -3.52537 | 3.07384 |
| O | 1.56463 | 0.53154 | -1.68802 |
| O | 0.13751 | -0.38285 | -2.22510 |
| H | 0.10820 | -1.22241 | -1.69127 |
| H | 0.50777 | -0.62785 | -3.08549 |

**Appendix 26 Optimized Cartesian Coordinates of TS(4-TSC).**

**ΔGsoln= -3372.6221 Hartree**

| | | | |
|---|---|---|---|
| Co | 0.00399 | -0.60164 | 0.15910 |
| F | -7.24659 | -1.49331 | -2.85340 |
| F | -4.57860 | -1.28044 | -2.52357 |
| F | 5.09149 | -1.73716 | 2.02469 |
| F | -8.91362 | -1.31471 | -0.70367 |
| F | 7.76134 | -1.96450 | 1.72500 |
| F | -5.22021 | -0.71015 | 2.14073 |
| F | 8.93541 | -1.31374 | -0.64604 |
| F | -7.88736 | -0.91957 | 1.78838 |
| N | 1.41304 | 0.70072 | 0.16041 |
| N | -1.41608 | 0.68541 | 0.22088 |
| F | 4.73923 | -0.22344 | -2.45824 |
| N | 1.23880 | -2.02838 | 0.03845 |
| N | 0.03417 | -0.70111 | 2.20779 |
| N | -1.22117 | -2.03877 | 0.05832 |
| F | 7.40875 | -0.43938 | -2.72841 |
| C | -3.48060 | 1.64893 | 0.11318 |
| H | -4.55318 | 1.75138 | 0.04410 |
| C | -2.76117 | 0.39832 | 0.12499 |
| C | -2.56151 | 2.65600 | 0.18402 |
| H | -2.75454 | 3.71709 | 0.18109 |
| C | -1.25329 | 2.04646 | 0.23754 |

| | | | |
|---|---|---|---|
| C | -0.01315 | 2.71969 | 0.23450 |
| C | -0.46036 | 4.91857 | 1.38949 |
| C | -2.57094 | -2.07255 | -0.05312 |
| C | -3.33033 | -0.88357 | -0.00246 |
| C | -0.02457 | 4.21570 | 0.25765 |
| C | 0.39677 | 4.96381 | -0.84957 |
| H | 0.73173 | 4.44883 | -1.74329 |
| C | 1.23672 | 2.06079 | 0.18008 |
| C | 2.58929 | -2.05276 | -0.07785 |
| C | -0.69919 | -3.28609 | -0.13083 |
| C | 3.34067 | -0.85976 | -0.04659 |
| C | -5.37184 | -1.19081 | -1.43381 |
| C | -6.74096 | -1.30281 | -1.62814 |
| C | -5.69078 | -0.90201 | 0.89804 |
| C | 2.76097 | 0.42145 | 0.06489 |
| C | 0.72493 | -3.27987 | -0.13978 |
| C | 7.00961 | -1.53512 | 0.70278 |
| C | 0.12099 | 0.40836 | 4.33209 |
| H | 0.17136 | 1.34557 | 4.87107 |
| C | -7.06584 | -1.00717 | 0.73404 |
| C | -7.59180 | -1.21015 | -0.53460 |
| C | 5.63324 | -1.41308 | 0.83963 |
| C | 2.97266 | -3.42222 | -0.30083 |
| H | 3.98305 | -3.78222 | -0.42775 |
| C | -4.80650 | -0.98945 | -0.17609 |

| | | | |
|---|---|---|---|
| C | 0.09620 | 0.42189 | 2.94337 |
| H | 0.12665 | 1.35430 | 2.40161 |
| C | -0.47215 | 6.31092 | 1.41185 |
| H | -0.80790 | 6.82518 | 2.30701 |
| C | 3.46917 | 1.67608 | 0.05422 |
| H | 4.54211 | 1.78910 | 0.01168 |
| C | -0.05094 | 7.05760 | 0.30558 |
| C | 7.61105 | -1.20237 | -0.50328 |
| C | -2.94668 | -3.44349 | -0.27711 |
| H | -3.95596 | -3.81153 | -0.38899 |
| C | -1.78194 | -4.19333 | -0.32640 |
| H | -1.70149 | -5.25792 | -0.48939 |
| C | 2.53906 | 2.68006 | 0.12767 |
| H | 2.72827 | 3.74149 | 0.15212 |
| C | 0.38534 | 6.35691 | -0.82374 |
| H | 0.71815 | 6.90717 | -1.69826 |
| C | 4.81871 | -0.96373 | -0.19995 |
| C | -0.00493 | -1.87880 | 2.85377 |
| H | -0.05570 | -2.76502 | 2.24048 |
| C | 0.08055 | -0.81164 | 4.99795 |
| H | 0.09854 | -0.85460 | 6.08028 |
| C | 1.81342 | -4.18008 | -0.34008 |
| H | 1.73934 | -5.24543 | -0.50103 |
| C | 5.45827 | -0.64589 | -1.39746 |
| C | 6.83197 | -0.75379 | -1.56129 |

| | | | |
|---|---|---|---|
| C | 0.01645 | -1.97517 | 4.23914 |
| H | -0.01712 | -2.95215 | 4.70364 |
| H | -0.78479 | 4.36681 | 2.26496 |
| C | -0.09139 | 8.56678 | 0.32341 |
| H | -1.08543 | 8.93601 | 0.04806 |
| H | 0.13565 | 8.95916 | 1.31751 |
| H | 0.62305 | 8.99266 | -0.38424 |
| O | 0.02102 | -0.63602 | -1.77514 |
| O | -0.16281 | 0.70344 | -2.33537 |
| H | -0.93873 | 0.59545 | -2.99435 |
| H | 0.74294 | 0.95785 | -2.73856 |
| O | 2.16991 | 1.41441 | -3.08375 |
| H | 2.51542 | 1.91511 | -2.32727 |
| H | 2.78264 | 0.67412 | -3.18339 |
| O | -2.21627 | 0.33430 | -3.79082 |
| H | -2.70344 | 1.13305 | -4.02710 |
| H | -2.84429 | -0.21946 | -3.30654 |

**Appendix 27 Optimized Cartesian Coordinates of TS(3T-TS).**

**ΔGsoln= -3912.9728 Hartree**

| | | | |
|---|---|---|---|
| Co | -0.58225 | -1.84769 | -0.03586 |
| F | 6.58074 | -5.38430 | 0.65356 |
| F | 3.95290 | -4.83742 | 0.92182 |
| F | -5.01753 | 0.82036 | -1.95831 |
| F | 8.17534 | -3.75183 | -0.83517 |
| F | -7.66975 | 1.21741 | -2.23015 |
| F | 4.48563 | -1.02048 | -1.83702 |
| F | -9.43886 | -0.45549 | -1.00685 |
| F | 7.11376 | -1.56386 | -2.06918 |
| N | -1.74905 | -0.40121 | 0.47457 |
| N | 1.00662 | -1.04995 | 0.67682 |
| F | -5.88065 | -2.93473 | 0.80305 |
| N | -2.01972 | -2.75458 | -0.87108 |
| N | 0.41357 | -3.15663 | -0.93786 |
| N | -0.87507 | -2.87546 | 1.66483 |
| N | 0.41941 | 4.86978 | 0.02872 |
| F | -8.52963 | -2.53242 | 0.50502 |
| C | 3.18436 | -0.60694 | 1.17833 |
| H | 4.25787 | -0.70576 | 1.22455 |
| C | 2.29442 | -1.48811 | 0.46460 |
| C | 2.42172 | 0.35651 | 1.77461 |
| H | 2.76599 | 1.17830 | 2.38229 |
| C | 1.04493 | 0.08390 | 1.43967 |

| | | | |
|---|---|---|---|
| C | -0.07698 | 0.87006 | 1.78458 |
| C | 0.24576 | 3.37354 | 2.07209 |
| C | 1.75753 | -3.42296 | -0.99601 |
| C | 0.00284 | 3.57705 | 0.57690 |
| C | 2.67533 | -2.60493 | -0.31394 |
| C | 0.16800 | 2.08200 | 2.63608 |
| C | 0.36032 | 1.92450 | 4.01326 |
| H | 0.30235 | 0.92879 | 4.43884 |
| C | -1.38950 | 0.60542 | 1.35988 |
| C | -3.33042 | -2.44741 | -1.03196 |
| C | -0.29764 | -4.09309 | -1.65533 |
| C | -3.89254 | -1.30554 | -0.42282 |
| C | 4.70603 | -4.01896 | 0.16858 |
| C | 6.05727 | -4.31301 | 0.04392 |
| C | 4.97417 | -2.10138 | -1.20694 |
| C | -3.13454 | -0.38368 | 0.33198 |
| C | -1.69109 | -3.83379 | -1.63788 |
| C | -7.22130 | 0.19867 | -1.48534 |
| C | 6.32944 | -2.36839 | -1.33933 |
| C | 6.87262 | -3.48170 | -0.71224 |
| C | -5.85894 | -0.01915 | -1.33335 |
| C | -3.89559 | -3.43698 | -1.91751 |
| H | -4.92493 | -3.47754 | -2.24095 |
| C | 4.12733 | -2.91010 | -0.44787 |
| C | 0.15440 | -3.51936 | 2.24349 |

| | | | |
|---|---|---|---|
| H | 1.11842 | -3.42264 | 1.76751 |
| C | -0.65663 | 5.86479 | -0.20737 |
| H | -1.45624 | 5.56135 | 0.47689 |
| C | 0.52540 | 4.45847 | 2.90632 |
| H | 0.61075 | 5.44516 | 2.46872 |
| C | -3.64022 | 0.70931 | 1.11299 |
| H | -4.68063 | 0.98455 | 1.19590 |
| C | 0.71301 | 4.28904 | 4.27668 |
| H | 0.92809 | 5.14836 | 4.90184 |
| C | -8.12499 | -0.65444 | -0.86642 |
| C | 1.90617 | -4.58991 | -1.81156 |
| H | 2.84210 | -5.06543 | -2.06373 |
| C | 0.64050 | -5.00104 | -2.21268 |
| H | 0.40812 | -5.85198 | -2.83527 |
| C | -2.58064 | 1.30909 | 1.73981 |
| H | -2.61556 | 2.14985 | 2.41540 |
| C | 0.62594 | 3.01731 | 4.83549 |
| H | 0.76938 | 2.87288 | 5.90022 |
| C | -2.09146 | -2.98207 | 2.22718 |
| H | -2.90288 | -2.46824 | 1.73478 |
| C | 0.00595 | -4.27761 | 3.39652 |
| H | 0.86887 | -4.77668 | 3.81754 |
| C | -5.35486 | -1.07275 | -0.56930 |
| C | -1.22403 | 5.80039 | -1.63808 |
| H | -1.52752 | 4.77239 | -1.86366 |

| | | | |
|---|---|---|---|
| H | -0.42678 | 6.06472 | -2.33762 |
| C | -1.25070 | -4.37768 | 3.98382 |
| H | -1.39645 | -4.95990 | 4.88553 |
| C | -2.31642 | -3.71575 | 3.38386 |
| H | -3.31609 | -3.76268 | 3.79548 |
| C | -2.87844 | -4.29230 | -2.29093 |
| H | -2.95110 | -5.13674 | -2.95977 |
| C | -6.29186 | -1.90877 | 0.03955 |
| C | -0.28001 | 7.30415 | 0.18238 |
| H | 0.07202 | 7.32316 | 1.21881 |
| H | 0.54403 | 7.64315 | -0.44693 |
| C | -7.65905 | -1.71398 | -0.09982 |
| C | -2.41609 | 6.75434 | -1.80923 |
| H | -3.25113 | 6.40836 | -1.18595 |
| H | -2.76867 | 6.72470 | -2.84516 |
| C | -1.48587 | 8.24293 | 0.01679 |
| H | -2.27310 | 7.95919 | 0.72764 |
| H | -1.19145 | 9.26606 | 0.27121 |
| C | -2.05234 | 8.19076 | -1.40905 |
| H | -1.30352 | 8.58259 | -2.10916 |
| H | -2.92996 | 8.84015 | -1.49159 |
| O | 1.90610 | 5.94947 | -1.35371 |
| C | 1.65081 | 5.01598 | -0.58726 |
| N | 2.57879 | 4.03846 | -0.30921 |
| H | 2.44718 | 3.46220 | 0.50830 |

| | | | |
|---|---|---|---|
| C | 4.42893 | 2.65918 | -1.15263 |
| H | 3.74135 | 2.17696 | -1.85375 |
| H | 4.39967 | 2.06989 | -0.22729 |
| C | 6.34255 | 4.82550 | -0.49488 |
| H | 6.36292 | 5.40962 | -1.42392 |
| H | 7.02052 | 5.32730 | 0.20273 |
| C | 3.93599 | 4.08321 | -0.85482 |
| H | 3.86251 | 4.63785 | -1.79300 |
| C | 5.85964 | 2.65556 | -1.71065 |
| H | 6.19078 | 1.62382 | -1.86466 |
| H | 5.86318 | 3.13617 | -2.69733 |
| C | 6.83195 | 3.39962 | -0.78467 |
| H | 6.92265 | 2.85010 | 0.16124 |
| H | 7.83195 | 3.42559 | -1.22937 |
| C | 4.91467 | 4.82729 | 0.07151 |
| H | 4.90954 | 4.33896 | 1.05488 |
| H | 4.56013 | 5.85149 | 0.22023 |
| O | -0.05820 | -1.23529 | -1.75039 |
| H | 0.47807 | 2.77089 | 0.01431 |
| H | -1.06747 | 3.46907 | 0.38135 |
| O | -0.73757 | 0.36982 | -2.02124 |
| H | -1.25342 | 0.35708 | -1.17547 |
| H | -1.36779 | 0.15395 | -2.72554 |

**Appendix 28 Optimized Cartesian Coordinates of TS(4-TSa).**

**ΔGsoln= - 3219.706729Hartree**

| | | | |
|---|---|---|---|
| Co | 0.02637 | -0.49871 | -0.13120 |
| F | -7.51539 | -1.99871 | -2.52663 |
| F | -4.83183 | -1.75370 | -2.53127 |
| F | 4.89253 | -1.69145 | 1.97695 |
| F | -8.95382 | -1.28400 | -0.32561 |
| F | 7.57733 | -1.93853 | 1.94863 |
| F | -4.99426 | -0.09363 | 1.90688 |
| F | 8.99108 | -1.27289 | -0.28405 |
| F | -7.67874 | -0.32827 | 1.88525 |
| N | 1.43491 | 0.78167 | -0.24035 |
| N | -1.40826 | 0.77813 | -0.16260 |
| F | 5.00638 | -0.12567 | -2.49524 |
| N | 1.25584 | -1.93357 | -0.18322 |
| N | -0.05136 | -0.61337 | 1.87494 |
| N | -1.20496 | -1.92478 | -0.39509 |
| F | 7.69184 | -0.36263 | -2.49963 |
| C | -3.45980 | 1.74918 | -0.38598 |
| H | -4.52726 | 1.85538 | -0.50489 |
| C | -2.75339 | 0.49739 | -0.28457 |
| C | -2.53334 | 2.75089 | -0.33503 |
| H | -2.71524 | 3.81150 | -0.40175 |

| | | | |
|---|---|---|---|
| C | -1.23590 | 2.13402 | -0.19944 |
| C | 0.01103 | 2.80268 | -0.15938 |
| C | -0.52262 | 4.99313 | 0.96971 |
| C | -2.57394 | -1.97849 | -0.30317 |
| C | -3.33387 | -0.79164 | -0.30680 |
| C | -0.00002 | 4.29772 | -0.12884 |
| C | 0.50105 | 5.04787 | -1.20067 |
| H | 0.89682 | 4.53563 | -2.07056 |
| C | 1.25612 | 2.15318 | -0.17572 |
| C | 2.60865 | -1.97453 | -0.25396 |
| C | -0.68252 | -3.19768 | -0.28983 |
| C | 3.37025 | -0.78417 | -0.24938 |
| C | -5.50533 | -1.40080 | -1.42492 |
| C | -6.88726 | -1.53095 | -1.44026 |
| C | -5.58848 | -0.55584 | 0.79368 |
| C | 2.79683 | 0.49978 | -0.22622 |
| C | 0.73949 | -3.19338 | -0.25318 |
| C | 6.93715 | -1.49517 | 0.85889 |
| C | 1.10790 | -0.71764 | 3.97030 |
| H | 2.05789 | -0.74943 | 4.48748 |
| C | -6.97132 | -0.67361 | 0.80184 |
| C | -7.62300 | -1.16414 | -0.32162 |
| C | 5.55528 | -1.36492 | 0.85465 |
| C | 2.98893 | -3.36067 | -0.33762 |
| H | 3.99967 | -3.73385 | -0.40929 |

| | | | |
|---|---|---|---|
| C | -4.81722 | -0.91005 | -0.31417 |
| C | 1.09139 | -0.65953 | 2.58372 |
| H | 2.01357 | -0.65210 | 2.02321 |
| C | -0.53859 | 6.38524 | 0.99505 |
| H | -0.94811 | 6.89603 | 1.86079 |
| C | 3.49084 | 1.75472 | -0.14988 |
| H | 4.56340 | 1.86956 | -0.11105 |
| C | -0.03558 | 7.13576 | -0.07371 |
| C | 7.66001 | -1.15367 | -0.27610 |
| C | -2.93600 | -3.35892 | -0.20447 |
| H | -3.94339 | -3.74163 | -0.13617 |
| C | -1.76674 | -4.10851 | -0.19661 |
| H | -1.68955 | -5.18300 | -0.12352 |
| C | 2.55946 | 2.75650 | -0.11510 |
| H | 2.75030 | 3.81524 | -0.04387 |
| C | 0.48091 | 6.44021 | -1.17230 |
| H | 0.86665 | 6.99420 | -2.02220 |
| C | 4.85483 | -0.90053 | -0.25899 |
| C | -1.21669 | -0.62967 | 2.54513 |
| H | -2.11930 | -0.58830 | 1.95586 |
| C | -0.09850 | -0.73730 | 4.66181 |
| H | -0.11704 | -0.78525 | 5.74378 |
| C | 1.82816 | -4.11370 | -0.33629 |
| H | 1.75055 | -5.18861 | -0.40178 |
| C | 5.61264 | -0.56956 | -1.38268 |

| | | | |
|---|---|---|---|
| C | 6.99579 | -0.68705 | -1.40228 |
| C | -1.28025 | -0.69456 | 3.93044 |
| H | -2.24751 | -0.70895 | 4.41546 |
| H | -0.91602 | 4.43738 | 1.81366 |
| C | -0.02860 | 8.64470 | -0.03146 |
| H | -0.06223 | 9.07093 | -1.03642 |
| H | -0.87948 | 9.03123 | 0.53406 |
| H | 0.88071 | 9.01839 | 0.45200 |
| O | 0.41175 | -0.10530 | -1.94613 |
| O | -0.60959 | -1.05957 | -2.96576 |
| H | -0.99708 | -1.57764 | -2.21235 |
| H | 0.05762 | -1.63176 | -3.37580 |

**Appendix 29 Optimized Cartesian Coordinates of TS(4-TSb).**

**ΔGsoln= -3428.942938 Hartree**

| | | | |
|---|---|---|---|
| Co | -0.10519 | -0.59359 | -0.22468 |
| F | 7.38347 | -1.65591 | 2.24591 |
| F | 4.70008 | -1.46450 | 2.15347 |
| F | -5.19902 | -1.76025 | -2.02710 |
| F | 8.86628 | -1.26968 | -0.01243 |
| F | -7.86555 | -1.99974 | -1.70746 |
| F | 4.94233 | -0.49668 | -2.48163 |
| F | -9.02558 | -1.34810 | 0.67016 |
| F | 7.62852 | -0.68724 | -2.36960 |
| N | -1.52042 | 0.70053 | -0.21130 |
| N | 1.30751 | 0.69610 | -0.33647 |
| F | -4.82205 | -0.23177 | 2.44885 |
| N | -1.32974 | -2.02479 | -0.06556 |
| N | -0.18052 | -0.71222 | -2.27008 |
| N | 1.12837 | -2.02301 | -0.14027 |
| F | -7.48820 | -0.45998 | 2.73888 |
| C | 3.37050 | 1.66677 | -0.33047 |
| H | 4.44448 | 1.77425 | -0.30823 |
| C | 2.65689 | 0.41287 | -0.30853 |
| C | 2.44582 | 2.67055 | -0.35699 |
| H | 2.63526 | 3.73226 | -0.36223 |
| C | 1.13866 | 2.05642 | -0.35394 |

| | | | |
|---|---|---|---|
| C | -0.10409 | 2.72455 | -0.32473 |
| C | 0.30679 | 4.92430 | -1.49347 |
| C | 2.48245 | -2.05713 | -0.11454 |
| C | 3.23734 | -0.86783 | -0.21637 |
| C | -0.10187 | 4.22054 | -0.35272 |
| C | -0.49899 | 4.96750 | 0.76472 |
| H | -0.80400 | 4.45108 | 1.66829 |
| C | -1.35054 | 2.06119 | -0.24804 |
| C | -2.67858 | -2.05629 | 0.06610 |
| C | 0.61730 | -3.27357 | 0.05590 |
| C | -3.43752 | -0.86824 | 0.02882 |
| C | 5.39437 | -1.27449 | 1.01613 |
| C | 6.77656 | -1.37663 | 1.08375 |
| C | 5.51517 | -0.78141 | -1.30051 |
| C | -2.86640 | 0.41500 | -0.10416 |
| C | -0.80638 | -3.27329 | 0.10424 |
| C | -7.10857 | -1.56365 | -0.69202 |
| C | -0.31428 | 0.38081 | -4.40050 |
| H | -0.37590 | 1.31394 | -4.94535 |
| C | 6.90000 | -0.87610 | -1.26089 |
| C | 7.53347 | -1.17571 | -0.06241 |
| C | -5.73382 | -1.43531 | -0.83921 |
| C | -3.05113 | -3.42740 | 0.29763 |
| H | -4.05769 | -3.79282 | 0.43871 |
| C | 4.72261 | -0.97249 | -0.16845 |

| | | | |
|---|---|---|---|
| C | -0.25770 | 0.40518 | -3.01294 |
| H | -0.27452 | 1.34203 | -2.47837 |
| C | 0.31413 | 6.31714 | -1.51584 |
| H | 0.63621 | 6.83243 | -2.41536 |
| C | -3.58053 | 1.66614 | -0.10282 |
| H | -4.65374 | 1.77452 | -0.05473 |
| C | -0.08823 | 7.06230 | -0.40209 |
| C | -7.70292 | -1.23051 | 0.51746 |
| C | 2.86961 | -3.43075 | 0.07069 |
| H | 3.88324 | -3.80065 | 0.11794 |
| C | 1.70974 | -4.18186 | 0.17948 |
| H | 1.63915 | -5.24861 | 0.33265 |
| C | -2.65540 | 2.67434 | -0.19212 |
| H | -2.85011 | 3.73454 | -0.22688 |
| C | -0.49189 | 6.36017 | 0.73896 |
| H | -0.79755 | 6.90956 | 1.62392 |
| C | -4.91400 | -0.97908 | 0.19319 |
| C | -0.15731 | -1.89492 | -2.90775 |
| H | -0.09387 | -2.77670 | -2.28930 |
| C | -0.29038 | -0.84428 | -5.05769 |
| H | -0.33314 | -0.89560 | -6.13896 |
| C | -1.88722 | -4.17896 | 0.32144 |
| H | -1.80529 | -5.24378 | 0.48207 |
| C | -5.54662 | -0.66083 | 1.39414 |
| C | -6.91849 | -0.77498 | 1.56852 |

| | | | |
|---|---|---|---|
| C | -0.21016 | -2.00187 | -4.29147 |
| H | -0.18813 | -2.98252 | -4.74887 |
| H | 0.62043 | 4.37367 | -2.37356 |
| C | -0.10953 | 8.57150 | -0.43828 |
| H | 0.65345 | 8.96457 | -1.11390 |
| H | -1.07880 | 8.94175 | -0.79010 |
| H | 0.05973 | 8.99575 | 0.55396 |
| O | -0.08180 | -0.61314 | 1.71408 |
| O | 0.05348 | 0.73580 | 2.27453 |
| H | 0.73483 | 0.62992 | 3.01325 |
| H | -0.88195 | 0.99165 | 2.61868 |
| O | -2.28363 | 1.45627 | 2.97312 |
| H | -2.64342 | 1.94642 | 2.21552 |
| H | -2.89874 | 0.72219 | 3.10128 |
| N | 1.99905 | 0.38884 | 4.04158 |
| C | 4.24316 | -0.15311 | 5.23801 |
| C | 2.99026 | 0.15096 | 4.57350 |
| H | 4.94874 | -0.55422 | 4.50888 |
| H | 4.65651 | 0.75387 | 5.68184 |
| H | 4.07478 | -0.89300 | 6.02209 |

**Computational Details for Gibbs Free energy calculation**

The free-energy profile for the complete catalytic cycle was constructed using DFT calculations. Within this framework, the final electron-transfer step involves formation of the Co-$O_2$ adduct, followed by $O_2$ release and regeneration of the catalyst. The potential associated with this step was therefore used as an estimate of the potential relevant to water oxidation. To estimate this voltage theoretically and to evaluate the corresponding free-energy change ($\Delta G_{redox}$), the redox potential of the Fc/Fc$^+$ couple was first computed using the same computational methodology and parameters, and the resulting value was subsequently referenced to the normal hydrogen electrode (NHE).

Determination of the redox potential for the specified reaction:

$$\mathbf{A_{sol} \longrightarrow B_{sol} + ne^-} \quad \mathbf{(1)}$$

The redox potential is determined using the following equation:

$$\mathbf{E^\circ_{sol} = -\frac{\Delta G^\circ_{rxn,sol}}{nF} = -\frac{\Delta G^\circ_{A\rightarrow B,sol} - \Delta G^\circ_{Fc/Fc+}}{nF}} \quad \mathbf{(2)}$$

Here, $\mathbf{\Delta G^\circ_{A\rightarrow B,\ sol}}$ represents the free-energy change of the redox couple in solution obtained from DFT calculations, while $\Delta G^\circ_{Fc/Fc+}$ denotes the free-energy change of the Fc/Fc$^+$ redox couple. F is the Faraday constant (23.06 kcal mol$^{-1}$ V$^{-1}$), and $n$ is the number of electrons transferred in the redox process. The resulting potential is then converted to the corresponding free-energy change referenced to the normal hydrogen electrode (NHE), then the NHE-referenced potential obtained from the terminal electron-transfer step was treated as the electrode potential and used consistently to evaluate the free-energy changes of all PCET steps along the catalytic cycle.

$$\mathbf{AH_{sol} = A_{sol} + H^+_{sol} + e^-} \quad \mathbf{(3)}$$

In the case of a proton-coupled electron transfer (PCET) process:

$$\Delta G^{o}_{H^{+},Solv} = \Delta G^{o}_{H^{+},gas} + \Delta G_{,solv} \quad (4)$$

To determine its redox potential relative to the normal hydrogen electrode (NHE), the experimentally established standard free energy of the proton $G^{\circ}_{H^{+}, solv}$ is employed: Here, $G^{\circ}_{H^{+}, gas}$ denotes the gas-phase Gibbs free energy of the proton (-6.3 kcal·mol$^{-1}$), while $\Delta G_{solv}$ represents the free energy of proton solvation in aqueous medium (-265.9 kcal·mol$^{-1}$). Accordingly, the standard free energy of the proton was taken as -272.2 kcal·mol$^{-1}$.[1]

**References:**

[1] Du, H.-Y.; Chen, S.-C.; Su, X.-J.; Jiao, L.; Zhang, M.-T. Redox-Active Ligand Assisted Multielectron Catalysis: A Case of CoIII Complex as Water Oxidation Catalyst. *J. Am. Chem. Soc.* **2018,** *140*, 1557–1565.
